# Protein–Protein Interaction-Guided Peptides Interfere with Early Zika Virus Entry-Associated Events

**DOI:** 10.3390/v18101066

**Published:** 2026-09-25

**Authors:** Carlos Andres Rodríguez-Salazar, Delia Piedad Recalde-Reyes, Santiago Linares-Jiménez, Carolina López-Cardoso, María Isabel Giraldo

**Affiliations:** 1 Molecular Biology and Virology Laboratory, Faculty of Medicine and Health Sciences, Corporación Universitaria Empresarial Alexander von Humboldt, Armenia 630003, Quindío, Colombia; drecalde5552@cue.edu.co (D.P.R.-R.); slinares290@cue.edu.co (S.L.-J.); clopez_233@cue.edu.co (C.L.-C.); 2 Department of Microbiology and Immunology, University of Texas Medical Branch, Galveston, TX 77555, USA

**Keywords:** Zika virus, antiviral agents, antimicrobial peptides, computational biology, viral fusion proteins, virus internalization

## Abstract

Zika virus (ZIKV) remains a public health concern because of its association with neurological complications and congenital disease, yet no specific antiviral therapy has been approved. Since the envelope (E) protein mediates viral attachment and early entry-associated membrane events, it represents an attractive antiviral target. Here, we used a protein–protein interaction-guided strategy to design peptides to interfere with predicted interfaces between the ZIKV E protein and cellular receptors involved in attachment. After in silico filtering and preliminary screening, six peptides (PZ1–PZ6) were selected. These peptides showed heterogeneous physicochemical profiles, low cytotoxicity and hemolytic activity under the tested conditions, and docking-predicted interactions with functional E protein regions. In HTR-8/SVneo cells, antiviral activity depended on the timing of exposure, with greater reductions in infectious titers during co-treatment and post-treatment. All peptides also reduced titers in anti-adsorption assays, supporting interference with viral attachment or early entry-associated events. Consistently, DiOC18 fluorescence signals were reduced in a peptide-specific manner, particularly for PZ3 and PZ6. Nuclease protection assays showed no extensive exposure of the viral genome, whereas transmission electron microscopy revealed ultrastructural alterations without generalized particle disruption. Overall, PZ1–PZ6 are promising protein–protein interaction-guided anti-ZIKV peptide candidates with activity against early viral entry-associated events.

## 1. Introduction

Zika virus (ZIKV) is an enveloped, positive-sense single-stranded RNA arbovirus of the family *Flaviviridae* and genus *Orthoflavivirus*, transmitted mainly by *Aedes* mosquitoes [1]. Although initially considered of limited clinical relevance after its identification in Uganda in 1947, outbreaks in Yap Island, French Polynesia, and the Americas revealed its association with neurological complications and congenital abnormalities [1,2,3]. Despite the decline in global incidence after 2017, ZIKV continues to circulate in tropical and subtropical regions, where vector expansion, urbanization, human mobility, climate change, and diagnostic cross-reactivity with dengue and other flaviviruses complicate surveillance and control [3,4]. Clinically, ZIKV infection is often mild or asymptomatic, but it can cause febrile illness and has been associated with Guillain–Barré syndrome and other neurological complications [5,6,7]. During pregnancy, ZIKV can infect placental cells and neural progenitors, leading to fetal growth restriction, microcephaly, and other manifestations of congenital Zika syndrome, including postnatal neurodevelopmental alterations [8,9,10].

The ZIKV genome is approximately 10.7 kb and encodes a single polyprotein that is processed into three structural proteins (capsid, premembrane/membrane, and envelope (E)) and seven non-structural proteins [1]. The ZIKV E protein mediates viral attachment, pH-dependent conformational rearrangements, and membrane fusion, making it a relevant target for entry-directed antiviral strategies [11,12]. No specific antiviral treatment or vaccine has been approved for ZIKV infection; therefore, clinical management remains supportive [11,13,14,15]. Although monoclonal antibodies, immunoglobulins, repurposed drugs, and viral protein inhibitors have been explored, none has become standard therapy [11,16,17]. In this context, antiviral peptides are promising candidates because they can interfere with protein–protein interactions, bind functional viral regions, or disrupt envelope-associated steps such as attachment and membrane fusion. Peptides derived from conserved viral regions, host proteins, or antimicrobial molecules have shown inhibitory activity against ZIKV and related flaviviruses [18,19,20]. However, many antiviral peptides still require further optimization because of limited potency, incomplete mechanistic characterization, or uncertain in vivo stability [21,22]. Therefore, a protein–protein interaction-guided strategy may help prioritize peptides derived from structurally defined virus–host interfaces involved in viral attachment or entry, rather than relying exclusively on conserved viral sequences, natural antimicrobial peptides, or empirical screening [23,24,25,26,27]. Based on this rationale, we evaluated peptides designed from predicted ZIKV E protein–host receptor interfaces and explored their ability to interfere with early viral entry-associated events in a human trophoblast cell model relevant to ZIKV infection during pregnancy.

## 2. Materials and Methods

### 2.1. In Silico Peptide Design

#### 2.1.1. Modeling of ZIKV Envelope Protein Interactions with Human Cellular Receptors

X-ray crystallographic structures with resolution better than 3 Å were retrieved from the Protein Data Bank (PDB). The ZIKV E protein structure (PDB: 5JHM) was used as the viral ligand, and a neutralizing anti-ZIKV antibody bound to E domain III (PDB: 5VIG) was included as a reference for inhibitory interfaces [16]. Representative C-type lectin receptors involved in flavivirus attachment [DC-SIGN/CD209 (PDB: 1XPH), MR/CD206 (PDB: 5XTS), and CLEC5A/MDL-1 (PDB: 2YHF)] were selected because they provide defined receptor-envelope interfaces, whereas TAM and TIM receptors were excluded because they primarily mediate entry through phosphatidylserine-associated ligands rather than direct E protein binding [12]. Glycan chains were not modeled because the objective of this analysis was to identify peptide fragments derived from protein–protein contacts between the ZIKV E ectodomain and selected receptor structures. Therefore, the predicted interfaces should be interpreted as protein-interface models and not as complete virion-surface models incorporating glycan-dependent effects. Docking was performed using ClusPro 2.0 (Boston University, Boston, MA, USA) [28], and balanced models were selected for interface analysis and peptide candidate identification.

#### 2.1.2. Selection of Inhibitory Peptides Derived from Protein–Protein Interactions

PeptiDerive from the Rosetta Online Server (ROSIE, RosettaCommons; Johns Hopkins University, Baltimore, MD, USA) was used to identify peptide fragments derived from receptor–virus interaction interfaces. Candidate peptides with interface energy scores below −10 Rosetta Energy Units (REU) and high relative interface score contributions were prioritized, because lower PeptiDerive interface energy scores identify fragments predicted to contribute more strongly to protein–protein binding at the modeled receptor–E protein interface [23,24,25]. Toxic sequences predicted by ToxinPred (Bioinformatics Centre, CSIR-Institute of Microbial Technology, Chandigarh, India) [29] were excluded, and the remaining candidates were evaluated for hydropathicity and predicted solubility using ProtParam (ExPASy/Swiss Institute of Bioinformatics, Lausanne, Switzerland) [30] and PepCalc (PepCalc version 49.3 Innovagen AB, Lund, Sweden). Based on these criteria, twenty peptides were selected for structural modeling with AlphaFold2 through the ColabFold Google Colab notebook (ColabFold; Mirdita et al., Max Planck Institute for Multidisciplinary Sciences, Göttingen, Germany) [31] and peptide–E protein docking using ClusPro 2.0 [28]. Balanced models with the lowest binding energies were analyzed and visualized using UCSF ChimeraX version 1.11.1 (University of California San Francisco, San Francisco, CA, USA) [32]. The same twenty peptides were synthesized and subjected to preliminary antiviral screening.

### 2.2. Peptide Synthesis

Peptides were custom-synthesized by Peptides 2.0 Inc. (Chantilly, VA, USA) using standard solid-phase peptide synthesis, purified to >95% by RP-HPLC, and verified by mass spectrometry.

### 2.3. Cell Lines and Virus Propagation

Vero cells were used for preliminary antiviral screening and plaque assay, C6/36 mosquito cells for ZIKV propagation, and HTR-8/SVneo trophoblast cells as a human placental model relevant to ZIKV infection. Vero, C6/36, and HTR-8/SVneo cells were cultured in DMEM, Leibovitz L-15, and RPMI-1640, (Gibco, Thermo Fisher Scientific, Waltham, MA, USA) respectively. Growth media were supplemented with 10% fetal bovine serum (FBS) (Eurobio Scientific, Les Ulis, France), 2 mM L-glutamine (Gibco, Thermo Fisher Scientific, Waltham, MA, USA), and 1× penicillin–streptomycin (PS) (Thermo Scientific, Thermo Fisher Scientific, Waltham, MA, USA); maintenance media contained 2% FBS with the same supplements. C6/36 medium was additionally supplemented with 10% tryptose phosphate broth (TPB) (Invitrogen, Thermo Fisher Scientific, Waltham, MA, USA). Vero and HTR-8/SVneo cells were maintained at 37 °C with 5% CO_2_, whereas C6/36 cells were incubated at 28 °C.

ZIKV stocks were propagated in C6/36 cells at a multiplicity of infection (MOI) of 0.1 for 5–6 days at 28 °C using maintenance medium. Viral supernatants were clarified by centrifugation at 5000× *g*, filtered through a 0.22 µm membrane, and stored at −80 °C until use. RT-qPCR confirmed viral identity.

### 2.4. Cell Viability and Hemolysis Assays

HTR-8/SVneo cells (1 × 10^4^ cells/well) were seeded in 96-well plates 24 h before the assay. Cells were washed with PBS (ChemCruz, Santa Cruz Biotechnology, Dallas, TX, USA), and 100 µL of maintenance medium containing two-fold serial dilutions of the peptides (25 to 400 µM) were added. Untreated cells were used as the viability control, while cells treated with 0.1% Triton X-100 (Sigma-Aldrich, St. Louis, MO, USA) served as a cell death control. Twenty-four hours post-incubation, cell viability was determined using the MTT assay [3-(4,5-dimethylthiazol-2-yl)-2,5-diphenyltetrazolium bromide] (Thermo Fisher Scientific, Waltham, MA, USA), following the manufacturer’s instructions; Absorbance was measured at 570 nm, with background correction at 630 nm.

For the hemolysis assays, human blood samples from healthy volunteers were collected into EDTA-K_3_ Vacutainer tubes (BD, Franklin Lakes, NJ, USA), following institutional ethical guidelines and informed consent. Samples were centrifuged at 400× *g* for 5 min, and erythrocytes were separated, diluted 1:20 in PBS, and washed four times. Erythrocyte suspensions were dispensed into 96-well polypropylene plates (90 µL/well) and incubated with two-fold serial dilutions of peptides (50–400 µM) for 24 h at 37 °C. The peptide vehicle was used as the negative control, and 0.1% Triton X-100 served as the hemolysis control. After centrifugation at 400× *g* for 5 min, supernatant absorbance was measured at 541 nm using an Epoch BioTek spectrophotometer (BioTek Instruments, Winooski, VT, USA). Hemolysis (%) was calculated as [(A_sample − A_negative control)/(A_Triton X-100 − A_negative control)] × 100.

### 2.5. Plaque Assay, Virus Neutralization Screening, and Anti-Adsorption Assay

Viral titers were determined by plaque assay in Vero cells seeded in 24-well plates at 1 × 10^5^ cells/well 24 h before infection. Viral inoculum from treated samples and controls were 10-fold serially diluted in DMEM, and 200 µL of each dilution was added to the cells. After 2 h of adsorption at 37 °C with gentle rocking every 15 min, the inoculum was removed and replaced with 1 mL of overlay medium containing DMEM, 2% carboxymethylcellulose (Invitrogen, Carlsbad, CA, USA), 2% FBS, and 1 × PS. Cells were incubated for 5 days, fixed with 4% formaldehyde (PanReac AppliChem, Castellar del Vallès, Barcelona, Spain) in PBS, stained with crystal violet (Invitrogen, Carlsbad, CA, USA), and plaques were counted. Viral titers were expressed as plaque-forming units per milliliter (PFU/mL).

For preliminary antiviral screening, Vero cells were seeded as described above. ZIKV (MOI = 1) was preincubated with peptides at different concentrations (200–5 µM) for 1 h at room temperature (RT) to allow virus–peptide interaction. Virus–peptide mixtures were then added to Vero cell monolayers for 1 h at 37 °C. After adsorption, cells were washed with PBS, overlaid, incubated for 5 days, fixed, and stained as described above.

For anti-adsorption assays, Vero cells were seeded in 48-well plates at 2.5 × 10^4^ cells/well 24 h before infection. Cells were incubated with ZIKV (MOI = 0.2) and peptides (100 µM) at 4 °C for 1 h to allow viral attachment while limiting entry. Cells were then washed twice with PBS to remove unbound virus and peptides, overlaid, incubated for 5 days at 37 °C, fixed, and stained as described above.

### 2.6. Time-of-Addition Antiviral Assay

To investigate the viral cycle stage affected by the peptides, pre-, co-, and post-infection treatments were performed in HTR-8/SVneo cells seeded in 24-well plates at 1 × 10^5^ cells/well 24 h before infection. For pre-treatment, cells were incubated with peptides at 100 µM for 1 h at 37 °C, washed, and then infected with ZIKV (MOI = 1) for 1 h. For co-treatment, peptides were mixed with ZIKV (MOI = 1) and immediately added to cells for 1 h at 37 °C. For post-treatment, cells were first infected with ZIKV (MOI = 1) for 1 h, after which the inoculum was removed, and peptides were added at 100 µM. After each treatment, cultures were maintained in 500 µL of maintenance medium. At 24 h post-infection, supernatants were collected and stored at −80 °C for viral titration (plaque assay), while cell monolayers were processed for total RNA extraction using the Isolate II RNA Mini Kit (Bioline Reagents Ltd., London, UK) for subsequent RT-qPCR analysis. Mock-infected and untreated infected cells served as controls.

### 2.7. DiOC18 Fluorescence Assay

HTR-8/SVneo cells were seeded on sterile round coverslips in 24-well plates at 1 × 10^5^ cells/well 24h before the assay. ZIKV stock (1 × 10^8^ PFU/mL) was labeled with 0.2 µM DiOC18 and purified using Amicon Ultra-15 centrifugal filters with a 100 kDa molecular weight cutoff (MilliporeSigma, Burlington, MA, USA), with three RPMI washes to remove unincorporated dye. Labeled virus was incubated with each peptide at 100 µM or with ammonium chloride (NH_4_Cl, 20 mM; PanReac AppliChem, Castellar del Vallès, Barcelona, Spain) as an inhibitory control of low-pH-dependent entry-associated events. Virus–peptide mixtures were added to cells at 4 °C for 30 min to allow viral binding, followed by incubation at 37 °C for 30 min to allow temperature-dependent internalization and early entry-associated membrane events. Cells were counterstained with Hoechst (Sigma-Aldrich, St. Louis, MO, USA), and images were acquired using an Axiolab V fluorescence microscope (Carl Zeiss Microscopy GmbH, Jena, Germany). DiOC18-positive signals were quantified using ImageJ version 1.54p (National Institutes of Health, Bethesda, MD, USA) [33] by measuring integrated green fluorescence intensity in 40× microscopic fields acquired under identical settings. Peptide effects were assessed relative to infected, mock-infected, and NH_4_Cl-treated controls.

### 2.8. Nuclease Protection Assay

To assess whether peptide treatment exposes the viral genome to nuclease digestion, approximately 5 × 10^6^ PFU-equivalents of ZIKV were incubated with peptides at 100 µM for 1 h at RT. Micrococcal nuclease (150 U; New England Biolabs, Ipswich, MA, USA) was then added for 45 min at 37 °C, and the reaction was stopped with 10 mM EDTA (AMRESCO, LLC, Solon, OH, USA). Control conditions included untreated virus, virus treated with nuclease alone, and virus treated with Triton X-100 as a positive control for envelope disruption. Viral RNA was extracted and analyzed by RT-qPCR as described below.

### 2.9. Transmission Electron Microscopy (TEM)

ZIKV stock containing 2.25 × 10^8^ PFU was concentrated and buffer-exchanged into HBS buffer (20 mM HEPES (Sigma-Aldrich, St. Louis, MO, USA), 150 mM NaCl (EMSURE^®^, Merck KGaA, Darmstadt, Germany), pH 7.4) using Amicon Ultra-15 centrifugal filters with a 100 kDa molecular weight cutoff (Millipore) at 4000× *g* for 10 min. Eight conditions were prepared before fixation: six peptide-treated samples (100 µM), one virus-only control, and one acid + heat control. The acid + heat control consisted of virus incubated in acidified HBS (pH 5.6–5.8) followed by 20 min at 37 °C and 20 min at 45 °C to induce flaviviral envelope rearrangements. All conditions were incubated for 60 min at RT without post-treatment washing to preserve potential virus–peptide complexes. Samples were fixed with 2% glutaraldehyde (Sigma-Aldrich, St. Louis, MO, USA) in HBS and transported at RT to the Transmission Electron Microscopy Facility at Universidad El Bosque, Colombia.

### 2.10. RNA Extraction, Reverse Transcription, and Quantitative PCR

Total RNA was extracted from cell culture supernatants using the PureLink Viral DNA/RNA Mini Kit (Invitrogen/Thermo Fisher Scientific, Waltham, MA, USA) and from corresponding infected cell monolayers using the Isolate II RNA Mini Kit, after removal of the supernatant. Relative ZIKV RNA levels were determined by one-step SYBR Green RT-qPCR using Luna Universal One-Step RT-qPCR Mix (New England Biolabs, Ipswich, MA, USA) on a Bio-Rad CFX96 Opus instrument (Bio-Rad Laboratories, Hercules, CA, USA). ZIKV was amplified using the primers Forward 5′-CCGCTGCCCAACACAAG-3′ and Reverse 5′-CCACTAACGTTCTTTTGCAGACAT-3′. For cell-associated RNA, levels were normalized to hGAPDH, using primers Forward 5′-TGTTGCCATCAATGACCCCTT-3′ and Reverse 5′-CTCCACGACGTACTCAGCG-3′.

### 2.11. Statistical Analysis

Unless otherwise specified, experiments were performed in three independent biological replicates. Technical replicates were averaged within each independent experiment before statistical analysis, so that each biological replicate represented one independent experimental unit. Specific technical replicate numbers are indicated in the corresponding figure legends. Data are presented as mean ± SD. Normality was assessed using the Shapiro–Wilk test, and all datasets analyzed statistically met the assumptions for parametric testing. Within each treatment condition, groups were compared by repeated-measures one-way ANOVA followed by Dunnett’s multiple-comparisons test against the corresponding reference control. RT-qPCR analyses were performed using ΔCt values, while relative RNA levels are displayed as 2^−ΔΔCt^. GraphPad Prism 10 GraphPad Prism version 10 (GraphPad Software, Boston, MA, USA) was used for all statistical analyses; *p* < 0.05 was considered statistically significant.

## 3. Results

### 3.1. Characterization and Docking Analysis of Selected Anti-ZIKV Peptides

Twenty peptides were synthesized for preliminary in vitro screening after in silico prioritization. Although most showed acceptable cytotoxicity and hemolysis profiles under the tested conditions, only six significantly reduced infectious viral titers and were selected for further characterization and named PZ1–PZ6. These peptides displayed heterogeneous physicochemical profiles, with molecular weights ranging from 2561.91 to 3103.41 g/mol. At pH 7.0, PZ1, PZ5, and PZ6 were positively charged, PZ3 was negatively charged, and PZ2/PZ4 were near-neutral. GRAVY scores indicated hydrophilic profiles for PZ1, PZ2, PZ5, and PZ6, and comparatively more hydrophobic profiles for PZ3 and PZ4. All six peptides were predicted to be non-toxic and were soluble under the experimental conditions (Table 1).

Docking analysis predicted peptide-specific interaction patterns across the ZIKV E protein. PZ1 and PZ5 were predicted to map to regions involving domain II; PZ2, PZ4, and PZ6 mapped near domain I/domain II and fusion-loop-proximal interfaces; and PZ3 mapped predominantly to domain I/domain III-associated surfaces (Figure 1 and Table 2). Heavy-atom contact mapping revealed distinct interaction profiles with the E protein dimer; Figure 1 shows selected representative residues, and Supplementary Table S1 provides complete residue- and atom-level contacts.

### 3.2. Candidate Peptides Showed Concentration-Dependent Cytotoxicity and Hemolysis Profiles

MTT and hemolysis assays showed an acceptable safety profile for PZ1–PZ6. Most peptides maintained cell viability above the 70% threshold, with only PZ1 reducing viability at the highest concentration tested (Figure 2A). Hemolysis remained low at 50 and 100 µM, although PZ1, PZ2, PZ5, and PZ6 showed concentration-dependent increases at higher concentrations, whereas PZ3 and PZ4 displayed minimal hemolytic activity (Figure 2B). These results supported the use of 100 µM for subsequent antiviral assays.

### 3.3. Time-of-Addition Assays Revealed Treatment-Dependent Antiviral Activity

Pre-treatment elicited limited effects on infectious virus production, with only PZ6 showing a modest but significant reduction in viral titer. In contrast, co-treatment markedly reduced infectious titers for several peptides, particularly PZ2 and PZ6, while post-treatment reduced titers for all peptides, indicating that peptide activity was not restricted to the initial virus-cell contact step (Figure 3A). Cell-associated viral RNA levels showed a treatment-dependent pattern that did not fully mirror infectious virus production, with reductions observed for selected peptides in each treatment condition and significant increases for PZ1 and PZ4 during co-treatment (Figure 3B). Together, these data indicate that peptide effects depend on the timing of exposure and differ between infectious particle production and cell-associated viral RNA accumulation.

### 3.4. Peptides Interfered with Early ZIKV Entry-Associated Events

Anti-adsorption and DiOC18-based assays further supported peptide effects on early ZIKV entry-associated events. In the anti-adsorption assay, all candidate peptides significantly reduced infectious titers relative to the infection control, with PZ5 showing the strongest effect (Figure 4A). DiOC18 fluorescence analysis showed peptide-specific reductions in entry-associated membrane signals: PZ3 and PZ6 produced the strongest decreases, PZ2 and PZ5 also markedly reduced fluorescence, PZ1 showed a partial reduction, and PZ4 displayed a weaker and more variable effect (Figure 4B). NH_4_Cl strongly reduced the DiOC18-associated signal, consistent with inhibition of low-pH-dependent flavivirus entry [34,35]. Representative fluorescence and bright-field overlay micrographs supported the quantitative analysis, showing reduced green DiOC18-associated signal in several peptide-treated conditions compared with the infection control (Figure 4C).

Overall, peptide performance was assay-dependent: PZ5 showed the strongest anti-adsorption effect, PZ3 and PZ6 produced the most pronounced reductions in DiOC18-associated fluorescence, and PZ6 showed one of the most consistent activity profiles across functional assays.

### 3.5. Peptide-Treated ZIKV Particles Retained Nuclease-Protected RNA but Displayed Altered Ultrastructural Features

Nuclease protection assays showed that peptide-treated samples did not exhibit a generalized loss of nuclease-resistant viral RNA, unlike the Triton X-100 control, which nearly abolished the protected RNA signal. PZ1, PZ4, PZ5, and PZ6 showed viral RNA levels close to or above the Virus + MNase control, whereas PZ2 and PZ3 showed lower and more variable levels (Figure 5A). TEM analysis revealed particle-like structures in control samples and heterogeneous ultrastructural changes in peptide-treated conditions, including smaller particle-like structures, electron-dense aggregates, and altered morphologies. Together, these findings suggest that peptide treatment can alter the ultrastructural appearance of ZIKV-containing samples without causing generalized particle disruption or extensive exposure of the viral genome to nuclease digestion (Figure 5B).

## 4. Discussion

Zika virus remains a public health concern in tropical and subtropical regions where *Aedes* mosquitoes are established. Although the major epidemic wave in the Americas has declined, ZIKV persists in the region, maintaining the risk of sporadic outbreaks and congenital infections [4,36]. In the absence of a licensed vaccine or specific antiviral therapy, identifying antiviral candidates remains a research priority [10,15]. This need is particularly relevant because antiviral development against ZIKV faces several challenges, including limited clinically advanced candidates, the requirement for favorable safety profiles, and the difficulty of blocking early virus–host interactions with high specificity. Antiviral peptides offer a flexible platform for targeting extended protein surfaces and can be optimized by sequence modification, stabilization, or formulation strategies. However, many peptide candidates still require improved potency, mechanistic definition, and in vivo stability. In this context, protein–protein interaction-guided design provides a rational alternative to purely empirical screening or sequence-conservation-based peptide selection by focusing on structurally defined virus–host interfaces involved in viral attachment and entry. Here, we combined computational peptide design, docking, and in vitro antiviral assays to identify peptides with anti-ZIKV activity. Of the twenty peptides synthesized after in silico prioritization, only six significantly reduced infectious viral titers in preliminary screening, highlighting the need for experimental validation of computationally predicted antiviral candidates [37].

These results contribute to a growing body of work exploring peptide-based strategies against ZIKV and related flaviviruses. Previously reported anti-ZIKV or anti-flavivirus peptide candidates include E-protein stem-derived peptides such as P5 and Z2, human claudin-derived peptides, the anti-dengue stem-derived peptide DV2, and engineered membrane-active peptides such as AH-D [18,19,20,38,39]. Some of these candidates have reached dose–response and in vivo validation, including protection in mouse models of ZIKV infection. Unlike these approaches, the present study used a protein–protein interaction-guided strategy to derive peptide candidates from predicted ZIKV E protein–host receptor interfaces. Thus, the main contribution of this work is not to establish superior potency over previously reported peptides, but to provide proof of concept that structurally guided selection from virus–host interfaces can identify peptide candidates with activity against early ZIKV entry-associated events.

The physicochemical diversity of the six peptides suggests that antiviral activity is not driven solely by charge, hydrophobicity, or predicted solubility [21]. Although PZ1, PZ5, and PZ6 were positively charged, the activity of negatively charged PZ3 and near-neutral PZ2 and PZ4 indicates that global cationicity is not strictly required [22,40]. Instead, antiviral activity may depend more on local residue composition, peptide conformation, predicted binding orientation, and compatibility with functional E protein regions than on global physicochemical properties alone. Because the E protein mediates receptor attachment, low-pH-triggered rearrangements, and membrane fusion, docking-predicted peptide association with these regions could interfere with viral entry through non-mutually exclusive mechanisms if confirmed experimentally. In particular, predicted interactions with domain II or fusion-loop-proximal regions could restrict conformational changes required for fusion-loop exposure and membrane insertion [12,16,41]. However, these predictions should be interpreted as mechanistic hypotheses, since docking alone does not confirm binding under physiological conditions or define the exact inhibited step.

Further insights emerged from time-of-addition assays, which indicated that peptide activity depends on the timing of exposure relative to infection [42]. The limited effect observed during pre-treatment suggests that the peptides do not primarily act by stably modifying the cell surface or blocking cellular receptors before viral exposure. In contrast, the stronger activity during co-treatment supports a mechanism requiring simultaneous interaction among peptide, virus, and target cell, consistent with direct virion binding, steric interference with virus–cell attachment, or disruption of early E protein-mediated conformational events. Post-treatment activity further indicates that peptide effects are not restricted to initial attachment and may involve post-attachment events, including internalization, endosomal trafficking, low-pH-triggered E protein rearrangements, fusion, or the production/release of infectious particles.

Effects on virion maturation, including prM–E rearrangement or furin-mediated prM cleavage, cannot be excluded, but the present study did not directly assess this possibility [12,16,43]. The incomplete agreement between infectious titers and RT-qPCR-based viral RNA levels suggests that some peptides may reduce particle infectivity or productive infection without proportionally decreasing viral RNA entry or accumulation [44,45,46]. Thus, plaque assay and RT-qPCR should be interpreted as complementary rather than interchangeable antiviral readouts.

Several non-mutually exclusive explanations may account for this mismatch. First, plaque assay quantifies infectious virions, whereas RT-qPCR detects viral RNA and does not necessarily reflect plaque-forming capacity [44,45]. Second, cell-associated viral RNA may include RNA from virions attached to, retained at, or incompletely processed by cells during early entry without necessarily completing productive infection, as ZIKV attachment can be quantified by qPCR after binding at 4 °C and altered entry conditions can retain virions at the cell surface [35,47]. Third, peptide-induced structural or entry-associated alterations may reduce particle infectivity without causing complete genome exposure or degradation, as reported for claudin-derived peptides that inhibit ZIKV/flavivirus membrane fusion while preserving RNase-protected viral RNA [19]. Fourth, altered particle maturation or particle quality could reduce infectivity without proportionally decreasing viral RNA abundance, since ZIKV maturation involves prM/E rearrangements and furin-mediated prM cleavage that influence infectivity [43]. Finally, because flaviviral membrane fusion and nucleocapsid delivery can be separable events, detectable viral RNA or membrane-associated signals do not necessarily indicate completion of productive entry [48]. Therefore, the partial discordance between PFU and RT-qPCR does not necessarily contradict antiviral activity; rather, it suggests that some peptides may affect infectivity, particle quality, or productive entry efficiency more strongly than total viral RNA abundance.

Complementary to these findings, the anti-adsorption and DiOC18 assays further support peptide effects on early entry-associated events. In the anti-adsorption assay, all six peptides reduced infectious titers under conditions that favor viral binding while limiting internalization. Consistently, several peptides reduced DiOC18 fluorescence associated with labeled virion membrane association and/or early membrane-mixing events, with PZ3 and PZ6 showing the strongest effects. Because DiOC18-labeled virions were first allowed to bind at 4 °C and then shifted to 37 °C for only 30 min, this assay likely emphasizes attachment and very early entry-associated events rather than the full kinetics of productive internalization and endosomal fusion [49,50]. However, residual infectivity detected by plaque assay or RT-qPCR suggests that peptide treatment limits, but does not fully abolish, productive infection [48,50]. Accordingly, these data should not be interpreted as definitive evidence of fusion inhibition. Instead, the DiOC18 assay provides a functional readout of early virion-associated membrane events that may include attachment, membrane association, early internalization, or membrane mixing.

Consistent with this interpretation, results from the nuclease protection assay and TEM analysis suggest that the peptides do not act mainly as nonspecific membranolytic agents. None of the peptide-treated conditions produced a loss of nuclease-protected viral RNA comparable to the Triton X-100 control, indicating that peptide treatment did not cause generalized envelope disruption or extensive genome exposure. Nevertheless, TEM revealed heterogeneous ultrastructural changes in peptide-treated ZIKV-containing samples, including altered particle-like morphologies and electron-dense aggregates. These findings suggest that the peptides may induce subtle or partial structural perturbations that affect infectivity or entry efficiency without causing complete particle disassembly. However, the TEM observations should be interpreted as supportive qualitative evidence rather than quantitative proof of particle remodeling. Future morphometric analysis should determine whether these changes reflect altered particle size distribution, aggregation frequency, or the proportion of structurally altered particles.

Together, these findings suggest that the peptides reduce ZIKV infection through a multifactorial entry-associated mechanism rather than broad virion disruption. Their activity may involve altered virion–cell interactions, constrained E protein conformational changes, or reduced particle infectivity, and appears to depend on peptide properties, local residue interactions, and compatibility with functional E protein regions. As a proof of concept, this study supports the feasibility of a protein–protein interaction-guided strategy to identify anti-ZIKV peptide candidates with activity against early entry-associated events. Docking analyses suggest that these peptides may interact with functional regions of the ZIKV E protein, but direct binding and precise molecular mechanisms remain to be confirmed. This highlights their potential as starting points for further optimization toward therapeutic development.

Although these findings support the antiviral potential of PZ1–PZ6, several limitations should be clearly recognized. First, direct peptide–E protein binding was not experimentally confirmed; therefore, docking results should be interpreted as structural hypotheses rather than direct evidence of E protein targeting. Future studies should include biophysical or biochemical binding assays, such as SPR, MST, BLI, ELISA-based binding, peptide–virion pull-down, or competition assays using soluble E protein. Second, formal antiviral dose–response curves, IC50 values, CC50 values, and selectivity indices were not established for the selected peptides, limiting direct potency comparisons with previously reported antiviral candidates. Third, the anti-adsorption and time-of-addition assays did not include reference antiviral compounds for each step of the viral cycle, and no scrambled peptide controls were included; these controls should be incorporated in future mechanistic validation. Fourth, the DiOC18 assay supports effects on early entry-associated membrane events but does not distinguish attachment, internalization, endosomal trafficking, E protein conformational rearrangement, or membrane fusion. Fifth, TEM observations were qualitative and should be complemented by quantitative morphometry, including particle size distribution, aggregation frequency, and the proportion of altered particles.

From a translational perspective, the route of administration remains undefined at this stage. Because linear peptides are often susceptible to proteolytic degradation, future in vivo studies should evaluate serum stability, pharmacokinetics, biodistribution, toxicity, formulation requirements, and safety in additional human cell models and in vivo systems. Parenteral administration would likely be the most practical initial route for animal studies, whereas further optimization, such as sequence stabilization, cyclization, lipidation, nanoparticle formulation, or other delivery strategies, may be required before considering clinical applications. These considerations are particularly relevant if peptide-based interventions are eventually explored in the context of pregnancy-associated ZIKV infection, where safety and tissue exposure would be critical.

## Figures and Tables

**Figure 1:**
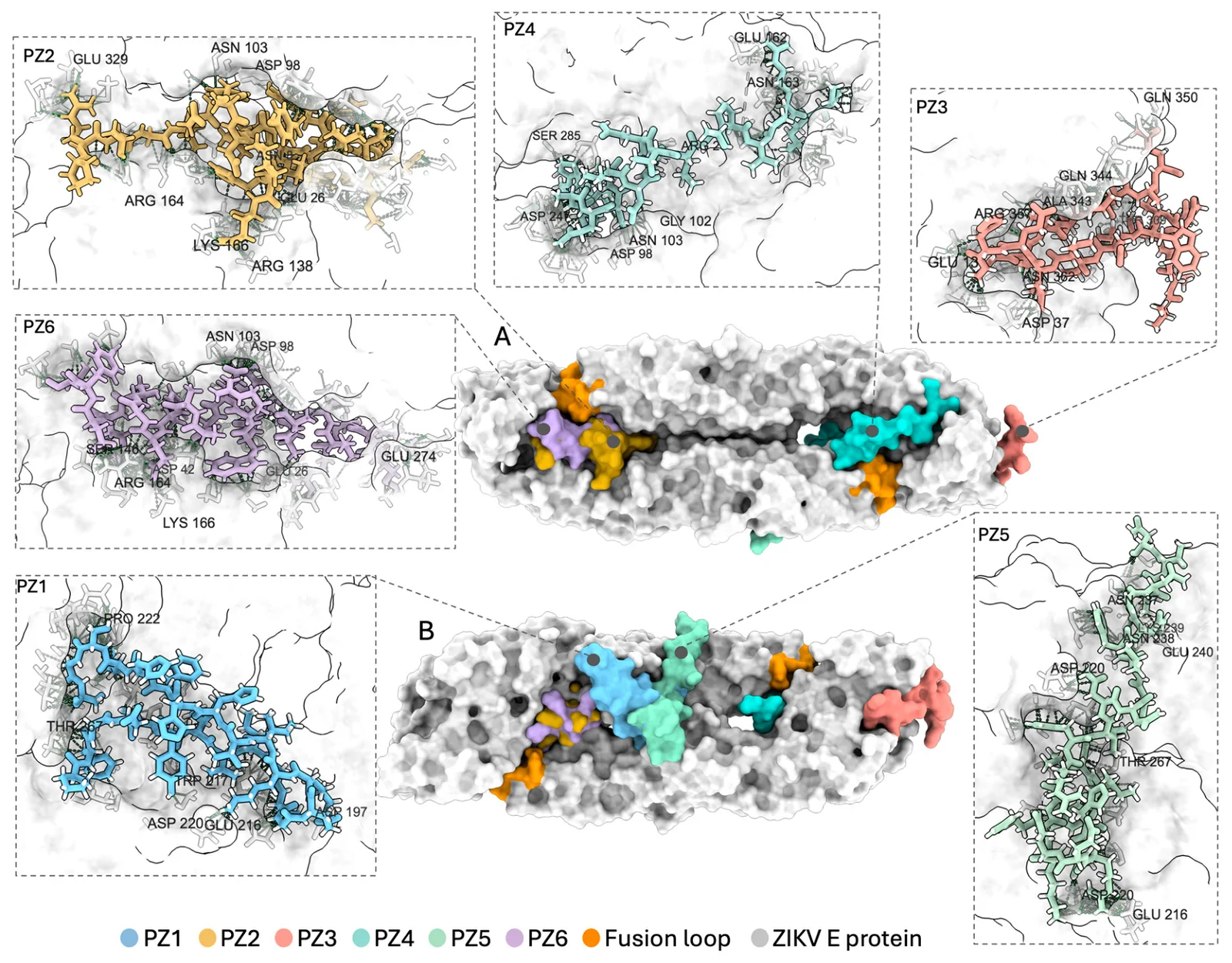
Predicted binding sites and intermolecular contacts between candidate peptides and the ZIKV E protein. (**A**) Surface representation of one orientation of the ZIKV E-protein dimer showing the predicted peptide-binding regions. (**B**) Opposite orientation of the same E-protein dimer. Enlarged panels show the predicted peptide protein interfaces for PZ1–PZ6. Light-green dashed lines indicate peptide–E-protein heavy-atom contacts within 4.0 Å, whereas dark-green dashed lines indicate predicted hydrogen bonds. Only selected representative E-protein residues are labeled for visual clarity. The complete residue-level and atom-to-atom contact datasets are provided in Supplementary Table S1. PZ1–PZ6 are displayed according to the indicated color code; the fusion loop is shown in orange and the E-protein surface in gray.

**Figure 2:**
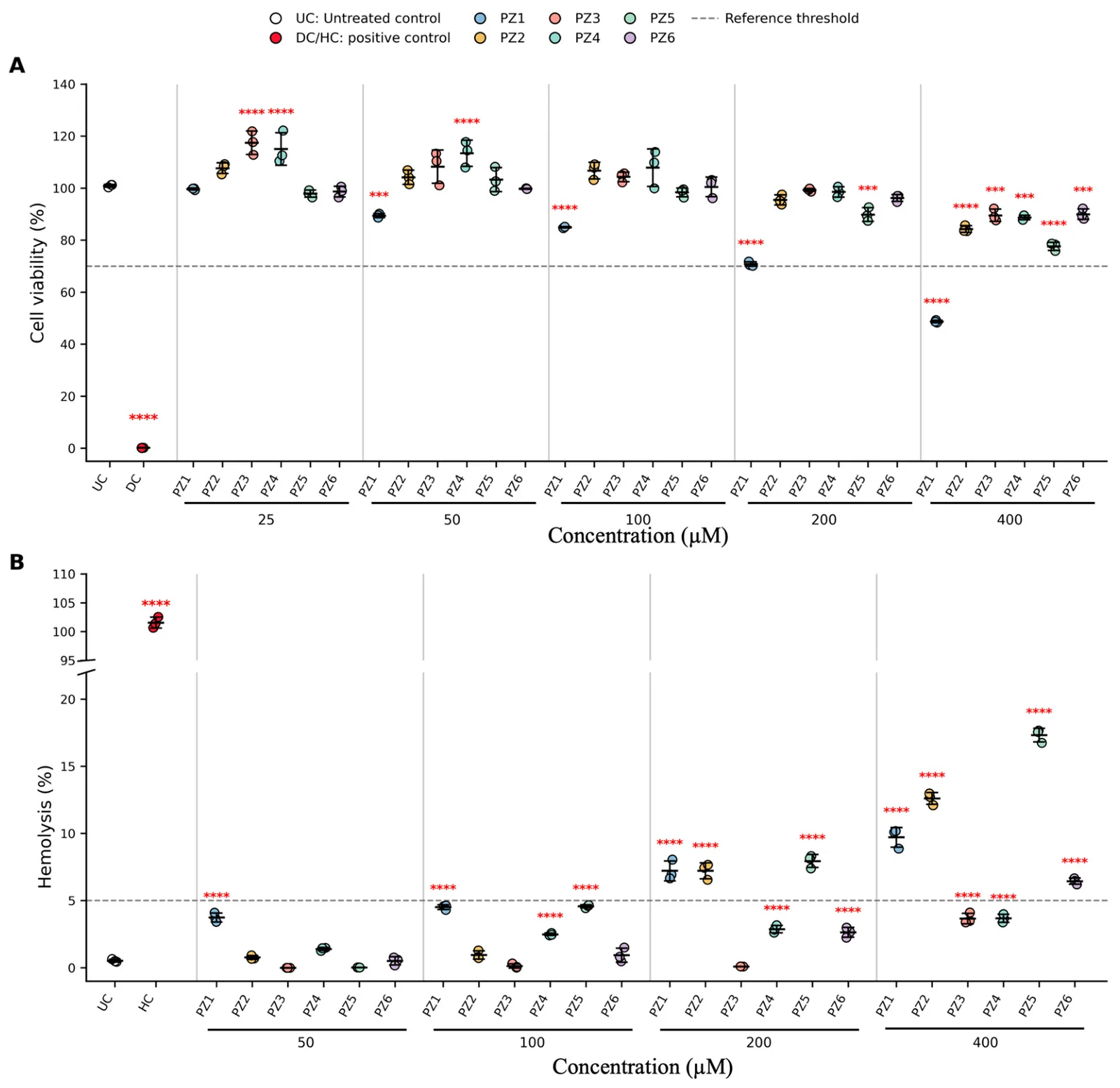
In vitro cytotoxicity and hemolytic activity of candidate anti-ZIKV peptides. (**A**) Cell viability of peptide-treated cells determined by MTT assay. PZ1–PZ6 were evaluated at the indicated concentrations, and viability was normalized to untreated control cells (UC). DC denotes the positive death control used in the viability assay. The dashed line indicates the predefined 70% cell viability threshold. (**B**) Hemolytic activity of candidate peptides evaluated at the indicated concentrations. Hemolysis was normalized to the Triton X-100 hemolysis control (HC). The dashed line indicates the predefined 5% hemolysis threshold. For both panels, each symbol represents the mean of one independent experiment performed in technical triplicate; horizontal lines and error bars indicate the mean ± SD of three independent experiments. Asterisks indicate statistically significant differences relative to UC in panel (**A**) or to the negative hemolysis control in panel (**B**); *** *p* < 0.001, **** *p* < 0.0001.

**Figure 3:**
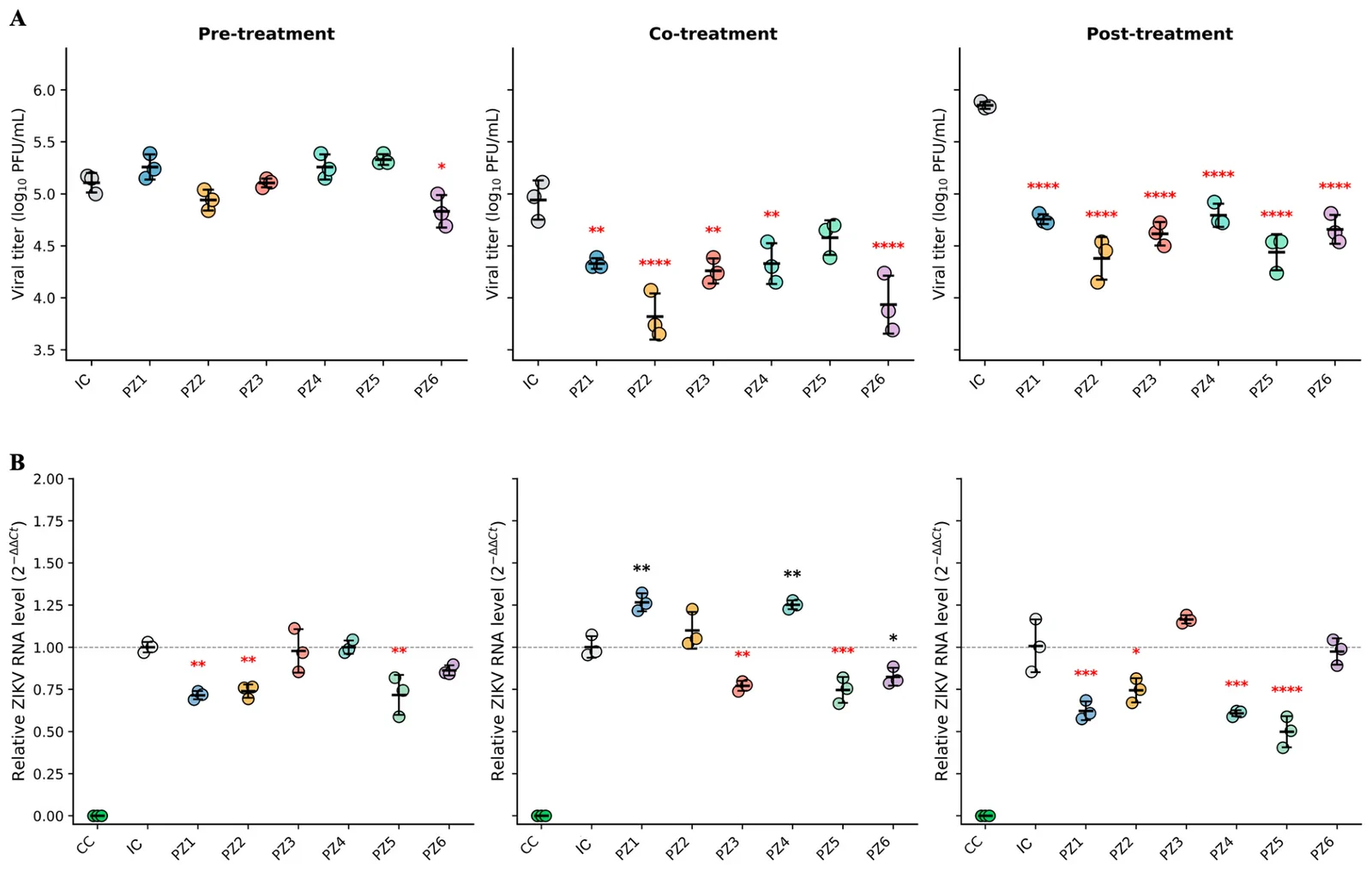
Time-of-addition antiviral activity of candidate peptides in HTR-8/SVneo trophoblast cells. HTR-8/SVneo cells were treated with candidate peptides at 100 µM before infection (pre-treatment), during infection (co-treatment), or after viral adsorption (post-treatment). (**A**) Infectious ZIKV titers were quantified by plaque assay and expressed as log_10_ PFU/mL. (**B**) Cell-associated ZIKV RNA levels were quantified by RT-qPCR and expressed as relative RNA levels using the 2^−ΔΔCt^ method, normalized to the infected cell control (IC); the dashed line indicates the IC baseline. GAPDH was used as the reference gene. Data represent three independent biological experiments; each symbol represents one independent experiment, and horizontal lines with error bars indicate mean ± SD. Asterisks indicate statistically significant differences relative to IC. In panel (**B**), red asterisks indicate significant reductions in viral RNA, whereas black asterisks indicate significant increases above the IC baseline. * *p* < 0.05, ** *p* < 0.01, *** *p* < 0.001, and **** *p* < 0.0001.

**Figure 4:**
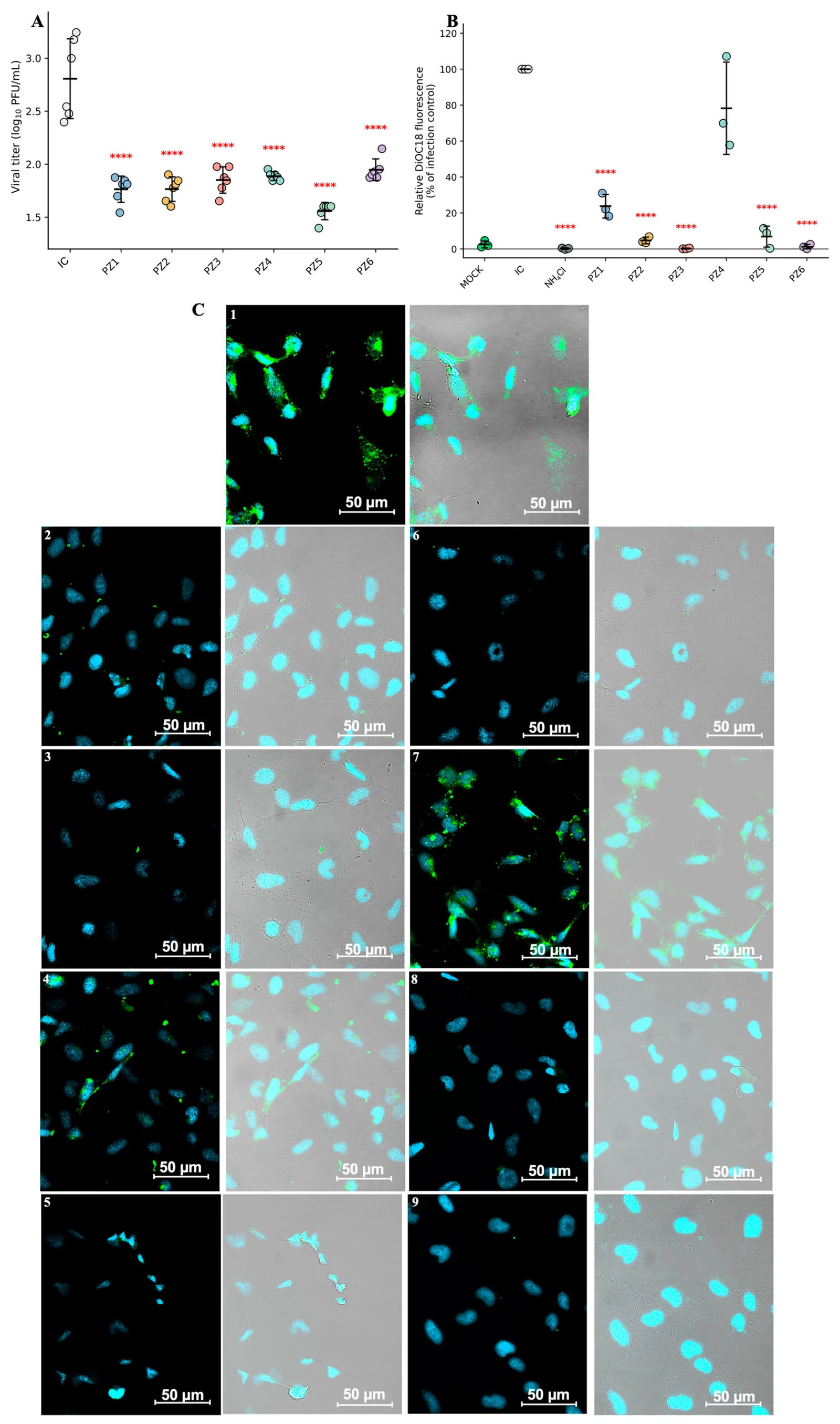
Candidate peptides interfere with early ZIKV entry-associated events. (**A**) Anti-adsorption activity of candidate peptides in Vero cells. Infectious viral titers were determined by plaque assay and expressed as log_10_ PFU/mL. (**B**) Quantification of DiOC18-associated fluorescence in HTR-8/SVneo cells using DiOC18-labeled ZIKV. Fluorescence values were normalized to the infection control (IC). (**C**) Representative fluorescence and bright-field overlay micrographs showing DiOC18-associated signal under the following conditions: 1: infection control, 2: mock-infected cells, 3: NH_4_Cl-treated control, 4: PZ1, 5: PZ2, 6: PZ3, 7: PZ4, 8: PZ5, and 9: PZ6. Green indicates DiOC18-associated viral signal, and cyan indicates nuclei. Scale bars: 50 µm. In panels (**A**,**B**), horizontal lines with error bars indicate mean ± SD. In panel (**A**), symbols represent technical replicates from three independent experiments; in panel (**B**), each symbol represents the mean fluorescence value from one independent experiment. This assay was used as a readout of early entry-associated membrane events and does not distinguish attachment, internalization, endosomal trafficking, or membrane fusion. Asterisks indicate statistically significant differences versus IC: **** *p* < 0.0001.

**Figure 5:**
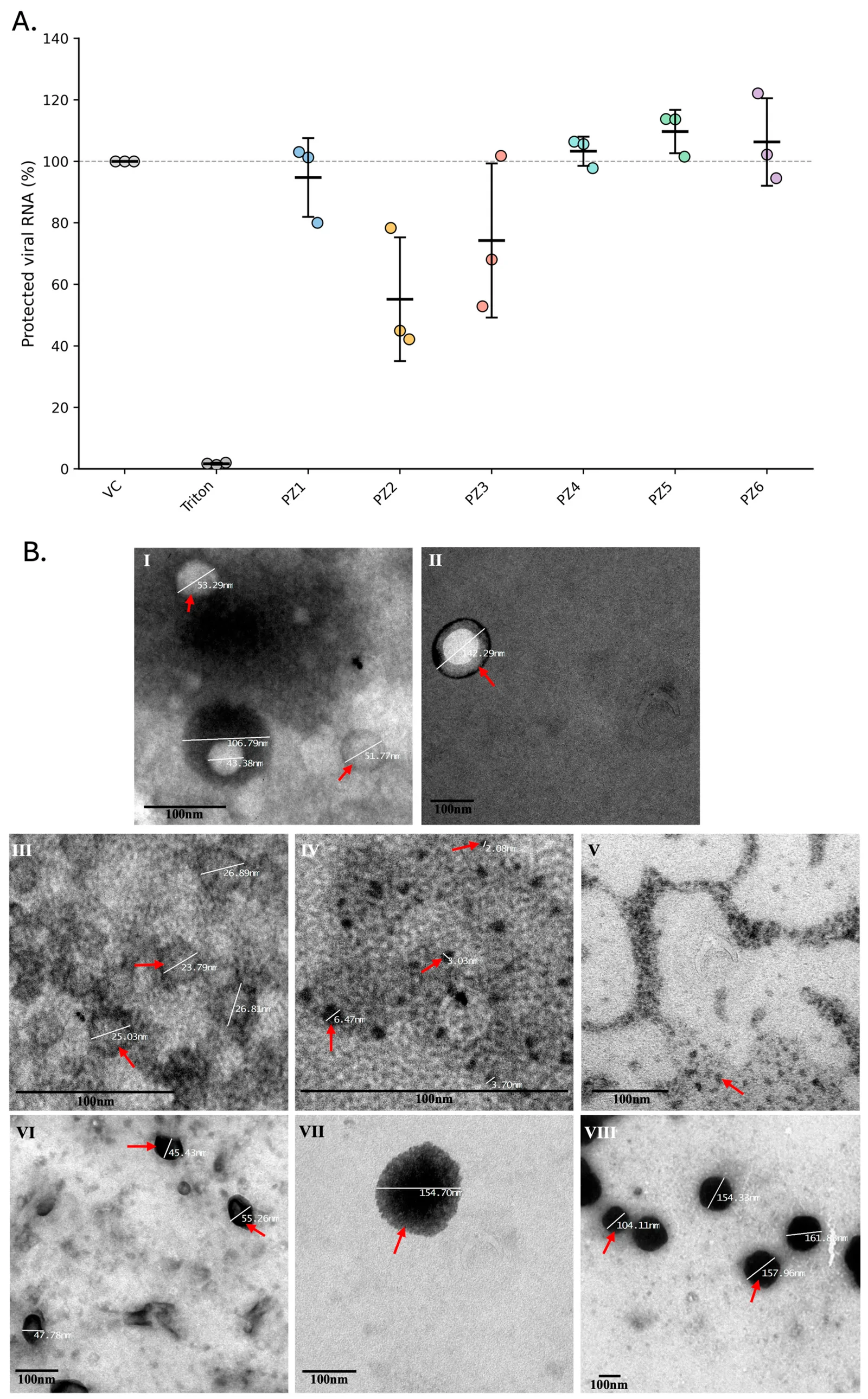
Peptide-treated ZIKV particles exhibit altered ultrastructural features while largely retaining nuclease-protected viral RNA. (**A**) Nuclease protection assay of peptide-treated ZIKV particles. Protected viral RNA was quantified by RT-qPCR and expressed relative to the Virus + MNase control (VC, dashed line). Triton X-100 + MNase was included as a positive disruption control. Symbols represent independent experiments, and horizontal lines with error bars indicate mean ± SD. (**B**) Representative TEM micrographs of ZIKV-containing samples: I, untreated ZIKV control; II, ZIKV exposed to HBS pH 5.6–5.8; and III–VIII, ZIKV treated with PZ1–PZ6, respectively. Red arrows indicate representative particle-like structures, electron-dense aggregates, or altered morphologies observed under each condition. White lines indicate representative diameter measurements. Scale bars: 100 nm. TEM images are shown as qualitative representative observations and were not used for morphometric quantification.

**Table 1:** Theoretical physicochemical properties and predicted toxicity of candidate peptides.

Peptide Name	Sequence	MW (g/mol)	pI	Net Charge at pH 7.0	GRAVY	Predicted Toxicity
PZ1	PFGDSYIVIGVGEKKITHHWHRSGS	2808.11	9.97	1.3	−0.508	Non-toxic
PZ2	PRTGLDFSDLYYLTMNNKHWLVHKE	3078.46	7.99	0.2	−0.792	Non-toxic
PZ3	VEGMSGGTWVDVVLEHGGCVTVMAQ	2561.91	3.69	−3	0.592	Non-toxic
PZ4	TDGPCKVPAQMAVDMQTLTPVGRLI	2642.13	6.05	−0.1	0.18	Non-toxic
PZ5	GLIYHREEKRWRWINNSVFNGNVTN	3103.41	10.44	2.1	−1.06	Non-toxic
PZ6	MKSRVIRAAWSWRESAAHEGTRDYT	2965.27	10.36	2.1	−1.03	Non-toxic

MW: molecular weight; pI: isoelectric point; GRAVY: grand average of hydropathicity.

**Table 2:** Predicted binding regions and selected interfacial residues of candidate peptides in docking complexes with the ZIKV E protein.

Peptide	Predicted E-Protein Region	Representative Interfacial Residues *	Potential Mechanistic Implication **
PZ1	Domain II	Asp197-A, Glu216-A, Trp217-A, Asp220-A, Pro222-B, Thr267-B	Predicted binding to DII, including residues Glu216 and Asp220, could perturb local E-protein stability or the conformational rearrangements required for membrane fusion.
PZ2	Domains I-III; fusion-loop-proximal region	Asn8-A, Glu26-A, Arg138-A, Arg164-A, Lys166-A, Glu329-A, Asp98-B, Asn103-B	Predicted binding across multiple domains and near the fusion loop could restrict fusion-loop exposure or insertion and interfere with the low-pH-induced rearrangements required for viral membrane fusion.
PZ3	Domains I and III	Glu13-B, Asp37-B, Thr309-B, Ala343-B, Gln344-B, Gln350-B, Arg357-B, Asn362-B	Predicted binding at DI/DIII surfaces could alter interdomain organization or accessibility of regions potentially involved in virion attachment; this mechanism remains less directly supported than fusion-loop interference.
PZ4	Domains I and II; fusion-loop-proximal region	Arg2-B, Asp98-A, Gly102-A, Asn103-A, Glu162-B, Asn163-B, Asp247-A, Ser285-B	Predicted contacts involving the fusion loop and adjacent DI/DII regions could sterically hinder fusion-loop exposure or membrane insertion during viral entry.
PZ5	Domain II	Glu216-B, Asp220-A, Asp220-B, Asn237-A, Asn238-A, Lys239-A, Glu240-A, Thr267-B	Predicted binding to DII could affect local dimer stability or restrict the conformational transition of E from the prefusion to the fusion-active state.
PZ6	Domains I and II; fusion-loop-proximal region	Glu26-A, Asp42-A, Asp98-B, Asn103-B, Ser146-A, Arg164-A, Lys166-A, Glu274-A	Predicted binding near the fusion loop and DI/DII interface could constrain hinge-associated movements and fusion-loop deployment required for endosomal membrane fusion.

* representative E-protein residues were selected from contacts detected within 4.0 Å based on contact frequency, chemical relevance, and visual clarity. ** potential mechanistic implications are inferred from predicted docking locations and require experimental validation.

## Data Availability

The data presented in this study are available within the article and Supplementary Materials. Additional raw data are available from the corresponding author upon reasonable request.

## Supplementary Materials

The following supporting information can be downloaded at: https://www.mdpi.com/article/10.3390/v18101066/s1. Table S1, complete residue-level and atom-to-atom contact datasets for peptide–ZIKV E protein docking complexes.

## Abbreviations

The following abbreviations are used in this manuscript:

ZIKV	Zika virus
E	Envelope protein
PZ	Peptide Zika
prM	Premembrane protein
DC-SIGN/CD209	Dendritic cell-specific intercellular adhesion molecule-3-grabbing non-integrin
MR/CD206	Mannose receptor
CLEC5A/MDL-1	C-type lectin domain family 5 member A/myeloid DAP12-associating lectin-1
PDB	Protein Data Bank
REU	Rosetta Energy Units
MOI	Multiplicity of infection
MTT	3-(4,5-dimethylthiazol-2-yl)-2,5-diphenyltetrazolium bromide
PFU	Plaque-forming units
RT-qPCR	Reverse transcription quantitative polymerase chain reaction
DiOC18	3,3′-dioctadecyloxacarbocyanine perchlorate
NH_4_Cl	Ammonium chloride
MNase	Micrococcal nuclease
TEM	Transmission electron microscopy
HBS	HEPES-buffered saline
UC	Untreated control
DC	Death control
HC	Hemolysis control
IC	Infected control
VC	Virus + MNase control
GRAVY	Grand average of hydropathicity
