# Supplementary material for: Protein–Protein Interaction-Guided Peptides Interfere with Early Zika Virus Entry-Associated Events
Source: Viruses. 2026 Sep 25;18(10):1066. doi: 10.3390/v18101066 (PMC13709921; doi:10.3390/v18101066)
Supplement: Supplementary file 1 [file viruses-18-01066-s001.zip › viruses-4556908-supplementary.pdf]

Table S1: complete residue-level and atom-to-atom contact datasets for peptide–ZIKV E protein docking complexes.

Peptide	peptide_sequence	E_chain	E_residue	E_atom	Peptide residue	Peptide atom	distance_A	E_region	interaction_candidate
**PZ1**	PFGDSYIVIGVGEKKITHHWHRSGS	A	PRO222	CG	PRO1	N	3.565	DII	hydrophobic/van der Waals
**PZ1**	PFGDSYIVIGVGEKKITHHWHRSGS	A	PRO222	CG	PRO1	CA	3.687	DII	hydrophobic/van der Waals
**PZ1**	PFGDSYIVIGVGEKKITHHWHRSGS	B	THR267	CG2	PRO1	C	3.969	DII	H-bond/polar candidate
**PZ1**	PFGDSYIVIGVGEKKITHHWHRSGS	B	THR267	CB	PRO1	O	3.123	DII	H-bond/polar candidate
**PZ1**	PFGDSYIVIGVGEKKITHHWHRSGS	B	THR267	OG1	PRO1	O	3.928	DII	H-bond/polar candidate
**PZ1**	PFGDSYIVIGVGEKKITHHWHRSGS	B	THR267	HG1	PRO1	O	3.542	DII	H-bond/polar candidate
**PZ1**	PFGDSYIVIGVGEKKITHHWHRSGS	B	THR267	CG2	PRO1	O	3.195	DII	H-bond/polar candidate
**PZ1**	PFGDSYIVIGVGEKKITHHWHRSGS	A	PRO222	CG	PHE2	N	3.775	DII	hydrophobic/van der Waals
**PZ1**	PFGDSYIVIGVGEKKITHHWHRSGS	A	PRO222	CD	PHE2	H	3.827	DII	hydrophobic/van der Waals
**PZ1**	PFGDSYIVIGVGEKKITHHWHRSGS	A	PRO222	CG	PHE2	H	2.955	DII	hydrophobic/van der Waals
**PZ1**	PFGDSYIVIGVGEKKITHHWHRSGS	B	THR267	HG1	PHE2	CA	3.444	DII	H-bond/polar candidate
**PZ1**	PFGDSYIVIGVGEKKITHHWHRSGS	B	THR267	OG1	PHE2	CB	3.839	DII	H-bond/polar candidate
**PZ1**	PFGDSYIVIGVGEKKITHHWHRSGS	B	THR267	HG1	PHE2	CB	3.062	DII	H-bond/polar candidate
**PZ1**	PFGDSYIVIGVGEKKITHHWHRSGS	B	THR267	CG2	PHE2	CB	3.862	DII	H-bond/polar candidate
**PZ1**	PFGDSYIVIGVGEKKITHHWHRSGS	A	ALA264	CB	PHE2	CD1	3.762	DII	hydrophobic/van der Waals
**PZ1**	PFGDSYIVIGVGEKKITHHWHRSGS	A	PRO222	CG	PHE2	CD2	3.842	DII	hydrophobic/van der Waals
**PZ1**	PFGDSYIVIGVGEKKITHHWHRSGS	A	LEU223	CG	PHE2	CE2	3.56	DII	hydrophobic/van der Waals
**PZ1**	PFGDSYIVIGVGEKKITHHWHRSGS	A	LEU223	CD1	PHE2	CE2	3.764	DII	hydrophobic/van der Waals
**PZ1**	PFGDSYIVIGVGEKKITHHWHRSGS	A	LEU223	CD2	PHE2	CE2	3.937	DII	hydrophobic/van der Waals
**PZ1**	PFGDSYIVIGVGEKKITHHWHRSGS	A	ILE221	CG2	PHE2	CZ	3.7	DII	hydrophobic/van der Waals
**PZ1**	PFGDSYIVIGVGEKKITHHWHRSGS	A	VAL265	CG2	PHE2	CZ	3.879	DII	hydrophobic/van der Waals
**PZ1**	PFGDSYIVIGVGEKKITHHWHRSGS	B	THR267	CB	PHE2	C	3.905	DII	H-bond/polar candidate
**PZ1**	PFGDSYIVIGVGEKKITHHWHRSGS	B	THR267	OG1	PHE2	C	3.639	DII	H-bond/polar candidate
**PZ1**	PFGDSYIVIGVGEKKITHHWHRSGS	B	THR267	HG1	PHE2	C	2.759	DII	H-bond/polar candidate
**PZ1**	PFGDSYIVIGVGEKKITHHWHRSGS	B	THR267	CB	PHE2	O	3.427	DII	H-bond/polar candidate
**PZ1**	PFGDSYIVIGVGEKKITHHWHRSGS	B	THR267	OG1	PHE2	O	2.774	DII	H-bond/polar candidate
**PZ1**	PFGDSYIVIGVGEKKITHHWHRSGS	B	THR267	HG1	PHE2	O	1.872	DII	H-bond/polar candidate

**PZ1**	PFGDSYIVIGVGEKKITHHWHRSGS	B	THR267	HG1	GLY3	N	3.799	DII	H-bond/polar candidate
**PZ1**	PFGDSYIVIGVGEKKITHHWHRSGS	A	THR267	CG2	SER5	CB	3.825	DII	H-bond/polar candidate
**PZ1**	PFGDSYIVIGVGEKKITHHWHRSGS	A	THR267	C	SER5	CB	3.971	DII	H-bond/polar candidate
**PZ1**	PFGDSYIVIGVGEKKITHHWHRSGS	A	ALA268	N	SER5	CB	3.708	DII	H-bond/polar candidate
**PZ1**	PFGDSYIVIGVGEKKITHHWHRSGS	A	ALA268	H	SER5	CB	3.91	DII	H-bond/polar candidate
**PZ1**	PFGDSYIVIGVGEKKITHHWHRSGS	A	ALA268	CA	SER5	CB	3.841	DII	H-bond/polar candidate
**PZ1**	PFGDSYIVIGVGEKKITHHWHRSGS	A	THR267	CG2	SER5	OG	3.952	DII	H-bond/polar candidate
**PZ1**	PFGDSYIVIGVGEKKITHHWHRSGS	A	ALA268	CA	SER5	C	3.802	DII	H-bond/polar candidate
**PZ1**	PFGDSYIVIGVGEKKITHHWHRSGS	A	THR267	O	SER5	O	3.883	DII	H-bond/polar candidate
**PZ1**	PFGDSYIVIGVGEKKITHHWHRSGS	A	ALA268	N	SER5	O	3.998	DII	H-bond/polar candidate
**PZ1**	PFGDSYIVIGVGEKKITHHWHRSGS	A	ALA268	CA	SER5	O	3.239	DII	H-bond/polar candidate
**PZ1**	PFGDSYIVIGVGEKKITHHWHRSGS	A	ALA268	CB	SER5	O	3.92	DII	H-bond/polar candidate
**PZ1**	PFGDSYIVIGVGEKKITHHWHRSGS	A	ALA268	C	SER5	O	3.989	DII	H-bond/polar candidate
**PZ1**	PFGDSYIVIGVGEKKITHHWHRSGS	A	ALA268	O	SER5	O	3.814	DII	H-bond/polar candidate
**PZ1**	PFGDSYIVIGVGEKKITHHWHRSGS	A	TRP217	HE1	TYR6	N	3.927	DII	H-bond/polar candidate; hydrophobic/van der Waals
**PZ1**	PFGDSYIVIGVGEKKITHHWHRSGS	A	TRP217	HE1	TYR6	CD1	3.889	DII	H-bond/polar candidate; hydrophobic/van der Waals
**PZ1**	PFGDSYIVIGVGEKKITHHWHRSGS	A	TRP217	CD1	TYR6	CE1	3.753	DII	H-bond/polar candidate; hydrophobic/van der Waals
**PZ1**	PFGDSYIVIGVGEKKITHHWHRSGS	A	TRP217	CD1	TYR6	CD2	3.998	DII	H-bond/polar candidate; hydrophobic/van der Waals
**PZ1**	PFGDSYIVIGVGEKKITHHWHRSGS	A	TRP217	CD1	TYR6	CE2	3.551	DII	H-bond/polar candidate; hydrophobic/van der Waals
**PZ1**	PFGDSYIVIGVGEKKITHHWHRSGS	A	TRP217	CD1	TYR6	CZ	3.394	DII	H-bond/polar candidate; hydrophobic/van der Waals
**PZ1**	PFGDSYIVIGVGEKKITHHWHRSGS	A	TRP217	CA	TYR6	OH	3.437	DII	H-bond/polar candidate; hydrophobic/van der Waals
**PZ1**	PFGDSYIVIGVGEKKITHHWHRSGS	A	TRP217	CB	TYR6	OH	3.88	DII	H-bond/polar candidate; hydrophobic/van der Waals
**PZ1**	PFGDSYIVIGVGEKKITHHWHRSGS	A	TRP217	CD1	TYR6	OH	3.691	DII	H-bond/polar candidate; hydrophobic/van der Waals
**PZ1**	PFGDSYIVIGVGEKKITHHWHRSGS	A	TRP217	CA	TYR6	HH	3.816	DII	H-bond/polar candidate; hydrophobic/van der Waals
**PZ1**	PFGDSYIVIGVGEKKITHHWHRSGS	A	ASP220	CB	TYR6	HH	3.553	DII	H-bond/polar candidate
**PZ1**	PFGDSYIVIGVGEKKITHHWHRSGS	A	TRP217	HE1	TYR6	C	3.289	DII	H-bond/polar candidate; hydrophobic/van der Waals
**PZ1**	PFGDSYIVIGVGEKKITHHWHRSGS	A	TRP217	CD1	TYR6	O	3.185	DII	H-bond/polar candidate; hydrophobic/van der Waals
**PZ1**	PFGDSYIVIGVGEKKITHHWHRSGS	A	TRP217	NE1	TYR6	O	2.809	DII	H-bond/polar candidate; hydrophobic/van der Waals
**PZ1**	PFGDSYIVIGVGEKKITHHWHRSGS	A	TRP217	HE1	TYR6	O	2.127	DII	H-bond/polar candidate; hydrophobic/van der Waals
**PZ1**	PFGDSYIVIGVGEKKITHHWHRSGS	A	ALA268	O	TYR6	O	3.837	DII	H-bond/polar candidate; hydrophobic/van der Waals

**PZ1**	PFGDSYIVIGVGEKKITHHWHRSGS	A	ALA268	O	ILE7	CA	3.727	DII	hydrophobic/van der Waals
**PZ1**	PFGDSYIVIGVGEKKITHHWHRSGS	A	ALA268	O	ILE7	CB	3.806	DII	hydrophobic/van der Waals
**PZ1**	PFGDSYIVIGVGEKKITHHWHRSGS	A	GLY271	H	ILE7	CB	3.615	DII	hydrophobic/van der Waals
**PZ1**	PFGDSYIVIGVGEKKITHHWHRSGS	A	ALA268	O	ILE7	CG2	3.062	DII	hydrophobic/van der Waals
**PZ1**	PFGDSYIVIGVGEKKITHHWHRSGS	A	ALA270	H	ILE7	CG2	3.881	DII	hydrophobic/van der Waals
**PZ1**	PFGDSYIVIGVGEKKITHHWHRSGS	A	GLY271	N	ILE7	CG2	3.729	DII	hydrophobic/van der Waals
**PZ1**	PFGDSYIVIGVGEKKITHHWHRSGS	A	GLY271	H	ILE7	CG2	2.82	DII	hydrophobic/van der Waals
**PZ1**	PFGDSYIVIGVGEKKITHHWHRSGS	A	HIS214	HD1	VAL8	H	3.831	DII	H-bond/polar candidate
**PZ1**	PFGDSYIVIGVGEKKITHHWHRSGS	A	HIS214	HD1	VAL8	CA	3.513	DII	H-bond/polar candidate
**PZ1**	PFGDSYIVIGVGEKKITHHWHRSGS	A	HIS214	ND1	VAL8	CB	3.727	DII	H-bond/polar candidate
**PZ1**	PFGDSYIVIGVGEKKITHHWHRSGS	A	HIS214	HD1	VAL8	CB	3.155	DII	H-bond/polar candidate
**PZ1**	PFGDSYIVIGVGEKKITHHWHRSGS	A	HIS214	CB	VAL8	CG1	3.873	DII	H-bond/polar candidate
**PZ1**	PFGDSYIVIGVGEKKITHHWHRSGS	A	HIS214	CG	VAL8	CG1	3.775	DII	H-bond/polar candidate
**PZ1**	PFGDSYIVIGVGEKKITHHWHRSGS	A	HIS214	ND1	VAL8	CG1	3.667	DII	H-bond/polar candidate
**PZ1**	PFGDSYIVIGVGEKKITHHWHRSGS	A	HIS214	HD1	VAL8	CG1	3.486	DII	H-bond/polar candidate
**PZ1**	PFGDSYIVIGVGEKKITHHWHRSGS	A	HIS214	ND1	VAL8	C	3.368	DII	H-bond/polar candidate
**PZ1**	PFGDSYIVIGVGEKKITHHWHRSGS	A	HIS214	HD1	VAL8	C	2.833	DII	H-bond/polar candidate
**PZ1**	PFGDSYIVIGVGEKKITHHWHRSGS	A	HIS214	CE1	VAL8	C	3.669	DII	H-bond/polar candidate
**PZ1**	PFGDSYIVIGVGEKKITHHWHRSGS	A	HIS214	CG	VAL8	O	3.996	DII	H-bond/polar candidate
**PZ1**	PFGDSYIVIGVGEKKITHHWHRSGS	A	HIS214	ND1	VAL8	O	2.752	DII	H-bond/polar candidate
**PZ1**	PFGDSYIVIGVGEKKITHHWHRSGS	A	HIS214	HD1	VAL8	O	2.09	DII	H-bond/polar candidate
**PZ1**	PFGDSYIVIGVGEKKITHHWHRSGS	A	HIS214	CE1	VAL8	O	3.056	DII	H-bond/polar candidate
**PZ1**	PFGDSYIVIGVGEKKITHHWHRSGS	A	HIS214	HD1	ILE9	N	3.749	DII	H-bond/polar candidate
**PZ1**	PFGDSYIVIGVGEKKITHHWHRSGS	A	HIS214	CE1	ILE9	N	3.93	DII	H-bond/polar candidate
**PZ1**	PFGDSYIVIGVGEKKITHHWHRSGS	A	HIS214	CE1	ILE9	CA	3.451	DII	H-bond/polar candidate
**PZ1**	PFGDSYIVIGVGEKKITHHWHRSGS	A	HIS214	NE2	ILE9	C	3.901	DII	H-bond/polar candidate
**PZ1**	PFGDSYIVIGVGEKKITHHWHRSGS	A	HIS214	CE1	ILE9	C	3.598	DII	H-bond/polar candidate
**PZ1**	PFGDSYIVIGVGEKKITHHWHRSGS	A	HIS214	NE2	GLY10	N	3.14	DII	H-bond/polar candidate
**PZ1**	PFGDSYIVIGVGEKKITHHWHRSGS	A	HIS214	CE1	GLY10	N	3.219	DII	H-bond/polar candidate
**PZ1**	PFGDSYIVIGVGEKKITHHWHRSGS	A	HIS214	ND1	GLY10	H	3.812	DII	H-bond/polar candidate

**PZ1**	PFGDSYIVIGVGEKKITHHWHRSGS	A	HIS214	CD2	GLY10	H	3.785	DII	H-bond/polar candidate
**PZ1**	PFGDSYIVIGVGEKKITHHWHRSGS	A	HIS214	NE2	GLY10	H	2.609	DII	H-bond/polar candidate
**PZ1**	PFGDSYIVIGVGEKKITHHWHRSGS	A	HIS214	CE1	GLY10	H	2.629	DII	H-bond/polar candidate
**PZ1**	PFGDSYIVIGVGEKKITHHWHRSGS	A	HIS214	NE2	GLY10	CA	3.613	DII	H-bond/polar candidate
**PZ1**	PFGDSYIVIGVGEKKITHHWHRSGS	A	HIS214	CD2	GLY10	C	3.486	DII	H-bond/polar candidate
**PZ1**	PFGDSYIVIGVGEKKITHHWHRSGS	A	HIS214	NE2	GLY10	C	3.262	DII	H-bond/polar candidate
**PZ1**	PFGDSYIVIGVGEKKITHHWHRSGS	A	HIS214	CG	GLY10	O	3.964	DII	H-bond/polar candidate
**PZ1**	PFGDSYIVIGVGEKKITHHWHRSGS	A	HIS214	CD2	GLY10	O	3.403	DII	H-bond/polar candidate
**PZ1**	PFGDSYIVIGVGEKKITHHWHRSGS	A	HIS214	NE2	GLY10	O	3.507	DII	H-bond/polar candidate
**PZ1**	PFGDSYIVIGVGEKKITHHWHRSGS	A	HIS214	CD2	VAL11	N	3.552	DII	H-bond/polar candidate
**PZ1**	PFGDSYIVIGVGEKKITHHWHRSGS	A	HIS214	NE2	VAL11	N	3.454	DII	H-bond/polar candidate
**PZ1**	PFGDSYIVIGVGEKKITHHWHRSGS	A	HIS214	NE2	VAL11	H	3.835	DII	H-bond/polar candidate
**PZ1**	PFGDSYIVIGVGEKKITHHWHRSGS	A	ASP200	OD1	VAL11	CA	3.702	DII	H-bond/polar candidate
**PZ1**	PFGDSYIVIGVGEKKITHHWHRSGS	A	HIS214	CD2	VAL11	CA	3.474	DII	H-bond/polar candidate
**PZ1**	PFGDSYIVIGVGEKKITHHWHRSGS	A	HIS214	NE2	VAL11	CA	3.898	DII	H-bond/polar candidate
**PZ1**	PFGDSYIVIGVGEKKITHHWHRSGS	A	ASP200	CB	VAL11	CB	3.453	DII	H-bond/polar candidate
**PZ1**	PFGDSYIVIGVGEKKITHHWHRSGS	A	ASP200	OD1	VAL11	CB	3.926	DII	H-bond/polar candidate
**PZ1**	PFGDSYIVIGVGEKKITHHWHRSGS	A	HIS214	CD2	VAL11	CB	3.995	DII	H-bond/polar candidate
**PZ1**	PFGDSYIVIGVGEKKITHHWHRSGS	A	ASP200	CB	VAL11	CG1	3.996	DII	H-bond/polar candidate
**PZ1**	PFGDSYIVIGVGEKKITHHWHRSGS	A	ASP200	C	VAL11	CG1	3.963	DII	H-bond/polar candidate
**PZ1**	PFGDSYIVIGVGEKKITHHWHRSGS	A	ASP200	O	VAL11	CG1	3.564	DII	H-bond/polar candidate
**PZ1**	PFGDSYIVIGVGEKKITHHWHRSGS	A	LEU201	CG	VAL11	CG1	3.704	DII	hydrophobic/van der Waals
**PZ1**	PFGDSYIVIGVGEKKITHHWHRSGS	A	HIS214	CD2	VAL11	CG1	3.48	DII	H-bond/polar candidate
**PZ1**	PFGDSYIVIGVGEKKITHHWHRSGS	A	HIS214	NE2	VAL11	CG1	3.652	DII	H-bond/polar candidate
**PZ1**	PFGDSYIVIGVGEKKITHHWHRSGS	A	ASP197	OD2	VAL11	CG2	3.411	DII	H-bond/polar candidate
**PZ1**	PFGDSYIVIGVGEKKITHHWHRSGS	A	ASP200	OD1	VAL11	C	3.817	DII	H-bond/polar candidate
**PZ1**	PFGDSYIVIGVGEKKITHHWHRSGS	A	ASP200	CB	GLY12	N	3.9	DII	H-bond/polar candidate
**PZ1**	PFGDSYIVIGVGEKKITHHWHRSGS	A	ASP200	CG	GLY12	N	3.73	DII	H-bond/polar candidate
**PZ1**	PFGDSYIVIGVGEKKITHHWHRSGS	A	ASP200	OD1	GLY12	N	2.95	DII	H-bond/polar candidate
**PZ1**	PFGDSYIVIGVGEKKITHHWHRSGS	A	ASP200	CB	GLY12	H	3.216	DII	H-bond/polar candidate

**PZ1**	PFGDSYIVIGVGEKKITHHWHRSGS	A	ASP200	CG	GLY12	H	2.822	DII	H-bond/polar candidate
**PZ1**	PFGDSYIVIGVGEKKITHHWHRSGS	A	ASP200	OD1	GLY12	H	1.955	DII	H-bond/polar candidate
**PZ1**	PFGDSYIVIGVGEKKITHHWHRSGS	A	ASP200	OD2	GLY12	H	3.898	DII	H-bond/polar candidate
**PZ1**	PFGDSYIVIGVGEKKITHHWHRSGS	A	ASP200	OD1	GLY12	CA	3.816	DII	H-bond/polar candidate
**PZ1**	PFGDSYIVIGVGEKKITHHWHRSGS	A	ASP197	OD2	LYS14	CE	3.464	DII	salt bridge candidate; H-bond/polar candidate
**PZ1**	PFGDSYIVIGVGEKKITHHWHRSGS	A	ASP197	OD2	LYS14	NZ	2.85	DII	salt bridge candidate; H-bond/polar candidate
**PZ1**	PFGDSYIVIGVGEKKITHHWHRSGS	A	ASP197	OD2	LYS14	HZ1	3.501	DII	salt bridge candidate; H-bond/polar candidate
**PZ1**	PFGDSYIVIGVGEKKITHHWHRSGS	A	ASP197	OD2	LYS14	HZ2	3.376	DII	salt bridge candidate; H-bond/polar candidate
**PZ1**	PFGDSYIVIGVGEKKITHHWHRSGS	A	ASP197	CG	LYS14	HZ3	3.076	DII	salt bridge candidate; H-bond/polar candidate
**PZ1**	PFGDSYIVIGVGEKKITHHWHRSGS	A	ASP197	OD1	LYS14	HZ3	3.885	DII	salt bridge candidate; H-bond/polar candidate
**PZ1**	PFGDSYIVIGVGEKKITHHWHRSGS	A	ASP197	OD2	LYS14	HZ3	1.836	DII	salt bridge candidate; H-bond/polar candidate
**PZ1**	PFGDSYIVIGVGEKKITHHWHRSGS	A	GLU216	OE1	LYS15	CE	3.747	DII	salt bridge candidate; H-bond/polar candidate
**PZ1**	PFGDSYIVIGVGEKKITHHWHRSGS	A	GLU216	OE2	LYS15	CE	3.795	DII	salt bridge candidate; H-bond/polar candidate
**PZ1**	PFGDSYIVIGVGEKKITHHWHRSGS	A	GLU216	CD	LYS15	NZ	2.821	DII	salt bridge candidate; H-bond/polar candidate
**PZ1**	PFGDSYIVIGVGEKKITHHWHRSGS	A	GLU216	OE1	LYS15	NZ	2.549	DII	salt bridge candidate; H-bond/polar candidate
**PZ1**	PFGDSYIVIGVGEKKITHHWHRSGS	A	GLU216	OE2	LYS15	NZ	2.556	DII	salt bridge candidate; H-bond/polar candidate
**PZ1**	PFGDSYIVIGVGEKKITHHWHRSGS	A	GLU216	CB	LYS15	HZ1	3.843	DII	salt bridge candidate; H-bond/polar candidate
**PZ1**	PFGDSYIVIGVGEKKITHHWHRSGS	A	GLU216	CG	LYS15	HZ1	3.442	DII	salt bridge candidate; H-bond/polar candidate
**PZ1**	PFGDSYIVIGVGEKKITHHWHRSGS	A	GLU216	CD	LYS15	HZ1	2.209	DII	salt bridge candidate; H-bond/polar candidate
**PZ1**	PFGDSYIVIGVGEKKITHHWHRSGS	A	GLU216	OE1	LYS15	HZ1	2.414	DII	salt bridge candidate; H-bond/polar candidate
**PZ1**	PFGDSYIVIGVGEKKITHHWHRSGS	A	GLU216	OE2	LYS15	HZ1	1.718	DII	salt bridge candidate; H-bond/polar candidate
**PZ1**	PFGDSYIVIGVGEKKITHHWHRSGS	A	GLU216	CB	LYS15	HZ2	3.888	DII	salt bridge candidate; H-bond/polar candidate
**PZ1**	PFGDSYIVIGVGEKKITHHWHRSGS	A	GLU216	CG	LYS15	HZ2	3.743	DII	salt bridge candidate; H-bond/polar candidate
**PZ1**	PFGDSYIVIGVGEKKITHHWHRSGS	A	GLU216	CD	LYS15	HZ2	2.433	DII	salt bridge candidate; H-bond/polar candidate
**PZ1**	PFGDSYIVIGVGEKKITHHWHRSGS	A	GLU216	OE1	LYS15	HZ2	1.765	DII	salt bridge candidate; H-bond/polar candidate
**PZ1**	PFGDSYIVIGVGEKKITHHWHRSGS	A	GLU216	OE2	LYS15	HZ2	2.629	DII	salt bridge candidate; H-bond/polar candidate
**PZ1**	PFGDSYIVIGVGEKKITHHWHRSGS	A	GLU216	CD	LYS15	HZ3	3.271	DII	salt bridge candidate; H-bond/polar candidate
**PZ1**	PFGDSYIVIGVGEKKITHHWHRSGS	A	GLU216	OE1	LYS15	HZ3	2.989	DII	salt bridge candidate; H-bond/polar candidate
**PZ1**	PFGDSYIVIGVGEKKITHHWHRSGS	A	GLU216	OE2	LYS15	HZ3	2.79	DII	salt bridge candidate; H-bond/polar candidate
**PZ1**	PFGDSYIVIGVGEKKITHHWHRSGS	A	ALA270	CB	TRP20	CE3	3.992	DII	hydrophobic/van der Waals

**PZ1**	PFGDSYIVIGVGEKKITHHWHRSGS	A	THR267	CG2	TRP20	CD1	3.788	DII	H-bond/polar candidate
**PZ1**	PFGDSYIVIGVGEKKITHHWHRSGS	A	ALA270	CB	TRP20	CZ3	3.91	DII	hydrophobic/van der Waals
**PZ1**	PFGDSYIVIGVGEKKITHHWHRSGS	A	THR267	CG2	ARG22	CB	3.728	DII	H-bond/polar candidate
**PZ1**	PFGDSYIVIGVGEKKITHHWHRSGS	B	PRO222	CD	ARG22	CG	3.893	DII	H-bond/polar candidate
**PZ1**	PFGDSYIVIGVGEKKITHHWHRSGS	B	PRO222	CG	ARG22	CG	3.626	DII	H-bond/polar candidate
**PZ1**	PFGDSYIVIGVGEKKITHHWHRSGS	A	THR267	OG1	ARG22	CD	3.63	DII	H-bond/polar candidate
**PZ1**	PFGDSYIVIGVGEKKITHHWHRSGS	A	THR267	CG2	ARG22	CD	3.778	DII	H-bond/polar candidate
**PZ1**	PFGDSYIVIGVGEKKITHHWHRSGS	B	GLN261	OE1	ARG22	CD	3.775	DII	H-bond/polar candidate
**PZ1**	PFGDSYIVIGVGEKKITHHWHRSGS	B	PRO222	CB	ARG22	NE	3.986	DII	H-bond/polar candidate
**PZ1**	PFGDSYIVIGVGEKKITHHWHRSGS	B	PRO222	O	ARG22	NE	3.263	DII	H-bond/polar candidate
**PZ1**	PFGDSYIVIGVGEKKITHHWHRSGS	B	GLN261	OE1	ARG22	NE	3.695	DII	H-bond/polar candidate
**PZ1**	PFGDSYIVIGVGEKKITHHWHRSGS	B	PRO222	CD	ARG22	HE	3.537	DII	H-bond/polar candidate
**PZ1**	PFGDSYIVIGVGEKKITHHWHRSGS	B	PRO222	CA	ARG22	HE	3.728	DII	H-bond/polar candidate
**PZ1**	PFGDSYIVIGVGEKKITHHWHRSGS	B	PRO222	CB	ARG22	HE	3.264	DII	H-bond/polar candidate
**PZ1**	PFGDSYIVIGVGEKKITHHWHRSGS	B	PRO222	CG	ARG22	HE	3.612	DII	H-bond/polar candidate
**PZ1**	PFGDSYIVIGVGEKKITHHWHRSGS	B	PRO222	C	ARG22	HE	3.321	DII	H-bond/polar candidate
**PZ1**	PFGDSYIVIGVGEKKITHHWHRSGS	B	PRO222	O	ARG22	HE	2.495	DII	H-bond/polar candidate
**PZ1**	PFGDSYIVIGVGEKKITHHWHRSGS	A	THR267	OG1	ARG22	CZ	3.898	DII	H-bond/polar candidate
**PZ1**	PFGDSYIVIGVGEKKITHHWHRSGS	B	PRO222	O	ARG22	CZ	3.45	DII	H-bond/polar candidate
**PZ1**	PFGDSYIVIGVGEKKITHHWHRSGS	B	GLN261	CD	ARG22	CZ	3.981	DII	H-bond/polar candidate
**PZ1**	PFGDSYIVIGVGEKKITHHWHRSGS	B	GLN261	OE1	ARG22	CZ	3.391	DII	H-bond/polar candidate
**PZ1**	PFGDSYIVIGVGEKKITHHWHRSGS	A	THR267	CB	ARG22	NH1	3.929	DII	H-bond/polar candidate
**PZ1**	PFGDSYIVIGVGEKKITHHWHRSGS	A	THR267	OG1	ARG22	NH1	2.758	DII	H-bond/polar candidate
**PZ1**	PFGDSYIVIGVGEKKITHHWHRSGS	A	THR267	HG1	ARG22	NH1	3.319	DII	H-bond/polar candidate
**PZ1**	PFGDSYIVIGVGEKKITHHWHRSGS	B	LEU258	CD2	ARG22	NH1	3.855	DII	H-bond/polar candidate
**PZ1**	PFGDSYIVIGVGEKKITHHWHRSGS	B	GLN261	CD	ARG22	NH1	3.666	DII	H-bond/polar candidate
**PZ1**	PFGDSYIVIGVGEKKITHHWHRSGS	B	GLN261	OE1	ARG22	NH1	3.003	DII	H-bond/polar candidate
**PZ1**	PFGDSYIVIGVGEKKITHHWHRSGS	B	GLN261	HE22	ARG22	NH1	3.729	DII	H-bond/polar candidate
**PZ1**	PFGDSYIVIGVGEKKITHHWHRSGS	A	THR267	CB	ARG22	HH11	3.023	DII	H-bond/polar candidate
**PZ1**	PFGDSYIVIGVGEKKITHHWHRSGS	A	THR267	OG1	ARG22	HH11	1.846	DII	H-bond/polar candidate

**PZ1**	PFGDSYIVIGVGEKKITHHWHRSGS	A	THR267	HG1	ARG22	HH11	2.46	DII	H-bond/polar candidate
**PZ1**	PFGDSYIVIGVGEKKITHHWHRSGS	A	THR267	CG2	ARG22	HH11	3.233	DII	H-bond/polar candidate
**PZ1**	PFGDSYIVIGVGEKKITHHWHRSGS	B	GLN261	CD	ARG22	HH11	3.724	DII	H-bond/polar candidate
**PZ1**	PFGDSYIVIGVGEKKITHHWHRSGS	B	GLN261	OE1	ARG22	HH11	2.814	DII	H-bond/polar candidate
**PZ1**	PFGDSYIVIGVGEKKITHHWHRSGS	B	GLN261	HE22	ARG22	HH11	3.774	DII	H-bond/polar candidate
**PZ1**	PFGDSYIVIGVGEKKITHHWHRSGS	A	THR267	OG1	ARG22	HH12	3.035	DII	H-bond/polar candidate
**PZ1**	PFGDSYIVIGVGEKKITHHWHRSGS	A	THR267	HG1	ARG22	HH12	3.514	DII	H-bond/polar candidate
**PZ1**	PFGDSYIVIGVGEKKITHHWHRSGS	B	ALA241	O	ARG22	HH12	3.835	DII	H-bond/polar candidate
**PZ1**	PFGDSYIVIGVGEKKITHHWHRSGS	B	LEU258	CD2	ARG22	HH12	2.866	DII	H-bond/polar candidate
**PZ1**	PFGDSYIVIGVGEKKITHHWHRSGS	B	GLN261	CD	ARG22	HH12	3.798	DII	H-bond/polar candidate
**PZ1**	PFGDSYIVIGVGEKKITHHWHRSGS	B	GLN261	OE1	ARG22	HH12	3.388	DII	H-bond/polar candidate
**PZ1**	PFGDSYIVIGVGEKKITHHWHRSGS	B	GLN261	NE2	ARG22	HH12	3.801	DII	H-bond/polar candidate
**PZ1**	PFGDSYIVIGVGEKKITHHWHRSGS	B	GLN261	HE22	ARG22	HH12	3.389	DII	H-bond/polar candidate
**PZ1**	PFGDSYIVIGVGEKKITHHWHRSGS	B	PRO222	C	ARG22	NH2	3.973	DII	H-bond/polar candidate
**PZ1**	PFGDSYIVIGVGEKKITHHWHRSGS	B	PRO222	O	ARG22	NH2	2.745	DII	H-bond/polar candidate
**PZ1**	PFGDSYIVIGVGEKKITHHWHRSGS	B	LEU223	CD2	ARG22	NH2	3.779	DII	H-bond/polar candidate
**PZ1**	PFGDSYIVIGVGEKKITHHWHRSGS	B	ALA241	C	ARG22	NH2	3.853	DII	H-bond/polar candidate
**PZ1**	PFGDSYIVIGVGEKKITHHWHRSGS	B	ALA241	O	ARG22	NH2	2.887	DII	H-bond/polar candidate
**PZ1**	PFGDSYIVIGVGEKKITHHWHRSGS	B	PRO222	O	ARG22	HH21	3.429	DII	H-bond/polar candidate
**PZ1**	PFGDSYIVIGVGEKKITHHWHRSGS	B	ALA241	CA	ARG22	HH21	3.789	DII	H-bond/polar candidate
**PZ1**	PFGDSYIVIGVGEKKITHHWHRSGS	B	ALA241	CB	ARG22	HH21	3.883	DII	H-bond/polar candidate
**PZ1**	PFGDSYIVIGVGEKKITHHWHRSGS	B	ALA241	C	ARG22	HH21	3.046	DII	H-bond/polar candidate
**PZ1**	PFGDSYIVIGVGEKKITHHWHRSGS	B	ALA241	O	ARG22	HH21	1.961	DII	H-bond/polar candidate
**PZ1**	PFGDSYIVIGVGEKKITHHWHRSGS	B	LEU258	CD2	ARG22	HH21	3.923	DII	H-bond/polar candidate
**PZ1**	PFGDSYIVIGVGEKKITHHWHRSGS	B	PRO222	CA	ARG22	HH22	3.998	DII	H-bond/polar candidate
**PZ1**	PFGDSYIVIGVGEKKITHHWHRSGS	B	PRO222	CB	ARG22	HH22	3.819	DII	H-bond/polar candidate
**PZ1**	PFGDSYIVIGVGEKKITHHWHRSGS	B	PRO222	C	ARG22	HH22	2.984	DII	H-bond/polar candidate
**PZ1**	PFGDSYIVIGVGEKKITHHWHRSGS	B	PRO222	O	ARG22	HH22	1.756	DII	H-bond/polar candidate
**PZ1**	PFGDSYIVIGVGEKKITHHWHRSGS	B	LEU223	N	ARG22	HH22	3.87	DII	H-bond/polar candidate
**PZ1**	PFGDSYIVIGVGEKKITHHWHRSGS	B	LEU223	CA	ARG22	HH22	3.954	DII	H-bond/polar candidate

**PZ1**	PFGDSYIVIGVGEKKITHHWHRSGS	B	LEU223	CD2	ARG22	HH22	3.326	DII	H-bond/polar candidate
**PZ1**	PFGDSYIVIGVGEKKITHHWHRSGS	B	ALA241	CB	ARG22	HH22	3.6	DII	H-bond/polar candidate
**PZ1**	PFGDSYIVIGVGEKKITHHWHRSGS	B	ALA241	C	ARG22	HH22	3.899	DII	H-bond/polar candidate
**PZ1**	PFGDSYIVIGVGEKKITHHWHRSGS	B	ALA241	O	ARG22	HH22	3.192	DII	H-bond/polar candidate
**PZ1**	PFGDSYIVIGVGEKKITHHWHRSGS	B	PRO222	CD	SER23	N	3.67	DII	H-bond/polar candidate
**PZ1**	PFGDSYIVIGVGEKKITHHWHRSGS	B	PRO222	CG	SER23	N	3.684	DII	H-bond/polar candidate
**PZ1**	PFGDSYIVIGVGEKKITHHWHRSGS	B	PRO222	CD	SER23	H	3.397	DII	H-bond/polar candidate
**PZ1**	PFGDSYIVIGVGEKKITHHWHRSGS	B	PRO222	CG	SER23	H	3.066	DII	H-bond/polar candidate
**PZ1**	PFGDSYIVIGVGEKKITHHWHRSGS	B	TRP217	HE1	SER23	CA	3.911	DII	H-bond/polar candidate
**PZ1**	PFGDSYIVIGVGEKKITHHWHRSGS	B	PRO222	CD	SER23	CA	3.908	DII	H-bond/polar candidate
**PZ1**	PFGDSYIVIGVGEKKITHHWHRSGS	B	ASP220	OD2	SER23	CB	3.425	DII	H-bond/polar candidate
**PZ1**	PFGDSYIVIGVGEKKITHHWHRSGS	B	ASP220	C	SER23	CB	3.764	DII	H-bond/polar candidate
**PZ1**	PFGDSYIVIGVGEKKITHHWHRSGS	B	ASP220	O	SER23	CB	3.397	DII	H-bond/polar candidate
**PZ1**	PFGDSYIVIGVGEKKITHHWHRSGS	B	PRO222	CD	SER23	CB	3.842	DII	H-bond/polar candidate
**PZ1**	PFGDSYIVIGVGEKKITHHWHRSGS	B	ASP220	CG	SER23	OG	3.632	DII	H-bond/polar candidate
**PZ1**	PFGDSYIVIGVGEKKITHHWHRSGS	B	ASP220	OD2	SER23	OG	2.824	DII	H-bond/polar candidate
**PZ1**	PFGDSYIVIGVGEKKITHHWHRSGS	B	ASP220	O	SER23	OG	3.446	DII	H-bond/polar candidate
**PZ1**	PFGDSYIVIGVGEKKITHHWHRSGS	B	PRO222	CG	SER23	OG	3.918	DII	H-bond/polar candidate
**PZ1**	PFGDSYIVIGVGEKKITHHWHRSGS	B	ASP220	CB	SER23	HG	3.415	DII	H-bond/polar candidate
**PZ1**	PFGDSYIVIGVGEKKITHHWHRSGS	B	ASP220	CG	SER23	HG	2.694	DII	H-bond/polar candidate
**PZ1**	PFGDSYIVIGVGEKKITHHWHRSGS	B	ASP220	OD1	SER23	HG	3.516	DII	H-bond/polar candidate
**PZ1**	PFGDSYIVIGVGEKKITHHWHRSGS	B	ASP220	OD2	SER23	HG	1.888	DII	H-bond/polar candidate
**PZ1**	PFGDSYIVIGVGEKKITHHWHRSGS	B	ASP220	C	SER23	HG	3.739	DII	H-bond/polar candidate
**PZ1**	PFGDSYIVIGVGEKKITHHWHRSGS	B	ASP220	O	SER23	HG	3.068	DII	H-bond/polar candidate
**PZ1**	PFGDSYIVIGVGEKKITHHWHRSGS	B	TRP217	NE1	SER23	C	3.892	DII	H-bond/polar candidate
**PZ1**	PFGDSYIVIGVGEKKITHHWHRSGS	B	TRP217	HE1	SER23	C	3.358	DII	H-bond/polar candidate
**PZ1**	PFGDSYIVIGVGEKKITHHWHRSGS	B	PRO222	CD	SER23	C	3.706	DII	H-bond/polar candidate
**PZ1**	PFGDSYIVIGVGEKKITHHWHRSGS	B	TRP217	HE1	SER23	O	3.89	DII	H-bond/polar candidate
**PZ1**	PFGDSYIVIGVGEKKITHHWHRSGS	B	ILE221	N	SER23	O	3.857	DII	H-bond/polar candidate
**PZ1**	PFGDSYIVIGVGEKKITHHWHRSGS	B	ILE221	CA	SER23	O	3.094	DII	H-bond/polar candidate

**PZ1**	PFGDSYIVIGVGEKKITHHWHRSGS	B	ILE221	CB	SER23	O	3.479	DII	H-bond/polar candidate
**PZ1**	PFGDSYIVIGVGEKKITHHWHRSGS	B	ILE221	CG2	SER23	O	3.59	DII	H-bond/polar candidate
**PZ1**	PFGDSYIVIGVGEKKITHHWHRSGS	B	ILE221	CG1	SER23	O	3.385	DII	H-bond/polar candidate
**PZ1**	PFGDSYIVIGVGEKKITHHWHRSGS	B	PRO222	N	SER23	O	3.986	DII	H-bond/polar candidate
**PZ1**	PFGDSYIVIGVGEKKITHHWHRSGS	B	PRO222	CD	SER23	O	3.112	DII	H-bond/polar candidate
**PZ1**	PFGDSYIVIGVGEKKITHHWHRSGS	B	TRP217	NE1	GLY24	N	3.767	DII	hydrophobic/van der Waals
**PZ1**	PFGDSYIVIGVGEKKITHHWHRSGS	B	TRP217	HE1	GLY24	N	3.023	DII	hydrophobic/van der Waals
**PZ1**	PFGDSYIVIGVGEKKITHHWHRSGS	B	TRP217	NE1	GLY24	H	3.979	DII	hydrophobic/van der Waals
**PZ1**	PFGDSYIVIGVGEKKITHHWHRSGS	B	TRP217	HE1	GLY24	H	3.089	DII	hydrophobic/van der Waals
**PZ1**	PFGDSYIVIGVGEKKITHHWHRSGS	B	TRP217	HE1	GLY24	CA	3.635	DII	hydrophobic/van der Waals
**PZ1**	PFGDSYIVIGVGEKKITHHWHRSGS	B	TRP217	CZ2	GLY24	CA	3.846	DII	hydrophobic/van der Waals
**PZ1**	PFGDSYIVIGVGEKKITHHWHRSGS	B	ALA268	CB	GLY24	CA	3.626	DII	hydrophobic/van der Waals
**PZ1**	PFGDSYIVIGVGEKKITHHWHRSGS	B	ALA264	C	GLY24	C	3.782	DII	hydrophobic/van der Waals
**PZ1**	PFGDSYIVIGVGEKKITHHWHRSGS	B	ALA264	O	GLY24	C	3.484	DII	hydrophobic/van der Waals
**PZ1**	PFGDSYIVIGVGEKKITHHWHRSGS	B	ALA268	CB	GLY24	C	3.72	DII	hydrophobic/van der Waals
**PZ1**	PFGDSYIVIGVGEKKITHHWHRSGS	B	ALA264	CA	GLY24	O	3.649	DII	hydrophobic/van der Waals
**PZ1**	PFGDSYIVIGVGEKKITHHWHRSGS	B	ALA264	CB	GLY24	O	3.212	DII	hydrophobic/van der Waals
**PZ1**	PFGDSYIVIGVGEKKITHHWHRSGS	B	ALA264	C	GLY24	O	2.933	DII	hydrophobic/van der Waals
**PZ1**	PFGDSYIVIGVGEKKITHHWHRSGS	B	ALA264	O	GLY24	O	3.011	DII	hydrophobic/van der Waals
**PZ1**	PFGDSYIVIGVGEKKITHHWHRSGS	B	VAL265	N	GLY24	O	3.093	DII	hydrophobic/van der Waals
**PZ1**	PFGDSYIVIGVGEKKITHHWHRSGS	B	VAL265	H	GLY24	O	3.497	DII	hydrophobic/van der Waals
**PZ1**	PFGDSYIVIGVGEKKITHHWHRSGS	B	VAL265	CA	GLY24	O	3.44	DII	hydrophobic/van der Waals
**PZ1**	PFGDSYIVIGVGEKKITHHWHRSGS	B	VAL265	CG2	GLY24	O	3.785	DII	hydrophobic/van der Waals
**PZ1**	PFGDSYIVIGVGEKKITHHWHRSGS	B	ALA264	O	SER25	N	3.701	DII	H-bond/polar candidate
**PZ1**	PFGDSYIVIGVGEKKITHHWHRSGS	B	ALA268	CB	SER25	N	3.933	DII	H-bond/polar candidate
**PZ1**	PFGDSYIVIGVGEKKITHHWHRSGS	B	ALA264	CA	SER25	CA	3.79	DII	H-bond/polar candidate
**PZ1**	PFGDSYIVIGVGEKKITHHWHRSGS	B	ALA264	CB	SER25	CA	3.568	DII	H-bond/polar candidate
**PZ1**	PFGDSYIVIGVGEKKITHHWHRSGS	B	ALA264	C	SER25	CA	3.607	DII	H-bond/polar candidate
**PZ1**	PFGDSYIVIGVGEKKITHHWHRSGS	B	ALA264	O	SER25	CA	3.089	DII	H-bond/polar candidate
**PZ1**	PFGDSYIVIGVGEKKITHHWHRSGS	B	THR267	HG1	SER25	CA	3.918	DII	H-bond/polar candidate

**PZ1**	PFGDSYIVIGVGEKKITHHWHRSGS	B	ALA264	CB	SER25	OG	3.772	DII	H-bond/polar candidate
**PZ1**	PFGDSYIVIGVGEKKITHHWHRSGS	A	THR267	CB	SER25	HG	3.872	DII	H-bond/polar candidate
**PZ1**	PFGDSYIVIGVGEKKITHHWHRSGS	A	THR267	CG2	SER25	HG	3.785	DII	H-bond/polar candidate
**PZ1**	PFGDSYIVIGVGEKKITHHWHRSGS	B	ALA264	C	SER25	C	3.803	DII	H-bond/polar candidate
**PZ1**	PFGDSYIVIGVGEKKITHHWHRSGS	B	ALA264	O	SER25	C	2.876	DII	H-bond/polar candidate
**PZ1**	PFGDSYIVIGVGEKKITHHWHRSGS	B	THR267	OG1	SER25	C	3.278	DII	H-bond/polar candidate
**PZ1**	PFGDSYIVIGVGEKKITHHWHRSGS	B	THR267	HG1	SER25	C	3.053	DII	H-bond/polar candidate
**PZ1**	PFGDSYIVIGVGEKKITHHWHRSGS	B	ALA268	N	SER25	C	3.594	DII	H-bond/polar candidate
**PZ1**	PFGDSYIVIGVGEKKITHHWHRSGS	B	ALA268	H	SER25	C	3.28	DII	H-bond/polar candidate
**PZ1**	PFGDSYIVIGVGEKKITHHWHRSGS	B	ALA268	CA	SER25	C	3.812	DII	H-bond/polar candidate
**PZ1**	PFGDSYIVIGVGEKKITHHWHRSGS	B	ALA268	CB	SER25	C	3.64	DII	H-bond/polar candidate
**PZ1**	PFGDSYIVIGVGEKKITHHWHRSGS	B	ALA264	O	SER25	O	3.692	DII	H-bond/polar candidate
**PZ1**	PFGDSYIVIGVGEKKITHHWHRSGS	B	THR267	HG1	SER25	O	3.903	DII	H-bond/polar candidate
**PZ1**	PFGDSYIVIGVGEKKITHHWHRSGS	B	ALA268	N	SER25	O	3.546	DII	H-bond/polar candidate
**PZ1**	PFGDSYIVIGVGEKKITHHWHRSGS	B	ALA268	H	SER25	O	3.543	DII	H-bond/polar candidate
**PZ1**	PFGDSYIVIGVGEKKITHHWHRSGS	B	ALA268	CA	SER25	O	3.326	DII	H-bond/polar candidate
**PZ1**	PFGDSYIVIGVGEKKITHHWHRSGS	B	ALA268	CB	SER25	O	3.214	DII	H-bond/polar candidate
**PZ2**	PRTGLDFSDLYYLTMNNKHWLVHKE	A	SER146	O	PRO1	C	3.97	DI	H-bond/polar candidate
**PZ2**	PRTGLDFSDLYYLTMNNKHWLVHKE	A	SER146	O	PRO1	O	3.497	DI	H-bond/polar candidate
**PZ2**	PRTGLDFSDLYYLTMNNKHWLVHKE	A	ARG164	CZ	PRO1	O	3.901	DI	H-bond/polar candidate
**PZ2**	PRTGLDFSDLYYLTMNNKHWLVHKE	A	ARG164	NH1	PRO1	O	3.536	DI	H-bond/polar candidate
**PZ2**	PRTGLDFSDLYYLTMNNKHWLVHKE	A	ARG164	HH11	PRO1	O	3.141	DI	H-bond/polar candidate
**PZ2**	PRTGLDFSDLYYLTMNNKHWLVHKE	A	ARG164	NH2	PRO1	O	3.929	DI	H-bond/polar candidate
**PZ2**	PRTGLDFSDLYYLTMNNKHWLVHKE	A	ARG164	HH21	PRO1	O	3.681	DI	H-bond/polar candidate
**PZ2**	PRTGLDFSDLYYLTMNNKHWLVHKE	A	SER146	O	ARG2	N	3.895	DI	H-bond/polar candidate
**PZ2**	PRTGLDFSDLYYLTMNNKHWLVHKE	A	SER146	O	ARG2	CA	3.078	DI	H-bond/polar candidate
**PZ2**	PRTGLDFSDLYYLTMNNKHWLVHKE	A	SER146	O	ARG2	CB	3.861	DI	H-bond/polar candidate
**PZ2**	PRTGLDFSDLYYLTMNNKHWLVHKE	A	GLY145	O	ARG2	CG	3.975	DI	H-bond/polar candidate
**PZ2**	PRTGLDFSDLYYLTMNNKHWLVHKE	A	SER146	O	ARG2	CG	3.663	DI	H-bond/polar candidate
**PZ2**	PRTGLDFSDLYYLTMNNKHWLVHKE	A	MET375	H	ARG2	CD	3.267	DIII	H-bond/polar candidate

**PZ2**	PRTGLDFSDLYYLTMNNKHWLVHKE	A	LYS373	O	ARG2	NE	3.531	DIII	H-bond/polar candidate
**PZ2**	PRTGLDFSDLYYLTMNNKHWLVHKE	A	MET374	CA	ARG2	NE	3.596	DIII	H-bond/polar candidate
**PZ2**	PRTGLDFSDLYYLTMNNKHWLVHKE	A	MET374	C	ARG2	NE	3.967	DIII	H-bond/polar candidate
**PZ2**	PRTGLDFSDLYYLTMNNKHWLVHKE	A	MET375	N	ARG2	NE	3.628	DIII	H-bond/polar candidate
**PZ2**	PRTGLDFSDLYYLTMNNKHWLVHKE	A	MET375	H	ARG2	NE	3.159	DIII	H-bond/polar candidate
**PZ2**	PRTGLDFSDLYYLTMNNKHWLVHKE	A	LYS373	C	ARG2	HE	3.174	DIII	H-bond/polar candidate
**PZ2**	PRTGLDFSDLYYLTMNNKHWLVHKE	A	LYS373	O	ARG2	HE	2.602	DIII	H-bond/polar candidate
**PZ2**	PRTGLDFSDLYYLTMNNKHWLVHKE	A	MET374	N	ARG2	HE	3.31	DIII	H-bond/polar candidate
**PZ2**	PRTGLDFSDLYYLTMNNKHWLVHKE	A	MET374	CA	ARG2	HE	2.854	DIII	H-bond/polar candidate
**PZ2**	PRTGLDFSDLYYLTMNNKHWLVHKE	A	MET374	CB	ARG2	HE	3.915	DIII	H-bond/polar candidate
**PZ2**	PRTGLDFSDLYYLTMNNKHWLVHKE	A	MET374	C	ARG2	HE	3.559	DIII	H-bond/polar candidate
**PZ2**	PRTGLDFSDLYYLTMNNKHWLVHKE	A	MET375	N	ARG2	HE	3.49	DIII	H-bond/polar candidate
**PZ2**	PRTGLDFSDLYYLTMNNKHWLVHKE	A	MET375	H	ARG2	HE	3.033	DIII	H-bond/polar candidate
**PZ2**	PRTGLDFSDLYYLTMNNKHWLVHKE	A	GLU329	OE2	ARG2	CZ	3.858	DIII	salt bridge candidate; H-bond/polar candidate
**PZ2**	PRTGLDFSDLYYLTMNNKHWLVHKE	A	MET375	H	ARG2	CZ	3.837	DIII	H-bond/polar candidate
**PZ2**	PRTGLDFSDLYYLTMNNKHWLVHKE	A	MET375	CG	ARG2	CZ	3.762	DIII	H-bond/polar candidate
**PZ2**	PRTGLDFSDLYYLTMNNKHWLVHKE	A	MET375	CG	ARG2	NH1	3.895	DIII	H-bond/polar candidate
**PZ2**	PRTGLDFSDLYYLTMNNKHWLVHKE	A	MET375	SD	ARG2	NH1	3.572	DIII	H-bond/polar candidate
**PZ2**	PRTGLDFSDLYYLTMNNKHWLVHKE	A	MET375	CE	ARG2	NH1	3.777	DIII	H-bond/polar candidate
**PZ2**	PRTGLDFSDLYYLTMNNKHWLVHKE	A	GLU329	OE2	ARG2	HH11	3.554	DIII	salt bridge candidate; H-bond/polar candidate
**PZ2**	PRTGLDFSDLYYLTMNNKHWLVHKE	A	MET375	CG	ARG2	HH11	3.885	DIII	H-bond/polar candidate
**PZ2**	PRTGLDFSDLYYLTMNNKHWLVHKE	A	MET375	SD	ARG2	HH11	3.316	DIII	H-bond/polar candidate
**PZ2**	PRTGLDFSDLYYLTMNNKHWLVHKE	A	MET375	CE	ARG2	HH11	3.1	DIII	H-bond/polar candidate
**PZ2**	PRTGLDFSDLYYLTMNNKHWLVHKE	A	MET375	SD	ARG2	HH12	3.913	DIII	H-bond/polar candidate
**PZ2**	PRTGLDFSDLYYLTMNNKHWLVHKE	A	GLU329	CG	ARG2	NH2	3.696	DIII	salt bridge candidate; H-bond/polar candidate
**PZ2**	PRTGLDFSDLYYLTMNNKHWLVHKE	A	GLU329	CD	ARG2	NH2	3.539	DIII	salt bridge candidate; H-bond/polar candidate
**PZ2**	PRTGLDFSDLYYLTMNNKHWLVHKE	A	GLU329	OE2	ARG2	NH2	2.819	DIII	salt bridge candidate; H-bond/polar candidate
**PZ2**	PRTGLDFSDLYYLTMNNKHWLVHKE	A	LYS373	CB	ARG2	NH2	3.491	DIII	H-bond/polar candidate
**PZ2**	PRTGLDFSDLYYLTMNNKHWLVHKE	A	LYS373	C	ARG2	NH2	3.677	DIII	H-bond/polar candidate
**PZ2**	PRTGLDFSDLYYLTMNNKHWLVHKE	A	LYS373	O	ARG2	NH2	3.829	DIII	H-bond/polar candidate

**PZ2**	PRTGLDFSDLYYLTMNNKHWLVHKE	A	MET374	N	ARG2	NH2	3.678		DIII	H-bond/polar candidate
**PZ2**	PRTGLDFSDLYYLTMNNKHWLVHKE	A	MET374	CA	ARG2	NH2	3.868		DIII	H-bond/polar candidate
**PZ2**	PRTGLDFSDLYYLTMNNKHWLVHKE	A	MET374	C	ARG2	NH2	3.947		DIII	H-bond/polar candidate
**PZ2**	PRTGLDFSDLYYLTMNNKHWLVHKE	A	MET375	CG	ARG2	NH2	3.787		DIII	H-bond/polar candidate
**PZ2**	PRTGLDFSDLYYLTMNNKHWLVHKE	A	LYS316	HZ3	ARG2	HH21	3.428		DIII	H-bond/polar candidate
**PZ2**	PRTGLDFSDLYYLTMNNKHWLVHKE	A	GLU329	CG	ARG2	HH21	3.242		DIII	salt bridge candidate; H-bond/polar candidate
**PZ2**	PRTGLDFSDLYYLTMNNKHWLVHKE	A	GLU329	CD	ARG2	HH21	2.752		DIII	salt bridge candidate; H-bond/polar candidate
**PZ2**	PRTGLDFSDLYYLTMNNKHWLVHKE	A	GLU329	OE1	ARG2	HH21	3.764		DIII	salt bridge candidate; H-bond/polar candidate
**PZ2**	PRTGLDFSDLYYLTMNNKHWLVHKE	A	GLU329	OE2	ARG2	HH21	1.869		DIII	salt bridge candidate; H-bond/polar candidate
**PZ2**	PRTGLDFSDLYYLTMNNKHWLVHKE	A	LYS373	CB	ARG2	HH21	3.651		DIII	H-bond/polar candidate
**PZ2**	PRTGLDFSDLYYLTMNNKHWLVHKE	A	MET375	CG	ARG2	HH21	3.804		DIII	H-bond/polar candidate
**PZ2**	PRTGLDFSDLYYLTMNNKHWLVHKE	A	MET375	CE	ARG2	HH21	3.716		DIII	H-bond/polar candidate
**PZ2**	PRTGLDFSDLYYLTMNNKHWLVHKE	A	GLU329	CG	ARG2	HH22	3.533		DIII	salt bridge candidate; H-bond/polar candidate
**PZ2**	PRTGLDFSDLYYLTMNNKHWLVHKE	A	GLU329	CD	ARG2	HH22	3.638		DIII	salt bridge candidate; H-bond/polar candidate
**PZ2**	PRTGLDFSDLYYLTMNNKHWLVHKE	A	GLU329	OE2	ARG2	HH22	3.229		DIII	salt bridge candidate; H-bond/polar candidate
**PZ2**	PRTGLDFSDLYYLTMNNKHWLVHKE	A	LYS373	CA	ARG2	HH22	3.388		DIII	H-bond/polar candidate
**PZ2**	PRTGLDFSDLYYLTMNNKHWLVHKE	A	LYS373	CB	ARG2	HH22	2.783		DIII	H-bond/polar candidate
**PZ2**	PRTGLDFSDLYYLTMNNKHWLVHKE	A	LYS373	C	ARG2	HH22	2.712		DIII	H-bond/polar candidate
**PZ2**	PRTGLDFSDLYYLTMNNKHWLVHKE	A	LYS373	O	ARG2	HH22	3.019		DIII	H-bond/polar candidate
**PZ2**	PRTGLDFSDLYYLTMNNKHWLVHKE	A	MET374	N	ARG2	HH22	2.715		DIII	H-bond/polar candidate
**PZ2**	PRTGLDFSDLYYLTMNNKHWLVHKE	A	MET374	H	ARG2	HH22	3.22		DIII	H-bond/polar candidate
**PZ2**	PRTGLDFSDLYYLTMNNKHWLVHKE	A	MET374	CA	ARG2	HH22	3.099		DIII	H-bond/polar candidate
**PZ2**	PRTGLDFSDLYYLTMNNKHWLVHKE	A	MET374	C	ARG2	HH22	3.4		DIII	H-bond/polar candidate
**PZ2**	PRTGLDFSDLYYLTMNNKHWLVHKE	A	MET374	O	ARG2	HH22	3.932		DIII	H-bond/polar candidate
**PZ2**	PRTGLDFSDLYYLTMNNKHWLVHKE	A	MET375	N	ARG2	HH22	3.785		DIII	H-bond/polar candidate
**PZ2**	PRTGLDFSDLYYLTMNNKHWLVHKE	A	MET375	H	ARG2	HH22	3.954		DIII	H-bond/polar candidate
**PZ2**	PRTGLDFSDLYYLTMNNKHWLVHKE	A	SER146	O	ARG2	C	3.623		DI	H-bond/polar candidate
**PZ2**	PRTGLDFSDLYYLTMNNKHWLVHKE	A	SER146	O	THR3	N	3.576		DI	H-bond/polar candidate
**PZ2**	PRTGLDFSDLYYLTMNNKHWLVHKE	A	ARG164	HH11	THR3	N	3.464		DI	H-bond/polar candidate
**PZ2**	PRTGLDFSDLYYLTMNNKHWLVHKE	A	ARG164	NH2	THR3	N	3.859		DI	H-bond/polar candidate

**PZ2**	PRTGLDFSDLYYLTMNNKHWLVHKE	A	ARG164	HH21	THR3	N	3.019	DI	H-bond/polar candidate
**PZ2**	PRTGLDFSDLYYLTMNNKHWLVHKE	A	SER146	O	THR3	H	2.994	DI	H-bond/polar candidate
**PZ2**	PRTGLDFSDLYYLTMNNKHWLVHKE	A	ARG164	CZ	THR3	H	3.663	DI	H-bond/polar candidate
**PZ2**	PRTGLDFSDLYYLTMNNKHWLVHKE	A	ARG164	NH1	THR3	H	3.478	DI	H-bond/polar candidate
**PZ2**	PRTGLDFSDLYYLTMNNKHWLVHKE	A	ARG164	HH11	THR3	H	2.83	DI	H-bond/polar candidate
**PZ2**	PRTGLDFSDLYYLTMNNKHWLVHKE	A	ARG164	NH2	THR3	H	3.168	DI	H-bond/polar candidate
**PZ2**	PRTGLDFSDLYYLTMNNKHWLVHKE	A	ARG164	HH21	THR3	H	2.493	DI	H-bond/polar candidate
**PZ2**	PRTGLDFSDLYYLTMNNKHWLVHKE	A	ARG164	HH22	THR3	H	3.833	DI	H-bond/polar candidate
**PZ2**	PRTGLDFSDLYYLTMNNKHWLVHKE	A	ARG164	HH11	THR3	CA	3.422	DI	H-bond/polar candidate
**PZ2**	PRTGLDFSDLYYLTMNNKHWLVHKE	A	ARG164	HH21	THR3	CA	3.361	DI	H-bond/polar candidate
**PZ2**	PRTGLDFSDLYYLTMNNKHWLVHKE	A	ARG164	NH1	THR3	CB	3.955	DI	H-bond/polar candidate
**PZ2**	PRTGLDFSDLYYLTMNNKHWLVHKE	A	ARG164	HH11	THR3	CB	2.95	DI	H-bond/polar candidate
**PZ2**	PRTGLDFSDLYYLTMNNKHWLVHKE	A	ARG164	HH21	THR3	CB	3.948	DI	H-bond/polar candidate
**PZ2**	PRTGLDFSDLYYLTMNNKHWLVHKE	A	ARG164	CZ	THR3	OG1	3.678	DI	H-bond/polar candidate
**PZ2**	PRTGLDFSDLYYLTMNNKHWLVHKE	A	ARG164	NH1	THR3	OG1	2.692	DI	H-bond/polar candidate
**PZ2**	PRTGLDFSDLYYLTMNNKHWLVHKE	A	ARG164	HH11	THR3	OG1	1.74	DI	H-bond/polar candidate
**PZ2**	PRTGLDFSDLYYLTMNNKHWLVHKE	A	ARG164	HH12	THR3	OG1	3.157	DI	H-bond/polar candidate
**PZ2**	PRTGLDFSDLYYLTMNNKHWLVHKE	A	ARG164	NH2	THR3	OG1	3.876	DI	H-bond/polar candidate
**PZ2**	PRTGLDFSDLYYLTMNNKHWLVHKE	A	ARG164	HH21	THR3	OG1	3.357	DI	H-bond/polar candidate
**PZ2**	PRTGLDFSDLYYLTMNNKHWLVHKE	A	ARG164	CZ	THR3	HG1	3.898	DI	H-bond/polar candidate
**PZ2**	PRTGLDFSDLYYLTMNNKHWLVHKE	A	ARG164	NH1	THR3	HG1	3.017	DI	H-bond/polar candidate
**PZ2**	PRTGLDFSDLYYLTMNNKHWLVHKE	A	ARG164	HH11	THR3	HG1	2.224	DI	H-bond/polar candidate
**PZ2**	PRTGLDFSDLYYLTMNNKHWLVHKE	A	ARG164	HH12	THR3	HG1	3.446	DI	H-bond/polar candidate
**PZ2**	PRTGLDFSDLYYLTMNNKHWLVHKE	A	ARG164	HH21	THR3	HG1	3.636	DI	H-bond/polar candidate
**PZ2**	PRTGLDFSDLYYLTMNNKHWLVHKE	A	ILE1	N	THR3	C	3.21	DI	H-bond/polar candidate
**PZ2**	PRTGLDFSDLYYLTMNNKHWLVHKE	A	ILE1	H	THR3	C	3.638	DI	H-bond/polar candidate
**PZ2**	PRTGLDFSDLYYLTMNNKHWLVHKE	A	ARG164	HH11	THR3	C	3.307	DI	H-bond/polar candidate
**PZ2**	PRTGLDFSDLYYLTMNNKHWLVHKE	A	ARG164	NH2	THR3	C	3.69	DI	H-bond/polar candidate
**PZ2**	PRTGLDFSDLYYLTMNNKHWLVHKE	A	ARG164	HH21	THR3	C	2.73	DI	H-bond/polar candidate
**PZ2**	PRTGLDFSDLYYLTMNNKHWLVHKE	A	ILE1	N	THR3	O	3.016	DI	H-bond/polar candidate

**PZ2**	PRTGLDFSDLYYLTMNNKHWLVHKE	A	ILE1	H	THR3	O	3.684	DI	H-bond/polar candidate
**PZ2**	PRTGLDFSDLYYLTMNNKHWLVHKE	A	ILE1	CA	THR3	O	3.398	DI	H-bond/polar candidate
**PZ2**	PRTGLDFSDLYYLTMNNKHWLVHKE	A	ILE1	C	THR3	O	3.909	DI	H-bond/polar candidate
**PZ2**	PRTGLDFSDLYYLTMNNKHWLVHKE	A	ARG164	CZ	THR3	O	3.506	DI	H-bond/polar candidate
**PZ2**	PRTGLDFSDLYYLTMNNKHWLVHKE	A	ARG164	NH1	THR3	O	3.606	DI	H-bond/polar candidate
**PZ2**	PRTGLDFSDLYYLTMNNKHWLVHKE	A	ARG164	HH11	THR3	O	3.058	DI	H-bond/polar candidate
**PZ2**	PRTGLDFSDLYYLTMNNKHWLVHKE	A	ARG164	NH2	THR3	O	2.636	DI	H-bond/polar candidate
**PZ2**	PRTGLDFSDLYYLTMNNKHWLVHKE	A	ARG164	HH21	THR3	O	1.688	DI	H-bond/polar candidate
**PZ2**	PRTGLDFSDLYYLTMNNKHWLVHKE	A	ARG164	HH22	THR3	O	3.21	DI	H-bond/polar candidate
**PZ2**	PRTGLDFSDLYYLTMNNKHWLVHKE	A	ILE1	N	GLY4	N	3.461	DI	hydrophobic/van der Waals
**PZ2**	PRTGLDFSDLYYLTMNNKHWLVHKE	A	ILE1	H	GLY4	N	3.528	DI	hydrophobic/van der Waals
**PZ2**	PRTGLDFSDLYYLTMNNKHWLVHKE	A	ARG164	HH21	GLY4	N	3.854	DI	H-bond/polar candidate
**PZ2**	PRTGLDFSDLYYLTMNNKHWLVHKE	A	ILE1	N	GLY4	CA	3.32	DI	hydrophobic/van der Waals
**PZ2**	PRTGLDFSDLYYLTMNNKHWLVHKE	A	ILE1	H	GLY4	CA	3.255	DI	hydrophobic/van der Waals
**PZ2**	PRTGLDFSDLYYLTMNNKHWLVHKE	A	ILE1	CA	GLY4	CA	3.878	DI	hydrophobic/van der Waals
**PZ2**	PRTGLDFSDLYYLTMNNKHWLVHKE	A	ILE1	C	GLY4	CA	3.517	DI	hydrophobic/van der Waals
**PZ2**	PRTGLDFSDLYYLTMNNKHWLVHKE	A	ILE1	O	GLY4	CA	3.358	DI	hydrophobic/van der Waals
**PZ2**	PRTGLDFSDLYYLTMNNKHWLVHKE	A	ARG2	CG	GLY4	CA	3.751	DI	H-bond/polar candidate
**PZ2**	PRTGLDFSDLYYLTMNNKHWLVHKE	A	ARG2	CG	GLY4	C	3.681	DI	H-bond/polar candidate
**PZ2**	PRTGLDFSDLYYLTMNNKHWLVHKE	A	ARG2	NH1	GLY4	C	3.726	DI	H-bond/polar candidate
**PZ2**	PRTGLDFSDLYYLTMNNKHWLVHKE	A	ARG2	HH12	GLY4	C	2.948	DI	H-bond/polar candidate
**PZ2**	PRTGLDFSDLYYLTMNNKHWLVHKE	A	ARG2	CG	GLY4	O	3.59	DI	H-bond/polar candidate
**PZ2**	PRTGLDFSDLYYLTMNNKHWLVHKE	A	ARG2	CD	GLY4	O	3.738	DI	H-bond/polar candidate
**PZ2**	PRTGLDFSDLYYLTMNNKHWLVHKE	A	ARG2	CZ	GLY4	O	3.781	DI	H-bond/polar candidate
**PZ2**	PRTGLDFSDLYYLTMNNKHWLVHKE	A	ARG2	NH1	GLY4	O	2.751	DI	H-bond/polar candidate
**PZ2**	PRTGLDFSDLYYLTMNNKHWLVHKE	A	ARG2	HH11	GLY4	O	3.116	DI	H-bond/polar candidate
**PZ2**	PRTGLDFSDLYYLTMNNKHWLVHKE	A	ARG2	HH12	GLY4	O	1.904	DI	H-bond/polar candidate
**PZ2**	PRTGLDFSDLYYLTMNNKHWLVHKE	A	ILE1	O	LEU5	N	3.932	DI	hydrophobic/van der Waals
**PZ2**	PRTGLDFSDLYYLTMNNKHWLVHKE	A	ARG2	HH12	LEU5	N	3.718	DI	H-bond/polar candidate
**PZ2**	PRTGLDFSDLYYLTMNNKHWLVHKE	A	ILE1	O	LEU5	H	3.392	DI	hydrophobic/van der Waals

**PZ2**	PRTGLDFSDLYYLTMNNKHWLVHKE	A	ARG2	NH1	LEU5	CA	3.917	DI	H-bond/polar candidate
**PZ2**	PRTGLDFSDLYYLTMNNKHWLVHKE	A	ARG2	HH11	LEU5	CA	3.913	DI	H-bond/polar candidate
**PZ2**	PRTGLDFSDLYYLTMNNKHWLVHKE	A	ARG2	HH12	LEU5	CA	3.702	DI	H-bond/polar candidate
**PZ2**	PRTGLDFSDLYYLTMNNKHWLVHKE	A	CYS3	N	LEU5	CG	3.63	DI	H-bond/polar candidate
**PZ2**	PRTGLDFSDLYYLTMNNKHWLVHKE	A	CYS3	H	LEU5	CG	2.926	DI	H-bond/polar candidate
**PZ2**	PRTGLDFSDLYYLTMNNKHWLVHKE	A	CYS3	N	LEU5	CD1	3.563	DI	H-bond/polar candidate
**PZ2**	PRTGLDFSDLYYLTMNNKHWLVHKE	A	CYS3	H	LEU5	CD1	3.163	DI	H-bond/polar candidate
**PZ2**	PRTGLDFSDLYYLTMNNKHWLVHKE	A	CYS3	CA	LEU5	CD1	3.919	DI	H-bond/polar candidate
**PZ2**	PRTGLDFSDLYYLTMNNKHWLVHKE	A	GLY5	N	LEU5	CD1	3.55	DI	hydrophobic/van der Waals
**PZ2**	PRTGLDFSDLYYLTMNNKHWLVHKE	A	GLY5	H	LEU5	CD1	2.73	DI	hydrophobic/van der Waals
**PZ2**	PRTGLDFSDLYYLTMNNKHWLVHKE	A	GLY5	CA	LEU5	CD1	3.854	DI	hydrophobic/van der Waals
**PZ2**	PRTGLDFSDLYYLTMNNKHWLVHKE	A	VAL6	CG2	LEU5	CD1	3.773	DI	hydrophobic/van der Waals
**PZ2**	PRTGLDFSDLYYLTMNNKHWLVHKE	A	ARG2	CZ	LEU5	CD2	3.658	DI	H-bond/polar candidate
**PZ2**	PRTGLDFSDLYYLTMNNKHWLVHKE	A	ARG2	NH1	LEU5	CD2	3.896	DI	H-bond/polar candidate
**PZ2**	PRTGLDFSDLYYLTMNNKHWLVHKE	A	ARG2	NH2	LEU5	CD2	3.754	DI	H-bond/polar candidate
**PZ2**	PRTGLDFSDLYYLTMNNKHWLVHKE	A	CYS3	N	LEU5	CD2	3.626	DI	H-bond/polar candidate
**PZ2**	PRTGLDFSDLYYLTMNNKHWLVHKE	A	CYS3	H	LEU5	CD2	2.692	DI	H-bond/polar candidate
**PZ2**	PRTGLDFSDLYYLTMNNKHWLVHKE	A	GLU44	OE1	LEU5	CD2	3.875	DI	H-bond/polar candidate
**PZ2**	PRTGLDFSDLYYLTMNNKHWLVHKE	B	GLY102	O	LEU5	O	3.594	fusion-loop region	hydrophobic/van der Waals
**PZ2**	PRTGLDFSDLYYLTMNNKHWLVHKE	A	ARG2	HH11	ASP6	N	3.851	DI	salt bridge candidate; H-bond/polar candidate
**PZ2**	PRTGLDFSDLYYLTMNNKHWLVHKE	A	ARG2	NH1	ASP6	H	3.418	DI	salt bridge candidate; H-bond/polar candidate
**PZ2**	PRTGLDFSDLYYLTMNNKHWLVHKE	A	ARG2	HH11	ASP6	H	2.96	DI	salt bridge candidate; H-bond/polar candidate
**PZ2**	PRTGLDFSDLYYLTMNNKHWLVHKE	A	ARG2	HH12	ASP6	H	3.224	DI	salt bridge candidate; H-bond/polar candidate
**PZ2**	PRTGLDFSDLYYLTMNNKHWLVHKE	B	GLY102	C	PHE7	CD2	3.846	fusion-loop region	hydrophobic/van der Waals
**PZ2**	PRTGLDFSDLYYLTMNNKHWLVHKE	B	GLY102	O	PHE7	CD2	3.315	fusion-loop region	hydrophobic/van der Waals

**PZ2**	PRTGLDFSDLYYLTMNNKHWLVHKE	B	ASN103	CB	PHE7	CD2	3.785	fusion-loop region	H-bond/polar candidate
**PZ2**	PRTGLDFSDLYYLTMNNKHWLVHKE	B	ASN103	HD21	PHE7	CD2	3.882	fusion-loop region	H-bond/polar candidate
**PZ2**	PRTGLDFSDLYYLTMNNKHWLVHKE	B	ASN103	HD21	PHE7	CE1	3.488	fusion-loop region	H-bond/polar candidate
**PZ2**	PRTGLDFSDLYYLTMNNKHWLVHKE	B	GLY102	C	PHE7	CE2	3.86	fusion-loop region	hydrophobic/van der Waals
**PZ2**	PRTGLDFSDLYYLTMNNKHWLVHKE	B	GLY102	O	PHE7	CE2	3.754	fusion-loop region	hydrophobic/van der Waals
**PZ2**	PRTGLDFSDLYYLTMNNKHWLVHKE	B	ASN103	CB	PHE7	CE2	3.884	fusion-loop region	H-bond/polar candidate
**PZ2**	PRTGLDFSDLYYLTMNNKHWLVHKE	B	ASN103	HD21	PHE7	CE2	3.288	fusion-loop region	H-bond/polar candidate
**PZ2**	PRTGLDFSDLYYLTMNNKHWLVHKE	B	ASN103	ND2	PHE7	CZ	4.0	fusion-loop region	H-bond/polar candidate
**PZ2**	PRTGLDFSDLYYLTMNNKHWLVHKE	B	ASN103	HD21	PHE7	CZ	3.056	fusion-loop region	H-bond/polar candidate
**PZ2**	PRTGLDFSDLYYLTMNNKHWLVHKE	B	HIS249	NE2	TYR11	CA	3.839	DII	H-bond/polar candidate
**PZ2**	PRTGLDFSDLYYLTMNNKHWLVHKE	B	HIS249	NE2	TYR11	CB	3.512	DII	H-bond/polar candidate
**PZ2**	PRTGLDFSDLYYLTMNNKHWLVHKE	B	HIS249	NE2	TYR11	CG	3.99	DII	H-bond/polar candidate
**PZ2**	PRTGLDFSDLYYLTMNNKHWLVHKE	B	HIS249	CD2	TYR11	CD1	3.808	DII	H-bond/polar candidate
**PZ2**	PRTGLDFSDLYYLTMNNKHWLVHKE	B	HIS249	NE2	TYR11	CD1	3.638	DII	H-bond/polar candidate
**PZ2**	PRTGLDFSDLYYLTMNNKHWLVHKE	B	ARG252	HE	TYR11	CD1	3.996	DII	H-bond/polar candidate
**PZ2**	PRTGLDFSDLYYLTMNNKHWLVHKE	B	HIS249	CG	TYR12	N	3.906	DII	H-bond/polar candidate
**PZ2**	PRTGLDFSDLYYLTMNNKHWLVHKE	B	HIS249	ND1	TYR12	N	3.381	DII	H-bond/polar candidate
**PZ2**	PRTGLDFSDLYYLTMNNKHWLVHKE	B	HIS249	HD1	TYR12	N	3.52	DII	H-bond/polar candidate
**PZ2**	PRTGLDFSDLYYLTMNNKHWLVHKE	B	HIS249	NE2	TYR12	N	3.808	DII	H-bond/polar candidate

**PZ2**	PRTGLDFSDLYYLTMNNKHWLVHKE	B	HIS249	CE1	TYR12	N	3.306	DII	H-bond/polar candidate
**PZ2**	PRTGLDFSDLYYLTMNNKHWLVHKE	B	HIS249	CB	TYR12	H	3.922	DII	H-bond/polar candidate
**PZ2**	PRTGLDFSDLYYLTMNNKHWLVHKE	B	HIS249	CG	TYR12	H	2.915	DII	H-bond/polar candidate
**PZ2**	PRTGLDFSDLYYLTMNNKHWLVHKE	B	HIS249	ND1	TYR12	H	2.476	DII	H-bond/polar candidate
**PZ2**	PRTGLDFSDLYYLTMNNKHWLVHKE	B	HIS249	HD1	TYR12	H	2.764	DII	H-bond/polar candidate
**PZ2**	PRTGLDFSDLYYLTMNNKHWLVHKE	B	HIS249	CD2	TYR12	H	3.185	DII	H-bond/polar candidate
**PZ2**	PRTGLDFSDLYYLTMNNKHWLVHKE	B	HIS249	NE2	TYR12	H	2.973	DII	H-bond/polar candidate
**PZ2**	PRTGLDFSDLYYLTMNNKHWLVHKE	B	HIS249	CE1	TYR12	H	2.51	DII	H-bond/polar candidate
**PZ2**	PRTGLDFSDLYYLTMNNKHWLVHKE	B	HIS249	ND1	TYR12	CA	3.939	DII	H-bond/polar candidate
**PZ2**	PRTGLDFSDLYYLTMNNKHWLVHKE	B	HIS249	HD1	TYR12	CA	3.736	DII	H-bond/polar candidate
**PZ2**	PRTGLDFSDLYYLTMNNKHWLVHKE	B	HIS249	CE1	TYR12	CA	3.93	DII	H-bond/polar candidate
**PZ2**	PRTGLDFSDLYYLTMNNKHWLVHKE	B	ASP247	CB	TYR12	CB	3.951	DII	H-bond/polar candidate
**PZ2**	PRTGLDFSDLYYLTMNNKHWLVHKE	B	ALA248	O	TYR12	CB	3.462	DII	H-bond/polar candidate; hydrophobic/van der Waals
**PZ2**	PRTGLDFSDLYYLTMNNKHWLVHKE	B	HIS249	ND1	TYR12	CB	3.655	DII	H-bond/polar candidate
**PZ2**	PRTGLDFSDLYYLTMNNKHWLVHKE	B	HIS249	HD1	TYR12	CB	3.096	DII	H-bond/polar candidate
**PZ2**	PRTGLDFSDLYYLTMNNKHWLVHKE	B	ALA248	O	TYR12	CG	3.153	DII	H-bond/polar candidate; hydrophobic/van der Waals
**PZ2**	PRTGLDFSDLYYLTMNNKHWLVHKE	B	HIS249	HD1	TYR12	CG	3.604	DII	H-bond/polar candidate
**PZ2**	PRTGLDFSDLYYLTMNNKHWLVHKE	B	ALA248	O	TYR12	CD1	3.793	DII	H-bond/polar candidate; hydrophobic/van der Waals
**PZ2**	PRTGLDFSDLYYLTMNNKHWLVHKE	B	ALA248	C	TYR12	CD2	3.895	DII	H-bond/polar candidate; hydrophobic/van der Waals
**PZ2**	PRTGLDFSDLYYLTMNNKHWLVHKE	B	ALA248	O	TYR12	CD2	3.053	DII	H-bond/polar candidate; hydrophobic/van der Waals
**PZ2**	PRTGLDFSDLYYLTMNNKHWLVHKE	B	HIS249	CA	TYR12	CD2	3.449	DII	H-bond/polar candidate
**PZ2**	PRTGLDFSDLYYLTMNNKHWLVHKE	B	HIS249	CB	TYR12	CD2	3.863	DII	H-bond/polar candidate
**PZ2**	PRTGLDFSDLYYLTMNNKHWLVHKE	B	HIS249	CG	TYR12	CD2	3.951	DII	H-bond/polar candidate
**PZ2**	PRTGLDFSDLYYLTMNNKHWLVHKE	B	HIS249	ND1	TYR12	CD2	3.734	DII	H-bond/polar candidate
**PZ2**	PRTGLDFSDLYYLTMNNKHWLVHKE	B	HIS249	HD1	TYR12	CD2	3.332	DII	H-bond/polar candidate
**PZ2**	PRTGLDFSDLYYLTMNNKHWLVHKE	B	ALA248	O	TYR12	CE2	3.556	DII	H-bond/polar candidate; hydrophobic/van der Waals
**PZ2**	PRTGLDFSDLYYLTMNNKHWLVHKE	B	HIS249	CA	TYR12	CE2	3.678	DII	H-bond/polar candidate
**PZ2**	PRTGLDFSDLYYLTMNNKHWLVHKE	B	ASP98	H	TYR12	OH	3.434	fusion-loop region	H-bond/polar candidate

**PZ2**	PRTGLDFSDLYYLTMNNKHWLVHKE	B	ASP98	CB	TYR12	OH	3.621	fusion-loop region	H-bond/polar candidate
**PZ2**	PRTGLDFSDLYYLTMNNKHWLVHKE	B	ASP98	OD2	TYR12	OH	3.871	fusion-loop region	H-bond/polar candidate
**PZ2**	PRTGLDFSDLYYLTMNNKHWLVHKE	B	ASP98	N	TYR12	HH	3.952	fusion-loop region	H-bond/polar candidate
**PZ2**	PRTGLDFSDLYYLTMNNKHWLVHKE	B	ASP98	H	TYR12	HH	3.155	fusion-loop region	H-bond/polar candidate
**PZ2**	PRTGLDFSDLYYLTMNNKHWLVHKE	B	ASP98	CB	TYR12	HH	3.236	fusion-loop region	H-bond/polar candidate
**PZ2**	PRTGLDFSDLYYLTMNNKHWLVHKE	B	ASP98	CG	TYR12	HH	3.341	fusion-loop region	H-bond/polar candidate
**PZ2**	PRTGLDFSDLYYLTMNNKHWLVHKE	B	ASP98	OD2	TYR12	HH	3.085	fusion-loop region	H-bond/polar candidate
**PZ2**	PRTGLDFSDLYYLTMNNKHWLVHKE	B	HIS249	CE1	TYR12	C	3.787	DII	H-bond/polar candidate
**PZ2**	PRTGLDFSDLYYLTMNNKHWLVHKE	B	ASP247	CB	TYR12	O	3.761	DII	H-bond/polar candidate
**PZ2**	PRTGLDFSDLYYLTMNNKHWLVHKE	B	ASP247	OD2	TYR12	O	3.934	DII	H-bond/polar candidate
**PZ2**	PRTGLDFSDLYYLTMNNKHWLVHKE	B	HIS249	ND1	TYR12	O	3.532	DII	H-bond/polar candidate
**PZ2**	PRTGLDFSDLYYLTMNNKHWLVHKE	B	HIS249	HD1	TYR12	O	3.468	DII	H-bond/polar candidate
**PZ2**	PRTGLDFSDLYYLTMNNKHWLVHKE	B	HIS249	NE2	TYR12	O	3.959	DII	H-bond/polar candidate
**PZ2**	PRTGLDFSDLYYLTMNNKHWLVHKE	B	HIS249	CE1	TYR12	O	2.904	DII	H-bond/polar candidate
**PZ2**	PRTGLDFSDLYYLTMNNKHWLVHKE	B	ASP247	CG	LEU13	CA	3.877	DII	H-bond/polar candidate
**PZ2**	PRTGLDFSDLYYLTMNNKHWLVHKE	B	ASP247	OD2	LEU13	CA	3.457	DII	H-bond/polar candidate
**PZ2**	PRTGLDFSDLYYLTMNNKHWLVHKE	A	GLU276	CD	LEU13	CG	3.749	DII	H-bond/polar candidate
**PZ2**	PRTGLDFSDLYYLTMNNKHWLVHKE	A	GLU276	OE1	LEU13	CG	3.46	DII	H-bond/polar candidate
**PZ2**	PRTGLDFSDLYYLTMNNKHWLVHKE	B	ASP247	OD2	LEU13	CD1	3.915	DII	H-bond/polar candidate
**PZ2**	PRTGLDFSDLYYLTMNNKHWLVHKE	A	GLU276	OE1	LEU13	CD2	3.617	DII	H-bond/polar candidate
**PZ2**	PRTGLDFSDLYYLTMNNKHWLVHKE	B	ASP247	CG	LEU13	C	3.551	DII	H-bond/polar candidate

**PZ2**	PRTGLDFSDLYYLTMNNKHWLVHKE	B	ASP247	OD1	LEU13	C	3.833	DII	H-bond/polar candidate
**PZ2**	PRTGLDFSDLYYLTMNNKHWLVHKE	B	ASP247	OD2	LEU13	C	3.387	DII	H-bond/polar candidate
**PZ2**	PRTGLDFSDLYYLTMNNKHWLVHKE	B	ASP247	CG	THR14	N	3.193	DII	H-bond/polar candidate
**PZ2**	PRTGLDFSDLYYLTMNNKHWLVHKE	B	ASP247	OD1	THR14	N	3.285	DII	H-bond/polar candidate
**PZ2**	PRTGLDFSDLYYLTMNNKHWLVHKE	B	ASP247	OD2	THR14	N	2.848	DII	H-bond/polar candidate
**PZ2**	PRTGLDFSDLYYLTMNNKHWLVHKE	B	ASP247	CG	THR14	H	2.924	DII	H-bond/polar candidate
**PZ2**	PRTGLDFSDLYYLTMNNKHWLVHKE	B	ASP247	OD1	THR14	H	3.225	DII	H-bond/polar candidate
**PZ2**	PRTGLDFSDLYYLTMNNKHWLVHKE	B	ASP247	OD2	THR14	H	2.232	DII	H-bond/polar candidate
**PZ2**	PRTGLDFSDLYYLTMNNKHWLVHKE	B	ASP247	CG	THR14	CA	3.752	DII	H-bond/polar candidate
**PZ2**	PRTGLDFSDLYYLTMNNKHWLVHKE	B	ASP247	OD1	THR14	CA	3.336	DII	H-bond/polar candidate
**PZ2**	PRTGLDFSDLYYLTMNNKHWLVHKE	B	ASP247	OD2	THR14	CA	3.696	DII	H-bond/polar candidate
**PZ2**	PRTGLDFSDLYYLTMNNKHWLVHKE	A	GLU274	OE1	THR14	CB	3.173	DII	H-bond/polar candidate
**PZ2**	PRTGLDFSDLYYLTMNNKHWLVHKE	B	LYS246	NZ	THR14	CB	3.698	DII	H-bond/polar candidate
**PZ2**	PRTGLDFSDLYYLTMNNKHWLVHKE	B	LYS246	HZ2	THR14	CB	3.817	DII	H-bond/polar candidate
**PZ2**	PRTGLDFSDLYYLTMNNKHWLVHKE	B	LYS246	HZ3	THR14	CB	2.725	DII	H-bond/polar candidate
**PZ2**	PRTGLDFSDLYYLTMNNKHWLVHKE	B	ASP247	CG	THR14	CB	3.892	DII	H-bond/polar candidate
**PZ2**	PRTGLDFSDLYYLTMNNKHWLVHKE	B	ASP247	OD1	THR14	CB	3.283	DII	H-bond/polar candidate
**PZ2**	PRTGLDFSDLYYLTMNNKHWLVHKE	B	ASP247	OD2	THR14	CB	3.742	DII	H-bond/polar candidate
**PZ2**	PRTGLDFSDLYYLTMNNKHWLVHKE	A	GLU274	OE1	THR14	OG1	3.299	DII	H-bond/polar candidate
**PZ2**	PRTGLDFSDLYYLTMNNKHWLVHKE	B	LYS246	CD	THR14	OG1	3.246	DII	H-bond/polar candidate
**PZ2**	PRTGLDFSDLYYLTMNNKHWLVHKE	B	LYS246	CE	THR14	OG1	3.202	DII	H-bond/polar candidate
**PZ2**	PRTGLDFSDLYYLTMNNKHWLVHKE	B	LYS246	NZ	THR14	OG1	2.832	DII	H-bond/polar candidate
**PZ2**	PRTGLDFSDLYYLTMNNKHWLVHKE	B	LYS246	HZ1	THR14	OG1	3.604	DII	H-bond/polar candidate
**PZ2**	PRTGLDFSDLYYLTMNNKHWLVHKE	B	LYS246	HZ2	THR14	OG1	3.249	DII	H-bond/polar candidate
**PZ2**	PRTGLDFSDLYYLTMNNKHWLVHKE	B	LYS246	HZ3	THR14	OG1	1.888	DII	H-bond/polar candidate
**PZ2**	PRTGLDFSDLYYLTMNNKHWLVHKE	B	ASP247	CG	THR14	OG1	3.184	DII	H-bond/polar candidate
**PZ2**	PRTGLDFSDLYYLTMNNKHWLVHKE	B	ASP247	OD1	THR14	OG1	2.828	DII	H-bond/polar candidate
**PZ2**	PRTGLDFSDLYYLTMNNKHWLVHKE	B	ASP247	OD2	THR14	OG1	2.778	DII	H-bond/polar candidate
**PZ2**	PRTGLDFSDLYYLTMNNKHWLVHKE	B	LYS246	CD	THR14	HG1	3.356	DII	H-bond/polar candidate
**PZ2**	PRTGLDFSDLYYLTMNNKHWLVHKE	B	LYS246	CE	THR14	HG1	3.712	DII	H-bond/polar candidate

**PZ2**	PRTGLDFSDLYYLTMNNKHWLVHKE	B	LYS246	NZ	THR14	HG1	3.516	DII	H-bond/polar candidate
**PZ2**	PRTGLDFSDLYYLTMNNKHWLVHKE	B	LYS246	HZ3	THR14	HG1	2.589	DII	H-bond/polar candidate
**PZ2**	PRTGLDFSDLYYLTMNNKHWLVHKE	B	ASP247	CB	THR14	HG1	3.733	DII	H-bond/polar candidate
**PZ2**	PRTGLDFSDLYYLTMNNKHWLVHKE	B	ASP247	CG	THR14	HG1	2.222	DII	H-bond/polar candidate
**PZ2**	PRTGLDFSDLYYLTMNNKHWLVHKE	B	ASP247	OD1	THR14	HG1	1.934	DII	H-bond/polar candidate
**PZ2**	PRTGLDFSDLYYLTMNNKHWLVHKE	B	ASP247	OD2	THR14	HG1	1.964	DII	H-bond/polar candidate
**PZ2**	PRTGLDFSDLYYLTMNNKHWLVHKE	A	GLU274	OE1	THR14	CG2	3.578	DII	H-bond/polar candidate
**PZ2**	PRTGLDFSDLYYLTMNNKHWLVHKE	B	LYS246	HZ3	THR14	CG2	3.042	DII	H-bond/polar candidate
**PZ2**	PRTGLDFSDLYYLTMNNKHWLVHKE	B	ASP247	OD1	THR14	CG2	3.399	DII	H-bond/polar candidate
**PZ2**	PRTGLDFSDLYYLTMNNKHWLVHKE	A	GLU274	H	THR14	C	3.895	DII	H-bond/polar candidate
**PZ2**	PRTGLDFSDLYYLTMNNKHWLVHKE	A	SER285	OG	THR14	C	3.957	DII	H-bond/polar candidate
**PZ2**	PRTGLDFSDLYYLTMNNKHWLVHKE	A	SER285	HG	THR14	C	3.165	DII	H-bond/polar candidate
**PZ2**	PRTGLDFSDLYYLTMNNKHWLVHKE	A	GLU274	H	THR14	O	3.496	DII	H-bond/polar candidate
**PZ2**	PRTGLDFSDLYYLTMNNKHWLVHKE	A	GLU274	CA	THR14	O	3.941	DII	H-bond/polar candidate
**PZ2**	PRTGLDFSDLYYLTMNNKHWLVHKE	A	GLU274	CB	THR14	O	3.408	DII	H-bond/polar candidate
**PZ2**	PRTGLDFSDLYYLTMNNKHWLVHKE	A	GLU274	C	THR14	O	3.898	DII	H-bond/polar candidate
**PZ2**	PRTGLDFSDLYYLTMNNKHWLVHKE	A	GLU274	O	THR14	O	3.83	DII	H-bond/polar candidate
**PZ2**	PRTGLDFSDLYYLTMNNKHWLVHKE	A	SER285	CB	THR14	O	3.785	DII	H-bond/polar candidate
**PZ2**	PRTGLDFSDLYYLTMNNKHWLVHKE	A	SER285	OG	THR14	O	3.084	DII	H-bond/polar candidate
**PZ2**	PRTGLDFSDLYYLTMNNKHWLVHKE	A	SER285	HG	THR14	O	2.183	DII	H-bond/polar candidate
**PZ2**	PRTGLDFSDLYYLTMNNKHWLVHKE	A	GLU274	H	MET15	N	3.891	DII	H-bond/polar candidate
**PZ2**	PRTGLDFSDLYYLTMNNKHWLVHKE	A	SER285	HG	MET15	N	3.682	DII	H-bond/polar candidate
**PZ2**	PRTGLDFSDLYYLTMNNKHWLVHKE	A	GLU274	N	MET15	CA	3.91	DII	H-bond/polar candidate
**PZ2**	PRTGLDFSDLYYLTMNNKHWLVHKE	A	GLU274	H	MET15	CA	2.924	DII	H-bond/polar candidate
**PZ2**	PRTGLDFSDLYYLTMNNKHWLVHKE	A	SER285	HG	MET15	CA	3.671	DII	H-bond/polar candidate
**PZ2**	PRTGLDFSDLYYLTMNNKHWLVHKE	A	GLU274	H	MET15	CB	3.452	DII	H-bond/polar candidate
**PZ2**	PRTGLDFSDLYYLTMNNKHWLVHKE	A	GLU274	O	MET15	CB	3.837	DII	H-bond/polar candidate
**PZ2**	PRTGLDFSDLYYLTMNNKHWLVHKE	A	SER285	CB	MET15	CB	3.981	DII	H-bond/polar candidate
**PZ2**	PRTGLDFSDLYYLTMNNKHWLVHKE	A	SER285	OG	MET15	CB	3.137	DII	H-bond/polar candidate
**PZ2**	PRTGLDFSDLYYLTMNNKHWLVHKE	A	SER285	HG	MET15	CB	3.079	DII	H-bond/polar candidate

**PZ2**	PRTGLDFSDLYYLTMNNKHWLVHKE	A	SER286	N	MET15	CB	3.843	DI	H-bond/polar candidate
**PZ2**	PRTGLDFSDLYYLTMNNKHWLVHKE	A	SER286	H	MET15	CB	3.536	DI	H-bond/polar candidate
**PZ2**	PRTGLDFSDLYYLTMNNKHWLVHKE	A	SER286	CB	MET15	CB	3.802	DI	H-bond/polar candidate
**PZ2**	PRTGLDFSDLYYLTMNNKHWLVHKE	A	LEU273	CA	MET15	CG	3.871	DII	hydrophobic/van der Waals
**PZ2**	PRTGLDFSDLYYLTMNNKHWLVHKE	A	LEU273	CB	MET15	CG	3.492	DII	hydrophobic/van der Waals
**PZ2**	PRTGLDFSDLYYLTMNNKHWLVHKE	A	LEU273	CG	MET15	CG	3.945	DII	hydrophobic/van der Waals
**PZ2**	PRTGLDFSDLYYLTMNNKHWLVHKE	A	LEU273	CD1	MET15	CG	3.817	DII	hydrophobic/van der Waals
**PZ2**	PRTGLDFSDLYYLTMNNKHWLVHKE	A	GLU274	N	MET15	CG	3.892	DII	H-bond/polar candidate
**PZ2**	PRTGLDFSDLYYLTMNNKHWLVHKE	A	GLU274	H	MET15	CG	3.217	DII	H-bond/polar candidate
**PZ2**	PRTGLDFSDLYYLTMNNKHWLVHKE	A	GLU274	O	MET15	CG	3.298	DII	H-bond/polar candidate
**PZ2**	PRTGLDFSDLYYLTMNNKHWLVHKE	A	SER285	OG	MET15	CG	3.557	DII	H-bond/polar candidate
**PZ2**	PRTGLDFSDLYYLTMNNKHWLVHKE	A	SER285	HG	MET15	CG	3.707	DII	H-bond/polar candidate
**PZ2**	PRTGLDFSDLYYLTMNNKHWLVHKE	A	SER286	N	MET15	CG	3.927	DI	H-bond/polar candidate
**PZ2**	PRTGLDFSDLYYLTMNNKHWLVHKE	A	SER286	H	MET15	CG	3.316	DI	H-bond/polar candidate
**PZ2**	PRTGLDFSDLYYLTMNNKHWLVHKE	A	SER286	CB	MET15	CG	3.59	DI	H-bond/polar candidate
**PZ2**	PRTGLDFSDLYYLTMNNKHWLVHKE	A	GLU274	O	MET15	SD	3.024	DII	H-bond/polar candidate
**PZ2**	PRTGLDFSDLYYLTMNNKHWLVHKE	A	SER285	N	MET15	SD	3.542	DII	H-bond/polar candidate
**PZ2**	PRTGLDFSDLYYLTMNNKHWLVHKE	A	SER285	H	MET15	SD	2.711	DII	H-bond/polar candidate
**PZ2**	PRTGLDFSDLYYLTMNNKHWLVHKE	A	SER285	CA	MET15	SD	3.986	DII	H-bond/polar candidate
**PZ2**	PRTGLDFSDLYYLTMNNKHWLVHKE	A	SER285	OG	MET15	SD	2.988	DII	H-bond/polar candidate
**PZ2**	PRTGLDFSDLYYLTMNNKHWLVHKE	A	SER285	HG	MET15	SD	3.537	DII	H-bond/polar candidate
**PZ2**	PRTGLDFSDLYYLTMNNKHWLVHKE	A	SER285	C	MET15	SD	3.962	DII	H-bond/polar candidate
**PZ2**	PRTGLDFSDLYYLTMNNKHWLVHKE	A	SER286	N	MET15	SD	3.049	DI	H-bond/polar candidate
**PZ2**	PRTGLDFSDLYYLTMNNKHWLVHKE	A	SER286	H	MET15	SD	2.139	DI	H-bond/polar candidate
**PZ2**	PRTGLDFSDLYYLTMNNKHWLVHKE	A	SER286	CA	MET15	SD	3.849	DI	H-bond/polar candidate
**PZ2**	PRTGLDFSDLYYLTMNNKHWLVHKE	A	SER286	CB	MET15	SD	3.486	DI	H-bond/polar candidate
**PZ2**	PRTGLDFSDLYYLTMNNKHWLVHKE	A	LEU212	CD1	MET15	CE	3.899	DII	hydrophobic/van der Waals
**PZ2**	PRTGLDFSDLYYLTMNNKHWLVHKE	A	LEU273	CB	MET15	CE	3.623	DII	hydrophobic/van der Waals
**PZ2**	PRTGLDFSDLYYLTMNNKHWLVHKE	A	LEU273	CG	MET15	CE	3.726	DII	hydrophobic/van der Waals
**PZ2**	PRTGLDFSDLYYLTMNNKHWLVHKE	A	SER285	H	MET15	CE	3.971	DII	H-bond/polar candidate

**PZ2**	PRTGLDFSDLYYLTMNNKHWLVHKE	A	SER286	H	MET15	CE	3.203	DI	H-bond/polar candidate
**PZ2**	PRTGLDFSDLYYLTMNNKHWLVHKE	A	SER286	CB	MET15	CE	3.728	DI	H-bond/polar candidate
**PZ2**	PRTGLDFSDLYYLTMNNKHWLVHKE	A	GLU274	H	MET15	C	3.575	DII	H-bond/polar candidate
**PZ2**	PRTGLDFSDLYYLTMNNKHWLVHKE	A	GLU274	H	ASN16	N	3.469	DII	H-bond/polar candidate
**PZ2**	PRTGLDFSDLYYLTMNNKHWLVHKE	A	ALA272	O	ASN16	H	3.814	DII	H-bond/polar candidate
**PZ2**	PRTGLDFSDLYYLTMNNKHWLVHKE	A	GLU274	N	ASN16	H	3.69	DII	H-bond/polar candidate
**PZ2**	PRTGLDFSDLYYLTMNNKHWLVHKE	A	GLU274	H	ASN16	H	2.889	DII	H-bond/polar candidate
**PZ2**	PRTGLDFSDLYYLTMNNKHWLVHKE	A	GLU274	OE1	ASN16	H	3.834	DII	H-bond/polar candidate
**PZ2**	PRTGLDFSDLYYLTMNNKHWLVHKE	A	GLY271	O	ASN16	CA	3.977	DII	H-bond/polar candidate
**PZ2**	PRTGLDFSDLYYLTMNNKHWLVHKE	A	GLY271	O	ASN16	CB	3.374	DII	H-bond/polar candidate
**PZ2**	PRTGLDFSDLYYLTMNNKHWLVHKE	A	LEU273	CD1	ASN16	CB	3.918	DII	H-bond/polar candidate
**PZ2**	PRTGLDFSDLYYLTMNNKHWLVHKE	A	GLY271	O	ASN16	CG	3.423	DII	H-bond/polar candidate
**PZ2**	PRTGLDFSDLYYLTMNNKHWLVHKE	A	GLY271	CA	ASN16	ND2	3.589	DII	H-bond/polar candidate
**PZ2**	PRTGLDFSDLYYLTMNNKHWLVHKE	A	GLY271	C	ASN16	ND2	3.493	DII	H-bond/polar candidate
**PZ2**	PRTGLDFSDLYYLTMNNKHWLVHKE	A	GLY271	O	ASN16	ND2	2.766	DII	H-bond/polar candidate
**PZ2**	PRTGLDFSDLYYLTMNNKHWLVHKE	A	ALA270	O	ASN16	HD21	3.366	DII	H-bond/polar candidate
**PZ2**	PRTGLDFSDLYYLTMNNKHWLVHKE	A	GLY271	CA	ASN16	HD21	3.172	DII	H-bond/polar candidate
**PZ2**	PRTGLDFSDLYYLTMNNKHWLVHKE	A	GLY271	C	ASN16	HD21	2.854	DII	H-bond/polar candidate
**PZ2**	PRTGLDFSDLYYLTMNNKHWLVHKE	A	GLY271	O	ASN16	HD21	1.964	DII	H-bond/polar candidate
**PZ2**	PRTGLDFSDLYYLTMNNKHWLVHKE	A	GLY271	CA	ASN16	HD22	3.619	DII	H-bond/polar candidate
**PZ2**	PRTGLDFSDLYYLTMNNKHWLVHKE	A	GLY271	C	ASN16	HD22	3.925	DII	H-bond/polar candidate
**PZ2**	PRTGLDFSDLYYLTMNNKHWLVHKE	A	GLY271	O	ASN16	HD22	3.422	DII	H-bond/polar candidate
**PZ2**	PRTGLDFSDLYYLTMNNKHWLVHKE	A	GLU26	OE1	LYS18	CD	3.595	DI	salt bridge candidate; H-bond/polar candidate
**PZ2**	PRTGLDFSDLYYLTMNNKHWLVHKE	A	ASN8	OD1	LYS18	CE	3.731	DI	H-bond/polar candidate
**PZ2**	PRTGLDFSDLYYLTMNNKHWLVHKE	A	GLU26	OE1	LYS18	CE	3.6	DI	salt bridge candidate; H-bond/polar candidate
**PZ2**	PRTGLDFSDLYYLTMNNKHWLVHKE	A	GLU26	OE2	LYS18	CE	3.858	DI	salt bridge candidate; H-bond/polar candidate
**PZ2**	PRTGLDFSDLYYLTMNNKHWLVHKE	A	ASN8	CG	LYS18	NZ	3.572	DI	H-bond/polar candidate
**PZ2**	PRTGLDFSDLYYLTMNNKHWLVHKE	A	ASN8	OD1	LYS18	NZ	2.685	DI	H-bond/polar candidate
**PZ2**	PRTGLDFSDLYYLTMNNKHWLVHKE	A	GLU26	CD	LYS18	NZ	2.903	DI	salt bridge candidate; H-bond/polar candidate
**PZ2**	PRTGLDFSDLYYLTMNNKHWLVHKE	A	GLU26	OE1	LYS18	NZ	2.532	DI	salt bridge candidate; H-bond/polar candidate

**PZ2**	PRTGLDFSDLYYLTMNNKHWLVHKE	A	GLU26	OE2	LYS18	NZ	2.547	DI	salt bridge candidate; H-bond/polar candidate
**PZ2**	PRTGLDFSDLYYLTMNNKHWLVHKE	A	ASN8	CB	LYS18	HZ1	3.618	DI	H-bond/polar candidate
**PZ2**	PRTGLDFSDLYYLTMNNKHWLVHKE	A	ASN8	CG	LYS18	HZ1	2.703	DI	H-bond/polar candidate
**PZ2**	PRTGLDFSDLYYLTMNNKHWLVHKE	A	ASN8	OD1	LYS18	HZ1	1.688	DI	H-bond/polar candidate
**PZ2**	PRTGLDFSDLYYLTMNNKHWLVHKE	A	ASN8	ND2	LYS18	HZ1	3.551	DI	H-bond/polar candidate
**PZ2**	PRTGLDFSDLYYLTMNNKHWLVHKE	A	ASN8	HD22	LYS18	HZ1	3.499	DI	H-bond/polar candidate
**PZ2**	PRTGLDFSDLYYLTMNNKHWLVHKE	A	GLU26	CD	LYS18	HZ1	3.299	DI	salt bridge candidate; H-bond/polar candidate
**PZ2**	PRTGLDFSDLYYLTMNNKHWLVHKE	A	GLU26	OE1	LYS18	HZ1	3.147	DI	salt bridge candidate; H-bond/polar candidate
**PZ2**	PRTGLDFSDLYYLTMNNKHWLVHKE	A	GLU26	OE2	LYS18	HZ1	2.664	DI	salt bridge candidate; H-bond/polar candidate
**PZ2**	PRTGLDFSDLYYLTMNNKHWLVHKE	A	ASN8	OD1	LYS18	HZ2	3.166	DI	H-bond/polar candidate
**PZ2**	PRTGLDFSDLYYLTMNNKHWLVHKE	A	GLU26	CG	LYS18	HZ2	3.999	DI	salt bridge candidate; H-bond/polar candidate
**PZ2**	PRTGLDFSDLYYLTMNNKHWLVHKE	A	GLU26	CD	LYS18	HZ2	2.533	DI	salt bridge candidate; H-bond/polar candidate
**PZ2**	PRTGLDFSDLYYLTMNNKHWLVHKE	A	GLU26	OE1	LYS18	HZ2	1.817	DI	salt bridge candidate; H-bond/polar candidate
**PZ2**	PRTGLDFSDLYYLTMNNKHWLVHKE	A	GLU26	OE2	LYS18	HZ2	2.595	DI	salt bridge candidate; H-bond/polar candidate
**PZ2**	PRTGLDFSDLYYLTMNNKHWLVHKE	A	ASN8	CG	LYS18	HZ3	3.484	DI	H-bond/polar candidate
**PZ2**	PRTGLDFSDLYYLTMNNKHWLVHKE	A	ASN8	OD1	LYS18	HZ3	2.923	DI	H-bond/polar candidate
**PZ2**	PRTGLDFSDLYYLTMNNKHWLVHKE	A	ASN8	HD22	LYS18	HZ3	3.866	DI	H-bond/polar candidate
**PZ2**	PRTGLDFSDLYYLTMNNKHWLVHKE	A	GLU26	CG	LYS18	HZ3	3.668	DI	salt bridge candidate; H-bond/polar candidate
**PZ2**	PRTGLDFSDLYYLTMNNKHWLVHKE	A	GLU26	CD	LYS18	HZ3	2.251	DI	salt bridge candidate; H-bond/polar candidate
**PZ2**	PRTGLDFSDLYYLTMNNKHWLVHKE	A	GLU26	OE1	LYS18	HZ3	2.245	DI	salt bridge candidate; H-bond/polar candidate
**PZ2**	PRTGLDFSDLYYLTMNNKHWLVHKE	A	GLU26	OE2	LYS18	HZ3	1.774	DI	salt bridge candidate; H-bond/polar candidate
**PZ2**	PRTGLDFSDLYYLTMNNKHWLVHKE	B	ASP247	CB	HIS19	CB	3.946	DII	salt bridge candidate; H-bond/polar candidate
**PZ2**	PRTGLDFSDLYYLTMNNKHWLVHKE	B	ASP247	CA	HIS19	CG	4.0	DII	salt bridge candidate; H-bond/polar candidate
**PZ2**	PRTGLDFSDLYYLTMNNKHWLVHKE	B	ASP247	CB	HIS19	CG	3.832	DII	salt bridge candidate; H-bond/polar candidate
**PZ2**	PRTGLDFSDLYYLTMNNKHWLVHKE	B	ASP247	OD1	HIS19	CG	3.84	DII	salt bridge candidate; H-bond/polar candidate
**PZ2**	PRTGLDFSDLYYLTMNNKHWLVHKE	B	ASP247	CA	HIS19	ND1	3.578	DII	salt bridge candidate; H-bond/polar candidate
**PZ2**	PRTGLDFSDLYYLTMNNKHWLVHKE	B	ASP247	CB	HIS19	ND1	3.468	DII	salt bridge candidate; H-bond/polar candidate
**PZ2**	PRTGLDFSDLYYLTMNNKHWLVHKE	B	ASP247	CG	HIS19	ND1	3.513	DII	salt bridge candidate; H-bond/polar candidate
**PZ2**	PRTGLDFSDLYYLTMNNKHWLVHKE	B	ASP247	OD1	HIS19	ND1	2.79	DII	salt bridge candidate; H-bond/polar candidate
**PZ2**	PRTGLDFSDLYYLTMNNKHWLVHKE	B	ASP247	CA	HIS19	HD1	3.502	DII	salt bridge candidate; H-bond/polar candidate

**PZ2**	PRTGLDFSDLYYLTMNNKHWLVHKE	B	ASP247	CB	HIS19	HD1	3.092	DII	salt bridge candidate; H-bond/polar candidate
**PZ2**	PRTGLDFSDLYYLTMNNKHWLVHKE	B	ASP247	CG	HIS19	HD1	2.786	DII	salt bridge candidate; H-bond/polar candidate
**PZ2**	PRTGLDFSDLYYLTMNNKHWLVHKE	B	ASP247	OD1	HIS19	HD1	1.959	DII	salt bridge candidate; H-bond/polar candidate
**PZ2**	PRTGLDFSDLYYLTMNNKHWLVHKE	B	ASP247	OD2	HIS19	HD1	3.897	DII	salt bridge candidate; H-bond/polar candidate
**PZ2**	PRTGLDFSDLYYLTMNNKHWLVHKE	B	ASP247	CA	HIS19	CE1	3.884	DII	salt bridge candidate; H-bond/polar candidate
**PZ2**	PRTGLDFSDLYYLTMNNKHWLVHKE	B	ASP247	OD1	HIS19	CE1	3.498	DII	salt bridge candidate; H-bond/polar candidate
**PZ2**	PRTGLDFSDLYYLTMNNKHWLVHKE	A	SER285	O	TRP20	CB	3.476	DII	H-bond/polar candidate
**PZ2**	PRTGLDFSDLYYLTMNNKHWLVHKE	A	SER285	O	TRP20	CG	3.444	DII	H-bond/polar candidate
**PZ2**	PRTGLDFSDLYYLTMNNKHWLVHKE	A	SER285	O	TRP20	CD2	3.748	DII	H-bond/polar candidate
**PZ2**	PRTGLDFSDLYYLTMNNKHWLVHKE	A	SER286	CA	TRP20	CD2	3.835	DI	H-bond/polar candidate
**PZ2**	PRTGLDFSDLYYLTMNNKHWLVHKE	A	GLU26	OE1	TRP20	CE2	3.886	DI	H-bond/polar candidate
**PZ2**	PRTGLDFSDLYYLTMNNKHWLVHKE	A	SER286	CA	TRP20	CE2	3.938	DI	H-bond/polar candidate
**PZ2**	PRTGLDFSDLYYLTMNNKHWLVHKE	A	SER286	CA	TRP20	CE3	3.862	DI	H-bond/polar candidate
**PZ2**	PRTGLDFSDLYYLTMNNKHWLVHKE	A	GLU26	CD	TRP20	CD1	3.916	DI	H-bond/polar candidate
**PZ2**	PRTGLDFSDLYYLTMNNKHWLVHKE	A	GLU26	OE1	TRP20	CD1	3.96	DI	H-bond/polar candidate
**PZ2**	PRTGLDFSDLYYLTMNNKHWLVHKE	A	HIS27	H	TRP20	CD1	3.761	DI	H-bond/polar candidate
**PZ2**	PRTGLDFSDLYYLTMNNKHWLVHKE	A	HIS27	CB	TRP20	CD1	3.669	DI	H-bond/polar candidate
**PZ2**	PRTGLDFSDLYYLTMNNKHWLVHKE	A	HIS27	C	TRP20	CD1	3.71	DI	H-bond/polar candidate
**PZ2**	PRTGLDFSDLYYLTMNNKHWLVHKE	A	HIS27	O	TRP20	CD1	2.999	DI	H-bond/polar candidate
**PZ2**	PRTGLDFSDLYYLTMNNKHWLVHKE	A	SER285	O	TRP20	CD1	3.987	DII	H-bond/polar candidate
**PZ2**	PRTGLDFSDLYYLTMNNKHWLVHKE	A	GLY287	H	TRP20	CD1	3.898	DI	hydrophobic/van der Waals
**PZ2**	PRTGLDFSDLYYLTMNNKHWLVHKE	A	GLU26	CG	TRP20	NE1	3.618	DI	H-bond/polar candidate
**PZ2**	PRTGLDFSDLYYLTMNNKHWLVHKE	A	GLU26	CD	TRP20	NE1	3.268	DI	H-bond/polar candidate
**PZ2**	PRTGLDFSDLYYLTMNNKHWLVHKE	A	GLU26	OE1	TRP20	NE1	2.986	DI	H-bond/polar candidate
**PZ2**	PRTGLDFSDLYYLTMNNKHWLVHKE	A	GLU26	OE2	TRP20	NE1	3.869	DI	H-bond/polar candidate
**PZ2**	PRTGLDFSDLYYLTMNNKHWLVHKE	A	HIS27	H	TRP20	NE1	3.877	DI	H-bond/polar candidate
**PZ2**	PRTGLDFSDLYYLTMNNKHWLVHKE	A	HIS27	O	TRP20	NE1	3.835	DI	H-bond/polar candidate
**PZ2**	PRTGLDFSDLYYLTMNNKHWLVHKE	A	GLY287	H	TRP20	NE1	3.879	DI	hydrophobic/van der Waals
**PZ2**	PRTGLDFSDLYYLTMNNKHWLVHKE	A	GLU26	CG	TRP20	HE1	2.768	DI	H-bond/polar candidate
**PZ2**	PRTGLDFSDLYYLTMNNKHWLVHKE	A	GLU26	CD	TRP20	HE1	2.354	DI	H-bond/polar candidate

**PZ2**	PRTGLDFSDLYYLTMNNKHWLVHKE	A	GLU26	OE1	TRP20	HE1	2.058	DI	H-bond/polar candidate
**PZ2**	PRTGLDFSDLYYLTMNNKHWLVHKE	A	GLU26	OE2	TRP20	HE1	3.119	DI	H-bond/polar candidate
**PZ2**	PRTGLDFSDLYYLTMNNKHWLVHKE	A	HIS27	H	TRP20	HE1	3.647	DI	H-bond/polar candidate
**PZ2**	PRTGLDFSDLYYLTMNNKHWLVHKE	A	HIS27	O	TRP20	HE1	3.837	DI	H-bond/polar candidate
**PZ2**	PRTGLDFSDLYYLTMNNKHWLVHKE	A	SER286	CB	TRP20	CZ2	3.922	DI	H-bond/polar candidate
**PZ2**	PRTGLDFSDLYYLTMNNKHWLVHKE	A	SER286	OG	TRP20	CZ2	3.289	DI	H-bond/polar candidate
**PZ2**	PRTGLDFSDLYYLTMNNKHWLVHKE	A	SER286	HG	TRP20	CZ2	3.936	DI	H-bond/polar candidate
**PZ2**	PRTGLDFSDLYYLTMNNKHWLVHKE	A	SER286	CA	TRP20	CZ3	3.982	DI	H-bond/polar candidate
**PZ2**	PRTGLDFSDLYYLTMNNKHWLVHKE	A	SER286	CB	TRP20	CZ3	3.616	DI	H-bond/polar candidate
**PZ2**	PRTGLDFSDLYYLTMNNKHWLVHKE	A	SER286	OG	TRP20	CZ3	3.733	DI	H-bond/polar candidate
**PZ2**	PRTGLDFSDLYYLTMNNKHWLVHKE	A	SER286	CB	TRP20	CH2	3.554	DI	H-bond/polar candidate
**PZ2**	PRTGLDFSDLYYLTMNNKHWLVHKE	A	SER286	OG	TRP20	CH2	3.116	DI	H-bond/polar candidate
**PZ2**	PRTGLDFSDLYYLTMNNKHWLVHKE	A	SER286	HG	TRP20	CH2	3.976	DI	H-bond/polar candidate
**PZ2**	PRTGLDFSDLYYLTMNNKHWLVHKE	A	GLY28	CA	LEU21	N	3.698	DI	hydrophobic/van der Waals
**PZ2**	PRTGLDFSDLYYLTMNNKHWLVHKE	A	HIS27	O	LEU21	H	3.821	DI	H-bond/polar candidate
**PZ2**	PRTGLDFSDLYYLTMNNKHWLVHKE	A	GLY28	N	LEU21	H	3.748	DI	hydrophobic/van der Waals
**PZ2**	PRTGLDFSDLYYLTMNNKHWLVHKE	A	GLY28	CA	LEU21	H	3.098	DI	hydrophobic/van der Waals
**PZ2**	PRTGLDFSDLYYLTMNNKHWLVHKE	A	GLY28	CA	LEU21	CA	3.804	DI	hydrophobic/van der Waals
**PZ2**	PRTGLDFSDLYYLTMNNKHWLVHKE	A	GLY28	CA	LEU21	CB	3.522	DI	hydrophobic/van der Waals
**PZ2**	PRTGLDFSDLYYLTMNNKHWLVHKE	A	GLY28	N	LEU21	C	3.971	DI	hydrophobic/van der Waals
**PZ2**	PRTGLDFSDLYYLTMNNKHWLVHKE	A	GLY28	H	LEU21	C	3.94	DI	hydrophobic/van der Waals
**PZ2**	PRTGLDFSDLYYLTMNNKHWLVHKE	A	GLY28	CA	LEU21	C	3.675	DI	hydrophobic/van der Waals
**PZ2**	PRTGLDFSDLYYLTMNNKHWLVHKE	A	GLY28	N	LEU21	O	3.256	DI	hydrophobic/van der Waals
**PZ2**	PRTGLDFSDLYYLTMNNKHWLVHKE	A	GLY28	H	LEU21	O	3.049	DI	hydrophobic/van der Waals
**PZ2**	PRTGLDFSDLYYLTMNNKHWLVHKE	A	GLY28	CA	LEU21	O	3.012	DI	hydrophobic/van der Waals
**PZ2**	PRTGLDFSDLYYLTMNNKHWLVHKE	A	HIS27	HD1	VAL22	CB	3.818	DI	H-bond/polar candidate
**PZ2**	PRTGLDFSDLYYLTMNNKHWLVHKE	A	HIS27	ND1	VAL22	CG1	3.477	DI	H-bond/polar candidate
**PZ2**	PRTGLDFSDLYYLTMNNKHWLVHKE	A	HIS27	HD1	VAL22	CG1	2.781	DI	H-bond/polar candidate
**PZ2**	PRTGLDFSDLYYLTMNNKHWLVHKE	A	HIS27	CE1	VAL22	CG1	3.917	DI	H-bond/polar candidate
**PZ2**	PRTGLDFSDLYYLTMNNKHWLVHKE	A	GLU276	OE2	VAL22	CG1	3.63	DII	H-bond/polar candidate

**PZ2**	PRTGLDFSDLYYLTMNNKHWLVHKE	A	SER285	CA	VAL22	CG1	3.994	DII	H-bond/polar candidate
**PZ2**	PRTGLDFSDLYYLTMNNKHWLVHKE	A	SER285	CB	VAL22	CG1	3.888	DII	H-bond/polar candidate
**PZ2**	PRTGLDFSDLYYLTMNNKHWLVHKE	A	GLU276	OE1	VAL22	CG2	3.586	DII	H-bond/polar candidate
**PZ2**	PRTGLDFSDLYYLTMNNKHWLVHKE	A	GLU276	OE2	VAL22	CG2	3.871	DII	H-bond/polar candidate
**PZ2**	PRTGLDFSDLYYLTMNNKHWLVHKE	A	ARG283	HE	VAL22	CG2	3.76	DII	H-bond/polar candidate
**PZ2**	PRTGLDFSDLYYLTMNNKHWLVHKE	A	VAL46	O	HIS23	N	3.12	DI	H-bond/polar candidate
**PZ2**	PRTGLDFSDLYYLTMNNKHWLVHKE	A	VAL46	C	HIS23	H	3.39	DI	H-bond/polar candidate
**PZ2**	PRTGLDFSDLYYLTMNNKHWLVHKE	A	VAL46	O	HIS23	H	2.215	DI	H-bond/polar candidate
**PZ2**	PRTGLDFSDLYYLTMNNKHWLVHKE	A	VAL46	O	HIS23	CA	3.709	DI	H-bond/polar candidate
**PZ2**	PRTGLDFSDLYYLTMNNKHWLVHKE	A	VAL46	CB	HIS23	CB	3.8	DI	H-bond/polar candidate
**PZ2**	PRTGLDFSDLYYLTMNNKHWLVHKE	A	VAL46	C	HIS23	CB	3.934	DI	H-bond/polar candidate
**PZ2**	PRTGLDFSDLYYLTMNNKHWLVHKE	A	VAL46	O	HIS23	CB	3.363	DI	H-bond/polar candidate
**PZ2**	PRTGLDFSDLYYLTMNNKHWLVHKE	A	ARG2	NH2	HIS23	CD2	3.701	DI	H-bond/polar candidate
**PZ2**	PRTGLDFSDLYYLTMNNKHWLVHKE	A	ARG2	HH21	HIS23	CD2	2.789	DI	H-bond/polar candidate
**PZ2**	PRTGLDFSDLYYLTMNNKHWLVHKE	A	VAL46	CG1	HIS23	CD2	3.733	DI	H-bond/polar candidate
**PZ2**	PRTGLDFSDLYYLTMNNKHWLVHKE	A	ARG2	CZ	HIS23	NE2	3.696	DI	H-bond/polar candidate
**PZ2**	PRTGLDFSDLYYLTMNNKHWLVHKE	A	ARG2	NH1	HIS23	NE2	3.668	DI	H-bond/polar candidate
**PZ2**	PRTGLDFSDLYYLTMNNKHWLVHKE	A	ARG2	HH11	HIS23	NE2	2.968	DI	H-bond/polar candidate
**PZ2**	PRTGLDFSDLYYLTMNNKHWLVHKE	A	ARG2	NH2	HIS23	NE2	2.88	DI	H-bond/polar candidate
**PZ2**	PRTGLDFSDLYYLTMNNKHWLVHKE	A	ARG2	HH21	HIS23	NE2	1.929	DI	H-bond/polar candidate
**PZ2**	PRTGLDFSDLYYLTMNNKHWLVHKE	A	ARG2	HH22	HIS23	NE2	3.549	DI	H-bond/polar candidate
**PZ2**	PRTGLDFSDLYYLTMNNKHWLVHKE	A	ARG2	NH1	HIS23	CE1	3.984	DI	H-bond/polar candidate
**PZ2**	PRTGLDFSDLYYLTMNNKHWLVHKE	A	ARG2	HH11	HIS23	CE1	3.101	DI	H-bond/polar candidate
**PZ2**	PRTGLDFSDLYYLTMNNKHWLVHKE	A	ARG2	NH2	HIS23	CE1	3.912	DI	H-bond/polar candidate
**PZ2**	PRTGLDFSDLYYLTMNNKHWLVHKE	A	ARG2	HH21	HIS23	CE1	3.063	DI	H-bond/polar candidate
**PZ2**	PRTGLDFSDLYYLTMNNKHWLVHKE	A	THR47	CG2	HIS23	C	3.995	DI	H-bond/polar candidate
**PZ2**	PRTGLDFSDLYYLTMNNKHWLVHKE	A	VAL46	O	HIS23	O	3.815	DI	H-bond/polar candidate
**PZ2**	PRTGLDFSDLYYLTMNNKHWLVHKE	A	THR47	CG2	HIS23	O	3.183	DI	H-bond/polar candidate
**PZ2**	PRTGLDFSDLYYLTMNNKHWLVHKE	A	ARG283	CD	HIS23	O	3.846	DII	H-bond/polar candidate
**PZ2**	PRTGLDFSDLYYLTMNNKHWLVHKE	A	THR47	CG2	LYS24	C	3.775	DI	H-bond/polar candidate

**PZ2**	PRTGLDFSDLYYLTMNNKHWLVHKE	A	ARG283	HH12	LYS24	C	3.966	DII	H-bond/polar candidate
**PZ2**	PRTGLDFSDLYYLTMNNKHWLVHKE	A	THR47	CG2	LYS24	O	3.388	DI	H-bond/polar candidate
**PZ2**	PRTGLDFSDLYYLTMNNKHWLVHKE	A	ARG283	CD	LYS24	O	3.999	DII	H-bond/polar candidate
**PZ2**	PRTGLDFSDLYYLTMNNKHWLVHKE	A	ARG283	NH1	LYS24	O	3.654	DII	H-bond/polar candidate
**PZ2**	PRTGLDFSDLYYLTMNNKHWLVHKE	A	ARG283	HH12	LYS24	O	3.01	DII	H-bond/polar candidate
**PZ2**	PRTGLDFSDLYYLTMNNKHWLVHKE	A	THR47	OG1	GLU25	CA	3.439	DI	H-bond/polar candidate
**PZ2**	PRTGLDFSDLYYLTMNNKHWLVHKE	A	THR47	HG1	GLU25	CA	3.867	DI	H-bond/polar candidate
**PZ2**	PRTGLDFSDLYYLTMNNKHWLVHKE	A	THR47	CG2	GLU25	CA	3.809	DI	H-bond/polar candidate
**PZ2**	PRTGLDFSDLYYLTMNNKHWLVHKE	A	LYS166	HZ1	GLU25	CA	3.823	DI	salt bridge candidate; H-bond/polar candidate
**PZ2**	PRTGLDFSDLYYLTMNNKHWLVHKE	A	LYS166	HZ1	GLU25	CB	3.846	DI	salt bridge candidate; H-bond/polar candidate
**PZ2**	PRTGLDFSDLYYLTMNNKHWLVHKE	A	LYS166	HZ2	GLU25	CB	3.796	DI	salt bridge candidate; H-bond/polar candidate
**PZ2**	PRTGLDFSDLYYLTMNNKHWLVHKE	A	LYS166	HZ3	GLU25	CB	3.915	DI	salt bridge candidate; H-bond/polar candidate
**PZ2**	PRTGLDFSDLYYLTMNNKHWLVHKE	A	LYS166	HZ2	GLU25	CG	3.62	DI	salt bridge candidate; H-bond/polar candidate
**PZ2**	PRTGLDFSDLYYLTMNNKHWLVHKE	A	LYS166	HZ3	GLU25	CG	3.55	DI	salt bridge candidate; H-bond/polar candidate
**PZ2**	PRTGLDFSDLYYLTMNNKHWLVHKE	A	ARG138	CD	GLU25	CD	3.969	DI	salt bridge candidate; H-bond/polar candidate
**PZ2**	PRTGLDFSDLYYLTMNNKHWLVHKE	A	ARG138	HH12	GLU25	CD	3.422	DI	salt bridge candidate; H-bond/polar candidate
**PZ2**	PRTGLDFSDLYYLTMNNKHWLVHKE	A	LYS166	NZ	GLU25	CD	2.807	DI	salt bridge candidate; H-bond/polar candidate
**PZ2**	PRTGLDFSDLYYLTMNNKHWLVHKE	A	LYS166	HZ1	GLU25	CD	3.179	DI	salt bridge candidate; H-bond/polar candidate
**PZ2**	PRTGLDFSDLYYLTMNNKHWLVHKE	A	LYS166	HZ2	GLU25	CD	2.433	DI	salt bridge candidate; H-bond/polar candidate
**PZ2**	PRTGLDFSDLYYLTMNNKHWLVHKE	A	LYS166	HZ3	GLU25	CD	2.214	DI	salt bridge candidate; H-bond/polar candidate
**PZ2**	PRTGLDFSDLYYLTMNNKHWLVHKE	A	VAL46	CG2	GLU25	OE1	3.949	DI	H-bond/polar candidate
**PZ2**	PRTGLDFSDLYYLTMNNKHWLVHKE	A	THR47	OG1	GLU25	OE1	3.451	DI	H-bond/polar candidate
**PZ2**	PRTGLDFSDLYYLTMNNKHWLVHKE	A	THR47	HG1	GLU25	OE1	3.126	DI	H-bond/polar candidate
**PZ2**	PRTGLDFSDLYYLTMNNKHWLVHKE	A	ARG138	CB	GLU25	OE1	3.635	DI	salt bridge candidate; H-bond/polar candidate
**PZ2**	PRTGLDFSDLYYLTMNNKHWLVHKE	A	ARG138	CG	GLU25	OE1	3.945	DI	salt bridge candidate; H-bond/polar candidate
**PZ2**	PRTGLDFSDLYYLTMNNKHWLVHKE	A	ARG138	CD	GLU25	OE1	3.24	DI	salt bridge candidate; H-bond/polar candidate
**PZ2**	PRTGLDFSDLYYLTMNNKHWLVHKE	A	ARG138	HH12	GLU25	OE1	3.085	DI	salt bridge candidate; H-bond/polar candidate
**PZ2**	PRTGLDFSDLYYLTMNNKHWLVHKE	A	LYS166	CD	GLU25	OE1	3.849	DI	salt bridge candidate; H-bond/polar candidate
**PZ2**	PRTGLDFSDLYYLTMNNKHWLVHKE	A	LYS166	CE	GLU25	OE1	3.78	DI	salt bridge candidate; H-bond/polar candidate
**PZ2**	PRTGLDFSDLYYLTMNNKHWLVHKE	A	LYS166	NZ	GLU25	OE1	2.688	DI	salt bridge candidate; H-bond/polar candidate

**PZ2**	PRTGLDFSDLYYLTMNNKHWLVHKE	A	LYS166	HZ1	GLU25	OE1	3.088		DI	salt bridge candidate; H-bond/polar candidate
**PZ2**	PRTGLDFSDLYYLTMNNKHWLVHKE	A	LYS166	HZ2	GLU25	OE1	2.761		DI	salt bridge candidate; H-bond/polar candidate
**PZ2**	PRTGLDFSDLYYLTMNNKHWLVHKE	A	LYS166	HZ3	GLU25	OE1	1.75		DI	salt bridge candidate; H-bond/polar candidate
**PZ2**	PRTGLDFSDLYYLTMNNKHWLVHKE	A	ARG138	HH12	GLU25	OE2	3.824		DI	salt bridge candidate; H-bond/polar candidate
**PZ2**	PRTGLDFSDLYYLTMNNKHWLVHKE	A	LYS166	CD	GLU25	OE2	3.618		DI	salt bridge candidate; H-bond/polar candidate
**PZ2**	PRTGLDFSDLYYLTMNNKHWLVHKE	A	LYS166	CE	GLU25	OE2	3.62		DI	salt bridge candidate; H-bond/polar candidate
**PZ2**	PRTGLDFSDLYYLTMNNKHWLVHKE	A	LYS166	NZ	GLU25	OE2	2.536		DI	salt bridge candidate; H-bond/polar candidate
**PZ2**	PRTGLDFSDLYYLTMNNKHWLVHKE	A	LYS166	HZ1	GLU25	OE2	3.123		DI	salt bridge candidate; H-bond/polar candidate
**PZ2**	PRTGLDFSDLYYLTMNNKHWLVHKE	A	LYS166	HZ2	GLU25	OE2	1.785		DI	salt bridge candidate; H-bond/polar candidate
**PZ2**	PRTGLDFSDLYYLTMNNKHWLVHKE	A	LYS166	HZ3	GLU25	OE2	2.312		DI	salt bridge candidate; H-bond/polar candidate
**PZ2**	PRTGLDFSDLYYLTMNNKHWLVHKE	A	VAL46	CG2	GLU25	C	3.72		DI	H-bond/polar candidate
**PZ2**	PRTGLDFSDLYYLTMNNKHWLVHKE	A	THR47	CB	GLU25	C	3.867		DI	H-bond/polar candidate
**PZ2**	PRTGLDFSDLYYLTMNNKHWLVHKE	A	THR47	OG1	GLU25	C	2.793		DI	H-bond/polar candidate
**PZ2**	PRTGLDFSDLYYLTMNNKHWLVHKE	A	THR47	HG1	GLU25	C	3.318		DI	H-bond/polar candidate
**PZ2**	PRTGLDFSDLYYLTMNNKHWLVHKE	A	THR47	CG2	GLU25	C	3.801		DI	H-bond/polar candidate
**PZ2**	PRTGLDFSDLYYLTMNNKHWLVHKE	A	LYS166	NZ	GLU25	C	3.382		DI	salt bridge candidate; H-bond/polar candidate
**PZ2**	PRTGLDFSDLYYLTMNNKHWLVHKE	A	LYS166	HZ1	GLU25	C	2.765		DI	salt bridge candidate; H-bond/polar candidate
**PZ2**	PRTGLDFSDLYYLTMNNKHWLVHKE	A	LYS166	HZ2	GLU25	C	3.729		DI	salt bridge candidate; H-bond/polar candidate
**PZ2**	PRTGLDFSDLYYLTMNNKHWLVHKE	A	LYS166	HZ3	GLU25	C	3.053		DI	salt bridge candidate; H-bond/polar candidate
**PZ2**	PRTGLDFSDLYYLTMNNKHWLVHKE	A	VAL46	CB	GLU25	O	3.693		DI	H-bond/polar candidate
**PZ2**	PRTGLDFSDLYYLTMNNKHWLVHKE	A	VAL46	CG2	GLU25	O	3.473		DI	H-bond/polar candidate
**PZ2**	PRTGLDFSDLYYLTMNNKHWLVHKE	A	THR47	OG1	GLU25	O	3.761		DI	H-bond/polar candidate
**PZ2**	PRTGLDFSDLYYLTMNNKHWLVHKE	A	LYS166	CE	GLU25	O	3.757		DI	salt bridge candidate; H-bond/polar candidate
**PZ2**	PRTGLDFSDLYYLTMNNKHWLVHKE	A	LYS166	NZ	GLU25	O	2.563		DI	salt bridge candidate; H-bond/polar candidate
**PZ2**	PRTGLDFSDLYYLTMNNKHWLVHKE	A	LYS166	HZ1	GLU25	O	1.716		DI	salt bridge candidate; H-bond/polar candidate
**PZ2**	PRTGLDFSDLYYLTMNNKHWLVHKE	A	LYS166	HZ2	GLU25	O	3.021		DI	salt bridge candidate; H-bond/polar candidate
**PZ2**	PRTGLDFSDLYYLTMNNKHWLVHKE	A	LYS166	HZ3	GLU25	O	2.589		DI	salt bridge candidate; H-bond/polar candidate
**PZ3**	VEGMSGGTWVDVVLEHGGCVTVMAQ	B	THR309	CG2	MET4	CB	3.344		DIII	H-bond/polar candidate
**PZ3**	VEGMSGGTWVDVVLEHGGCVTVMAQ	B	LEU307	CB	MET4	SD	3.491		DIII	hydrophobic/van der Waals
**PZ3**	VEGMSGGTWVDVVLEHGGCVTVMAQ	B	LEU307	CD1	MET4	SD	3.724		DIII	hydrophobic/van der Waals

**PZ3**	VEGMSGGTWVDVVLEHGGCVTVMAQ	B	CYS308	O	MET4	CE	3.875	DIII	H-bond/polar candidate
**PZ3**	VEGMSGGTWVDVVLEHGGCVTVMAQ	B	THR309	CA	MET4	CE	3.84	DIII	H-bond/polar candidate
**PZ3**	VEGMSGGTWVDVVLEHGGCVTVMAQ	B	THR309	CG2	MET4	CE	3.687	DIII	H-bond/polar candidate
**PZ3**	VEGMSGGTWVDVVLEHGGCVTVMAQ	B	VAL391	CB	MET4	CE	3.972	DIII	hydrophobic/van der Waals
**PZ3**	VEGMSGGTWVDVVLEHGGCVTVMAQ	B	VAL391	CG2	MET4	CE	2.941	DIII	hydrophobic/van der Waals
**PZ3**	VEGMSGGTWVDVVLEHGGCVTVMAQ	B	VAL391	CG1	THR8	CG2	3.92	DIII	H-bond/polar candidate
**PZ3**	VEGMSGGTWVDVVLEHGGCVTVMAQ	B	LEU307	CG	VAL10	CB	3.711	DIII	hydrophobic/van der Waals
**PZ3**	VEGMSGGTWVDVVLEHGGCVTVMAQ	B	LEU307	CD1	VAL10	CB	2.996	DIII	hydrophobic/van der Waals
**PZ3**	VEGMSGGTWVDVVLEHGGCVTVMAQ	B	LEU307	CD2	VAL10	CB	3.697	DIII	hydrophobic/van der Waals
**PZ3**	VEGMSGGTWVDVVLEHGGCVTVMAQ	B	LEU307	CD1	VAL10	CG1	3.962	DIII	hydrophobic/van der Waals
**PZ3**	VEGMSGGTWVDVVLEHGGCVTVMAQ	B	LEU307	CD2	VAL10	CG1	3.833	DIII	hydrophobic/van der Waals
**PZ3**	VEGMSGGTWVDVVLEHGGCVTVMAQ	B	LEU307	CG	VAL10	CG2	3.098	DIII	hydrophobic/van der Waals
**PZ3**	VEGMSGGTWVDVVLEHGGCVTVMAQ	B	LEU307	CD1	VAL10	CG2	1.925	DIII	hydrophobic/van der Waals
**PZ3**	VEGMSGGTWVDVVLEHGGCVTVMAQ	B	LEU307	CD2	VAL10	CG2	3.354	DIII	hydrophobic/van der Waals
**PZ3**	VEGMSGGTWVDVVLEHGGCVTVMAQ	B	LYS340	NZ	VAL12	CG2	3.452	DIII	H-bond/polar candidate
**PZ3**	VEGMSGGTWVDVVLEHGGCVTVMAQ	B	GLN36	OE1	LEU14	CB	3.925	DI	H-bond/polar candidate
**PZ3**	VEGMSGGTWVDVVLEHGGCVTVMAQ	B	ASP37	OD1	LEU14	CG	3.351	DI	H-bond/polar candidate
**PZ3**	VEGMSGGTWVDVVLEHGGCVTVMAQ	B	GLN36	CB	LEU14	CD1	3.714	DI	H-bond/polar candidate
**PZ3**	VEGMSGGTWVDVVLEHGGCVTVMAQ	B	GLN36	CG	LEU14	CD1	3.75	DI	H-bond/polar candidate
**PZ3**	VEGMSGGTWVDVVLEHGGCVTVMAQ	B	GLN36	CD	LEU14	CD1	3.925	DI	H-bond/polar candidate
**PZ3**	VEGMSGGTWVDVVLEHGGCVTVMAQ	B	GLN36	OE1	LEU14	CD1	3.563	DI	H-bond/polar candidate
**PZ3**	VEGMSGGTWVDVVLEHGGCVTVMAQ	B	ASP37	N	LEU14	CD1	3.718	DI	H-bond/polar candidate
**PZ3**	VEGMSGGTWVDVVLEHGGCVTVMAQ	B	ASP37	CG	LEU14	CD1	2.856	DI	H-bond/polar candidate
**PZ3**	VEGMSGGTWVDVVLEHGGCVTVMAQ	B	ASP37	OD1	LEU14	CD1	2.373	DI	H-bond/polar candidate
**PZ3**	VEGMSGGTWVDVVLEHGGCVTVMAQ	B	ASP37	OD2	LEU14	CD1	2.957	DI	H-bond/polar candidate
**PZ3**	VEGMSGGTWVDVVLEHGGCVTVMAQ	B	ASP37	N	LEU14	CD2	3.672	DI	H-bond/polar candidate
**PZ3**	VEGMSGGTWVDVVLEHGGCVTVMAQ	B	ASP37	OD1	LEU14	CD2	3.466	DI	H-bond/polar candidate
**PZ3**	VEGMSGGTWVDVVLEHGGCVTVMAQ	B	GLY356	O	GLU15	OE2	3.89	DIII	H-bond/polar candidate
**PZ3**	VEGMSGGTWVDVVLEHGGCVTVMAQ	B	GLU13	OE1	HIS16	C	3.767	DI	salt bridge candidate; H-bond/polar candidate
**PZ3**	VEGMSGGTWVDVVLEHGGCVTVMAQ	B	ARG357	NH1	HIS16	C	3.733	DIII	H-bond/polar candidate

**PZ3**	VEGMSGGTWVDVVLEHGGCVTVMAQ	B	GLU13	OE1	HIS16	CB	3.921	DI	salt bridge candidate; H-bond/polar candidate
**PZ3**	VEGMSGGTWVDVVLEHGGCVTVMAQ	B	GLU13	CG	HIS16	O	3.813	DI	salt bridge candidate; H-bond/polar candidate
**PZ3**	VEGMSGGTWVDVVLEHGGCVTVMAQ	B	GLU13	CD	HIS16	O	3.418	DI	salt bridge candidate; H-bond/polar candidate
**PZ3**	VEGMSGGTWVDVVLEHGGCVTVMAQ	B	GLU13	OE1	HIS16	O	2.985	DI	salt bridge candidate; H-bond/polar candidate
**PZ3**	VEGMSGGTWVDVVLEHGGCVTVMAQ	B	ARG357	CZ	HIS16	O	3.718	DIII	H-bond/polar candidate
**PZ3**	VEGMSGGTWVDVVLEHGGCVTVMAQ	B	ARG357	NH1	HIS16	O	2.586	DIII	H-bond/polar candidate
**PZ3**	VEGMSGGTWVDVVLEHGGCVTVMAQ	B	GLN36	N	GLY17	CA	3.677	DI	H-bond/polar candidate
**PZ3**	VEGMSGGTWVDVVLEHGGCVTVMAQ	B	MET34	C	GLY17	O	3.874	DI	hydrophobic/van der Waals
**PZ3**	VEGMSGGTWVDVVLEHGGCVTVMAQ	B	MET34	CB	GLY17	O	3.534	DI	hydrophobic/van der Waals
**PZ3**	VEGMSGGTWVDVVLEHGGCVTVMAQ	B	MET34	CE	GLY17	O	3.793	DI	hydrophobic/van der Waals
**PZ3**	VEGMSGGTWVDVVLEHGGCVTVMAQ	B	ALA35	N	GLY17	O	3.461	DI	hydrophobic/van der Waals
**PZ3**	VEGMSGGTWVDVVLEHGGCVTVMAQ	B	ALA35	CA	GLY17	O	3.912	DI	hydrophobic/van der Waals
**PZ3**	VEGMSGGTWVDVVLEHGGCVTVMAQ	B	ALA35	C	GLY17	O	3.599	DI	hydrophobic/van der Waals
**PZ3**	VEGMSGGTWVDVVLEHGGCVTVMAQ	B	ALA35	O	GLY17	O	3.674	DI	hydrophobic/van der Waals
**PZ3**	VEGMSGGTWVDVVLEHGGCVTVMAQ	B	GLN36	N	GLY17	O	3.951	DI	H-bond/polar candidate
**PZ3**	VEGMSGGTWVDVVLEHGGCVTVMAQ	B	ARG357	CD	GLY18	CA	3.954	DIII	H-bond/polar candidate
**PZ3**	VEGMSGGTWVDVVLEHGGCVTVMAQ	B	ASN362	OD1	CYS19	CB	3.796	DIII	H-bond/polar candidate
**PZ3**	VEGMSGGTWVDVVLEHGGCVTVMAQ	B	LEU358	N	CYS19	O	3.352	DIII	H-bond/polar candidate
**PZ3**	VEGMSGGTWVDVVLEHGGCVTVMAQ	B	LEU358	CB	CYS19	O	3.661	DIII	H-bond/polar candidate
**PZ3**	VEGMSGGTWVDVVLEHGGCVTVMAQ	B	LEU358	CG	CYS19	O	3.223	DIII	H-bond/polar candidate
**PZ3**	VEGMSGGTWVDVVLEHGGCVTVMAQ	B	LEU358	CD1	CYS19	O	3.74	DIII	H-bond/polar candidate
**PZ3**	VEGMSGGTWVDVVLEHGGCVTVMAQ	B	LEU358	CD1	CYS19	SG	3.994	DIII	H-bond/polar candidate
**PZ3**	VEGMSGGTWVDVVLEHGGCVTVMAQ	B	ALA361	CA	CYS19	SG	3.295	DIII	H-bond/polar candidate
**PZ3**	VEGMSGGTWVDVVLEHGGCVTVMAQ	B	ALA361	C	CYS19	SG	3.652	DIII	H-bond/polar candidate
**PZ3**	VEGMSGGTWVDVVLEHGGCVTVMAQ	B	ALA361	CB	CYS19	SG	3.15	DIII	H-bond/polar candidate
**PZ3**	VEGMSGGTWVDVVLEHGGCVTVMAQ	B	ASN362	N	CYS19	SG	3.026	DIII	H-bond/polar candidate
**PZ3**	VEGMSGGTWVDVVLEHGGCVTVMAQ	B	ASN362	CG	CYS19	SG	3.893	DIII	H-bond/polar candidate
**PZ3**	VEGMSGGTWVDVVLEHGGCVTVMAQ	B	ASN362	OD1	CYS19	SG	3.635	DIII	H-bond/polar candidate
**PZ3**	VEGMSGGTWVDVVLEHGGCVTVMAQ	B	PRO354	CB	VAL20	CB	3.061	DIII	hydrophobic/van der Waals
**PZ3**	VEGMSGGTWVDVVLEHGGCVTVMAQ	B	PRO354	CG	VAL20	CB	3.805	DIII	hydrophobic/van der Waals

**PZ3**	VEGMSGGTWVDVVLEHGGCVTVMAQ	B	PRO354	CA	VAL20	CG1	3.083	DIII	hydrophobic/van der Waals
**PZ3**	VEGMSGGTWVDVVLEHGGCVTVMAQ	B	PRO354	C	VAL20	CG1	3.727	DIII	hydrophobic/van der Waals
**PZ3**	VEGMSGGTWVDVVLEHGGCVTVMAQ	B	PRO354	CB	VAL20	CG1	1.86	DIII	hydrophobic/van der Waals
**PZ3**	VEGMSGGTWVDVVLEHGGCVTVMAQ	B	PRO354	CG	VAL20	CG1	2.794	DIII	hydrophobic/van der Waals
**PZ3**	VEGMSGGTWVDVVLEHGGCVTVMAQ	B	PRO354	CB	VAL20	CG2	3.93	DIII	hydrophobic/van der Waals
**PZ3**	VEGMSGGTWVDVVLEHGGCVTVMAQ	B	GLY356	C	VAL20	CG2	3.85	DIII	hydrophobic/van der Waals
**PZ3**	VEGMSGGTWVDVVLEHGGCVTVMAQ	B	GLY356	O	VAL20	CG2	2.865	DIII	hydrophobic/van der Waals
**PZ3**	VEGMSGGTWVDVVLEHGGCVTVMAQ	B	ARG357	CA	VAL20	CG2	3.79	DIII	H-bond/polar candidate
**PZ3**	VEGMSGGTWVDVVLEHGGCVTVMAQ	B	ALA343	O	THR21	N	3.786	DIII	H-bond/polar candidate
**PZ3**	VEGMSGGTWVDVVLEHGGCVTVMAQ	B	GLN344	CB	THR21	N	3.999	DIII	H-bond/polar candidate
**PZ3**	VEGMSGGTWVDVVLEHGGCVTVMAQ	B	GLN344	CB	THR21	C	3.715	DIII	H-bond/polar candidate
**PZ3**	VEGMSGGTWVDVVLEHGGCVTVMAQ	B	GLN344	CD	THR21	C	3.714	DIII	H-bond/polar candidate
**PZ3**	VEGMSGGTWVDVVLEHGGCVTVMAQ	B	GLN344	OE1	THR21	C	3.379	DIII	H-bond/polar candidate
**PZ3**	VEGMSGGTWVDVVLEHGGCVTVMAQ	B	PRO354	CG	THR21	C	3.707	DIII	H-bond/polar candidate
**PZ3**	VEGMSGGTWVDVVLEHGGCVTVMAQ	B	ALA343	O	THR21	CB	3.307	DIII	H-bond/polar candidate
**PZ3**	VEGMSGGTWVDVVLEHGGCVTVMAQ	B	GLN344	CB	THR21	O	2.637	DIII	H-bond/polar candidate
**PZ3**	VEGMSGGTWVDVVLEHGGCVTVMAQ	B	GLN344	CG	THR21	O	3.072	DIII	H-bond/polar candidate
**PZ3**	VEGMSGGTWVDVVLEHGGCVTVMAQ	B	GLN344	CD	THR21	O	2.608	DIII	H-bond/polar candidate
**PZ3**	VEGMSGGTWVDVVLEHGGCVTVMAQ	B	GLN344	OE1	THR21	O	2.572	DIII	H-bond/polar candidate
**PZ3**	VEGMSGGTWVDVVLEHGGCVTVMAQ	B	GLN344	NE2	THR21	O	3.214	DIII	H-bond/polar candidate
**PZ3**	VEGMSGGTWVDVVLEHGGCVTVMAQ	B	PRO354	CG	THR21	O	3.528	DIII	H-bond/polar candidate
**PZ3**	VEGMSGGTWVDVVLEHGGCVTVMAQ	B	ALA343	O	THR21	CG2	3.581	DIII	H-bond/polar candidate
**PZ3**	VEGMSGGTWVDVVLEHGGCVTVMAQ	B	ALA343	N	THR21	OG1	3.179	DIII	H-bond/polar candidate
**PZ3**	VEGMSGGTWVDVVLEHGGCVTVMAQ	B	ALA343	CA	THR21	OG1	3.582	DIII	H-bond/polar candidate
**PZ3**	VEGMSGGTWVDVVLEHGGCVTVMAQ	B	ALA343	C	THR21	OG1	2.779	DIII	H-bond/polar candidate
**PZ3**	VEGMSGGTWVDVVLEHGGCVTVMAQ	B	ALA343	O	THR21	OG1	2.085	DIII	H-bond/polar candidate
**PZ3**	VEGMSGGTWVDVVLEHGGCVTVMAQ	B	GLN344	N	THR21	OG1	3.522	DIII	H-bond/polar candidate
**PZ3**	VEGMSGGTWVDVVLEHGGCVTVMAQ	B	GLN344	CA	THR21	OG1	3.738	DIII	H-bond/polar candidate
**PZ3**	VEGMSGGTWVDVVLEHGGCVTVMAQ	B	GLN344	CB	THR21	OG1	3.219	DIII	H-bond/polar candidate
**PZ3**	VEGMSGGTWVDVVLEHGGCVTVMAQ	B	GLN344	OE1	VAL22	N	3.738	DIII	H-bond/polar candidate

**PZ3**	VEGMSGGTWVDVVLEHGGCVTVMAQ	B	PRO354	CG	VAL22	N	3.448	DIII	hydrophobic/van der Waals
**PZ3**	VEGMSGGTWVDVVLEHGGCVTVMAQ	B	GLN344	CD	VAL22	CA	3.914	DIII	H-bond/polar candidate
**PZ3**	VEGMSGGTWVDVVLEHGGCVTVMAQ	B	GLN344	OE1	VAL22	CA	3.255	DIII	H-bond/polar candidate
**PZ3**	VEGMSGGTWVDVVLEHGGCVTVMAQ	B	LEU352	O	VAL22	CA	3.647	DIII	hydrophobic/van der Waals
**PZ3**	VEGMSGGTWVDVVLEHGGCVTVMAQ	B	PRO354	CG	VAL22	CA	3.4	DIII	hydrophobic/van der Waals
**PZ3**	VEGMSGGTWVDVVLEHGGCVTVMAQ	B	PRO354	CD	VAL22	CA	3.766	DIII	hydrophobic/van der Waals
**PZ3**	VEGMSGGTWVDVVLEHGGCVTVMAQ	B	GLN344	OE1	VAL22	C	3.441	DIII	H-bond/polar candidate
**PZ3**	VEGMSGGTWVDVVLEHGGCVTVMAQ	B	LEU352	O	VAL22	CB	3.743	DIII	hydrophobic/van der Waals
**PZ3**	VEGMSGGTWVDVVLEHGGCVTVMAQ	B	PRO354	CG	VAL22	CB	3.07	DIII	hydrophobic/van der Waals
**PZ3**	VEGMSGGTWVDVVLEHGGCVTVMAQ	B	PRO354	CD	VAL22	CB	2.994	DIII	hydrophobic/van der Waals
**PZ3**	VEGMSGGTWVDVVLEHGGCVTVMAQ	B	LEU352	C	VAL22	CG1	3.717	DIII	hydrophobic/van der Waals
**PZ3**	VEGMSGGTWVDVVLEHGGCVTVMAQ	B	LEU352	O	VAL22	CG1	2.994	DIII	hydrophobic/van der Waals
**PZ3**	VEGMSGGTWVDVVLEHGGCVTVMAQ	B	THR353	N	VAL22	CG1	3.989	DIII	H-bond/polar candidate
**PZ3**	VEGMSGGTWVDVVLEHGGCVTVMAQ	B	THR353	CA	VAL22	CG1	3.58	DIII	H-bond/polar candidate
**PZ3**	VEGMSGGTWVDVVLEHGGCVTVMAQ	B	PRO354	CG	VAL22	CG1	3.888	DIII	hydrophobic/van der Waals
**PZ3**	VEGMSGGTWVDVVLEHGGCVTVMAQ	B	PRO354	CD	VAL22	CG1	3.226	DIII	hydrophobic/van der Waals
**PZ3**	VEGMSGGTWVDVVLEHGGCVTVMAQ	B	PRO354	N	VAL22	CG2	3.243	DIII	hydrophobic/van der Waals
**PZ3**	VEGMSGGTWVDVVLEHGGCVTVMAQ	B	PRO354	CA	VAL22	CG2	3.945	DIII	hydrophobic/van der Waals
**PZ3**	VEGMSGGTWVDVVLEHGGCVTVMAQ	B	PRO354	CB	VAL22	CG2	3.274	DIII	hydrophobic/van der Waals
**PZ3**	VEGMSGGTWVDVVLEHGGCVTVMAQ	B	PRO354	CG	VAL22	CG2	1.796	DIII	hydrophobic/van der Waals
**PZ3**	VEGMSGGTWVDVVLEHGGCVTVMAQ	B	PRO354	CD	VAL22	CG2	1.847	DIII	hydrophobic/van der Waals
**PZ3**	VEGMSGGTWVDVVLEHGGCVTVMAQ	B	GLN344	CD	MET23	N	3.835	DIII	H-bond/polar candidate
**PZ3**	VEGMSGGTWVDVVLEHGGCVTVMAQ	B	GLN344	OE1	MET23	N	2.714	DIII	H-bond/polar candidate
**PZ3**	VEGMSGGTWVDVVLEHGGCVTVMAQ	B	LEU352	O	MET23	N	3.65	DIII	hydrophobic/van der Waals
**PZ3**	VEGMSGGTWVDVVLEHGGCVTVMAQ	B	GLN344	OE1	MET23	CA	3.722	DIII	H-bond/polar candidate
**PZ3**	VEGMSGGTWVDVVLEHGGCVTVMAQ	B	LEU352	CB	MET23	C	3.843	DIII	hydrophobic/van der Waals
**PZ3**	VEGMSGGTWVDVVLEHGGCVTVMAQ	B	GLN344	OE1	MET23	CB	3.592	DIII	H-bond/polar candidate
**PZ3**	VEGMSGGTWVDVVLEHGGCVTVMAQ	B	THR351	O	MET23	O	3.471	DIII	H-bond/polar candidate
**PZ3**	VEGMSGGTWVDVVLEHGGCVTVMAQ	B	LEU352	CA	MET23	O	3.928	DIII	hydrophobic/van der Waals
**PZ3**	VEGMSGGTWVDVVLEHGGCVTVMAQ	B	LEU352	O	MET23	O	3.773	DIII	hydrophobic/van der Waals

**PZ3**	VEGMSGGTWVDVVLEHGGCVTVMAQ	B	LEU352	CB	MET23	O	2.799	DIII	hydrophobic/van der Waals
**PZ3**	VEGMSGGTWVDVVLEHGGCVTVMAQ	B	LEU352	CG	MET23	O	3.632	DIII	hydrophobic/van der Waals
**PZ3**	VEGMSGGTWVDVVLEHGGCVTVMAQ	B	GLN344	OE1	MET23	CG	3.205	DIII	H-bond/polar candidate
**PZ3**	VEGMSGGTWVDVVLEHGGCVTVMAQ	B	PRO342	CB	MET23	SD	3.864	DIII	hydrophobic/van der Waals
**PZ3**	VEGMSGGTWVDVVLEHGGCVTVMAQ	B	VAL391	CG1	MET23	SD	3.474	DIII	hydrophobic/van der Waals
**PZ3**	VEGMSGGTWVDVVLEHGGCVTVMAQ	B	VAL391	CA	MET23	CE	3.88	DIII	hydrophobic/van der Waals
**PZ3**	VEGMSGGTWVDVVLEHGGCVTVMAQ	B	VAL391	CB	MET23	CE	3.196	DIII	hydrophobic/van der Waals
**PZ3**	VEGMSGGTWVDVVLEHGGCVTVMAQ	B	VAL391	CG1	MET23	CE	2.268	DIII	hydrophobic/van der Waals
**PZ3**	VEGMSGGTWVDVVLEHGGCVTVMAQ	B	GLN350	O	GLN25	CG	3.666	DIII	H-bond/polar candidate
**PZ3**	VEGMSGGTWVDVVLEHGGCVTVMAQ	B	LEU352	CD2	GLN25	CG	3.94	DIII	H-bond/polar candidate
**PZ3**	VEGMSGGTWVDVVLEHGGCVTVMAQ	B	MET349	O	GLN25	CD	3.774	DIII	H-bond/polar candidate
**PZ3**	VEGMSGGTWVDVVLEHGGCVTVMAQ	B	GLN350	C	GLN25	CD	3.975	DIII	H-bond/polar candidate
**PZ3**	VEGMSGGTWVDVVLEHGGCVTVMAQ	B	GLN350	O	GLN25	CD	3.239	DIII	H-bond/polar candidate
**PZ3**	VEGMSGGTWVDVVLEHGGCVTVMAQ	B	GLN350	OE1	GLN25	CD	3.368	DIII	H-bond/polar candidate
**PZ3**	VEGMSGGTWVDVVLEHGGCVTVMAQ	B	MET349	C	GLN25	NE2	3.885	DIII	H-bond/polar candidate
**PZ3**	VEGMSGGTWVDVVLEHGGCVTVMAQ	B	MET349	O	GLN25	NE2	2.766	DIII	H-bond/polar candidate
**PZ3**	VEGMSGGTWVDVVLEHGGCVTVMAQ	B	GLN350	CA	GLN25	NE2	3.842	DIII	H-bond/polar candidate
**PZ3**	VEGMSGGTWVDVVLEHGGCVTVMAQ	B	GLN350	O	GLN25	NE2	3.769	DIII	H-bond/polar candidate
**PZ3**	VEGMSGGTWVDVVLEHGGCVTVMAQ	B	GLN350	OE1	GLN25	NE2	3.487	DIII	H-bond/polar candidate
**PZ3**	VEGMSGGTWVDVVLEHGGCVTVMAQ	B	LEU352	CD2	GLN25	NE2	3.417	DIII	H-bond/polar candidate
**PZ3**	VEGMSGGTWVDVVLEHGGCVTVMAQ	B	GLN350	CA	GLN25	OE1	3.851	DIII	H-bond/polar candidate
**PZ3**	VEGMSGGTWVDVVLEHGGCVTVMAQ	B	GLN350	C	GLN25	OE1	3.893	DIII	H-bond/polar candidate
**PZ3**	VEGMSGGTWVDVVLEHGGCVTVMAQ	B	GLN350	O	GLN25	OE1	3.119	DIII	H-bond/polar candidate
**PZ3**	VEGMSGGTWVDVVLEHGGCVTVMAQ	B	GLN350	CD	GLN25	OE1	3.689	DIII	H-bond/polar candidate
**PZ3**	VEGMSGGTWVDVVLEHGGCVTVMAQ	B	GLN350	OE1	GLN25	OE1	2.545	DIII	H-bond/polar candidate
**PZ4**	TDGPCKVPAQMAVDMQTLTPVGRLI	B	HIS27	C	PRO4	CA	3.956	DI	H-bond/polar candidate
**PZ4**	TDGPCKVPAQMAVDMQTLTPVGRLI	B	HIS27	O	PRO4	CA	3.008	DI	H-bond/polar candidate
**PZ4**	TDGPCKVPAQMAVDMQTLTPVGRLI	B	HIS27	O	PRO4	C	3.368	DI	H-bond/polar candidate
**PZ4**	TDGPCKVPAQMAVDMQTLTPVGRLI	B	HIS27	CA	PRO4	CB	3.623	DI	H-bond/polar candidate
**PZ4**	TDGPCKVPAQMAVDMQTLTPVGRLI	B	HIS27	C	PRO4	CB	3.35	DI	H-bond/polar candidate

**PZ4**	TDGPCKVPAQMAVDMQTLTPVGRLI	B	HIS27	O	PRO4	CB	2.84	DI	H-bond/polar candidate
**PZ4**	TDGPCKVPAQMAVDMQTLTPVGRLI	B	HIS27	CB	PRO4	CB	2.999	DI	H-bond/polar candidate
**PZ4**	TDGPCKVPAQMAVDMQTLTPVGRLI	B	SER285	O	PRO4	CB	3.397	DII	H-bond/polar candidate
**PZ4**	TDGPCKVPAQMAVDMQTLTPVGRLI	B	HIS27	CB	PRO4	CG	3.705	DI	H-bond/polar candidate
**PZ4**	TDGPCKVPAQMAVDMQTLTPVGRLI	B	SER285	C	PRO4	CG	3.484	DII	H-bond/polar candidate
**PZ4**	TDGPCKVPAQMAVDMQTLTPVGRLI	B	SER285	O	PRO4	CG	2.267	DII	H-bond/polar candidate
**PZ4**	TDGPCKVPAQMAVDMQTLTPVGRLI	B	SER285	O	PRO4	CD	3.256	DII	H-bond/polar candidate
**PZ4**	TDGPCKVPAQMAVDMQTLTPVGRLI	B	HIS27	O	CYS5	N	3.278	DI	H-bond/polar candidate
**PZ4**	TDGPCKVPAQMAVDMQTLTPVGRLI	B	ASN8	CB	CYS5	CB	3.239	DI	H-bond/polar candidate
**PZ4**	TDGPCKVPAQMAVDMQTLTPVGRLI	B	GLY28	O	CYS5	CB	3.707	DI	H-bond/polar candidate
**PZ4**	TDGPCKVPAQMAVDMQTLTPVGRLI	B	HIS27	O	CYS5	O	3.889	DI	H-bond/polar candidate
**PZ4**	TDGPCKVPAQMAVDMQTLTPVGRLI	B	GLY28	CA	CYS5	O	3.269	DI	H-bond/polar candidate
**PZ4**	TDGPCKVPAQMAVDMQTLTPVGRLI	B	GLY28	C	CYS5	O	3.955	DI	H-bond/polar candidate
**PZ4**	TDGPCKVPAQMAVDMQTLTPVGRLI	B	GLY28	O	CYS5	O	3.988	DI	H-bond/polar candidate
**PZ4**	TDGPCKVPAQMAVDMQTLTPVGRLI	B	SER7	N	CYS5	SG	3.828	DI	H-bond/polar candidate
**PZ4**	TDGPCKVPAQMAVDMQTLTPVGRLI	B	SER7	CB	CYS5	SG	3.486	DI	H-bond/polar candidate
**PZ4**	TDGPCKVPAQMAVDMQTLTPVGRLI	B	SER7	OG	CYS5	SG	3.88	DI	H-bond/polar candidate
**PZ4**	TDGPCKVPAQMAVDMQTLTPVGRLI	B	ASN8	N	CYS5	SG	3.589	DI	H-bond/polar candidate
**PZ4**	TDGPCKVPAQMAVDMQTLTPVGRLI	B	ASN8	CB	CYS5	SG	3.928	DI	H-bond/polar candidate
**PZ4**	TDGPCKVPAQMAVDMQTLTPVGRLI	A	ASP247	N	LYS6	NZ	3.794	DII	salt bridge candidate; H-bond/polar candidate
**PZ4**	TDGPCKVPAQMAVDMQTLTPVGRLI	A	ASP247	CA	LYS6	NZ	3.649	DII	salt bridge candidate; H-bond/polar candidate
**PZ4**	TDGPCKVPAQMAVDMQTLTPVGRLI	A	HIS249	ND1	VAL7	O	3.772	DII	H-bond/polar candidate
**PZ4**	TDGPCKVPAQMAVDMQTLTPVGRLI	A	HIS249	CE1	VAL7	O	3.762	DII	H-bond/polar candidate
**PZ4**	TDGPCKVPAQMAVDMQTLTPVGRLI	B	GLY28	O	VAL7	CG2	3.626	DI	hydrophobic/van der Waals
**PZ4**	TDGPCKVPAQMAVDMQTLTPVGRLI	A	HIS249	CA	PRO8	CA	3.843	DII	H-bond/polar candidate
**PZ4**	TDGPCKVPAQMAVDMQTLTPVGRLI	A	HIS249	CB	PRO8	CA	3.877	DII	H-bond/polar candidate
**PZ4**	TDGPCKVPAQMAVDMQTLTPVGRLI	A	HIS249	CG	PRO8	CA	3.814	DII	H-bond/polar candidate
**PZ4**	TDGPCKVPAQMAVDMQTLTPVGRLI	A	HIS249	ND1	PRO8	CA	3.443	DII	H-bond/polar candidate
**PZ4**	TDGPCKVPAQMAVDMQTLTPVGRLI	A	ALA250	N	PRO8	CA	3.969	DII	hydrophobic/van der Waals
**PZ4**	TDGPCKVPAQMAVDMQTLTPVGRLI	A	HIS249	ND1	PRO8	C	3.907	DII	H-bond/polar candidate

**PZ4**	TDGPCKVPAQMAVDMQTLTPVGRLI	A	HIS249	CA	PRO8	CB	3.649	DII	H-bond/polar candidate
**PZ4**	TDGPCKVPAQMAVDMQTLTPVGRLI	A	HIS249	C	PRO8	CB	3.668	DII	H-bond/polar candidate
**PZ4**	TDGPCKVPAQMAVDMQTLTPVGRLI	A	HIS249	CB	PRO8	CB	3.923	DII	H-bond/polar candidate
**PZ4**	TDGPCKVPAQMAVDMQTLTPVGRLI	A	ALA250	N	PRO8	CB	2.881	DII	hydrophobic/van der Waals
**PZ4**	TDGPCKVPAQMAVDMQTLTPVGRLI	A	ALA250	CA	PRO8	CB	3.725	DII	hydrophobic/van der Waals
**PZ4**	TDGPCKVPAQMAVDMQTLTPVGRLI	A	ALA250	CB	PRO8	CB	3.609	DII	hydrophobic/van der Waals
**PZ4**	TDGPCKVPAQMAVDMQTLTPVGRLI	A	ALA250	N	PRO8	CG	3.525	DII	hydrophobic/van der Waals
**PZ4**	TDGPCKVPAQMAVDMQTLTPVGRLI	A	HIS249	CB	ALA9	N	3.752	DII	H-bond/polar candidate
**PZ4**	TDGPCKVPAQMAVDMQTLTPVGRLI	A	HIS249	CG	ALA9	N	3.26	DII	H-bond/polar candidate
**PZ4**	TDGPCKVPAQMAVDMQTLTPVGRLI	A	HIS249	ND1	ALA9	N	3.397	DII	H-bond/polar candidate
**PZ4**	TDGPCKVPAQMAVDMQTLTPVGRLI	A	HIS249	CD2	ALA9	N	3.434	DII	H-bond/polar candidate
**PZ4**	TDGPCKVPAQMAVDMQTLTPVGRLI	A	HIS249	CE1	ALA9	N	3.655	DII	H-bond/polar candidate
**PZ4**	TDGPCKVPAQMAVDMQTLTPVGRLI	A	HIS249	NE2	ALA9	N	3.677	DII	H-bond/polar candidate
**PZ4**	TDGPCKVPAQMAVDMQTLTPVGRLI	A	HIS249	CD2	ALA9	CA	3.858	DII	H-bond/polar candidate
**PZ4**	TDGPCKVPAQMAVDMQTLTPVGRLI	A	HIS249	CG	ALA9	CB	3.811	DII	H-bond/polar candidate
**PZ4**	TDGPCKVPAQMAVDMQTLTPVGRLI	A	HIS249	CD2	ALA9	CB	3.081	DII	H-bond/polar candidate
**PZ4**	TDGPCKVPAQMAVDMQTLTPVGRLI	A	HIS249	NE2	ALA9	CB	3.38	DII	H-bond/polar candidate
**PZ4**	TDGPCKVPAQMAVDMQTLTPVGRLI	A	LYS251	N	GLN10	CD	3.511	DII	H-bond/polar candidate
**PZ4**	TDGPCKVPAQMAVDMQTLTPVGRLI	A	LYS251	N	GLN10	NE2	3.06	DII	H-bond/polar candidate
**PZ4**	TDGPCKVPAQMAVDMQTLTPVGRLI	A	LYS251	CA	GLN10	NE2	3.364	DII	H-bond/polar candidate
**PZ4**	TDGPCKVPAQMAVDMQTLTPVGRLI	A	LYS251	C	GLN10	NE2	3.51	DII	H-bond/polar candidate
**PZ4**	TDGPCKVPAQMAVDMQTLTPVGRLI	A	LYS251	CB	GLN10	NE2	3.168	DII	H-bond/polar candidate
**PZ4**	TDGPCKVPAQMAVDMQTLTPVGRLI	A	LYS251	CG	GLN10	NE2	3.968	DII	H-bond/polar candidate
**PZ4**	TDGPCKVPAQMAVDMQTLTPVGRLI	A	ARG252	N	GLN10	NE2	3.474	DII	H-bond/polar candidate
**PZ4**	TDGPCKVPAQMAVDMQTLTPVGRLI	A	ARG252	CG	GLN10	NE2	3.356	DII	H-bond/polar candidate
**PZ4**	TDGPCKVPAQMAVDMQTLTPVGRLI	A	ARG252	CD	GLN10	NE2	3.941	DII	H-bond/polar candidate
**PZ4**	TDGPCKVPAQMAVDMQTLTPVGRLI	A	HIS249	CB	GLN10	OE1	3.27	DII	H-bond/polar candidate
**PZ4**	TDGPCKVPAQMAVDMQTLTPVGRLI	A	ALA250	N	GLN10	OE1	3.443	DII	H-bond/polar candidate
**PZ4**	TDGPCKVPAQMAVDMQTLTPVGRLI	A	ALA250	CB	GLN10	OE1	3.891	DII	H-bond/polar candidate
**PZ4**	TDGPCKVPAQMAVDMQTLTPVGRLI	A	LYS251	N	GLN10	OE1	3.735	DII	H-bond/polar candidate

**PZ4**	TDGPCKVPAQMAVDMQTLTPVGRLI	A	ASN103	CB	MET11	CG	3.589	fusion-loop region	H-bond/polar candidate
**PZ4**	TDGPCKVPAQMAVDMQTLTPVGRLI	A	ASN103	CG	MET11	CG	3.642	fusion-loop region	H-bond/polar candidate
**PZ4**	TDGPCKVPAQMAVDMQTLTPVGRLI	A	ASN103	ND2	MET11	CG	2.782	fusion-loop region	H-bond/polar candidate
**PZ4**	TDGPCKVPAQMAVDMQTLTPVGRLI	A	ASN103	ND2	MET11	SD	2.949	fusion-loop region	H-bond/polar candidate
**PZ4**	TDGPCKVPAQMAVDMQTLTPVGRLI	A	ASP98	C	MET11	CE	3.918	fusion-loop region	H-bond/polar candidate
**PZ4**	TDGPCKVPAQMAVDMQTLTPVGRLI	A	ASP98	O	MET11	CE	3.192	fusion-loop region	H-bond/polar candidate
**PZ4**	TDGPCKVPAQMAVDMQTLTPVGRLI	A	ASN103	CG	MET11	CE	3.053	fusion-loop region	H-bond/polar candidate
**PZ4**	TDGPCKVPAQMAVDMQTLTPVGRLI	A	ASN103	OD1	MET11	CE	3.729	fusion-loop region	H-bond/polar candidate
**PZ4**	TDGPCKVPAQMAVDMQTLTPVGRLI	A	ASN103	ND2	MET11	CE	1.77	fusion-loop region	H-bond/polar candidate
**PZ4**	TDGPCKVPAQMAVDMQTLTPVGRLI	A	GLY102	O	ASP14	CB	2.984	fusion-loop region	H-bond/polar candidate
**PZ4**	TDGPCKVPAQMAVDMQTLTPVGRLI	A	GLY102	O	ASP14	CG	2.998	fusion-loop region	H-bond/polar candidate
**PZ4**	TDGPCKVPAQMAVDMQTLTPVGRLI	A	GLY102	C	ASP14	OD2	3.374	fusion-loop region	H-bond/polar candidate
**PZ4**	TDGPCKVPAQMAVDMQTLTPVGRLI	A	GLY102	O	ASP14	OD2	2.311	fusion-loop region	H-bond/polar candidate

**PZ4**	TDGPCKVPAQMAVDMQTLTPVGRLI	A	ASN103	N	ASP14	OD2	3.821	fusion-loop region	H-bond/polar candidate
**PZ4**	TDGPCKVPAQMAVDMQTLTPVGRLI	A	ASN103	CA	ASP14	OD2	3.333	fusion-loop region	H-bond/polar candidate
**PZ4**	TDGPCKVPAQMAVDMQTLTPVGRLI	B	ARG2	NH1	MET15	CB	3.439	DI	H-bond/polar candidate
**PZ4**	TDGPCKVPAQMAVDMQTLTPVGRLI	B	ARG2	NH1	MET15	O	3.829	DI	H-bond/polar candidate
**PZ4**	TDGPCKVPAQMAVDMQTLTPVGRLI	B	ARG2	NE	MET15	CG	3.916	DI	H-bond/polar candidate
**PZ4**	TDGPCKVPAQMAVDMQTLTPVGRLI	B	ARG2	CZ	MET15	CG	2.78	DI	H-bond/polar candidate
**PZ4**	TDGPCKVPAQMAVDMQTLTPVGRLI	B	ARG2	NH1	MET15	CG	1.97	DI	H-bond/polar candidate
**PZ4**	TDGPCKVPAQMAVDMQTLTPVGRLI	B	ARG2	NH2	MET15	CG	3.041	DI	H-bond/polar candidate
**PZ4**	TDGPCKVPAQMAVDMQTLTPVGRLI	B	ARG2	NE	MET15	SD	3.2	DI	H-bond/polar candidate
**PZ4**	TDGPCKVPAQMAVDMQTLTPVGRLI	B	ARG2	CZ	MET15	SD	2.11	DI	H-bond/polar candidate
**PZ4**	TDGPCKVPAQMAVDMQTLTPVGRLI	B	ARG2	NH1	MET15	SD	2.328	DI	H-bond/polar candidate
**PZ4**	TDGPCKVPAQMAVDMQTLTPVGRLI	B	ARG2	NH2	MET15	SD	1.725	DI	H-bond/polar candidate
**PZ4**	TDGPCKVPAQMAVDMQTLTPVGRLI	B	ARG2	CZ	MET15	CE	3.572	DI	H-bond/polar candidate
**PZ4**	TDGPCKVPAQMAVDMQTLTPVGRLI	B	ARG2	NH1	MET15	CE	3.773	DI	H-bond/polar candidate
**PZ4**	TDGPCKVPAQMAVDMQTLTPVGRLI	B	ARG2	NH2	MET15	CE	3.299	DI	H-bond/polar candidate
**PZ4**	TDGPCKVPAQMAVDMQTLTPVGRLI	B	SER146	C	PRO20	CA	3.917	DI	H-bond/polar candidate
**PZ4**	TDGPCKVPAQMAVDMQTLTPVGRLI	B	SER146	O	PRO20	CA	2.725	DI	H-bond/polar candidate
**PZ4**	TDGPCKVPAQMAVDMQTLTPVGRLI	B	SER146	O	PRO20	C	2.871	DI	H-bond/polar candidate
**PZ4**	TDGPCKVPAQMAVDMQTLTPVGRLI	B	SER146	O	PRO20	CB	3.394	DI	H-bond/polar candidate
**PZ4**	TDGPCKVPAQMAVDMQTLTPVGRLI	B	SER146	C	VAL21	N	3.517	DI	H-bond/polar candidate
**PZ4**	TDGPCKVPAQMAVDMQTLTPVGRLI	B	SER146	O	VAL21	N	2.47	DI	H-bond/polar candidate
**PZ4**	TDGPCKVPAQMAVDMQTLTPVGRLI	B	SER146	O	VAL21	CA	3.481	DI	H-bond/polar candidate
**PZ4**	TDGPCKVPAQMAVDMQTLTPVGRLI	B	SER146	O	VAL21	C	3.7	DI	H-bond/polar candidate
**PZ4**	TDGPCKVPAQMAVDMQTLTPVGRLI	B	SER146	O	VAL21	CB	3.949	DI	H-bond/polar candidate
**PZ4**	TDGPCKVPAQMAVDMQTLTPVGRLI	B	SER146	CA	VAL21	O	3.318	DI	H-bond/polar candidate
**PZ4**	TDGPCKVPAQMAVDMQTLTPVGRLI	B	SER146	C	VAL21	O	3.487	DI	H-bond/polar candidate
**PZ4**	TDGPCKVPAQMAVDMQTLTPVGRLI	B	SER146	O	VAL21	O	3.09	DI	H-bond/polar candidate

**PZ4**	TDGPCKVPAQMAVDMQTLTPVGRLI	B	SER146	CB	VAL21	O	3.864	DI	H-bond/polar candidate
**PZ4**	TDGPCKVPAQMAVDMQTLTPVGRLI	B	ASN163	ND2	GLY22	C	3.819	DI	H-bond/polar candidate
**PZ4**	TDGPCKVPAQMAVDMQTLTPVGRLI	B	SER146	CA	GLY22	O	3.921	DI	H-bond/polar candidate
**PZ4**	TDGPCKVPAQMAVDMQTLTPVGRLI	B	ASN163	ND2	GLY22	O	3.571	DI	H-bond/polar candidate
**PZ4**	TDGPCKVPAQMAVDMQTLTPVGRLI	B	ASN163	ND2	ARG23	N	2.999	DI	H-bond/polar candidate
**PZ4**	TDGPCKVPAQMAVDMQTLTPVGRLI	B	ASN163	CG	ARG23	CA	3.547	DI	H-bond/polar candidate
**PZ4**	TDGPCKVPAQMAVDMQTLTPVGRLI	B	ASN163	OD1	ARG23	CA	3.586	DI	H-bond/polar candidate
**PZ4**	TDGPCKVPAQMAVDMQTLTPVGRLI	B	ASN163	ND2	ARG23	CA	2.759	DI	H-bond/polar candidate
**PZ4**	TDGPCKVPAQMAVDMQTLTPVGRLI	B	ASN163	CG	ARG23	CB	3.612	DI	H-bond/polar candidate
**PZ4**	TDGPCKVPAQMAVDMQTLTPVGRLI	B	ASN163	OD1	ARG23	CB	3.17	DI	H-bond/polar candidate
**PZ4**	TDGPCKVPAQMAVDMQTLTPVGRLI	B	ASN163	ND2	ARG23	CB	3.246	DI	H-bond/polar candidate
**PZ4**	TDGPCKVPAQMAVDMQTLTPVGRLI	B	LYS301	CE	ARG23	CB	3.877	DI	H-bond/polar candidate
**PZ4**	TDGPCKVPAQMAVDMQTLTPVGRLI	B	LYS301	NZ	ARG23	CB	2.739	DI	H-bond/polar candidate
**PZ4**	TDGPCKVPAQMAVDMQTLTPVGRLI	B	THR366	CG2	ARG23	O	3.675	DIII	H-bond/polar candidate
**PZ4**	TDGPCKVPAQMAVDMQTLTPVGRLI	B	ASN163	CG	ARG23	CG	2.714	DI	H-bond/polar candidate
**PZ4**	TDGPCKVPAQMAVDMQTLTPVGRLI	B	ASN163	OD1	ARG23	CG	2.098	DI	H-bond/polar candidate
**PZ4**	TDGPCKVPAQMAVDMQTLTPVGRLI	B	ASN163	ND2	ARG23	CG	2.725	DI	H-bond/polar candidate
**PZ4**	TDGPCKVPAQMAVDMQTLTPVGRLI	B	LYS301	NZ	ARG23	CG	3.417	DI	H-bond/polar candidate
**PZ4**	TDGPCKVPAQMAVDMQTLTPVGRLI	B	ASN163	CG	ARG23	CD	3.789	DI	H-bond/polar candidate
**PZ4**	TDGPCKVPAQMAVDMQTLTPVGRLI	B	ASN163	OD1	ARG23	CD	2.825	DI	H-bond/polar candidate
**PZ4**	TDGPCKVPAQMAVDMQTLTPVGRLI	B	LYS301	CE	ARG23	CD	3.486	DI	H-bond/polar candidate
**PZ4**	TDGPCKVPAQMAVDMQTLTPVGRLI	B	LYS301	NZ	ARG23	CD	3.001	DI	H-bond/polar candidate
**PZ4**	TDGPCKVPAQMAVDMQTLTPVGRLI	B	GLU162	N	ARG23	NE	3.262	DI	salt bridge candidate; H-bond/polar candidate
**PZ4**	TDGPCKVPAQMAVDMQTLTPVGRLI	B	GLU162	C	ARG23	NE	3.859	DI	salt bridge candidate; H-bond/polar candidate
**PZ4**	TDGPCKVPAQMAVDMQTLTPVGRLI	B	GLU162	O	ARG23	NE	3.809	DI	salt bridge candidate; H-bond/polar candidate
**PZ4**	TDGPCKVPAQMAVDMQTLTPVGRLI	B	ASN163	OD1	ARG23	NE	3.171	DI	H-bond/polar candidate
**PZ4**	TDGPCKVPAQMAVDMQTLTPVGRLI	B	GLY181	CA	ARG23	NH1	3.665	DI	H-bond/polar candidate
**PZ4**	TDGPCKVPAQMAVDMQTLTPVGRLI	B	GLY181	C	ARG23	NH1	3.96	DI	H-bond/polar candidate
**PZ4**	TDGPCKVPAQMAVDMQTLTPVGRLI	B	GLY182	N	ARG23	NH1	3.299	DI	H-bond/polar candidate
**PZ4**	TDGPCKVPAQMAVDMQTLTPVGRLI	B	GLU162	N	ARG23	NH2	2.598	DI	salt bridge candidate; H-bond/polar candidate

**PZ4**	TDGPCKVPAQMAVDMQTLTPVGRLI	B	GLU162	CA	ARG23	NH2	3.541	DI	salt bridge candidate; H-bond/polar candidate
**PZ4**	TDGPCKVPAQMAVDMQTLTPVGRLI	B	GLU162	C	ARG23	NH2	3.864	DI	salt bridge candidate; H-bond/polar candidate
**PZ4**	TDGPCKVPAQMAVDMQTLTPVGRLI	B	GLU162	O	ARG23	NH2	3.713	DI	salt bridge candidate; H-bond/polar candidate
**PZ4**	TDGPCKVPAQMAVDMQTLTPVGRLI	B	GLU162	CB	ARG23	NH2	3.792	DI	salt bridge candidate; H-bond/polar candidate
**PZ4**	TDGPCKVPAQMAVDMQTLTPVGRLI	B	GLY181	CA	ARG23	NH2	3.763	DI	H-bond/polar candidate
**PZ4**	TDGPCKVPAQMAVDMQTLTPVGRLI	B	GLU162	N	ARG23	CZ	3.352	DI	salt bridge candidate; H-bond/polar candidate
**PZ4**	TDGPCKVPAQMAVDMQTLTPVGRLI	B	GLU162	O	ARG23	CZ	3.994	DI	salt bridge candidate; H-bond/polar candidate
**PZ4**	TDGPCKVPAQMAVDMQTLTPVGRLI	B	GLY181	CA	ARG23	CZ	3.802	DI	H-bond/polar candidate
**PZ4**	TDGPCKVPAQMAVDMQTLTPVGRLI	B	PHE183	CZ	LEU24	C	3.507	DI	hydrophobic/van der Waals
**PZ4**	TDGPCKVPAQMAVDMQTLTPVGRLI	B	PHE183	CE1	LEU24	O	3.491	DI	hydrophobic/van der Waals
**PZ4**	TDGPCKVPAQMAVDMQTLTPVGRLI	B	PHE183	CE2	LEU24	O	2.957	DI	hydrophobic/van der Waals
**PZ4**	TDGPCKVPAQMAVDMQTLTPVGRLI	B	PHE183	CZ	LEU24	O	2.341	DI	hydrophobic/van der Waals
**PZ4**	TDGPCKVPAQMAVDMQTLTPVGRLI	B	ALA361	O	LEU24	CG	3.273	DIII	hydrophobic/van der Waals
**PZ4**	TDGPCKVPAQMAVDMQTLTPVGRLI	B	ASN362	C	LEU24	CG	3.958	DIII	H-bond/polar candidate
**PZ4**	TDGPCKVPAQMAVDMQTLTPVGRLI	B	ASN362	O	LEU24	CG	3.348	DIII	H-bond/polar candidate
**PZ4**	TDGPCKVPAQMAVDMQTLTPVGRLI	B	ALA361	O	LEU24	CD1	3.888	DIII	hydrophobic/van der Waals
**PZ4**	TDGPCKVPAQMAVDMQTLTPVGRLI	B	ASN362	C	LEU24	CD1	3.731	DIII	H-bond/polar candidate
**PZ4**	TDGPCKVPAQMAVDMQTLTPVGRLI	B	ASN362	O	LEU24	CD1	3.229	DIII	H-bond/polar candidate
**PZ4**	TDGPCKVPAQMAVDMQTLTPVGRLI	B	PRO363	N	LEU24	CD1	3.643	DIII	hydrophobic/van der Waals
**PZ4**	TDGPCKVPAQMAVDMQTLTPVGRLI	B	PRO363	CA	LEU24	CD1	2.906	DIII	hydrophobic/van der Waals
**PZ4**	TDGPCKVPAQMAVDMQTLTPVGRLI	B	PRO363	C	LEU24	CD1	3.572	DIII	hydrophobic/van der Waals
**PZ4**	TDGPCKVPAQMAVDMQTLTPVGRLI	B	PRO363	CB	LEU24	CD1	3.899	DIII	hydrophobic/van der Waals
**PZ4**	TDGPCKVPAQMAVDMQTLTPVGRLI	B	VAL364	N	LEU24	CD1	3.288	DIII	hydrophobic/van der Waals
**PZ4**	TDGPCKVPAQMAVDMQTLTPVGRLI	B	MET374	CE	LEU24	CD1	3.573	DIII	hydrophobic/van der Waals
**PZ4**	TDGPCKVPAQMAVDMQTLTPVGRLI	B	HIS144	O	LEU24	CD2	3.754	DI	H-bond/polar candidate
**PZ4**	TDGPCKVPAQMAVDMQTLTPVGRLI	B	THR360	C	LEU24	CD2	3.976	DIII	H-bond/polar candidate
**PZ4**	TDGPCKVPAQMAVDMQTLTPVGRLI	B	ALA361	N	LEU24	CD2	3.913	DIII	hydrophobic/van der Waals
**PZ4**	TDGPCKVPAQMAVDMQTLTPVGRLI	B	ALA361	C	LEU24	CD2	3.716	DIII	hydrophobic/van der Waals
**PZ4**	TDGPCKVPAQMAVDMQTLTPVGRLI	B	ALA361	O	LEU24	CD2	2.558	DIII	hydrophobic/van der Waals
**PZ4**	TDGPCKVPAQMAVDMQTLTPVGRLI	B	ASN362	O	ILE25	C	3.8	DIII	H-bond/polar candidate

**PZ4**	TDGPCKVPAQMAVDMQTLTPVGRLI	B	VAL364	CG2	ILE25	C	3.805	DIII	hydrophobic/van der Waals
**PZ4**	TDGPCKVPAQMAVDMQTLTPVGRLI	B	LYS301	CD	ILE25	CB	3.919	DI	H-bond/polar candidate
**PZ4**	TDGPCKVPAQMAVDMQTLTPVGRLI	B	TYR305	CE2	ILE25	CB	3.695	DIII	H-bond/polar candidate; hydrophobic/van der Waals
**PZ4**	TDGPCKVPAQMAVDMQTLTPVGRLI	B	ASN362	C	ILE25	O	3.921	DIII	H-bond/polar candidate
**PZ4**	TDGPCKVPAQMAVDMQTLTPVGRLI	B	ASN362	O	ILE25	O	2.733	DIII	H-bond/polar candidate
**PZ4**	TDGPCKVPAQMAVDMQTLTPVGRLI	B	VAL364	CB	ILE25	O	3.707	DIII	hydrophobic/van der Waals
**PZ4**	TDGPCKVPAQMAVDMQTLTPVGRLI	B	VAL364	CG2	ILE25	O	2.859	DIII	hydrophobic/van der Waals
**PZ4**	TDGPCKVPAQMAVDMQTLTPVGRLI	B	LYS301	NZ	ILE25	CG1	3.762	DI	H-bond/polar candidate
**PZ4**	TDGPCKVPAQMAVDMQTLTPVGRLI	B	TYR305	OH	ILE25	CG1	3.986	DIII	H-bond/polar candidate; hydrophobic/van der Waals
**PZ4**	TDGPCKVPAQMAVDMQTLTPVGRLI	B	LYS301	CB	ILE25	CG2	3.31	DI	H-bond/polar candidate
**PZ4**	TDGPCKVPAQMAVDMQTLTPVGRLI	B	LYS301	CG	ILE25	CG2	3.801	DI	H-bond/polar candidate
**PZ4**	TDGPCKVPAQMAVDMQTLTPVGRLI	B	LYS301	CD	ILE25	CG2	3.202	DI	H-bond/polar candidate
**PZ4**	TDGPCKVPAQMAVDMQTLTPVGRLI	B	TYR305	CE2	ILE25	CG2	3.606	DIII	H-bond/polar candidate; hydrophobic/van der Waals
**PZ4**	TDGPCKVPAQMAVDMQTLTPVGRLI	B	LYS301	CD	ILE25	CD1	3.298	DI	H-bond/polar candidate
**PZ4**	TDGPCKVPAQMAVDMQTLTPVGRLI	B	LYS301	CE	ILE25	CD1	3.817	DI	H-bond/polar candidate
**PZ4**	TDGPCKVPAQMAVDMQTLTPVGRLI	B	LYS301	NZ	ILE25	CD1	3.12	DI	H-bond/polar candidate
**PZ4**	TDGPCKVPAQMAVDMQTLTPVGRLI	B	TYR305	OH	ILE25	CD1	3.545	DIII	H-bond/polar candidate; hydrophobic/van der Waals
**PZ5**	GLIYHREEKRWRWINNSVFNGNVTN	A	PRO222	CG	GLY1	N	3.762	DII	hydrophobic/van der Waals
**PZ5**	GLIYHREEKRWRWINNSVFNGNVTN	A	PRO222	O	GLY1	N	3.823	DII	hydrophobic/van der Waals
**PZ5**	GLIYHREEKRWRWINNSVFNGNVTN	A	PRO222	CD	GLY1	H	3.808	DII	hydrophobic/van der Waals
**PZ5**	GLIYHREEKRWRWINNSVFNGNVTN	A	PRO222	CB	GLY1	H	3.778	DII	hydrophobic/van der Waals
**PZ5**	GLIYHREEKRWRWINNSVFNGNVTN	A	PRO222	CG	GLY1	H	2.904	DII	hydrophobic/van der Waals
**PZ5**	GLIYHREEKRWRWINNSVFNGNVTN	A	PRO222	C	GLY1	H	3.991	DII	hydrophobic/van der Waals
**PZ5**	GLIYHREEKRWRWINNSVFNGNVTN	A	PRO222	O	GLY1	H	3.22	DII	hydrophobic/van der Waals
**PZ5**	GLIYHREEKRWRWINNSVFNGNVTN	A	GLN261	CD	GLY1	CA	3.454	DII	H-bond/polar candidate
**PZ5**	GLIYHREEKRWRWINNSVFNGNVTN	A	GLN261	OE1	GLY1	CA	3.403	DII	H-bond/polar candidate
**PZ5**	GLIYHREEKRWRWINNSVFNGNVTN	A	GLN261	NE2	GLY1	CA	3.654	DII	H-bond/polar candidate
**PZ5**	GLIYHREEKRWRWINNSVFNGNVTN	A	GLN261	HE22	GLY1	CA	3.72	DII	H-bond/polar candidate
**PZ5**	GLIYHREEKRWRWINNSVFNGNVTN	B	THR267	CG2	GLY1	CA	3.838	DII	H-bond/polar candidate
**PZ5**	GLIYHREEKRWRWINNSVFNGNVTN	B	THR267	CG2	GLY1	C	3.928	DII	H-bond/polar candidate

**PZ5**	GLIYHREEKRWRWINNSVFNGNVTN	A	PRO222	CG	GLY1	O	3.739	DII	hydrophobic/van der Waals
**PZ5**	GLIYHREEKRWRWINNSVFNGNVTN	B	THR267	CB	LEU2	N	3.892	DII	H-bond/polar candidate
**PZ5**	GLIYHREEKRWRWINNSVFNGNVTN	B	THR267	OG1	LEU2	N	3.37	DII	H-bond/polar candidate
**PZ5**	GLIYHREEKRWRWINNSVFNGNVTN	B	THR267	HG1	LEU2	N	3.484	DII	H-bond/polar candidate
**PZ5**	GLIYHREEKRWRWINNSVFNGNVTN	A	GLN261	OE1	LEU2	H	3.641	DII	H-bond/polar candidate
**PZ5**	GLIYHREEKRWRWINNSVFNGNVTN	A	ALA264	CB	LEU2	H	3.377	DII	hydrophobic/van der Waals
**PZ5**	GLIYHREEKRWRWINNSVFNGNVTN	B	ALA264	CA	LEU2	H	3.957	DII	hydrophobic/van der Waals
**PZ5**	GLIYHREEKRWRWINNSVFNGNVTN	B	THR267	CB	LEU2	H	3.719	DII	H-bond/polar candidate
**PZ5**	GLIYHREEKRWRWINNSVFNGNVTN	B	THR267	OG1	LEU2	H	2.874	DII	H-bond/polar candidate
**PZ5**	GLIYHREEKRWRWINNSVFNGNVTN	B	THR267	HG1	LEU2	H	3.019	DII	H-bond/polar candidate
**PZ5**	GLIYHREEKRWRWINNSVFNGNVTN	B	THR267	CG2	LEU2	H	3.931	DII	H-bond/polar candidate
**PZ5**	GLIYHREEKRWRWINNSVFNGNVTN	B	THR267	OG1	LEU2	CA	3.925	DII	H-bond/polar candidate
**PZ5**	GLIYHREEKRWRWINNSVFNGNVTN	B	THR267	HG1	LEU2	CA	3.713	DII	H-bond/polar candidate
**PZ5**	GLIYHREEKRWRWINNSVFNGNVTN	A	ALA264	CA	LEU2	CD1	3.956	DII	hydrophobic/van der Waals
**PZ5**	GLIYHREEKRWRWINNSVFNGNVTN	A	ALA264	CB	LEU2	CD1	3.531	DII	hydrophobic/van der Waals
**PZ5**	GLIYHREEKRWRWINNSVFNGNVTN	A	ALA264	C	LEU2	CD1	3.57	DII	hydrophobic/van der Waals
**PZ5**	GLIYHREEKRWRWINNSVFNGNVTN	A	ALA264	O	LEU2	CD1	3.144	DII	hydrophobic/van der Waals
**PZ5**	GLIYHREEKRWRWINNSVFNGNVTN	B	THR267	CB	LEU2	C	3.641	DII	H-bond/polar candidate
**PZ5**	GLIYHREEKRWRWINNSVFNGNVTN	B	THR267	OG1	LEU2	C	3.283	DII	H-bond/polar candidate
**PZ5**	GLIYHREEKRWRWINNSVFNGNVTN	B	THR267	HG1	LEU2	C	2.784	DII	H-bond/polar candidate
**PZ5**	GLIYHREEKRWRWINNSVFNGNVTN	B	ALA264	O	LEU2	O	3.351	DII	hydrophobic/van der Waals
**PZ5**	GLIYHREEKRWRWINNSVFNGNVTN	B	THR267	CB	LEU2	O	3.354	DII	H-bond/polar candidate
**PZ5**	GLIYHREEKRWRWINNSVFNGNVTN	B	THR267	OG1	LEU2	O	2.63	DII	H-bond/polar candidate
**PZ5**	GLIYHREEKRWRWINNSVFNGNVTN	B	THR267	HG1	LEU2	O	1.909	DII	H-bond/polar candidate
**PZ5**	GLIYHREEKRWRWINNSVFNGNVTN	B	ALA268	H	LEU2	O	3.835	DII	hydrophobic/van der Waals
**PZ5**	GLIYHREEKRWRWINNSVFNGNVTN	B	THR267	HG1	ILE3	N	3.579	DII	H-bond/polar candidate
**PZ5**	GLIYHREEKRWRWINNSVFNGNVTN	B	THR267	HG1	ILE3	CA	3.736	DII	H-bond/polar candidate
**PZ5**	GLIYHREEKRWRWINNSVFNGNVTN	B	THR267	O	ILE3	CB	3.666	DII	H-bond/polar candidate
**PZ5**	GLIYHREEKRWRWINNSVFNGNVTN	B	THR267	O	ILE3	CG1	3.516	DII	H-bond/polar candidate
**PZ5**	GLIYHREEKRWRWINNSVFNGNVTN	B	ALA268	CA	ILE3	CG1	3.663	DII	hydrophobic/van der Waals

**PZ5**	GLIYHREEKRWRWINNSVFNGNVTN	B	THR267	O	ILE3	CD1	3.647	DII	H-bond/polar candidate
**PZ5**	GLIYHREEKRWRWINNSVFNGNVTN	B	ALA268	CA	TYR4	N	3.89	DII	H-bond/polar candidate; hydrophobic/van der Waals
**PZ5**	GLIYHREEKRWRWINNSVFNGNVTN	B	ALA268	CB	TYR4	N	3.74	DII	H-bond/polar candidate; hydrophobic/van der Waals
**PZ5**	GLIYHREEKRWRWINNSVFNGNVTN	B	ALA268	N	TYR4	H	3.615	DII	H-bond/polar candidate; hydrophobic/van der Waals
**PZ5**	GLIYHREEKRWRWINNSVFNGNVTN	B	ALA268	H	TYR4	H	3.936	DII	H-bond/polar candidate; hydrophobic/van der Waals
**PZ5**	GLIYHREEKRWRWINNSVFNGNVTN	B	ALA268	CA	TYR4	H	2.909	DII	H-bond/polar candidate; hydrophobic/van der Waals
**PZ5**	GLIYHREEKRWRWINNSVFNGNVTN	B	ALA268	CB	TYR4	H	2.784	DII	H-bond/polar candidate; hydrophobic/van der Waals
**PZ5**	GLIYHREEKRWRWINNSVFNGNVTN	B	TRP217	NE1	TYR4	CB	3.871	DII	H-bond/polar candidate; hydrophobic/van der Waals
**PZ5**	GLIYHREEKRWRWINNSVFNGNVTN	B	TRP217	HE1	TYR4	CB	2.942	DII	H-bond/polar candidate; hydrophobic/van der Waals
**PZ5**	GLIYHREEKRWRWINNSVFNGNVTN	B	ALA268	CB	TYR4	CB	3.83	DII	H-bond/polar candidate; hydrophobic/van der Waals
**PZ5**	GLIYHREEKRWRWINNSVFNGNVTN	B	TRP217	NE1	TYR4	CG	3.833	DII	H-bond/polar candidate; hydrophobic/van der Waals
**PZ5**	GLIYHREEKRWRWINNSVFNGNVTN	B	TRP217	HE1	TYR4	CG	3.083	DII	H-bond/polar candidate; hydrophobic/van der Waals
**PZ5**	GLIYHREEKRWRWINNSVFNGNVTN	B	TRP217	CD1	TYR4	CD1	3.564	DII	H-bond/polar candidate; hydrophobic/van der Waals
**PZ5**	GLIYHREEKRWRWINNSVFNGNVTN	B	TRP217	NE1	TYR4	CD1	3.322	DII	H-bond/polar candidate; hydrophobic/van der Waals
**PZ5**	GLIYHREEKRWRWINNSVFNGNVTN	B	TRP217	HE1	TYR4	CD1	2.738	DII	H-bond/polar candidate; hydrophobic/van der Waals
**PZ5**	GLIYHREEKRWRWINNSVFNGNVTN	B	TRP217	CD1	TYR4	CE1	3.895	DII	H-bond/polar candidate; hydrophobic/van der Waals
**PZ5**	GLIYHREEKRWRWINNSVFNGNVTN	B	TRP217	HE1	TYR4	CE1	3.732	DII	H-bond/polar candidate; hydrophobic/van der Waals
**PZ5**	GLIYHREEKRWRWINNSVFNGNVTN	B	ASP220	CB	TYR4	OH	3.446	DII	H-bond/polar candidate
**PZ5**	GLIYHREEKRWRWINNSVFNGNVTN	B	ASP220	CG	TYR4	OH	3.598	DII	H-bond/polar candidate
**PZ5**	GLIYHREEKRWRWINNSVFNGNVTN	B	ASP220	OD1	TYR4	OH	3.907	DII	H-bond/polar candidate
**PZ5**	GLIYHREEKRWRWINNSVFNGNVTN	B	ASP220	OD2	TYR4	OH	3.996	DII	H-bond/polar candidate
**PZ5**	GLIYHREEKRWRWINNSVFNGNVTN	B	ASP220	CB	TYR4	HH	3.256	DII	H-bond/polar candidate
**PZ5**	GLIYHREEKRWRWINNSVFNGNVTN	B	ASP220	CG	TYR4	HH	3.394	DII	H-bond/polar candidate
**PZ5**	GLIYHREEKRWRWINNSVFNGNVTN	B	ASP220	OD1	TYR4	HH	3.455	DII	H-bond/polar candidate
**PZ5**	GLIYHREEKRWRWINNSVFNGNVTN	B	ASP220	C	TYR4	HH	3.8	DII	H-bond/polar candidate
**PZ5**	GLIYHREEKRWRWINNSVFNGNVTN	B	ASP220	O	TYR4	HH	3.222	DII	H-bond/polar candidate
**PZ5**	GLIYHREEKRWRWINNSVFNGNVTN	B	TRP217	HE1	TYR4	O	3.772	DII	H-bond/polar candidate; hydrophobic/van der Waals
**PZ5**	GLIYHREEKRWRWINNSVFNGNVTN	B	GLU216	OE2	ARG6	CD	3.877	DII	salt bridge candidate; H-bond/polar candidate
**PZ5**	GLIYHREEKRWRWINNSVFNGNVTN	B	GLU216	CD	ARG6	NE	3.303	DII	salt bridge candidate; H-bond/polar candidate
**PZ5**	GLIYHREEKRWRWINNSVFNGNVTN	B	GLU216	OE1	ARG6	NE	2.955	DII	salt bridge candidate; H-bond/polar candidate

**PZ5**	GLIYHREEKRWRWINNSVFNGNVTN	B	GLU216	OE2	ARG6	NE	2.906	DII	salt bridge candidate; H-bond/polar candidate
**PZ5**	GLIYHREEKRWRWINNSVFNGNVTN	B	GLU216	CG	ARG6	HE	3.892	DII	salt bridge candidate; H-bond/polar candidate
**PZ5**	GLIYHREEKRWRWINNSVFNGNVTN	B	GLU216	CD	ARG6	HE	2.384	DII	salt bridge candidate; H-bond/polar candidate
**PZ5**	GLIYHREEKRWRWINNSVFNGNVTN	B	GLU216	OE1	ARG6	HE	2.142	DII	salt bridge candidate; H-bond/polar candidate
**PZ5**	GLIYHREEKRWRWINNSVFNGNVTN	B	GLU216	OE2	ARG6	HE	2.029	DII	salt bridge candidate; H-bond/polar candidate
**PZ5**	GLIYHREEKRWRWINNSVFNGNVTN	B	GLU216	CD	ARG6	CZ	3.727	DII	salt bridge candidate; H-bond/polar candidate
**PZ5**	GLIYHREEKRWRWINNSVFNGNVTN	B	GLU216	OE1	ARG6	CZ	3.248	DII	salt bridge candidate; H-bond/polar candidate
**PZ5**	GLIYHREEKRWRWINNSVFNGNVTN	B	GLU216	OE2	ARG6	CZ	3.587	DII	salt bridge candidate; H-bond/polar candidate
**PZ5**	GLIYHREEKRWRWINNSVFNGNVTN	B	GLU216	O	ARG6	CZ	3.542	DII	salt bridge candidate; H-bond/polar candidate
**PZ5**	GLIYHREEKRWRWINNSVFNGNVTN	B	ASP220	CB	ARG6	CZ	3.904	DII	salt bridge candidate; H-bond/polar candidate
**PZ5**	GLIYHREEKRWRWINNSVFNGNVTN	B	ASP220	CG	ARG6	CZ	3.925	DII	salt bridge candidate; H-bond/polar candidate
**PZ5**	GLIYHREEKRWRWINNSVFNGNVTN	B	ASP220	OD2	ARG6	CZ	3.216	DII	salt bridge candidate; H-bond/polar candidate
**PZ5**	GLIYHREEKRWRWINNSVFNGNVTN	B	ASP220	CB	ARG6	NH1	3.31	DII	salt bridge candidate; H-bond/polar candidate
**PZ5**	GLIYHREEKRWRWINNSVFNGNVTN	B	ASP220	CG	ARG6	NH1	3.256	DII	salt bridge candidate; H-bond/polar candidate
**PZ5**	GLIYHREEKRWRWINNSVFNGNVTN	B	ASP220	OD2	ARG6	NH1	2.803	DII	salt bridge candidate; H-bond/polar candidate
**PZ5**	GLIYHREEKRWRWINNSVFNGNVTN	B	ASP220	CB	ARG6	HH11	2.575	DII	salt bridge candidate; H-bond/polar candidate
**PZ5**	GLIYHREEKRWRWINNSVFNGNVTN	B	ASP220	CG	ARG6	HH11	2.282	DII	salt bridge candidate; H-bond/polar candidate
**PZ5**	GLIYHREEKRWRWINNSVFNGNVTN	B	ASP220	OD1	ARG6	HH11	3.19	DII	salt bridge candidate; H-bond/polar candidate
**PZ5**	GLIYHREEKRWRWINNSVFNGNVTN	B	ASP220	OD2	ARG6	HH11	1.925	DII	salt bridge candidate; H-bond/polar candidate
**PZ5**	GLIYHREEKRWRWINNSVFNGNVTN	B	ASP220	CB	ARG6	HH12	3.87	DII	salt bridge candidate; H-bond/polar candidate
**PZ5**	GLIYHREEKRWRWINNSVFNGNVTN	B	ASP220	CG	ARG6	HH12	3.91	DII	salt bridge candidate; H-bond/polar candidate
**PZ5**	GLIYHREEKRWRWINNSVFNGNVTN	B	ASP220	OD2	ARG6	HH12	3.628	DII	salt bridge candidate; H-bond/polar candidate
**PZ5**	GLIYHREEKRWRWINNSVFNGNVTN	B	GLU216	CD	ARG6	NH2	3.236	DII	salt bridge candidate; H-bond/polar candidate
**PZ5**	GLIYHREEKRWRWINNSVFNGNVTN	B	GLU216	OE1	ARG6	NH2	2.709	DII	salt bridge candidate; H-bond/polar candidate
**PZ5**	GLIYHREEKRWRWINNSVFNGNVTN	B	GLU216	OE2	ARG6	NH2	3.463	DII	salt bridge candidate; H-bond/polar candidate
**PZ5**	GLIYHREEKRWRWINNSVFNGNVTN	B	GLU216	C	ARG6	NH2	3.428	DII	salt bridge candidate; H-bond/polar candidate
**PZ5**	GLIYHREEKRWRWINNSVFNGNVTN	B	GLU216	O	ARG6	NH2	2.72	DII	salt bridge candidate; H-bond/polar candidate
**PZ5**	GLIYHREEKRWRWINNSVFNGNVTN	B	ASP220	CB	ARG6	NH2	3.769	DII	salt bridge candidate; H-bond/polar candidate
**PZ5**	GLIYHREEKRWRWINNSVFNGNVTN	B	ASP220	CG	ARG6	NH2	3.663	DII	salt bridge candidate; H-bond/polar candidate
**PZ5**	GLIYHREEKRWRWINNSVFNGNVTN	B	ASP220	OD2	ARG6	NH2	2.782	DII	salt bridge candidate; H-bond/polar candidate

**PZ5**	GLIYHREEKRWRWINNSVFNGNVTN	B	GLU216	OE1	ARG6	HH21	3.498	DII	salt bridge candidate; H-bond/polar candidate
**PZ5**	GLIYHREEKRWRWINNSVFNGNVTN	B	GLU216	C	ARG6	HH21	3.575	DII	salt bridge candidate; H-bond/polar candidate
**PZ5**	GLIYHREEKRWRWINNSVFNGNVTN	B	GLU216	O	ARG6	HH21	2.607	DII	salt bridge candidate; H-bond/polar candidate
**PZ5**	GLIYHREEKRWRWINNSVFNGNVTN	B	ASP220	CB	ARG6	HH21	3.133	DII	salt bridge candidate; H-bond/polar candidate
**PZ5**	GLIYHREEKRWRWINNSVFNGNVTN	B	ASP220	CG	ARG6	HH21	2.845	DII	salt bridge candidate; H-bond/polar candidate
**PZ5**	GLIYHREEKRWRWINNSVFNGNVTN	B	ASP220	OD1	ARG6	HH21	3.978	DII	salt bridge candidate; H-bond/polar candidate
**PZ5**	GLIYHREEKRWRWINNSVFNGNVTN	B	ASP220	OD2	ARG6	HH21	1.935	DII	salt bridge candidate; H-bond/polar candidate
**PZ5**	GLIYHREEKRWRWINNSVFNGNVTN	B	GLU216	CA	ARG6	HH22	3.7	DII	salt bridge candidate; H-bond/polar candidate
**PZ5**	GLIYHREEKRWRWINNSVFNGNVTN	B	GLU216	CB	ARG6	HH22	3.259	DII	salt bridge candidate; H-bond/polar candidate
**PZ5**	GLIYHREEKRWRWINNSVFNGNVTN	B	GLU216	CG	ARG6	HH22	3.329	DII	salt bridge candidate; H-bond/polar candidate
**PZ5**	GLIYHREEKRWRWINNSVFNGNVTN	B	GLU216	CD	ARG6	HH22	2.317	DII	salt bridge candidate; H-bond/polar candidate
**PZ5**	GLIYHREEKRWRWINNSVFNGNVTN	B	GLU216	OE1	ARG6	HH22	1.785	DII	salt bridge candidate; H-bond/polar candidate
**PZ5**	GLIYHREEKRWRWINNSVFNGNVTN	B	GLU216	OE2	ARG6	HH22	2.781	DII	salt bridge candidate; H-bond/polar candidate
**PZ5**	GLIYHREEKRWRWINNSVFNGNVTN	B	GLU216	C	ARG6	HH22	3.09	DII	salt bridge candidate; H-bond/polar candidate
**PZ5**	GLIYHREEKRWRWINNSVFNGNVTN	B	GLU216	O	ARG6	HH22	2.706	DII	salt bridge candidate; H-bond/polar candidate
**PZ5**	GLIYHREEKRWRWINNSVFNGNVTN	B	TRP217	N	ARG6	HH22	3.738	DII	H-bond/polar candidate
**PZ5**	GLIYHREEKRWRWINNSVFNGNVTN	B	ASP220	OD2	ARG6	HH22	3.648	DII	salt bridge candidate; H-bond/polar candidate
**PZ5**	GLIYHREEKRWRWINNSVFNGNVTN	B	ASP220	CG	LYS9	CD	3.933	DII	salt bridge candidate; H-bond/polar candidate
**PZ5**	GLIYHREEKRWRWINNSVFNGNVTN	B	ASP220	OD1	LYS9	CD	3.512	DII	salt bridge candidate; H-bond/polar candidate
**PZ5**	GLIYHREEKRWRWINNSVFNGNVTN	B	ASP220	OD2	LYS9	CD	3.899	DII	salt bridge candidate; H-bond/polar candidate
**PZ5**	GLIYHREEKRWRWINNSVFNGNVTN	B	ASP220	CG	LYS9	CE	3.721	DII	salt bridge candidate; H-bond/polar candidate
**PZ5**	GLIYHREEKRWRWINNSVFNGNVTN	B	ASP220	OD1	LYS9	CE	3.475	DII	salt bridge candidate; H-bond/polar candidate
**PZ5**	GLIYHREEKRWRWINNSVFNGNVTN	B	ASP220	OD2	LYS9	CE	3.282	DII	salt bridge candidate; H-bond/polar candidate
**PZ5**	GLIYHREEKRWRWINNSVFNGNVTN	B	ASP220	CG	LYS9	NZ	2.952	DII	salt bridge candidate; H-bond/polar candidate
**PZ5**	GLIYHREEKRWRWINNSVFNGNVTN	B	ASP220	OD1	LYS9	NZ	2.509	DII	salt bridge candidate; H-bond/polar candidate
**PZ5**	GLIYHREEKRWRWINNSVFNGNVTN	B	ASP220	OD2	LYS9	NZ	2.663	DII	salt bridge candidate; H-bond/polar candidate
**PZ5**	GLIYHREEKRWRWINNSVFNGNVTN	B	ASP220	CB	LYS9	HZ1	3.792	DII	salt bridge candidate; H-bond/polar candidate
**PZ5**	GLIYHREEKRWRWINNSVFNGNVTN	B	ASP220	CG	LYS9	HZ1	2.299	DII	salt bridge candidate; H-bond/polar candidate
**PZ5**	GLIYHREEKRWRWINNSVFNGNVTN	B	ASP220	OD1	LYS9	HZ1	2.253	DII	salt bridge candidate; H-bond/polar candidate
**PZ5**	GLIYHREEKRWRWINNSVFNGNVTN	B	ASP220	OD2	LYS9	HZ1	1.745	DII	salt bridge candidate; H-bond/polar candidate

**PZ5**	GLIYHREEKRWRWINNSVFNGNVTN	B	ASP220	CG	LYS9	HZ2	3.743	DII	salt bridge candidate; H-bond/polar candidate
**PZ5**	GLIYHREEKRWRWINNSVFNGNVTN	B	ASP220	OD1	LYS9	HZ2	3.195	DII	salt bridge candidate; H-bond/polar candidate
**PZ5**	GLIYHREEKRWRWINNSVFNGNVTN	B	ASP220	OD2	LYS9	HZ2	3.433	DII	salt bridge candidate; H-bond/polar candidate
**PZ5**	GLIYHREEKRWRWINNSVFNGNVTN	B	ASP220	CB	LYS9	HZ3	3.98	DII	salt bridge candidate; H-bond/polar candidate
**PZ5**	GLIYHREEKRWRWINNSVFNGNVTN	B	ASP220	CG	LYS9	HZ3	2.575	DII	salt bridge candidate; H-bond/polar candidate
**PZ5**	GLIYHREEKRWRWINNSVFNGNVTN	B	ASP220	OD1	LYS9	HZ3	1.776	DII	salt bridge candidate; H-bond/polar candidate
**PZ5**	GLIYHREEKRWRWINNSVFNGNVTN	B	ASP220	OD2	LYS9	HZ3	2.748	DII	salt bridge candidate; H-bond/polar candidate
**PZ5**	GLIYHREEKRWRWINNSVFNGNVTN	B	ALA264	O	TRP11	CE3	3.997	DII	hydrophobic/van der Waals
**PZ5**	GLIYHREEKRWRWINNSVFNGNVTN	A	THR267	CG2	TRP11	CD1	3.686	DII	H-bond/polar candidate
**PZ5**	GLIYHREEKRWRWINNSVFNGNVTN	B	PRO222	CG	TRP11	HE1	3.367	DII	hydrophobic/van der Waals
**PZ5**	GLIYHREEKRWRWINNSVFNGNVTN	B	ALA264	CB	TRP11	CZ3	3.644	DII	hydrophobic/van der Waals
**PZ5**	GLIYHREEKRWRWINNSVFNGNVTN	B	ALA264	C	TRP11	CZ3	3.55	DII	hydrophobic/van der Waals
**PZ5**	GLIYHREEKRWRWINNSVFNGNVTN	B	ALA264	O	TRP11	CZ3	3.091	DII	hydrophobic/van der Waals
**PZ5**	GLIYHREEKRWRWINNSVFNGNVTN	B	ALA268	CB	TRP11	CZ3	3.821	DII	hydrophobic/van der Waals
**PZ5**	GLIYHREEKRWRWINNSVFNGNVTN	B	ALA264	O	TRP11	CH2	3.924	DII	hydrophobic/van der Waals
**PZ5**	GLIYHREEKRWRWINNSVFNGNVTN	A	PRO222	CD	TRP13	CB	3.784	DII	hydrophobic/van der Waals
**PZ5**	GLIYHREEKRWRWINNSVFNGNVTN	A	PRO222	CD	TRP13	CG	3.841	DII	hydrophobic/van der Waals
**PZ5**	GLIYHREEKRWRWINNSVFNGNVTN	A	PRO222	CD	TRP13	CD2	3.595	DII	hydrophobic/van der Waals
**PZ5**	GLIYHREEKRWRWINNSVFNGNVTN	A	TRP217	CE2	TRP13	CE2	3.908	DII	hydrophobic/van der Waals
**PZ5**	GLIYHREEKRWRWINNSVFNGNVTN	A	TRP217	NE1	TRP13	CE2	3.859	DII	hydrophobic/van der Waals
**PZ5**	GLIYHREEKRWRWINNSVFNGNVTN	A	TRP217	HE1	TRP13	CE2	3.766	DII	hydrophobic/van der Waals
**PZ5**	GLIYHREEKRWRWINNSVFNGNVTN	A	TRP217	CZ2	TRP13	CE2	3.95	DII	hydrophobic/van der Waals
**PZ5**	GLIYHREEKRWRWINNSVFNGNVTN	A	ILE221	CG1	TRP13	CE2	3.994	DII	hydrophobic/van der Waals
**PZ5**	GLIYHREEKRWRWINNSVFNGNVTN	A	PRO222	CD	TRP13	CE3	3.274	DII	hydrophobic/van der Waals
**PZ5**	GLIYHREEKRWRWINNSVFNGNVTN	A	PRO222	CG	TRP13	CE3	3.684	DII	hydrophobic/van der Waals
**PZ5**	GLIYHREEKRWRWINNSVFNGNVTN	A	TRP217	NE1	TRP13	CD1	3.887	DII	hydrophobic/van der Waals
**PZ5**	GLIYHREEKRWRWINNSVFNGNVTN	A	TRP217	HE1	TRP13	CD1	3.777	DII	hydrophobic/van der Waals
**PZ5**	GLIYHREEKRWRWINNSVFNGNVTN	A	TRP217	CE2	TRP13	NE1	3.344	DII	hydrophobic/van der Waals
**PZ5**	GLIYHREEKRWRWINNSVFNGNVTN	A	TRP217	CD1	TRP13	NE1	3.429	DII	hydrophobic/van der Waals
**PZ5**	GLIYHREEKRWRWINNSVFNGNVTN	A	TRP217	NE1	TRP13	NE1	2.946	DII	hydrophobic/van der Waals

**PZ5**	GLIYHREEKRWRWINNSVFNGNVTN	A	TRP217	HE1	TRP13	NE1	2.941	DII	hydrophobic/van der Waals
**PZ5**	GLIYHREEKRWRWINNSVFNGNVTN	A	TRP217	CZ2	TRP13	NE1	3.877	DII	hydrophobic/van der Waals
**PZ5**	GLIYHREEKRWRWINNSVFNGNVTN	A	TRP217	CG	TRP13	HE1	3.124	DII	hydrophobic/van der Waals
**PZ5**	GLIYHREEKRWRWINNSVFNGNVTN	A	TRP217	CD2	TRP13	HE1	3.095	DII	hydrophobic/van der Waals
**PZ5**	GLIYHREEKRWRWINNSVFNGNVTN	A	TRP217	CE2	TRP13	HE1	2.472	DII	hydrophobic/van der Waals
**PZ5**	GLIYHREEKRWRWINNSVFNGNVTN	A	TRP217	CD1	TRP13	HE1	2.517	DII	hydrophobic/van der Waals
**PZ5**	GLIYHREEKRWRWINNSVFNGNVTN	A	TRP217	NE1	TRP13	HE1	2.067	DII	hydrophobic/van der Waals
**PZ5**	GLIYHREEKRWRWINNSVFNGNVTN	A	TRP217	HE1	TRP13	HE1	2.291	DII	hydrophobic/van der Waals
**PZ5**	GLIYHREEKRWRWINNSVFNGNVTN	A	TRP217	CZ2	TRP13	HE1	3.241	DII	hydrophobic/van der Waals
**PZ5**	GLIYHREEKRWRWINNSVFNGNVTN	A	TRP217	CE2	TRP13	CZ2	3.827	DII	hydrophobic/van der Waals
**PZ5**	GLIYHREEKRWRWINNSVFNGNVTN	A	TRP217	CZ2	TRP13	CZ2	3.372	DII	hydrophobic/van der Waals
**PZ5**	GLIYHREEKRWRWINNSVFNGNVTN	A	TRP217	CH2	TRP13	CZ2	3.957	DII	hydrophobic/van der Waals
**PZ5**	GLIYHREEKRWRWINNSVFNGNVTN	A	ILE221	CG1	TRP13	CZ2	3.746	DII	hydrophobic/van der Waals
**PZ5**	GLIYHREEKRWRWINNSVFNGNVTN	A	ILE221	CG2	TRP13	CZ3	3.844	DII	hydrophobic/van der Waals
**PZ5**	GLIYHREEKRWRWINNSVFNGNVTN	A	ILE221	CG2	TRP13	CH2	3.673	DII	hydrophobic/van der Waals
**PZ5**	GLIYHREEKRWRWINNSVFNGNVTN	A	VAL265	CG2	TRP13	CH2	3.791	DII	hydrophobic/van der Waals
**PZ5**	GLIYHREEKRWRWINNSVFNGNVTN	A	PRO222	CG	ILE14	N	3.63	DII	hydrophobic/van der Waals
**PZ5**	GLIYHREEKRWRWINNSVFNGNVTN	A	PRO222	CG	ILE14	H	3.144	DII	hydrophobic/van der Waals
**PZ5**	GLIYHREEKRWRWINNSVFNGNVTN	B	THR267	CB	ILE14	CG2	3.806	DII	H-bond/polar candidate
**PZ5**	GLIYHREEKRWRWINNSVFNGNVTN	B	THR267	CG2	ILE14	CG2	3.837	DII	H-bond/polar candidate
**PZ5**	GLIYHREEKRWRWINNSVFNGNVTN	A	PRO222	CG	ILE14	CG1	3.705	DII	hydrophobic/van der Waals
**PZ5**	GLIYHREEKRWRWINNSVFNGNVTN	B	THR267	CG2	ILE14	CG1	3.993	DII	H-bond/polar candidate
**PZ5**	GLIYHREEKRWRWINNSVFNGNVTN	B	THR267	CG2	ILE14	CD1	3.674	DII	H-bond/polar candidate
**PZ5**	GLIYHREEKRWRWINNSVFNGNVTN	A	PRO222	CG	ILE14	C	3.934	DII	hydrophobic/van der Waals
**PZ5**	GLIYHREEKRWRWINNSVFNGNVTN	A	PRO222	CG	ASN15	N	3.917	DII	H-bond/polar candidate
**PZ5**	GLIYHREEKRWRWINNSVFNGNVTN	A	ASP220	OD2	ASN15	CB	3.539	DII	H-bond/polar candidate
**PZ5**	GLIYHREEKRWRWINNSVFNGNVTN	A	ASP220	O	ASN15	CB	3.826	DII	H-bond/polar candidate
**PZ5**	GLIYHREEKRWRWINNSVFNGNVTN	A	ASP220	OD2	ASN15	CG	3.823	DII	H-bond/polar candidate
**PZ5**	GLIYHREEKRWRWINNSVFNGNVTN	A	ASP220	CG	ASN15	ND2	3.163	DII	H-bond/polar candidate
**PZ5**	GLIYHREEKRWRWINNSVFNGNVTN	A	ASP220	OD1	ASN15	ND2	2.879	DII	H-bond/polar candidate

**PZ5**	GLIYHREEKRWRWINNSVFNGNVTN	A	ASP220	OD2	ASN15	ND2	3.052	DII	H-bond/polar candidate
**PZ5**	GLIYHREEKRWRWINNSVFNGNVTN	A	ASP220	O	ASN15	ND2	3.783	DII	H-bond/polar candidate
**PZ5**	GLIYHREEKRWRWINNSVFNGNVTN	A	ASP220	CA	ASN15	HD21	3.877	DII	H-bond/polar candidate
**PZ5**	GLIYHREEKRWRWINNSVFNGNVTN	A	ASP220	CB	ASN15	HD21	3.565	DII	H-bond/polar candidate
**PZ5**	GLIYHREEKRWRWINNSVFNGNVTN	A	ASP220	CG	ASN15	HD21	2.252	DII	H-bond/polar candidate
**PZ5**	GLIYHREEKRWRWINNSVFNGNVTN	A	ASP220	OD1	ASN15	HD21	2.169	DII	H-bond/polar candidate
**PZ5**	GLIYHREEKRWRWINNSVFNGNVTN	A	ASP220	OD2	ASN15	HD21	2.054	DII	H-bond/polar candidate
**PZ5**	GLIYHREEKRWRWINNSVFNGNVTN	A	ASP220	O	ASN15	HD21	3.425	DII	H-bond/polar candidate
**PZ5**	GLIYHREEKRWRWINNSVFNGNVTN	A	ASP220	CG	ASN15	HD22	3.45	DII	H-bond/polar candidate
**PZ5**	GLIYHREEKRWRWINNSVFNGNVTN	A	ASP220	OD1	ASN15	HD22	2.818	DII	H-bond/polar candidate
**PZ5**	GLIYHREEKRWRWINNSVFNGNVTN	A	ASP220	OD2	ASN15	HD22	3.636	DII	H-bond/polar candidate
**PZ5**	GLIYHREEKRWRWINNSVFNGNVTN	A	ASN238	CG	PHE19	CA	3.919	DII	H-bond/polar candidate
**PZ5**	GLIYHREEKRWRWINNSVFNGNVTN	A	ASN238	OD1	PHE19	CA	3.847	DII	H-bond/polar candidate
**PZ5**	GLIYHREEKRWRWINNSVFNGNVTN	A	ASN238	ND2	PHE19	CA	3.977	DII	H-bond/polar candidate
**PZ5**	GLIYHREEKRWRWINNSVFNGNVTN	A	ASN238	HD22	PHE19	CA	3.888	DII	H-bond/polar candidate
**PZ5**	GLIYHREEKRWRWINNSVFNGNVTN	A	TRP225	HE1	PHE19	CB	3.929	DII	hydrophobic/van der Waals
**PZ5**	GLIYHREEKRWRWINNSVFNGNVTN	A	TRP225	NE1	PHE19	CG	3.918	DII	hydrophobic/van der Waals
**PZ5**	GLIYHREEKRWRWINNSVFNGNVTN	A	TRP225	HE1	PHE19	CG	3.007	DII	hydrophobic/van der Waals
**PZ5**	GLIYHREEKRWRWINNSVFNGNVTN	A	TRP225	HE1	PHE19	CD1	3.424	DII	hydrophobic/van der Waals
**PZ5**	GLIYHREEKRWRWINNSVFNGNVTN	A	TRP225	NE1	PHE19	CD2	3.307	DII	hydrophobic/van der Waals
**PZ5**	GLIYHREEKRWRWINNSVFNGNVTN	A	TRP225	HE1	PHE19	CD2	2.6	DII	hydrophobic/van der Waals
**PZ5**	GLIYHREEKRWRWINNSVFNGNVTN	A	ASN238	CB	PHE19	CD2	3.382	DII	H-bond/polar candidate
**PZ5**	GLIYHREEKRWRWINNSVFNGNVTN	A	ASN238	CG	PHE19	CD2	3.777	DII	H-bond/polar candidate
**PZ5**	GLIYHREEKRWRWINNSVFNGNVTN	A	ASN238	ND2	PHE19	CD2	3.85	DII	H-bond/polar candidate
**PZ5**	GLIYHREEKRWRWINNSVFNGNVTN	A	ASN238	HD21	PHE19	CD2	3.727	DII	H-bond/polar candidate
**PZ5**	GLIYHREEKRWRWINNSVFNGNVTN	A	TRP225	HE1	PHE19	CE1	3.494	DII	hydrophobic/van der Waals
**PZ5**	GLIYHREEKRWRWINNSVFNGNVTN	A	TRP225	CZ2	PHE19	CE1	3.765	DII	hydrophobic/van der Waals
**PZ5**	GLIYHREEKRWRWINNSVFNGNVTN	A	TRP225	CE2	PHE19	CE2	3.585	DII	hydrophobic/van der Waals
**PZ5**	GLIYHREEKRWRWINNSVFNGNVTN	A	TRP225	CD1	PHE19	CE2	3.936	DII	hydrophobic/van der Waals
**PZ5**	GLIYHREEKRWRWINNSVFNGNVTN	A	TRP225	NE1	PHE19	CE2	3.098	DII	hydrophobic/van der Waals

**PZ5**	GLIYHREEKRWRWINNSVFNGNVTN	A	TRP225	HE1	PHE19	CE2	2.698	DII	hydrophobic/van der Waals
**PZ5**	GLIYHREEKRWRWINNSVFNGNVTN	A	TRP225	CZ2	PHE19	CE2	3.857	DII	hydrophobic/van der Waals
**PZ5**	GLIYHREEKRWRWINNSVFNGNVTN	A	ASN237	CB	PHE19	CE2	3.787	DII	H-bond/polar candidate
**PZ5**	GLIYHREEKRWRWINNSVFNGNVTN	A	ASN238	CB	PHE19	CE2	3.726	DII	H-bond/polar candidate
**PZ5**	GLIYHREEKRWRWINNSVFNGNVTN	A	TRP225	CE2	PHE19	CZ	3.607	DII	hydrophobic/van der Waals
**PZ5**	GLIYHREEKRWRWINNSVFNGNVTN	A	TRP225	NE1	PHE19	CZ	3.548	DII	hydrophobic/van der Waals
**PZ5**	GLIYHREEKRWRWINNSVFNGNVTN	A	TRP225	HE1	PHE19	CZ	3.158	DII	hydrophobic/van der Waals
**PZ5**	GLIYHREEKRWRWINNSVFNGNVTN	A	TRP225	CZ2	PHE19	CZ	3.335	DII	hydrophobic/van der Waals
**PZ5**	GLIYHREEKRWRWINNSVFNGNVTN	A	ASN238	OD1	PHE19	C	3.856	DII	H-bond/polar candidate
**PZ5**	GLIYHREEKRWRWINNSVFNGNVTN	A	ASN238	CB	ASN20	N	3.725	DII	H-bond/polar candidate
**PZ5**	GLIYHREEKRWRWINNSVFNGNVTN	A	ASN238	CG	ASN20	N	3.354	DII	H-bond/polar candidate
**PZ5**	GLIYHREEKRWRWINNSVFNGNVTN	A	ASN238	OD1	ASN20	N	2.884	DII	H-bond/polar candidate
**PZ5**	GLIYHREEKRWRWINNSVFNGNVTN	A	ASN238	CB	ASN20	H	3.313	DII	H-bond/polar candidate
**PZ5**	GLIYHREEKRWRWINNSVFNGNVTN	A	ASN238	CG	ASN20	H	2.615	DII	H-bond/polar candidate
**PZ5**	GLIYHREEKRWRWINNSVFNGNVTN	A	ASN238	OD1	ASN20	H	1.962	DII	H-bond/polar candidate
**PZ5**	GLIYHREEKRWRWINNSVFNGNVTN	A	ASN238	ND2	ASN20	H	3.47	DII	H-bond/polar candidate
**PZ5**	GLIYHREEKRWRWINNSVFNGNVTN	A	ASN238	HD22	ASN20	H	3.603	DII	H-bond/polar candidate
**PZ5**	GLIYHREEKRWRWINNSVFNGNVTN	A	ASN238	OD1	ASN20	CA	3.675	DII	H-bond/polar candidate
**PZ5**	GLIYHREEKRWRWINNSVFNGNVTN	A	ASN238	OD1	ASN20	CB	3.524	DII	H-bond/polar candidate
**PZ5**	GLIYHREEKRWRWINNSVFNGNVTN	A	GLU240	OE1	ASN20	CB	3.349	DII	H-bond/polar candidate
**PZ5**	GLIYHREEKRWRWINNSVFNGNVTN	A	GLU240	OE1	ASN20	CG	3.649	DII	H-bond/polar candidate
**PZ5**	GLIYHREEKRWRWINNSVFNGNVTN	A	GLU240	CD	ASN20	ND2	3.439	DII	H-bond/polar candidate
**PZ5**	GLIYHREEKRWRWINNSVFNGNVTN	A	GLU240	OE1	ASN20	ND2	2.938	DII	H-bond/polar candidate
**PZ5**	GLIYHREEKRWRWINNSVFNGNVTN	A	GLU240	OE2	ASN20	ND2	3.857	DII	H-bond/polar candidate
**PZ5**	GLIYHREEKRWRWINNSVFNGNVTN	A	GLU240	H	ASN20	HD21	3.997	DII	H-bond/polar candidate
**PZ5**	GLIYHREEKRWRWINNSVFNGNVTN	A	GLU240	CG	ASN20	HD21	3.443	DII	H-bond/polar candidate
**PZ5**	GLIYHREEKRWRWINNSVFNGNVTN	A	GLU240	CD	ASN20	HD21	2.449	DII	H-bond/polar candidate
**PZ5**	GLIYHREEKRWRWINNSVFNGNVTN	A	GLU240	OE1	ASN20	HD21	1.964	DII	H-bond/polar candidate
**PZ5**	GLIYHREEKRWRWINNSVFNGNVTN	A	GLU240	OE2	ASN20	HD21	2.975	DII	H-bond/polar candidate
**PZ5**	GLIYHREEKRWRWINNSVFNGNVTN	A	GLU240	CD	ASN20	HD22	3.892	DII	H-bond/polar candidate

**PZ5**	GLIYHREEKRWRWINNSVFNGNVTN	A	GLU240	OE1	ASN20	HD22	3.62	DII	H-bond/polar candidate
**PZ5**	GLIYHREEKRWRWINNSVFNGNVTN	A	ASN238	H	ASN20	C	3.844	DII	H-bond/polar candidate
**PZ5**	GLIYHREEKRWRWINNSVFNGNVTN	A	ASN238	CB	ASN20	C	3.922	DII	H-bond/polar candidate
**PZ5**	GLIYHREEKRWRWINNSVFNGNVTN	A	ASN237	HD21	ASN20	O	3.84	DII	H-bond/polar candidate
**PZ5**	GLIYHREEKRWRWINNSVFNGNVTN	A	ASN238	N	ASN20	O	3.175	DII	H-bond/polar candidate
**PZ5**	GLIYHREEKRWRWINNSVFNGNVTN	A	ASN238	H	ASN20	O	2.636	DII	H-bond/polar candidate
**PZ5**	GLIYHREEKRWRWINNSVFNGNVTN	A	ASN238	CA	ASN20	O	3.316	DII	H-bond/polar candidate
**PZ5**	GLIYHREEKRWRWINNSVFNGNVTN	A	ASN238	CB	ASN20	O	3.268	DII	H-bond/polar candidate
**PZ5**	GLIYHREEKRWRWINNSVFNGNVTN	A	ASN237	HD21	GLY21	CA	3.597	DII	H-bond/polar candidate
**PZ5**	GLIYHREEKRWRWINNSVFNGNVTN	A	ASN237	HD21	GLY21	C	3.036	DII	H-bond/polar candidate
**PZ5**	GLIYHREEKRWRWINNSVFNGNVTN	A	ASN237	ND2	GLY21	O	2.938	DII	H-bond/polar candidate
**PZ5**	GLIYHREEKRWRWINNSVFNGNVTN	A	ASN237	HD21	GLY21	O	2.034	DII	H-bond/polar candidate
**PZ5**	GLIYHREEKRWRWINNSVFNGNVTN	A	ASN237	HD22	GLY21	O	3.209	DII	H-bond/polar candidate
**PZ5**	GLIYHREEKRWRWINNSVFNGNVTN	A	ASN237	HD21	VAL23	N	3.788	DII	H-bond/polar candidate
**PZ5**	GLIYHREEKRWRWINNSVFNGNVTN	A	ASN237	ND2	VAL23	H	3.501	DII	H-bond/polar candidate
**PZ5**	GLIYHREEKRWRWINNSVFNGNVTN	A	ASN237	HD21	VAL23	H	2.842	DII	H-bond/polar candidate
**PZ5**	GLIYHREEKRWRWINNSVFNGNVTN	A	ASN237	HD22	VAL23	H	3.7	DII	H-bond/polar candidate
**PZ5**	GLIYHREEKRWRWINNSVFNGNVTN	A	ASN238	H	VAL23	H	3.835	DII	H-bond/polar candidate
**PZ5**	GLIYHREEKRWRWINNSVFNGNVTN	A	HIS235	NE2	VAL23	CB	3.616	DII	H-bond/polar candidate
**PZ5**	GLIYHREEKRWRWINNSVFNGNVTN	A	ASN238	H	VAL23	CB	3.935	DII	H-bond/polar candidate
**PZ5**	GLIYHREEKRWRWINNSVFNGNVTN	A	LYS239	HZ1	VAL23	CB	3.31	DII	H-bond/polar candidate
**PZ5**	GLIYHREEKRWRWINNSVFNGNVTN	A	HIS235	NE2	VAL23	CG1	3.724	DII	H-bond/polar candidate
**PZ5**	GLIYHREEKRWRWINNSVFNGNVTN	A	HIS235	CE1	VAL23	CG1	3.76	DII	H-bond/polar candidate
**PZ5**	GLIYHREEKRWRWINNSVFNGNVTN	A	LYS239	CE	VAL23	CG1	3.743	DII	H-bond/polar candidate
**PZ5**	GLIYHREEKRWRWINNSVFNGNVTN	A	LYS239	NZ	VAL23	CG1	3.538	DII	H-bond/polar candidate
**PZ5**	GLIYHREEKRWRWINNSVFNGNVTN	A	LYS239	HZ1	VAL23	CG1	2.627	DII	H-bond/polar candidate
**PZ5**	GLIYHREEKRWRWINNSVFNGNVTN	A	TRP236	O	VAL23	CG2	3.965	DII	hydrophobic/van der Waals
**PZ5**	GLIYHREEKRWRWINNSVFNGNVTN	A	ASN238	N	VAL23	CG2	3.473	DII	H-bond/polar candidate
**PZ5**	GLIYHREEKRWRWINNSVFNGNVTN	A	ASN238	H	VAL23	CG2	2.828	DII	H-bond/polar candidate
**PZ5**	GLIYHREEKRWRWINNSVFNGNVTN	A	LYS239	H	VAL23	CG2	3.284	DII	H-bond/polar candidate

**PZ5**	GLIYHREEKRWRWINNSVFNGNVTN	A	LYS239	HZ1	VAL23	CG2	3.559	DII	H-bond/polar candidate
**PZ5**	GLIYHREEKRWRWINNSVFNGNVTN	A	GLU240	OE2	VAL23	CG2	3.873	DII	H-bond/polar candidate
**PZ5**	GLIYHREEKRWRWINNSVFNGNVTN	A	HIS235	NE2	VAL23	O	3.614	DII	H-bond/polar candidate
**PZ5**	GLIYHREEKRWRWINNSVFNGNVTN	A	HIS235	CE1	VAL23	O	3.877	DII	H-bond/polar candidate
**PZ5**	GLIYHREEKRWRWINNSVFNGNVTN	A	THR88	CG2	THR24	O	3.91	DII	H-bond/polar candidate
**PZ5**	GLIYHREEKRWRWINNSVFNGNVTN	A	THR88	CG2	ASN25	HD21	3.552	DII	H-bond/polar candidate
**PZ5**	GLIYHREEKRWRWINNSVFNGNVTN	A	HIS235	NE2	ASN25	C	3.986	DII	H-bond/polar candidate
**PZ5**	GLIYHREEKRWRWINNSVFNGNVTN	A	HIS235	CE1	ASN25	C	3.45	DII	H-bond/polar candidate
**PZ5**	GLIYHREEKRWRWINNSVFNGNVTN	A	THR88	CG2	ASN25	O	3.257	DII	H-bond/polar candidate
**PZ5**	GLIYHREEKRWRWINNSVFNGNVTN	A	HIS235	ND1	ASN25	O	3.51	DII	H-bond/polar candidate
**PZ5**	GLIYHREEKRWRWINNSVFNGNVTN	A	HIS235	HD1	ASN25	O	3.485	DII	H-bond/polar candidate
**PZ5**	GLIYHREEKRWRWINNSVFNGNVTN	A	HIS235	CE1	ASN25	O	3.202	DII	H-bond/polar candidate
**PZ6**	MKSRVIRAAWSWRESAAHEGTRDYT	A	LEU273	CD1	MET1	CB	3.892	DII	hydrophobic/van der Waals
**PZ6**	MKSRVIRAAWSWRESAAHEGTRDYT	A	LEU273	CD2	MET1	CG	3.959	DII	hydrophobic/van der Waals
**PZ6**	MKSRVIRAAWSWRESAAHEGTRDYT	A	LEU273	CD2	MET1	SD	3.876	DII	hydrophobic/van der Waals
**PZ6**	MKSRVIRAAWSWRESAAHEGTRDYT	A	ALA270	O	LYS2	CD	3.698	DII	H-bond/polar candidate
**PZ6**	MKSRVIRAAWSWRESAAHEGTRDYT	A	GLY271	O	LYS2	CD	3.692	DII	H-bond/polar candidate
**PZ6**	MKSRVIRAAWSWRESAAHEGTRDYT	A	GLU274	OE1	LYS2	CD	3.932	DII	salt bridge candidate; H-bond/polar candidate
**PZ6**	MKSRVIRAAWSWRESAAHEGTRDYT	A	ALA270	O	LYS2	CE	3.708	DII	H-bond/polar candidate
**PZ6**	MKSRVIRAAWSWRESAAHEGTRDYT	A	GLY271	O	LYS2	CE	3.071	DII	H-bond/polar candidate
**PZ6**	MKSRVIRAAWSWRESAAHEGTRDYT	A	ALA272	O	LYS2	CE	2.911	DII	H-bond/polar candidate
**PZ6**	MKSRVIRAAWSWRESAAHEGTRDYT	A	GLU274	CD	LYS2	CE	3.289	DII	salt bridge candidate; H-bond/polar candidate
**PZ6**	MKSRVIRAAWSWRESAAHEGTRDYT	A	GLU274	OE1	LYS2	CE	3.277	DII	salt bridge candidate; H-bond/polar candidate
**PZ6**	MKSRVIRAAWSWRESAAHEGTRDYT	A	GLU274	OE2	LYS2	CE	3.283	DII	salt bridge candidate; H-bond/polar candidate
**PZ6**	MKSRVIRAAWSWRESAAHEGTRDYT	A	TRP211	HE1	LYS2	NZ	3.781	DII	H-bond/polar candidate
**PZ6**	MKSRVIRAAWSWRESAAHEGTRDYT	A	LEU269	O	LYS2	NZ	3.461	DII	H-bond/polar candidate
**PZ6**	MKSRVIRAAWSWRESAAHEGTRDYT	A	ALA270	C	LYS2	NZ	3.758	DII	H-bond/polar candidate
**PZ6**	MKSRVIRAAWSWRESAAHEGTRDYT	A	ALA270	O	LYS2	NZ	2.896	DII	H-bond/polar candidate
**PZ6**	MKSRVIRAAWSWRESAAHEGTRDYT	A	GLY271	C	LYS2	NZ	3.616	DII	H-bond/polar candidate
**PZ6**	MKSRVIRAAWSWRESAAHEGTRDYT	A	GLY271	O	LYS2	NZ	2.91	DII	H-bond/polar candidate

**PZ6**	MKSRVIRAAWSWRESAAHEGTRDYT	A	ALA272	C	LYS2	NZ	3.779	DII	H-bond/polar candidate
**PZ6**	MKSRVIRAAWSWRESAAHEGTRDYT	A	ALA272	O	LYS2	NZ	2.68	DII	H-bond/polar candidate
**PZ6**	MKSRVIRAAWSWRESAAHEGTRDYT	A	GLU274	CD	LYS2	NZ	3.341	DII	salt bridge candidate; H-bond/polar candidate
**PZ6**	MKSRVIRAAWSWRESAAHEGTRDYT	A	GLU274	OE1	LYS2	NZ	3.765	DII	salt bridge candidate; H-bond/polar candidate
**PZ6**	MKSRVIRAAWSWRESAAHEGTRDYT	A	GLU274	OE2	LYS2	NZ	2.847	DII	salt bridge candidate; H-bond/polar candidate
**PZ6**	MKSRVIRAAWSWRESAAHEGTRDYT	A	TRP211	HE1	LYS2	HZ1	3.607	DII	H-bond/polar candidate
**PZ6**	MKSRVIRAAWSWRESAAHEGTRDYT	A	LEU269	O	LYS2	HZ1	3.159	DII	H-bond/polar candidate
**PZ6**	MKSRVIRAAWSWRESAAHEGTRDYT	A	ALA270	C	LYS2	HZ1	3.9	DII	H-bond/polar candidate
**PZ6**	MKSRVIRAAWSWRESAAHEGTRDYT	A	ALA270	O	LYS2	HZ1	3.285	DII	H-bond/polar candidate
**PZ6**	MKSRVIRAAWSWRESAAHEGTRDYT	A	GLY271	C	LYS2	HZ1	3.115	DII	H-bond/polar candidate
**PZ6**	MKSRVIRAAWSWRESAAHEGTRDYT	A	GLY271	O	LYS2	HZ1	2.516	DII	H-bond/polar candidate
**PZ6**	MKSRVIRAAWSWRESAAHEGTRDYT	A	ALA272	N	LYS2	HZ1	3.457	DII	H-bond/polar candidate
**PZ6**	MKSRVIRAAWSWRESAAHEGTRDYT	A	ALA272	CA	LYS2	HZ1	3.371	DII	H-bond/polar candidate
**PZ6**	MKSRVIRAAWSWRESAAHEGTRDYT	A	ALA272	CB	LYS2	HZ1	3.688	DII	H-bond/polar candidate
**PZ6**	MKSRVIRAAWSWRESAAHEGTRDYT	A	ALA272	C	LYS2	HZ1	2.795	DII	H-bond/polar candidate
**PZ6**	MKSRVIRAAWSWRESAAHEGTRDYT	A	ALA272	O	LYS2	HZ1	1.742	DII	H-bond/polar candidate
**PZ6**	MKSRVIRAAWSWRESAAHEGTRDYT	A	LEU273	N	LYS2	HZ1	3.975	DII	H-bond/polar candidate
**PZ6**	MKSRVIRAAWSWRESAAHEGTRDYT	A	GLU274	CG	LYS2	HZ1	3.971	DII	salt bridge candidate; H-bond/polar candidate
**PZ6**	MKSRVIRAAWSWRESAAHEGTRDYT	A	GLU274	CD	LYS2	HZ1	3.59	DII	salt bridge candidate; H-bond/polar candidate
**PZ6**	MKSRVIRAAWSWRESAAHEGTRDYT	A	GLU274	OE2	LYS2	HZ1	3.214	DII	salt bridge candidate; H-bond/polar candidate
**PZ6**	MKSRVIRAAWSWRESAAHEGTRDYT	A	LEU269	O	LYS2	HZ2	3.13	DII	H-bond/polar candidate
**PZ6**	MKSRVIRAAWSWRESAAHEGTRDYT	A	ALA270	CA	LYS2	HZ2	3.68	DII	H-bond/polar candidate
**PZ6**	MKSRVIRAAWSWRESAAHEGTRDYT	A	ALA270	C	LYS2	HZ2	2.797	DII	H-bond/polar candidate
**PZ6**	MKSRVIRAAWSWRESAAHEGTRDYT	A	ALA270	O	LYS2	HZ2	1.865	DII	H-bond/polar candidate
**PZ6**	MKSRVIRAAWSWRESAAHEGTRDYT	A	GLY271	N	LYS2	HZ2	3.648	DII	H-bond/polar candidate
**PZ6**	MKSRVIRAAWSWRESAAHEGTRDYT	A	GLY271	CA	LYS2	HZ2	3.801	DII	H-bond/polar candidate
**PZ6**	MKSRVIRAAWSWRESAAHEGTRDYT	A	GLY271	C	LYS2	HZ2	3.246	DII	H-bond/polar candidate
**PZ6**	MKSRVIRAAWSWRESAAHEGTRDYT	A	GLY271	O	LYS2	HZ2	2.726	DII	H-bond/polar candidate
**PZ6**	MKSRVIRAAWSWRESAAHEGTRDYT	A	ALA272	O	LYS2	HZ2	3.314	DII	H-bond/polar candidate
**PZ6**	MKSRVIRAAWSWRESAAHEGTRDYT	A	GLU274	OE2	LYS2	HZ2	3.638	DII	salt bridge candidate; H-bond/polar candidate

**PZ6**	MKSRVIRAAWSWRESAAHEGTRDYT	A	TRP211	NE1	LYS2	HZ3	3.842	DII	H-bond/polar candidate
**PZ6**	MKSRVIRAAWSWRESAAHEGTRDYT	A	TRP211	HE1	LYS2	HZ3	3.012	DII	H-bond/polar candidate
**PZ6**	MKSRVIRAAWSWRESAAHEGTRDYT	A	LEU269	O	LYS2	HZ3	3.57	DII	H-bond/polar candidate
**PZ6**	MKSRVIRAAWSWRESAAHEGTRDYT	A	ALA270	O	LYS2	HZ3	3.53	DII	H-bond/polar candidate
**PZ6**	MKSRVIRAAWSWRESAAHEGTRDYT	A	GLY271	O	LYS2	HZ3	3.928	DII	H-bond/polar candidate
**PZ6**	MKSRVIRAAWSWRESAAHEGTRDYT	A	ALA272	O	LYS2	HZ3	3.259	DII	H-bond/polar candidate
**PZ6**	MKSRVIRAAWSWRESAAHEGTRDYT	A	GLU274	CG	LYS2	HZ3	3.578	DII	salt bridge candidate; H-bond/polar candidate
**PZ6**	MKSRVIRAAWSWRESAAHEGTRDYT	A	GLU274	CD	LYS2	HZ3	2.636	DII	salt bridge candidate; H-bond/polar candidate
**PZ6**	MKSRVIRAAWSWRESAAHEGTRDYT	A	GLU274	OE1	LYS2	HZ3	3.204	DII	salt bridge candidate; H-bond/polar candidate
**PZ6**	MKSRVIRAAWSWRESAAHEGTRDYT	A	GLU274	OE2	LYS2	HZ3	1.895	DII	salt bridge candidate; H-bond/polar candidate
**PZ6**	MKSRVIRAAWSWRESAAHEGTRDYT	B	LYS246	NZ	LYS2	HZ3	3.898	DII	H-bond/polar candidate
**PZ6**	MKSRVIRAAWSWRESAAHEGTRDYT	B	LYS246	HZ1	LYS2	HZ3	3.936	DII	H-bond/polar candidate
**PZ6**	MKSRVIRAAWSWRESAAHEGTRDYT	B	LYS246	HZ2	LYS2	HZ3	3.206	DII	H-bond/polar candidate
**PZ6**	MKSRVIRAAWSWRESAAHEGTRDYT	B	LYS246	HZ3	LYS2	HZ3	3.902	DII	H-bond/polar candidate
**PZ6**	MKSRVIRAAWSWRESAAHEGTRDYT	A	SER286	OG	ARG4	CB	3.837	DI	H-bond/polar candidate
**PZ6**	MKSRVIRAAWSWRESAAHEGTRDYT	A	SER286	OG	ARG4	CD	3.57	DI	H-bond/polar candidate
**PZ6**	MKSRVIRAAWSWRESAAHEGTRDYT	A	SER286	HG	ARG4	CD	3.936	DI	H-bond/polar candidate
**PZ6**	MKSRVIRAAWSWRESAAHEGTRDYT	A	GLU26	CD	ARG4	NE	3.989	DI	salt bridge candidate; H-bond/polar candidate
**PZ6**	MKSRVIRAAWSWRESAAHEGTRDYT	A	GLU26	OE1	ARG4	NE	2.999	DI	salt bridge candidate; H-bond/polar candidate
**PZ6**	MKSRVIRAAWSWRESAAHEGTRDYT	A	GLU26	CD	ARG4	HE	3.293	DI	salt bridge candidate; H-bond/polar candidate
**PZ6**	MKSRVIRAAWSWRESAAHEGTRDYT	A	GLU26	OE1	ARG4	HE	2.201	DI	salt bridge candidate; H-bond/polar candidate
**PZ6**	MKSRVIRAAWSWRESAAHEGTRDYT	A	GLU26	OE2	ARG4	HE	3.974	DI	salt bridge candidate; H-bond/polar candidate
**PZ6**	MKSRVIRAAWSWRESAAHEGTRDYT	A	GLU26	CD	ARG4	CZ	3.898	DI	salt bridge candidate; H-bond/polar candidate
**PZ6**	MKSRVIRAAWSWRESAAHEGTRDYT	A	GLU26	OE1	ARG4	CZ	3.236	DI	salt bridge candidate; H-bond/polar candidate
**PZ6**	MKSRVIRAAWSWRESAAHEGTRDYT	A	SER286	OG	ARG4	CZ	3.732	DI	H-bond/polar candidate
**PZ6**	MKSRVIRAAWSWRESAAHEGTRDYT	A	GLY287	O	ARG4	CZ	3.117	DI	H-bond/polar candidate
**PZ6**	MKSRVIRAAWSWRESAAHEGTRDYT	A	SER286	CA	ARG4	NH1	3.753	DI	H-bond/polar candidate
**PZ6**	MKSRVIRAAWSWRESAAHEGTRDYT	A	SER286	CB	ARG4	NH1	3.746	DI	H-bond/polar candidate
**PZ6**	MKSRVIRAAWSWRESAAHEGTRDYT	A	SER286	OG	ARG4	NH1	2.681	DI	H-bond/polar candidate
**PZ6**	MKSRVIRAAWSWRESAAHEGTRDYT	A	SER286	HG	ARG4	NH1	3.277	DI	H-bond/polar candidate

**PZ6**	MKSRVIRAAWSWRESAAHEGTRDYT	A	SER286	C	ARG4	NH1	3.487	DI	H-bond/polar candidate
**PZ6**	MKSRVIRAAWSWRESAAHEGTRDYT	A	SER286	O	ARG4	NH1	3.808	DI	H-bond/polar candidate
**PZ6**	MKSRVIRAAWSWRESAAHEGTRDYT	A	GLY287	N	ARG4	NH1	3.685	DI	H-bond/polar candidate
**PZ6**	MKSRVIRAAWSWRESAAHEGTRDYT	A	GLY287	H	ARG4	NH1	3.93	DI	H-bond/polar candidate
**PZ6**	MKSRVIRAAWSWRESAAHEGTRDYT	A	GLY287	C	ARG4	NH1	3.715	DI	H-bond/polar candidate
**PZ6**	MKSRVIRAAWSWRESAAHEGTRDYT	A	GLY287	O	ARG4	NH1	2.747	DI	H-bond/polar candidate
**PZ6**	MKSRVIRAAWSWRESAAHEGTRDYT	A	SER286	CA	ARG4	HH11	3.761	DI	H-bond/polar candidate
**PZ6**	MKSRVIRAAWSWRESAAHEGTRDYT	A	SER286	CB	ARG4	HH11	3.953	DI	H-bond/polar candidate
**PZ6**	MKSRVIRAAWSWRESAAHEGTRDYT	A	SER286	OG	ARG4	HH11	3.077	DI	H-bond/polar candidate
**PZ6**	MKSRVIRAAWSWRESAAHEGTRDYT	A	SER286	HG	ARG4	HH11	3.624	DI	H-bond/polar candidate
**PZ6**	MKSRVIRAAWSWRESAAHEGTRDYT	A	SER286	C	ARG4	HH11	3.087	DI	H-bond/polar candidate
**PZ6**	MKSRVIRAAWSWRESAAHEGTRDYT	A	SER286	O	ARG4	HH11	3.261	DI	H-bond/polar candidate
**PZ6**	MKSRVIRAAWSWRESAAHEGTRDYT	A	GLY287	N	ARG4	HH11	3.167	DI	H-bond/polar candidate
**PZ6**	MKSRVIRAAWSWRESAAHEGTRDYT	A	GLY287	H	ARG4	HH11	3.609	DI	H-bond/polar candidate
**PZ6**	MKSRVIRAAWSWRESAAHEGTRDYT	A	GLY287	CA	ARG4	HH11	3.506	DI	H-bond/polar candidate
**PZ6**	MKSRVIRAAWSWRESAAHEGTRDYT	A	GLY287	C	ARG4	HH11	2.801	DI	H-bond/polar candidate
**PZ6**	MKSRVIRAAWSWRESAAHEGTRDYT	A	GLY287	O	ARG4	HH11	1.936	DI	H-bond/polar candidate
**PZ6**	MKSRVIRAAWSWRESAAHEGTRDYT	A	HIS288	N	ARG4	HH11	3.759	DI	H-bond/polar candidate
**PZ6**	MKSRVIRAAWSWRESAAHEGTRDYT	A	HIS288	CB	ARG4	HH11	3.724	DI	H-bond/polar candidate
**PZ6**	MKSRVIRAAWSWRESAAHEGTRDYT	A	SER286	CA	ARG4	HH12	3.381	DI	H-bond/polar candidate
**PZ6**	MKSRVIRAAWSWRESAAHEGTRDYT	A	SER286	CB	ARG4	HH12	3.015	DI	H-bond/polar candidate
**PZ6**	MKSRVIRAAWSWRESAAHEGTRDYT	A	SER286	OG	ARG4	HH12	1.778	DI	H-bond/polar candidate
**PZ6**	MKSRVIRAAWSWRESAAHEGTRDYT	A	SER286	HG	ARG4	HH12	2.333	DI	H-bond/polar candidate
**PZ6**	MKSRVIRAAWSWRESAAHEGTRDYT	A	SER286	C	ARG4	HH12	3.415	DI	H-bond/polar candidate
**PZ6**	MKSRVIRAAWSWRESAAHEGTRDYT	A	SER286	O	ARG4	HH12	3.668	DI	H-bond/polar candidate
**PZ6**	MKSRVIRAAWSWRESAAHEGTRDYT	A	GLY287	N	ARG4	HH12	3.943	DI	H-bond/polar candidate
**PZ6**	MKSRVIRAAWSWRESAAHEGTRDYT	A	GLY287	O	ARG4	HH12	3.588	DI	H-bond/polar candidate
**PZ6**	MKSRVIRAAWSWRESAAHEGTRDYT	A	GLU26	CB	ARG4	NH2	3.877	DI	salt bridge candidate; H-bond/polar candidate
**PZ6**	MKSRVIRAAWSWRESAAHEGTRDYT	A	GLU26	CG	ARG4	NH2	3.276	DI	salt bridge candidate; H-bond/polar candidate
**PZ6**	MKSRVIRAAWSWRESAAHEGTRDYT	A	GLU26	CD	ARG4	NH2	2.982	DI	salt bridge candidate; H-bond/polar candidate

**PZ6**	MKSRVIRAAWSWRESAAHEGTRDYT	A	GLU26	OE1	ARG4	NH2	2.71	DI	salt bridge candidate; H-bond/polar candidate
**PZ6**	MKSRVIRAAWSWRESAAHEGTRDYT	A	GLU26	OE2	ARG4	NH2	3.644	DI	salt bridge candidate; H-bond/polar candidate
**PZ6**	MKSRVIRAAWSWRESAAHEGTRDYT	A	HIS27	H	ARG4	NH2	3.991	DI	H-bond/polar candidate
**PZ6**	MKSRVIRAAWSWRESAAHEGTRDYT	A	GLY287	C	ARG4	NH2	3.892	DI	H-bond/polar candidate
**PZ6**	MKSRVIRAAWSWRESAAHEGTRDYT	A	GLY287	O	ARG4	NH2	2.669	DI	H-bond/polar candidate
**PZ6**	MKSRVIRAAWSWRESAAHEGTRDYT	A	GLU26	CA	ARG4	HH21	3.942	DI	salt bridge candidate; H-bond/polar candidate
**PZ6**	MKSRVIRAAWSWRESAAHEGTRDYT	A	GLU26	CB	ARG4	HH21	3.635	DI	salt bridge candidate; H-bond/polar candidate
**PZ6**	MKSRVIRAAWSWRESAAHEGTRDYT	A	GLU26	CG	ARG4	HH21	3.376	DI	salt bridge candidate; H-bond/polar candidate
**PZ6**	MKSRVIRAAWSWRESAAHEGTRDYT	A	GLU26	CD	ARG4	HH21	3.439	DI	salt bridge candidate; H-bond/polar candidate
**PZ6**	MKSRVIRAAWSWRESAAHEGTRDYT	A	GLU26	OE1	ARG4	HH21	3.462	DI	salt bridge candidate; H-bond/polar candidate
**PZ6**	MKSRVIRAAWSWRESAAHEGTRDYT	A	HIS27	H	ARG4	HH21	3.584	DI	H-bond/polar candidate
**PZ6**	MKSRVIRAAWSWRESAAHEGTRDYT	A	GLY287	C	ARG4	HH21	3.034	DI	H-bond/polar candidate
**PZ6**	MKSRVIRAAWSWRESAAHEGTRDYT	A	GLY287	O	ARG4	HH21	1.837	DI	H-bond/polar candidate
**PZ6**	MKSRVIRAAWSWRESAAHEGTRDYT	A	HIS288	N	ARG4	HH21	3.827	DI	H-bond/polar candidate
**PZ6**	MKSRVIRAAWSWRESAAHEGTRDYT	A	HIS288	CA	ARG4	HH21	3.795	DI	H-bond/polar candidate
**PZ6**	MKSRVIRAAWSWRESAAHEGTRDYT	A	HIS288	CB	ARG4	HH21	3.787	DI	H-bond/polar candidate
**PZ6**	MKSRVIRAAWSWRESAAHEGTRDYT	A	GLU26	CB	ARG4	HH22	3.402	DI	salt bridge candidate; H-bond/polar candidate
**PZ6**	MKSRVIRAAWSWRESAAHEGTRDYT	A	GLU26	CG	ARG4	HH22	2.703	DI	salt bridge candidate; H-bond/polar candidate
**PZ6**	MKSRVIRAAWSWRESAAHEGTRDYT	A	GLU26	CD	ARG4	HH22	2.067	DI	salt bridge candidate; H-bond/polar candidate
**PZ6**	MKSRVIRAAWSWRESAAHEGTRDYT	A	GLU26	OE1	ARG4	HH22	1.86	DI	salt bridge candidate; H-bond/polar candidate
**PZ6**	MKSRVIRAAWSWRESAAHEGTRDYT	A	GLU26	OE2	ARG4	HH22	2.674	DI	salt bridge candidate; H-bond/polar candidate
**PZ6**	MKSRVIRAAWSWRESAAHEGTRDYT	A	GLY287	O	ARG4	HH22	3.514	DI	H-bond/polar candidate
**PZ6**	MKSRVIRAAWSWRESAAHEGTRDYT	A	SER285	HG	VAL5	CG1	3.992	DII	H-bond/polar candidate
**PZ6**	MKSRVIRAAWSWRESAAHEGTRDYT	A	GLU274	H	VAL5	CG2	3.516	DII	H-bond/polar candidate
**PZ6**	MKSRVIRAAWSWRESAAHEGTRDYT	A	SER285	OG	VAL5	CG2	3.984	DII	H-bond/polar candidate
**PZ6**	MKSRVIRAAWSWRESAAHEGTRDYT	A	SER285	HG	VAL5	CG2	3.93	DII	H-bond/polar candidate
**PZ6**	MKSRVIRAAWSWRESAAHEGTRDYT	B	ASP247	OD1	ILE6	H	3.964	DII	H-bond/polar candidate
**PZ6**	MKSRVIRAAWSWRESAAHEGTRDYT	B	ASP247	CB	ILE6	CA	3.666	DII	H-bond/polar candidate
**PZ6**	MKSRVIRAAWSWRESAAHEGTRDYT	B	ASP247	CG	ILE6	CA	3.969	DII	H-bond/polar candidate
**PZ6**	MKSRVIRAAWSWRESAAHEGTRDYT	B	ASP247	OD1	ILE6	CA	3.874	DII	H-bond/polar candidate

**PZ6**	MKSRVIRAAWSWRESAAHEGTRDYT	B	ASP247	CA	ILE6	CG2	3.954	DII	H-bond/polar candidate
**PZ6**	MKSRVIRAAWSWRESAAHEGTRDYT	B	ASP247	O	ILE6	CG2	3.484	DII	H-bond/polar candidate
**PZ6**	MKSRVIRAAWSWRESAAHEGTRDYT	B	ASP247	OD1	ILE6	CG1	3.277	DII	H-bond/polar candidate
**PZ6**	MKSRVIRAAWSWRESAAHEGTRDYT	A	ASN8	CB	ARG7	NE	3.937	DI	H-bond/polar candidate
**PZ6**	MKSRVIRAAWSWRESAAHEGTRDYT	A	ASN8	CG	ARG7	NE	3.491	DI	H-bond/polar candidate
**PZ6**	MKSRVIRAAWSWRESAAHEGTRDYT	A	ASN8	OD1	ARG7	NE	2.823	DI	H-bond/polar candidate
**PZ6**	MKSRVIRAAWSWRESAAHEGTRDYT	A	ASN8	CB	ARG7	HE	3.478	DI	H-bond/polar candidate
**PZ6**	MKSRVIRAAWSWRESAAHEGTRDYT	A	ASN8	CG	ARG7	HE	2.711	DI	H-bond/polar candidate
**PZ6**	MKSRVIRAAWSWRESAAHEGTRDYT	A	ASN8	OD1	ARG7	HE	1.996	DI	H-bond/polar candidate
**PZ6**	MKSRVIRAAWSWRESAAHEGTRDYT	A	ASN8	ND2	ARG7	HE	3.51	DI	H-bond/polar candidate
**PZ6**	MKSRVIRAAWSWRESAAHEGTRDYT	A	ASN8	HD22	ARG7	HE	3.648	DI	H-bond/polar candidate
**PZ6**	MKSRVIRAAWSWRESAAHEGTRDYT	A	SER7	CB	ARG7	CZ	3.954	DI	H-bond/polar candidate
**PZ6**	MKSRVIRAAWSWRESAAHEGTRDYT	A	SER7	OG	ARG7	CZ	3.253	DI	H-bond/polar candidate
**PZ6**	MKSRVIRAAWSWRESAAHEGTRDYT	A	ASN8	CB	ARG7	CZ	3.982	DI	H-bond/polar candidate
**PZ6**	MKSRVIRAAWSWRESAAHEGTRDYT	A	ASN8	CG	ARG7	CZ	3.877	DI	H-bond/polar candidate
**PZ6**	MKSRVIRAAWSWRESAAHEGTRDYT	A	ASN8	OD1	ARG7	CZ	3.154	DI	H-bond/polar candidate
**PZ6**	MKSRVIRAAWSWRESAAHEGTRDYT	A	SER7	CB	ARG7	NH1	3.525	DI	H-bond/polar candidate
**PZ6**	MKSRVIRAAWSWRESAAHEGTRDYT	A	SER7	OG	ARG7	NH1	2.907	DI	H-bond/polar candidate
**PZ6**	MKSRVIRAAWSWRESAAHEGTRDYT	A	SER7	HG	ARG7	NH1	3.75	DI	H-bond/polar candidate
**PZ6**	MKSRVIRAAWSWRESAAHEGTRDYT	A	SER7	CB	ARG7	HH11	2.759	DI	H-bond/polar candidate
**PZ6**	MKSRVIRAAWSWRESAAHEGTRDYT	A	SER7	OG	ARG7	HH11	2.071	DI	H-bond/polar candidate
**PZ6**	MKSRVIRAAWSWRESAAHEGTRDYT	A	SER7	HG	ARG7	HH11	2.865	DI	H-bond/polar candidate
**PZ6**	MKSRVIRAAWSWRESAAHEGTRDYT	A	SER7	OG	ARG7	HH12	3.775	DI	H-bond/polar candidate
**PZ6**	MKSRVIRAAWSWRESAAHEGTRDYT	A	SER7	CB	ARG7	NH2	3.596	DI	H-bond/polar candidate
**PZ6**	MKSRVIRAAWSWRESAAHEGTRDYT	A	SER7	OG	ARG7	NH2	2.753	DI	H-bond/polar candidate
**PZ6**	MKSRVIRAAWSWRESAAHEGTRDYT	A	SER7	HG	ARG7	NH2	3.461	DI	H-bond/polar candidate
**PZ6**	MKSRVIRAAWSWRESAAHEGTRDYT	A	SER7	C	ARG7	NH2	3.443	DI	H-bond/polar candidate
**PZ6**	MKSRVIRAAWSWRESAAHEGTRDYT	A	SER7	O	ARG7	NH2	3.714	DI	H-bond/polar candidate
**PZ6**	MKSRVIRAAWSWRESAAHEGTRDYT	A	ASN8	N	ARG7	NH2	3.286	DI	H-bond/polar candidate
**PZ6**	MKSRVIRAAWSWRESAAHEGTRDYT	A	ASN8	H	ARG7	NH2	3.724	DI	H-bond/polar candidate

**PZ6**	MKSRVIRAAWSWRESAAHEGTRDYT	A	ASN8	CA	ARG7	NH2	3.444	DI	H-bond/polar candidate
**PZ6**	MKSRVIRAAWSWRESAAHEGTRDYT	A	ASN8	CB	ARG7	NH2	3.565	DI	H-bond/polar candidate
**PZ6**	MKSRVIRAAWSWRESAAHEGTRDYT	A	ASN8	CG	ARG7	NH2	3.483	DI	H-bond/polar candidate
**PZ6**	MKSRVIRAAWSWRESAAHEGTRDYT	A	ASN8	OD1	ARG7	NH2	2.672	DI	H-bond/polar candidate
**PZ6**	MKSRVIRAAWSWRESAAHEGTRDYT	A	SER7	CA	ARG7	HH21	3.414	DI	H-bond/polar candidate
**PZ6**	MKSRVIRAAWSWRESAAHEGTRDYT	A	SER7	CB	ARG7	HH21	2.875	DI	H-bond/polar candidate
**PZ6**	MKSRVIRAAWSWRESAAHEGTRDYT	A	SER7	OG	ARG7	HH21	1.87	DI	H-bond/polar candidate
**PZ6**	MKSRVIRAAWSWRESAAHEGTRDYT	A	SER7	HG	ARG7	HH21	2.488	DI	H-bond/polar candidate
**PZ6**	MKSRVIRAAWSWRESAAHEGTRDYT	A	SER7	C	ARG7	HH21	2.899	DI	H-bond/polar candidate
**PZ6**	MKSRVIRAAWSWRESAAHEGTRDYT	A	SER7	O	ARG7	HH21	3.105	DI	H-bond/polar candidate
**PZ6**	MKSRVIRAAWSWRESAAHEGTRDYT	A	ASN8	N	ARG7	HH21	3.123	DI	H-bond/polar candidate
**PZ6**	MKSRVIRAAWSWRESAAHEGTRDYT	A	ASN8	H	ARG7	HH21	3.625	DI	H-bond/polar candidate
**PZ6**	MKSRVIRAAWSWRESAAHEGTRDYT	A	ASN8	CA	ARG7	HH21	3.583	DI	H-bond/polar candidate
**PZ6**	MKSRVIRAAWSWRESAAHEGTRDYT	A	ASN8	OD1	ARG7	HH21	3.482	DI	H-bond/polar candidate
**PZ6**	MKSRVIRAAWSWRESAAHEGTRDYT	A	SER7	OG	ARG7	HH22	3.585	DI	H-bond/polar candidate
**PZ6**	MKSRVIRAAWSWRESAAHEGTRDYT	A	SER7	C	ARG7	HH22	3.615	DI	H-bond/polar candidate
**PZ6**	MKSRVIRAAWSWRESAAHEGTRDYT	A	SER7	O	ARG7	HH22	3.699	DI	H-bond/polar candidate
**PZ6**	MKSRVIRAAWSWRESAAHEGTRDYT	A	ASN8	N	ARG7	HH22	3.309	DI	H-bond/polar candidate
**PZ6**	MKSRVIRAAWSWRESAAHEGTRDYT	A	ASN8	H	ARG7	HH22	3.89	DI	H-bond/polar candidate
**PZ6**	MKSRVIRAAWSWRESAAHEGTRDYT	A	ASN8	CA	ARG7	HH22	3.035	DI	H-bond/polar candidate
**PZ6**	MKSRVIRAAWSWRESAAHEGTRDYT	A	ASN8	CB	ARG7	HH22	3.12	DI	H-bond/polar candidate
**PZ6**	MKSRVIRAAWSWRESAAHEGTRDYT	A	ASN8	CG	ARG7	HH22	2.739	DI	H-bond/polar candidate
**PZ6**	MKSRVIRAAWSWRESAAHEGTRDYT	A	ASN8	OD1	ARG7	HH22	1.798	DI	H-bond/polar candidate
**PZ6**	MKSRVIRAAWSWRESAAHEGTRDYT	A	ASN8	ND2	ARG7	HH22	3.989	DI	H-bond/polar candidate
**PZ6**	MKSRVIRAAWSWRESAAHEGTRDYT	A	HIS27	CB	ALA8	CB	3.917	DI	H-bond/polar candidate
**PZ6**	MKSRVIRAAWSWRESAAHEGTRDYT	A	HIS27	HD1	ALA8	CB	3.711	DI	H-bond/polar candidate
**PZ6**	MKSRVIRAAWSWRESAAHEGTRDYT	A	SER285	O	ALA8	CB	3.652	DII	H-bond/polar candidate
**PZ6**	MKSRVIRAAWSWRESAAHEGTRDYT	B	ASP247	CB	ALA9	CB	3.871	DII	H-bond/polar candidate
**PZ6**	MKSRVIRAAWSWRESAAHEGTRDYT	B	HIS249	CE1	ALA9	CB	3.674	DII	H-bond/polar candidate
**PZ6**	MKSRVIRAAWSWRESAAHEGTRDYT	B	HIS249	ND1	ALA9	C	3.851	DII	H-bond/polar candidate

**PZ6**	MKSRVIRAAWSWRESAAHEGTRDYT	B	HIS249	HD1	ALA9	C	3.614	DII	H-bond/polar candidate
**PZ6**	MKSRVIRAAWSWRESAAHEGTRDYT	B	HIS249	CE1	ALA9	C	3.677	DII	H-bond/polar candidate
**PZ6**	MKSRVIRAAWSWRESAAHEGTRDYT	B	HIS249	ND1	ALA9	O	3.75	DII	H-bond/polar candidate
**PZ6**	MKSRVIRAAWSWRESAAHEGTRDYT	B	HIS249	HD1	ALA9	O	3.744	DII	H-bond/polar candidate
**PZ6**	MKSRVIRAAWSWRESAAHEGTRDYT	B	HIS249	CE1	ALA9	O	3.564	DII	H-bond/polar candidate
**PZ6**	MKSRVIRAAWSWRESAAHEGTRDYT	B	HIS249	ND1	TRP10	N	3.874	DII	H-bond/polar candidate
**PZ6**	MKSRVIRAAWSWRESAAHEGTRDYT	B	HIS249	HD1	TRP10	N	3.31	DII	H-bond/polar candidate
**PZ6**	MKSRVIRAAWSWRESAAHEGTRDYT	B	HIS249	HD1	TRP10	H	3.73	DII	H-bond/polar candidate
**PZ6**	MKSRVIRAAWSWRESAAHEGTRDYT	B	HIS249	ND1	TRP10	CA	3.846	DII	H-bond/polar candidate
**PZ6**	MKSRVIRAAWSWRESAAHEGTRDYT	B	HIS249	HD1	TRP10	CA	3.206	DII	H-bond/polar candidate
**PZ6**	MKSRVIRAAWSWRESAAHEGTRDYT	B	ALA248	O	TRP10	CB	3.718	DII	hydrophobic/van der Waals
**PZ6**	MKSRVIRAAWSWRESAAHEGTRDYT	B	HIS249	HD1	TRP10	CB	3.736	DII	H-bond/polar candidate
**PZ6**	MKSRVIRAAWSWRESAAHEGTRDYT	B	ASP98	H	TRP10	CE2	3.899	fusion-loop region	H-bond/polar candidate
**PZ6**	MKSRVIRAAWSWRESAAHEGTRDYT	B	ASP98	CB	TRP10	CE2	3.606	fusion-loop region	H-bond/polar candidate
**PZ6**	MKSRVIRAAWSWRESAAHEGTRDYT	B	ASP98	C	TRP10	CE2	3.934	fusion-loop region	H-bond/polar candidate
**PZ6**	MKSRVIRAAWSWRESAAHEGTRDYT	B	ASP98	O	TRP10	CE2	3.203	fusion-loop region	H-bond/polar candidate
**PZ6**	MKSRVIRAAWSWRESAAHEGTRDYT	B	ASN103	HD22	TRP10	CE2	3.87	fusion-loop region	H-bond/polar candidate
**PZ6**	MKSRVIRAAWSWRESAAHEGTRDYT	B	VAL97	CG2	TRP10	CD1	3.483	DII	hydrophobic/van der Waals
**PZ6**	MKSRVIRAAWSWRESAAHEGTRDYT	B	ALA248	O	TRP10	CD1	3.871	DII	hydrophobic/van der Waals
**PZ6**	MKSRVIRAAWSWRESAAHEGTRDYT	B	HIS249	CA	TRP10	CD1	3.683	DII	H-bond/polar candidate
**PZ6**	MKSRVIRAAWSWRESAAHEGTRDYT	B	HIS249	C	TRP10	CD1	3.817	DII	H-bond/polar candidate
**PZ6**	MKSRVIRAAWSWRESAAHEGTRDYT	B	ALA250	N	TRP10	CD1	3.785	DII	hydrophobic/van der Waals
**PZ6**	MKSRVIRAAWSWRESAAHEGTRDYT	B	ALA250	H	TRP10	CD1	3.56	DII	hydrophobic/van der Waals
**PZ6**	MKSRVIRAAWSWRESAAHEGTRDYT	B	VAL97	CG2	TRP10	NE1	3.815	DII	hydrophobic/van der Waals

**PZ6**	MKSRVIRAAWSWRESAAHEGTRDYT	B	ASP98	H	TRP10	NE1	3.798	fusion-loop region	H-bond/polar candidate
**PZ6**	MKSRVIRAAWSWRESAAHEGTRDYT	B	ASP98	O	TRP10	NE1	3.026	fusion-loop region	H-bond/polar candidate
**PZ6**	MKSRVIRAAWSWRESAAHEGTRDYT	B	ASN103	HD21	TRP10	NE1	3.853	fusion-loop region	H-bond/polar candidate
**PZ6**	MKSRVIRAAWSWRESAAHEGTRDYT	B	ASN103	HD22	TRP10	NE1	3.746	fusion-loop region	H-bond/polar candidate
**PZ6**	MKSRVIRAAWSWRESAAHEGTRDYT	B	ALA250	N	TRP10	NE1	3.809	DII	hydrophobic/van der Waals
**PZ6**	MKSRVIRAAWSWRESAAHEGTRDYT	B	ALA250	H	TRP10	NE1	3.573	DII	hydrophobic/van der Waals
**PZ6**	MKSRVIRAAWSWRESAAHEGTRDYT	B	VAL97	CG2	TRP10	HE1	3.768	DII	hydrophobic/van der Waals
**PZ6**	MKSRVIRAAWSWRESAAHEGTRDYT	B	ASP98	C	TRP10	HE1	3.653	fusion-loop region	H-bond/polar candidate
**PZ6**	MKSRVIRAAWSWRESAAHEGTRDYT	B	ASP98	O	TRP10	HE1	2.518	fusion-loop region	H-bond/polar candidate
**PZ6**	MKSRVIRAAWSWRESAAHEGTRDYT	B	ASN103	ND2	TRP10	HE1	3.487	fusion-loop region	H-bond/polar candidate
**PZ6**	MKSRVIRAAWSWRESAAHEGTRDYT	B	ASN103	HD21	TRP10	HE1	3.252	fusion-loop region	H-bond/polar candidate
**PZ6**	MKSRVIRAAWSWRESAAHEGTRDYT	B	ASN103	HD22	TRP10	HE1	3.061	fusion-loop region	H-bond/polar candidate
**PZ6**	MKSRVIRAAWSWRESAAHEGTRDYT	B	HIS249	C	TRP10	HE1	3.901	DII	H-bond/polar candidate
**PZ6**	MKSRVIRAAWSWRESAAHEGTRDYT	B	ALA250	N	TRP10	HE1	3.276	DII	hydrophobic/van der Waals
**PZ6**	MKSRVIRAAWSWRESAAHEGTRDYT	B	ALA250	H	TRP10	HE1	3.146	DII	hydrophobic/van der Waals
**PZ6**	MKSRVIRAAWSWRESAAHEGTRDYT	B	ALA250	CA	TRP10	HE1	3.339	DII	hydrophobic/van der Waals
**PZ6**	MKSRVIRAAWSWRESAAHEGTRDYT	B	ALA250	CB	TRP10	HE1	3.537	DII	hydrophobic/van der Waals
**PZ6**	MKSRVIRAAWSWRESAAHEGTRDYT	B	ASP98	CA	TRP10	CZ2	3.899	fusion-loop region	H-bond/polar candidate

**PZ6**	MKSRVIRAAWSWRESAAHEGTRDYT	B	ASP98	CB	TRP10	CZ2	3.304	fusion-loop region	H-bond/polar candidate
**PZ6**	MKSRVIRAAWSWRESAAHEGTRDYT	B	ASP98	C	TRP10	CZ2	3.583	fusion-loop region	H-bond/polar candidate
**PZ6**	MKSRVIRAAWSWRESAAHEGTRDYT	B	ASP98	O	TRP10	CZ2	3.047	fusion-loop region	H-bond/polar candidate
**PZ6**	MKSRVIRAAWSWRESAAHEGTRDYT	B	ASN103	ND2	TRP10	CZ2	3.925	fusion-loop region	H-bond/polar candidate
**PZ6**	MKSRVIRAAWSWRESAAHEGTRDYT	B	ASN103	HD21	TRP10	CZ2	3.744	fusion-loop region	H-bond/polar candidate
**PZ6**	MKSRVIRAAWSWRESAAHEGTRDYT	B	ASN103	HD22	TRP10	CZ2	3.342	fusion-loop region	H-bond/polar candidate
**PZ6**	MKSRVIRAAWSWRESAAHEGTRDYT	A	GLY5	O	TRP10	CZ3	3.793	DI	hydrophobic/van der Waals
**PZ6**	MKSRVIRAAWSWRESAAHEGTRDYT	A	SER7	H	TRP10	CZ3	3.349	DI	H-bond/polar candidate
**PZ6**	MKSRVIRAAWSWRESAAHEGTRDYT	B	ASP98	CB	TRP10	CZ3	3.887	fusion-loop region	H-bond/polar candidate
**PZ6**	MKSRVIRAAWSWRESAAHEGTRDYT	A	GLY5	O	TRP10	CH2	3.262	DI	hydrophobic/van der Waals
**PZ6**	MKSRVIRAAWSWRESAAHEGTRDYT	B	ASP98	CB	TRP10	CH2	3.465	fusion-loop region	H-bond/polar candidate
**PZ6**	MKSRVIRAAWSWRESAAHEGTRDYT	A	GLY28	CA	SER11	CB	3.679	DI	H-bond/polar candidate
**PZ6**	MKSRVIRAAWSWRESAAHEGTRDYT	A	GLY28	C	SER11	CB	3.702	DI	H-bond/polar candidate
**PZ6**	MKSRVIRAAWSWRESAAHEGTRDYT	A	GLY28	O	SER11	CB	2.985	DI	H-bond/polar candidate
**PZ6**	MKSRVIRAAWSWRESAAHEGTRDYT	A	VAL6	CG1	SER11	OG	3.664	DI	H-bond/polar candidate
**PZ6**	MKSRVIRAAWSWRESAAHEGTRDYT	A	SER7	H	SER11	OG	3.476	DI	H-bond/polar candidate
**PZ6**	MKSRVIRAAWSWRESAAHEGTRDYT	A	ASN8	H	SER11	OG	3.793	DI	H-bond/polar candidate
**PZ6**	MKSRVIRAAWSWRESAAHEGTRDYT	A	GLY28	C	SER11	OG	3.773	DI	H-bond/polar candidate
**PZ6**	MKSRVIRAAWSWRESAAHEGTRDYT	A	GLY28	O	SER11	OG	2.696	DI	H-bond/polar candidate
**PZ6**	MKSRVIRAAWSWRESAAHEGTRDYT	A	VAL6	CB	SER11	HG	3.894	DI	H-bond/polar candidate

**PZ6**	MKSRVIRAAWSWRESAAHEGTRDYT	A	VAL6	CG1	SER11	HG	3.059	DI	H-bond/polar candidate
**PZ6**	MKSRVIRAAWSWRESAAHEGTRDYT	A	VAL6	CG2	SER11	HG	3.978	DI	H-bond/polar candidate
**PZ6**	MKSRVIRAAWSWRESAAHEGTRDYT	A	SER7	H	SER11	HG	3.803	DI	H-bond/polar candidate
**PZ6**	MKSRVIRAAWSWRESAAHEGTRDYT	A	ASN8	H	SER11	HG	3.747	DI	H-bond/polar candidate
**PZ6**	MKSRVIRAAWSWRESAAHEGTRDYT	A	GLY28	CA	SER11	HG	3.709	DI	H-bond/polar candidate
**PZ6**	MKSRVIRAAWSWRESAAHEGTRDYT	A	GLY28	C	SER11	HG	2.994	DI	H-bond/polar candidate
**PZ6**	MKSRVIRAAWSWRESAAHEGTRDYT	A	GLY28	O	SER11	HG	1.837	DI	H-bond/polar candidate
**PZ6**	MKSRVIRAAWSWRESAAHEGTRDYT	A	GLY28	CA	SER11	C	3.916	DI	H-bond/polar candidate
**PZ6**	MKSRVIRAAWSWRESAAHEGTRDYT	A	GLY28	CA	TRP12	N	3.628	DI	hydrophobic/van der Waals
**PZ6**	MKSRVIRAAWSWRESAAHEGTRDYT	A	GLY28	CA	TRP12	H	3.698	DI	hydrophobic/van der Waals
**PZ6**	MKSRVIRAAWSWRESAAHEGTRDYT	A	VAL46	O	TRP12	CD2	3.865	DI	hydrophobic/van der Waals
**PZ6**	MKSRVIRAAWSWRESAAHEGTRDYT	A	VAL46	O	TRP12	CE2	3.493	DI	hydrophobic/van der Waals
**PZ6**	MKSRVIRAAWSWRESAAHEGTRDYT	A	ARG2	HH21	TRP12	CE3	3.534	DI	H-bond/polar candidate
**PZ6**	MKSRVIRAAWSWRESAAHEGTRDYT	A	VAL46	O	TRP12	CD1	3.805	DI	hydrophobic/van der Waals
**PZ6**	MKSRVIRAAWSWRESAAHEGTRDYT	A	VAL46	O	TRP12	NE1	3.468	DI	hydrophobic/van der Waals
**PZ6**	MKSRVIRAAWSWRESAAHEGTRDYT	A	VAL46	O	TRP12	HE1	3.681	DI	hydrophobic/van der Waals
**PZ6**	MKSRVIRAAWSWRESAAHEGTRDYT	A	THR47	CG2	TRP12	HE1	3.538	DI	H-bond/polar candidate
**PZ6**	MKSRVIRAAWSWRESAAHEGTRDYT	A	VAL46	CB	TRP12	CZ2	3.837	DI	hydrophobic/van der Waals
**PZ6**	MKSRVIRAAWSWRESAAHEGTRDYT	A	VAL46	O	TRP12	CZ2	3.962	DI	hydrophobic/van der Waals
**PZ6**	MKSRVIRAAWSWRESAAHEGTRDYT	A	ARG2	NH2	TRP12	CZ3	3.451	DI	H-bond/polar candidate
**PZ6**	MKSRVIRAAWSWRESAAHEGTRDYT	A	ARG2	HH21	TRP12	CZ3	2.728	DI	H-bond/polar candidate
**PZ6**	MKSRVIRAAWSWRESAAHEGTRDYT	A	ARG2	HH22	TRP12	CZ3	3.709	DI	H-bond/polar candidate
**PZ6**	MKSRVIRAAWSWRESAAHEGTRDYT	A	VAL46	CG1	TRP12	CZ3	3.846	DI	hydrophobic/van der Waals
**PZ6**	MKSRVIRAAWSWRESAAHEGTRDYT	A	ARG2	HH21	TRP12	CH2	3.689	DI	H-bond/polar candidate
**PZ6**	MKSRVIRAAWSWRESAAHEGTRDYT	A	VAL46	CB	TRP12	CH2	3.647	DI	hydrophobic/van der Waals
**PZ6**	MKSRVIRAAWSWRESAAHEGTRDYT	A	VAL46	CG1	TRP12	CH2	3.683	DI	hydrophobic/van der Waals
**PZ6**	MKSRVIRAAWSWRESAAHEGTRDYT	B	HIS249	CD2	ARG13	CG	3.588	DII	H-bond/polar candidate
**PZ6**	MKSRVIRAAWSWRESAAHEGTRDYT	B	HIS249	NE2	ARG13	CG	3.87	DII	H-bond/polar candidate
**PZ6**	MKSRVIRAAWSWRESAAHEGTRDYT	B	HIS249	CD2	ARG13	CD	3.912	DII	H-bond/polar candidate
**PZ6**	MKSRVIRAAWSWRESAAHEGTRDYT	B	ALA250	H	ARG13	HH12	3.923	DII	H-bond/polar candidate

**PZ6**	MKSRVIRAAWSWRESAAHEGTRDYT	B	ASN103	HD21	GLU14	CB	3.367	fusion-loop region	H-bond/polar candidate
**PZ6**	MKSRVIRAAWSWRESAAHEGTRDYT	B	ASN103	ND2	GLU14	CG	3.85	fusion-loop region	H-bond/polar candidate
**PZ6**	MKSRVIRAAWSWRESAAHEGTRDYT	B	ASN103	HD21	GLU14	CG	2.902	fusion-loop region	H-bond/polar candidate
**PZ6**	MKSRVIRAAWSWRESAAHEGTRDYT	B	ALA250	H	GLU14	CG	3.661	DII	H-bond/polar candidate
**PZ6**	MKSRVIRAAWSWRESAAHEGTRDYT	B	ASN103	ND2	GLU14	CD	3.807	fusion-loop region	H-bond/polar candidate
**PZ6**	MKSRVIRAAWSWRESAAHEGTRDYT	B	ASN103	HD21	GLU14	CD	2.862	fusion-loop region	H-bond/polar candidate
**PZ6**	MKSRVIRAAWSWRESAAHEGTRDYT	B	ALA250	N	GLU14	CD	3.902	DII	H-bond/polar candidate
**PZ6**	MKSRVIRAAWSWRESAAHEGTRDYT	B	ALA250	H	GLU14	CD	3.004	DII	H-bond/polar candidate
**PZ6**	MKSRVIRAAWSWRESAAHEGTRDYT	B	ALA250	CB	GLU14	CD	3.474	DII	H-bond/polar candidate
**PZ6**	MKSRVIRAAWSWRESAAHEGTRDYT	B	ASN103	HD21	GLU14	OE1	3.828	fusion-loop region	H-bond/polar candidate
**PZ6**	MKSRVIRAAWSWRESAAHEGTRDYT	B	HIS249	CB	GLU14	OE1	3.541	DII	salt bridge candidate; H-bond/polar candidate
**PZ6**	MKSRVIRAAWSWRESAAHEGTRDYT	B	ALA250	N	GLU14	OE1	3.189	DII	H-bond/polar candidate
**PZ6**	MKSRVIRAAWSWRESAAHEGTRDYT	B	ALA250	H	GLU14	OE1	2.219	DII	H-bond/polar candidate
**PZ6**	MKSRVIRAAWSWRESAAHEGTRDYT	B	ALA250	CA	GLU14	OE1	3.873	DII	H-bond/polar candidate
**PZ6**	MKSRVIRAAWSWRESAAHEGTRDYT	B	ALA250	CB	GLU14	OE1	3.366	DII	H-bond/polar candidate
**PZ6**	MKSRVIRAAWSWRESAAHEGTRDYT	B	ASN103	CB	GLU14	OE2	3.152	fusion-loop region	H-bond/polar candidate
**PZ6**	MKSRVIRAAWSWRESAAHEGTRDYT	B	ASN103	CG	GLU14	OE2	3.877	fusion-loop region	H-bond/polar candidate
**PZ6**	MKSRVIRAAWSWRESAAHEGTRDYT	B	ASN103	ND2	GLU14	OE2	3.461	fusion-loop region	H-bond/polar candidate

**PZ6**	MKSRVIRAAWSWRESAAHEGTRDYT	B	ASN103	HD21	GLU14	OE2	2.571	fusion-loop region	H-bond/polar candidate
**PZ6**	MKSRVIRAAWSWRESAAHEGTRDYT	B	ALA250	H	GLU14	OE2	3.82	DII	H-bond/polar candidate
**PZ6**	MKSRVIRAAWSWRESAAHEGTRDYT	B	ALA250	CB	GLU14	OE2	3.495	DII	H-bond/polar candidate
**PZ6**	MKSRVIRAAWSWRESAAHEGTRDYT	A	ARG2	NH2	SER15	CA	3.95	DI	H-bond/polar candidate
**PZ6**	MKSRVIRAAWSWRESAAHEGTRDYT	A	ARG2	HH21	SER15	CA	3.299	DI	H-bond/polar candidate
**PZ6**	MKSRVIRAAWSWRESAAHEGTRDYT	A	ARG2	NH2	SER15	CB	3.399	DI	H-bond/polar candidate
**PZ6**	MKSRVIRAAWSWRESAAHEGTRDYT	A	ARG2	HH21	SER15	CB	2.933	DI	H-bond/polar candidate
**PZ6**	MKSRVIRAAWSWRESAAHEGTRDYT	A	ARG2	HH22	SER15	CB	3.487	DI	H-bond/polar candidate
**PZ6**	MKSRVIRAAWSWRESAAHEGTRDYT	A	GLU44	OE1	SER15	CB	3.953	DI	H-bond/polar candidate
**PZ6**	MKSRVIRAAWSWRESAAHEGTRDYT	A	ARG2	HE	SER15	OG	3.925	DI	H-bond/polar candidate
**PZ6**	MKSRVIRAAWSWRESAAHEGTRDYT	A	ARG2	CZ	SER15	OG	3.54	DI	H-bond/polar candidate
**PZ6**	MKSRVIRAAWSWRESAAHEGTRDYT	A	ARG2	NH2	SER15	OG	2.783	DI	H-bond/polar candidate
**PZ6**	MKSRVIRAAWSWRESAAHEGTRDYT	A	ARG2	HH21	SER15	OG	2.746	DI	H-bond/polar candidate
**PZ6**	MKSRVIRAAWSWRESAAHEGTRDYT	A	ARG2	HH22	SER15	OG	2.753	DI	H-bond/polar candidate
**PZ6**	MKSRVIRAAWSWRESAAHEGTRDYT	A	CYS3	H	SER15	OG	3.874	DI	H-bond/polar candidate
**PZ6**	MKSRVIRAAWSWRESAAHEGTRDYT	A	VAL6	CG2	SER15	OG	3.94	DI	H-bond/polar candidate
**PZ6**	MKSRVIRAAWSWRESAAHEGTRDYT	A	GLU44	OE1	SER15	OG	2.928	DI	H-bond/polar candidate
**PZ6**	MKSRVIRAAWSWRESAAHEGTRDYT	A	ARG2	NE	SER15	HG	3.946	DI	H-bond/polar candidate
**PZ6**	MKSRVIRAAWSWRESAAHEGTRDYT	A	ARG2	HE	SER15	HG	3.583	DI	H-bond/polar candidate
**PZ6**	MKSRVIRAAWSWRESAAHEGTRDYT	A	ARG2	CZ	SER15	HG	3.606	DI	H-bond/polar candidate
**PZ6**	MKSRVIRAAWSWRESAAHEGTRDYT	A	ARG2	NH2	SER15	HG	2.732	DI	H-bond/polar candidate
**PZ6**	MKSRVIRAAWSWRESAAHEGTRDYT	A	ARG2	HH21	SER15	HG	2.888	DI	H-bond/polar candidate
**PZ6**	MKSRVIRAAWSWRESAAHEGTRDYT	A	ARG2	HH22	SER15	HG	2.352	DI	H-bond/polar candidate
**PZ6**	MKSRVIRAAWSWRESAAHEGTRDYT	A	CYS3	H	SER15	HG	3.881	DI	H-bond/polar candidate
**PZ6**	MKSRVIRAAWSWRESAAHEGTRDYT	A	VAL6	CG2	SER15	HG	3.712	DI	H-bond/polar candidate
**PZ6**	MKSRVIRAAWSWRESAAHEGTRDYT	A	GLU44	CD	SER15	HG	3.247	DI	H-bond/polar candidate
**PZ6**	MKSRVIRAAWSWRESAAHEGTRDYT	A	GLU44	OE1	SER15	HG	2.105	DI	H-bond/polar candidate
**PZ6**	MKSRVIRAAWSWRESAAHEGTRDYT	A	GLU44	OE2	SER15	HG	3.85	DI	H-bond/polar candidate
**PZ6**	MKSRVIRAAWSWRESAAHEGTRDYT	A	ARG2	NH1	SER15	C	3.934	DI	H-bond/polar candidate

**PZ6**	MKSRVIRAAWSWRESAAHEGTRDYT	A	ARG2	HH11	SER15	C	3.164	DI	H-bond/polar candidate
**PZ6**	MKSRVIRAAWSWRESAAHEGTRDYT	A	ARG2	NH2	SER15	C	3.521	DI	H-bond/polar candidate
**PZ6**	MKSRVIRAAWSWRESAAHEGTRDYT	A	ARG2	HH21	SER15	C	2.688	DI	H-bond/polar candidate
**PZ6**	MKSRVIRAAWSWRESAAHEGTRDYT	A	ARG2	CZ	SER15	O	3.037	DI	H-bond/polar candidate
**PZ6**	MKSRVIRAAWSWRESAAHEGTRDYT	A	ARG2	NH1	SER15	O	2.717	DI	H-bond/polar candidate
**PZ6**	MKSRVIRAAWSWRESAAHEGTRDYT	A	ARG2	HH11	SER15	O	1.941	DI	H-bond/polar candidate
**PZ6**	MKSRVIRAAWSWRESAAHEGTRDYT	A	ARG2	HH12	SER15	O	3.585	DI	H-bond/polar candidate
**PZ6**	MKSRVIRAAWSWRESAAHEGTRDYT	A	ARG2	NH2	SER15	O	2.706	DI	H-bond/polar candidate
**PZ6**	MKSRVIRAAWSWRESAAHEGTRDYT	A	ARG2	HH21	SER15	O	1.995	DI	H-bond/polar candidate
**PZ6**	MKSRVIRAAWSWRESAAHEGTRDYT	A	ARG2	HH22	SER15	O	3.589	DI	H-bond/polar candidate
**PZ6**	MKSRVIRAAWSWRESAAHEGTRDYT	A	ARG2	HH11	ALA16	N	3.964	DI	H-bond/polar candidate
**PZ6**	MKSRVIRAAWSWRESAAHEGTRDYT	A	ARG2	HH21	ALA16	N	3.701	DI	H-bond/polar candidate
**PZ6**	MKSRVIRAAWSWRESAAHEGTRDYT	A	ARG2	HH11	ALA16	CA	3.787	DI	H-bond/polar candidate
**PZ6**	MKSRVIRAAWSWRESAAHEGTRDYT	A	ARG2	HH11	ALA16	C	3.883	DI	H-bond/polar candidate
**PZ6**	MKSRVIRAAWSWRESAAHEGTRDYT	A	ARG2	HH11	ALA16	O	3.609	DI	H-bond/polar candidate
**PZ6**	MKSRVIRAAWSWRESAAHEGTRDYT	B	GLY102	O	ALA17	CB	3.624	fusion-loop region	hydrophobic/van der Waals
**PZ6**	MKSRVIRAAWSWRESAAHEGTRDYT	A	ARG2	NH1	HIS18	CA	3.993	DI	H-bond/polar candidate
**PZ6**	MKSRVIRAAWSWRESAAHEGTRDYT	A	ARG2	HH11	HIS18	CA	3.786	DI	H-bond/polar candidate
**PZ6**	MKSRVIRAAWSWRESAAHEGTRDYT	A	ARG2	CG	HIS18	CB	3.894	DI	H-bond/polar candidate
**PZ6**	MKSRVIRAAWSWRESAAHEGTRDYT	A	ARG2	CZ	HIS18	CB	3.843	DI	H-bond/polar candidate
**PZ6**	MKSRVIRAAWSWRESAAHEGTRDYT	A	ARG2	NH1	HIS18	CB	3.714	DI	H-bond/polar candidate
**PZ6**	MKSRVIRAAWSWRESAAHEGTRDYT	A	ARG2	HH11	HIS18	CB	3.682	DI	H-bond/polar candidate
**PZ6**	MKSRVIRAAWSWRESAAHEGTRDYT	A	CYS3	H	HIS18	CB	3.893	DI	H-bond/polar candidate
**PZ6**	MKSRVIRAAWSWRESAAHEGTRDYT	A	ILE1	O	HIS18	CG	3.981	DI	H-bond/polar candidate
**PZ6**	MKSRVIRAAWSWRESAAHEGTRDYT	A	CYS3	H	HIS18	CG	3.451	DI	H-bond/polar candidate
**PZ6**	MKSRVIRAAWSWRESAAHEGTRDYT	A	ILE1	O	HIS18	ND1	3.649	DI	H-bond/polar candidate
**PZ6**	MKSRVIRAAWSWRESAAHEGTRDYT	A	ILE1	O	HIS18	HD1	3.81	DI	H-bond/polar candidate

**PZ6**	MKSRVIRAAWSWRESAAHEGTRDYT	B	GLY102	CA	HIS18	HD1	3.777	fusion-loop region	H-bond/polar candidate
**PZ6**	MKSRVIRAAWSWRESAAHEGTRDYT	A	CYS3	N	HIS18	CD2	3.319	DI	H-bond/polar candidate
**PZ6**	MKSRVIRAAWSWRESAAHEGTRDYT	A	CYS3	H	HIS18	CD2	2.701	DI	H-bond/polar candidate
**PZ6**	MKSRVIRAAWSWRESAAHEGTRDYT	A	GLY5	H	HIS18	CD2	3.291	DI	H-bond/polar candidate
**PZ6**	MKSRVIRAAWSWRESAAHEGTRDYT	A	CYS3	N	HIS18	NE2	3.688	DI	H-bond/polar candidate
**PZ6**	MKSRVIRAAWSWRESAAHEGTRDYT	A	CYS3	H	HIS18	NE2	3.409	DI	H-bond/polar candidate
**PZ6**	MKSRVIRAAWSWRESAAHEGTRDYT	A	ILE4	N	HIS18	NE2	3.811	DI	H-bond/polar candidate
**PZ6**	MKSRVIRAAWSWRESAAHEGTRDYT	A	ILE4	H	HIS18	NE2	3.777	DI	H-bond/polar candidate
**PZ6**	MKSRVIRAAWSWRESAAHEGTRDYT	A	GLY5	N	HIS18	NE2	3.144	DI	H-bond/polar candidate
**PZ6**	MKSRVIRAAWSWRESAAHEGTRDYT	A	GLY5	H	HIS18	NE2	2.312	DI	H-bond/polar candidate
**PZ6**	MKSRVIRAAWSWRESAAHEGTRDYT	A	GLY5	CA	HIS18	NE2	3.601	DI	H-bond/polar candidate
**PZ6**	MKSRVIRAAWSWRESAAHEGTRDYT	A	ILE1	O	HIS18	CE1	3.753	DI	H-bond/polar candidate
**PZ6**	MKSRVIRAAWSWRESAAHEGTRDYT	A	ILE4	CG1	HIS18	CE1	3.969	DI	H-bond/polar candidate
**PZ6**	MKSRVIRAAWSWRESAAHEGTRDYT	A	GLY5	N	HIS18	CE1	3.976	DI	H-bond/polar candidate
**PZ6**	MKSRVIRAAWSWRESAAHEGTRDYT	A	GLY5	H	HIS18	CE1	3.295	DI	H-bond/polar candidate
**PZ6**	MKSRVIRAAWSWRESAAHEGTRDYT	A	ARG2	CG	HIS18	C	3.98	DI	H-bond/polar candidate
**PZ6**	MKSRVIRAAWSWRESAAHEGTRDYT	A	ARG2	NH1	HIS18	C	3.399	DI	H-bond/polar candidate
**PZ6**	MKSRVIRAAWSWRESAAHEGTRDYT	A	ARG2	HH11	HIS18	C	3.375	DI	H-bond/polar candidate
**PZ6**	MKSRVIRAAWSWRESAAHEGTRDYT	A	ARG2	HH12	HIS18	C	3.196	DI	H-bond/polar candidate
**PZ6**	MKSRVIRAAWSWRESAAHEGTRDYT	A	ARG2	CG	HIS18	O	3.429	DI	H-bond/polar candidate
**PZ6**	MKSRVIRAAWSWRESAAHEGTRDYT	A	ARG2	NH1	HIS18	O	3.917	DI	H-bond/polar candidate
**PZ6**	MKSRVIRAAWSWRESAAHEGTRDYT	A	ARG2	HH12	HIS18	O	3.487	DI	H-bond/polar candidate
**PZ6**	MKSRVIRAAWSWRESAAHEGTRDYT	A	ARG2	NH1	GLU19	N	3.038	DI	salt bridge candidate; H-bond/polar candidate
**PZ6**	MKSRVIRAAWSWRESAAHEGTRDYT	A	ARG2	HH11	GLU19	N	2.79	DI	salt bridge candidate; H-bond/polar candidate
**PZ6**	MKSRVIRAAWSWRESAAHEGTRDYT	A	ARG2	HH12	GLU19	N	2.786	DI	salt bridge candidate; H-bond/polar candidate
**PZ6**	MKSRVIRAAWSWRESAAHEGTRDYT	A	ARG2	NH1	GLU19	H	3.186	DI	salt bridge candidate; H-bond/polar candidate
**PZ6**	MKSRVIRAAWSWRESAAHEGTRDYT	A	ARG2	HH11	GLU19	H	2.623	DI	salt bridge candidate; H-bond/polar candidate
**PZ6**	MKSRVIRAAWSWRESAAHEGTRDYT	A	ARG2	HH12	GLU19	H	3.191	DI	salt bridge candidate; H-bond/polar candidate
**PZ6**	MKSRVIRAAWSWRESAAHEGTRDYT	A	ARG2	NH1	GLU19	CA	3.563	DI	salt bridge candidate; H-bond/polar candidate

**PZ6**	MKSRVIRAAWSWRESAAHEGTRDYT	A	ARG2	HH11	GLU19	CA	3.523	DI	salt bridge candidate; H-bond/polar candidate
**PZ6**	MKSRVIRAAWSWRESAAHEGTRDYT	A	ARG2	HH12	GLU19	CA	2.928	DI	salt bridge candidate; H-bond/polar candidate
**PZ6**	MKSRVIRAAWSWRESAAHEGTRDYT	A	ARG164	NH2	GLU19	CA	3.625	DI	salt bridge candidate; H-bond/polar candidate
**PZ6**	MKSRVIRAAWSWRESAAHEGTRDYT	A	ARG164	HH21	GLU19	CA	3.385	DI	salt bridge candidate; H-bond/polar candidate
**PZ6**	MKSRVIRAAWSWRESAAHEGTRDYT	A	ARG164	HH22	GLU19	CA	3.14	DI	salt bridge candidate; H-bond/polar candidate
**PZ6**	MKSRVIRAAWSWRESAAHEGTRDYT	A	ARG2	NH1	GLU19	CB	3.865	DI	salt bridge candidate; H-bond/polar candidate
**PZ6**	MKSRVIRAAWSWRESAAHEGTRDYT	A	ARG2	HH11	GLU19	CB	3.654	DI	salt bridge candidate; H-bond/polar candidate
**PZ6**	MKSRVIRAAWSWRESAAHEGTRDYT	A	ARG2	HH12	GLU19	CB	3.303	DI	salt bridge candidate; H-bond/polar candidate
**PZ6**	MKSRVIRAAWSWRESAAHEGTRDYT	A	ARG164	NH2	GLU19	CB	3.576	DI	salt bridge candidate; H-bond/polar candidate
**PZ6**	MKSRVIRAAWSWRESAAHEGTRDYT	A	ARG164	HH21	GLU19	CB	3.406	DI	salt bridge candidate; H-bond/polar candidate
**PZ6**	MKSRVIRAAWSWRESAAHEGTRDYT	A	ARG164	HH22	GLU19	CB	2.996	DI	salt bridge candidate; H-bond/polar candidate
**PZ6**	MKSRVIRAAWSWRESAAHEGTRDYT	A	ARG2	NH1	GLU19	CG	3.197	DI	salt bridge candidate; H-bond/polar candidate
**PZ6**	MKSRVIRAAWSWRESAAHEGTRDYT	A	ARG2	HH11	GLU19	CG	3.052	DI	salt bridge candidate; H-bond/polar candidate
**PZ6**	MKSRVIRAAWSWRESAAHEGTRDYT	A	ARG2	HH12	GLU19	CG	2.73	DI	salt bridge candidate; H-bond/polar candidate
**PZ6**	MKSRVIRAAWSWRESAAHEGTRDYT	A	ARG164	NH2	GLU19	CG	3.893	DI	salt bridge candidate; H-bond/polar candidate
**PZ6**	MKSRVIRAAWSWRESAAHEGTRDYT	A	ARG164	HH22	GLU19	CG	3.04	DI	salt bridge candidate; H-bond/polar candidate
**PZ6**	MKSRVIRAAWSWRESAAHEGTRDYT	A	LYS166	HZ1	GLU19	CG	3.764	DI	salt bridge candidate; H-bond/polar candidate
**PZ6**	MKSRVIRAAWSWRESAAHEGTRDYT	A	ARG2	NH1	GLU19	CD	3.381	DI	salt bridge candidate; H-bond/polar candidate
**PZ6**	MKSRVIRAAWSWRESAAHEGTRDYT	A	ARG2	HH11	GLU19	CD	3.668	DI	salt bridge candidate; H-bond/polar candidate
**PZ6**	MKSRVIRAAWSWRESAAHEGTRDYT	A	ARG2	HH12	GLU19	CD	2.65	DI	salt bridge candidate; H-bond/polar candidate
**PZ6**	MKSRVIRAAWSWRESAAHEGTRDYT	A	MET140	SD	GLU19	CD	3.811	DI	H-bond/polar candidate
**PZ6**	MKSRVIRAAWSWRESAAHEGTRDYT	A	MET140	CE	GLU19	CD	3.858	DI	H-bond/polar candidate
**PZ6**	MKSRVIRAAWSWRESAAHEGTRDYT	A	ARG164	HE	GLU19	CD	3.307	DI	salt bridge candidate; H-bond/polar candidate
**PZ6**	MKSRVIRAAWSWRESAAHEGTRDYT	A	ARG164	CZ	GLU19	CD	3.962	DI	salt bridge candidate; H-bond/polar candidate
**PZ6**	MKSRVIRAAWSWRESAAHEGTRDYT	A	ARG164	NH2	GLU19	CD	3.072	DI	salt bridge candidate; H-bond/polar candidate
**PZ6**	MKSRVIRAAWSWRESAAHEGTRDYT	A	ARG164	HH21	GLU19	CD	3.634	DI	salt bridge candidate; H-bond/polar candidate
**PZ6**	MKSRVIRAAWSWRESAAHEGTRDYT	A	ARG164	HH22	GLU19	CD	2.099	DI	salt bridge candidate; H-bond/polar candidate
**PZ6**	MKSRVIRAAWSWRESAAHEGTRDYT	A	LYS166	NZ	GLU19	CD	3.648	DI	salt bridge candidate; H-bond/polar candidate
**PZ6**	MKSRVIRAAWSWRESAAHEGTRDYT	A	LYS166	HZ1	GLU19	CD	2.902	DI	salt bridge candidate; H-bond/polar candidate
**PZ6**	MKSRVIRAAWSWRESAAHEGTRDYT	A	LYS166	HZ3	GLU19	CD	3.409	DI	salt bridge candidate; H-bond/polar candidate

**PZ6**	MKSRVIRAAWSWRESAAHEGTRDYT	A	ARG2	HH12	GLU19	OE1	3.766	DI	salt bridge candidate; H-bond/polar candidate
**PZ6**	MKSRVIRAAWSWRESAAHEGTRDYT	A	MET140	SD	GLU19	OE1	3.406	DI	H-bond/polar candidate
**PZ6**	MKSRVIRAAWSWRESAAHEGTRDYT	A	MET140	CE	GLU19	OE1	3.885	DI	H-bond/polar candidate
**PZ6**	MKSRVIRAAWSWRESAAHEGTRDYT	A	ARG164	NE	GLU19	OE1	3.739	DI	salt bridge candidate; H-bond/polar candidate
**PZ6**	MKSRVIRAAWSWRESAAHEGTRDYT	A	ARG164	HE	GLU19	OE1	3.018	DI	salt bridge candidate; H-bond/polar candidate
**PZ6**	MKSRVIRAAWSWRESAAHEGTRDYT	A	ARG164	CZ	GLU19	OE1	3.898	DI	salt bridge candidate; H-bond/polar candidate
**PZ6**	MKSRVIRAAWSWRESAAHEGTRDYT	A	ARG164	NH2	GLU19	OE1	3.303	DI	salt bridge candidate; H-bond/polar candidate
**PZ6**	MKSRVIRAAWSWRESAAHEGTRDYT	A	ARG164	HH21	GLU19	OE1	3.986	DI	salt bridge candidate; H-bond/polar candidate
**PZ6**	MKSRVIRAAWSWRESAAHEGTRDYT	A	ARG164	HH22	GLU19	OE1	2.493	DI	salt bridge candidate; H-bond/polar candidate
**PZ6**	MKSRVIRAAWSWRESAAHEGTRDYT	A	LYS166	CE	GLU19	OE1	3.704	DI	salt bridge candidate; H-bond/polar candidate
**PZ6**	MKSRVIRAAWSWRESAAHEGTRDYT	A	LYS166	NZ	GLU19	OE1	2.616	DI	salt bridge candidate; H-bond/polar candidate
**PZ6**	MKSRVIRAAWSWRESAAHEGTRDYT	A	LYS166	HZ1	GLU19	OE1	1.742	DI	salt bridge candidate; H-bond/polar candidate
**PZ6**	MKSRVIRAAWSWRESAAHEGTRDYT	A	LYS166	HZ2	GLU19	OE1	3.141	DI	salt bridge candidate; H-bond/polar candidate
**PZ6**	MKSRVIRAAWSWRESAAHEGTRDYT	A	LYS166	HZ3	GLU19	OE1	2.622	DI	salt bridge candidate; H-bond/polar candidate
**PZ6**	MKSRVIRAAWSWRESAAHEGTRDYT	A	ARG2	CD	GLU19	OE2	3.3	DI	salt bridge candidate; H-bond/polar candidate
**PZ6**	MKSRVIRAAWSWRESAAHEGTRDYT	A	ARG2	NE	GLU19	OE2	3.97	DI	salt bridge candidate; H-bond/polar candidate
**PZ6**	MKSRVIRAAWSWRESAAHEGTRDYT	A	ARG2	CZ	GLU19	OE2	3.856	DI	salt bridge candidate; H-bond/polar candidate
**PZ6**	MKSRVIRAAWSWRESAAHEGTRDYT	A	ARG2	NH1	GLU19	OE2	3.022	DI	salt bridge candidate; H-bond/polar candidate
**PZ6**	MKSRVIRAAWSWRESAAHEGTRDYT	A	ARG2	HH11	GLU19	OE2	3.594	DI	salt bridge candidate; H-bond/polar candidate
**PZ6**	MKSRVIRAAWSWRESAAHEGTRDYT	A	ARG2	HH12	GLU19	OE2	2.1	DI	salt bridge candidate; H-bond/polar candidate
**PZ6**	MKSRVIRAAWSWRESAAHEGTRDYT	A	MET140	SD	GLU19	OE2	3.712	DI	H-bond/polar candidate
**PZ6**	MKSRVIRAAWSWRESAAHEGTRDYT	A	MET140	CE	GLU19	OE2	3.763	DI	H-bond/polar candidate
**PZ6**	MKSRVIRAAWSWRESAAHEGTRDYT	A	ARG164	NE	GLU19	OE2	3.613	DI	salt bridge candidate; H-bond/polar candidate
**PZ6**	MKSRVIRAAWSWRESAAHEGTRDYT	A	ARG164	HE	GLU19	OE2	2.897	DI	salt bridge candidate; H-bond/polar candidate
**PZ6**	MKSRVIRAAWSWRESAAHEGTRDYT	A	ARG164	CZ	GLU19	OE2	3.668	DI	salt bridge candidate; H-bond/polar candidate
**PZ6**	MKSRVIRAAWSWRESAAHEGTRDYT	A	ARG164	NH2	GLU19	OE2	2.872	DI	salt bridge candidate; H-bond/polar candidate
**PZ6**	MKSRVIRAAWSWRESAAHEGTRDYT	A	ARG164	HH21	GLU19	OE2	3.527	DI	salt bridge candidate; H-bond/polar candidate
**PZ6**	MKSRVIRAAWSWRESAAHEGTRDYT	A	ARG164	HH22	GLU19	OE2	1.896	DI	salt bridge candidate; H-bond/polar candidate
**PZ6**	MKSRVIRAAWSWRESAAHEGTRDYT	A	LYS166	HZ1	GLU19	OE2	3.759	DI	salt bridge candidate; H-bond/polar candidate
**PZ6**	MKSRVIRAAWSWRESAAHEGTRDYT	A	ARG164	NH2	GLU19	C	3.746	DI	salt bridge candidate; H-bond/polar candidate

**PZ6**	MKSRVIRAAWSWRESAAHEGTRDYT	A	ARG164	HH21	GLU19	C	3.134	DI	salt bridge candidate; H-bond/polar candidate
**PZ6**	MKSRVIRAAWSWRESAAHEGTRDYT	A	ARG164	HH22	GLU19	C	3.665	DI	salt bridge candidate; H-bond/polar candidate
**PZ6**	MKSRVIRAAWSWRESAAHEGTRDYT	A	ARG164	NH2	GLU19	O	3.387	DI	salt bridge candidate; H-bond/polar candidate
**PZ6**	MKSRVIRAAWSWRESAAHEGTRDYT	A	ARG164	HH21	GLU19	O	2.708	DI	salt bridge candidate; H-bond/polar candidate
**PZ6**	MKSRVIRAAWSWRESAAHEGTRDYT	A	ARG164	HH22	GLU19	O	3.571	DI	salt bridge candidate; H-bond/polar candidate
**PZ6**	MKSRVIRAAWSWRESAAHEGTRDYT	A	ILE1	H	THR21	CA	3.755	DI	H-bond/polar candidate
**PZ6**	MKSRVIRAAWSWRESAAHEGTRDYT	A	ILE1	N	THR21	CB	3.802	DI	H-bond/polar candidate
**PZ6**	MKSRVIRAAWSWRESAAHEGTRDYT	A	ILE1	H	THR21	CB	3.254	DI	H-bond/polar candidate
**PZ6**	MKSRVIRAAWSWRESAAHEGTRDYT	B	GLY102	CA	THR21	OG1	3.795	fusion-loop region	H-bond/polar candidate
**PZ6**	MKSRVIRAAWSWRESAAHEGTRDYT	A	ILE1	N	THR21	CG2	3.517	DI	H-bond/polar candidate
**PZ6**	MKSRVIRAAWSWRESAAHEGTRDYT	A	ILE1	H	THR21	CG2	2.661	DI	H-bond/polar candidate
**PZ6**	MKSRVIRAAWSWRESAAHEGTRDYT	A	ILE1	O	THR21	CG2	3.745	DI	H-bond/polar candidate
**PZ6**	MKSRVIRAAWSWRESAAHEGTRDYT	A	ILE1	N	THR21	C	3.144	DI	H-bond/polar candidate
**PZ6**	MKSRVIRAAWSWRESAAHEGTRDYT	A	ILE1	H	THR21	C	3.075	DI	H-bond/polar candidate
**PZ6**	MKSRVIRAAWSWRESAAHEGTRDYT	A	ILE1	N	THR21	O	3.085	DI	H-bond/polar candidate
**PZ6**	MKSRVIRAAWSWRESAAHEGTRDYT	A	ILE1	H	THR21	O	2.936	DI	H-bond/polar candidate
**PZ6**	MKSRVIRAAWSWRESAAHEGTRDYT	A	MET375	CE	THR21	O	3.735	DIII	H-bond/polar candidate
**PZ6**	MKSRVIRAAWSWRESAAHEGTRDYT	A	ILE1	N	ARG22	N	3.191	DI	H-bond/polar candidate
**PZ6**	MKSRVIRAAWSWRESAAHEGTRDYT	A	ILE1	H	ARG22	N	3.493	DI	H-bond/polar candidate
**PZ6**	MKSRVIRAAWSWRESAAHEGTRDYT	A	ILE1	N	ARG22	H	3.816	DI	H-bond/polar candidate
**PZ6**	MKSRVIRAAWSWRESAAHEGTRDYT	A	ILE1	N	ARG22	CA	3.284	DI	H-bond/polar candidate
**PZ6**	MKSRVIRAAWSWRESAAHEGTRDYT	A	ILE1	H	ARG22	CA	3.883	DI	H-bond/polar candidate
**PZ6**	MKSRVIRAAWSWRESAAHEGTRDYT	A	ILE1	N	ARG22	CB	3.931	DI	H-bond/polar candidate
**PZ6**	MKSRVIRAAWSWRESAAHEGTRDYT	A	SER146	O	ARG22	CB	3.258	DI	H-bond/polar candidate
**PZ6**	MKSRVIRAAWSWRESAAHEGTRDYT	A	ILE1	N	ARG22	CG	3.669	DI	H-bond/polar candidate
**PZ6**	MKSRVIRAAWSWRESAAHEGTRDYT	A	ILE1	CA	ARG22	CG	3.778	DI	H-bond/polar candidate
**PZ6**	MKSRVIRAAWSWRESAAHEGTRDYT	A	SER146	O	ARG22	CG	3.861	DI	H-bond/polar candidate
**PZ6**	MKSRVIRAAWSWRESAAHEGTRDYT	A	ARG2	CG	ARG22	CD	3.861	DI	H-bond/polar candidate
**PZ6**	MKSRVIRAAWSWRESAAHEGTRDYT	A	ARG164	NE	ARG22	CD	3.724	DI	H-bond/polar candidate

**PZ6**	MKSRVIRAAWSWRESAAHEGTRDYT	A	ARG164	HE	ARG22	CD	3.818	DI	H-bond/polar candidate
**PZ6**	MKSRVIRAAWSWRESAAHEGTRDYT	A	ARG164	CZ	ARG22	CD	3.575	DI	H-bond/polar candidate
**PZ6**	MKSRVIRAAWSWRESAAHEGTRDYT	A	ARG164	NH2	ARG22	CD	3.534	DI	H-bond/polar candidate
**PZ6**	MKSRVIRAAWSWRESAAHEGTRDYT	A	ARG164	HH21	ARG22	CD	3.887	DI	H-bond/polar candidate
**PZ6**	MKSRVIRAAWSWRESAAHEGTRDYT	A	ARG164	HH22	ARG22	CD	3.611	DI	H-bond/polar candidate
**PZ6**	MKSRVIRAAWSWRESAAHEGTRDYT	A	SER146	OG	ARG22	NE	3.92	DI	H-bond/polar candidate
**PZ6**	MKSRVIRAAWSWRESAAHEGTRDYT	A	SER146	C	ARG22	NE	3.197	DI	H-bond/polar candidate
**PZ6**	MKSRVIRAAWSWRESAAHEGTRDYT	A	SER146	O	ARG22	NE	3.285	DI	H-bond/polar candidate
**PZ6**	MKSRVIRAAWSWRESAAHEGTRDYT	A	ARG164	NE	ARG22	NE	3.989	DI	H-bond/polar candidate
**PZ6**	MKSRVIRAAWSWRESAAHEGTRDYT	A	SER146	CA	ARG22	HE	3.527	DI	H-bond/polar candidate
**PZ6**	MKSRVIRAAWSWRESAAHEGTRDYT	A	SER146	CB	ARG22	HE	3.626	DI	H-bond/polar candidate
**PZ6**	MKSRVIRAAWSWRESAAHEGTRDYT	A	SER146	OG	ARG22	HE	3.488	DI	H-bond/polar candidate
**PZ6**	MKSRVIRAAWSWRESAAHEGTRDYT	A	SER146	C	ARG22	HE	2.445	DI	H-bond/polar candidate
**PZ6**	MKSRVIRAAWSWRESAAHEGTRDYT	A	SER146	O	ARG22	HE	2.378	DI	H-bond/polar candidate
**PZ6**	MKSRVIRAAWSWRESAAHEGTRDYT	A	SER142	CB	ARG22	CZ	3.67	DI	H-bond/polar candidate
**PZ6**	MKSRVIRAAWSWRESAAHEGTRDYT	A	SER142	OG	ARG22	CZ	3.211	DI	H-bond/polar candidate
**PZ6**	MKSRVIRAAWSWRESAAHEGTRDYT	A	SER146	OG	ARG22	CZ	3.637	DI	H-bond/polar candidate
**PZ6**	MKSRVIRAAWSWRESAAHEGTRDYT	A	SER146	C	ARG22	CZ	3.755	DI	H-bond/polar candidate
**PZ6**	MKSRVIRAAWSWRESAAHEGTRDYT	A	ARG2	H	ARG22	NH1	3.996	DI	H-bond/polar candidate
**PZ6**	MKSRVIRAAWSWRESAAHEGTRDYT	A	ARG2	CB	ARG22	NH1	3.497	DI	H-bond/polar candidate
**PZ6**	MKSRVIRAAWSWRESAAHEGTRDYT	A	ARG2	CG	ARG22	NH1	3.251	DI	H-bond/polar candidate
**PZ6**	MKSRVIRAAWSWRESAAHEGTRDYT	A	ARG2	CD	ARG22	NH1	3.154	DI	H-bond/polar candidate
**PZ6**	MKSRVIRAAWSWRESAAHEGTRDYT	A	SER142	CB	ARG22	NH1	3.356	DI	H-bond/polar candidate
**PZ6**	MKSRVIRAAWSWRESAAHEGTRDYT	A	SER142	OG	ARG22	NH1	2.635	DI	H-bond/polar candidate
**PZ6**	MKSRVIRAAWSWRESAAHEGTRDYT	A	SER142	HG	ARG22	NH1	3.443	DI	H-bond/polar candidate
**PZ6**	MKSRVIRAAWSWRESAAHEGTRDYT	A	ARG2	CB	ARG22	HH11	3.423	DI	H-bond/polar candidate
**PZ6**	MKSRVIRAAWSWRESAAHEGTRDYT	A	ARG2	CG	ARG22	HH11	3.517	DI	H-bond/polar candidate
**PZ6**	MKSRVIRAAWSWRESAAHEGTRDYT	A	ARG2	CD	ARG22	HH11	3.303	DI	H-bond/polar candidate
**PZ6**	MKSRVIRAAWSWRESAAHEGTRDYT	A	ASP42	OD2	ARG22	HH11	3.951	DI	salt bridge candidate; H-bond/polar candidate
**PZ6**	MKSRVIRAAWSWRESAAHEGTRDYT	A	SER142	CB	ARG22	HH11	2.731	DI	H-bond/polar candidate

**PZ6**	MKSRVIRAAWSWRESAAHEGTRDYT	A	SER142	OG	ARG22	HH11	1.739	DI	H-bond/polar candidate
**PZ6**	MKSRVIRAAWSWRESAAHEGTRDYT	A	SER142	HG	ARG22	HH11	2.465	DI	H-bond/polar candidate
**PZ6**	MKSRVIRAAWSWRESAAHEGTRDYT	A	ARG2	CB	ARG22	HH12	3.252	DI	H-bond/polar candidate
**PZ6**	MKSRVIRAAWSWRESAAHEGTRDYT	A	ARG2	CG	ARG22	HH12	2.602	DI	H-bond/polar candidate
**PZ6**	MKSRVIRAAWSWRESAAHEGTRDYT	A	ARG2	CD	ARG22	HH12	2.358	DI	H-bond/polar candidate
**PZ6**	MKSRVIRAAWSWRESAAHEGTRDYT	A	ARG2	NE	ARG22	HH12	3.751	DI	H-bond/polar candidate
**PZ6**	MKSRVIRAAWSWRESAAHEGTRDYT	A	SER142	OG	ARG22	HH12	3.388	DI	H-bond/polar candidate
**PZ6**	MKSRVIRAAWSWRESAAHEGTRDYT	A	ARG164	HE	ARG22	HH12	3.868	DI	H-bond/polar candidate
**PZ6**	MKSRVIRAAWSWRESAAHEGTRDYT	A	SER142	CB	ARG22	NH2	3.285	DI	H-bond/polar candidate
**PZ6**	MKSRVIRAAWSWRESAAHEGTRDYT	A	SER142	OG	ARG22	NH2	2.93	DI	H-bond/polar candidate
**PZ6**	MKSRVIRAAWSWRESAAHEGTRDYT	A	SER142	HG	ARG22	NH2	3.764	DI	H-bond/polar candidate
**PZ6**	MKSRVIRAAWSWRESAAHEGTRDYT	A	VAL143	H	ARG22	NH2	3.984	DI	H-bond/polar candidate
**PZ6**	MKSRVIRAAWSWRESAAHEGTRDYT	A	VAL143	C	ARG22	NH2	3.839	DI	H-bond/polar candidate
**PZ6**	MKSRVIRAAWSWRESAAHEGTRDYT	A	VAL143	O	ARG22	NH2	2.792	DI	H-bond/polar candidate
**PZ6**	MKSRVIRAAWSWRESAAHEGTRDYT	A	SER146	H	ARG22	NH2	3.597	DI	H-bond/polar candidate
**PZ6**	MKSRVIRAAWSWRESAAHEGTRDYT	A	SER146	CA	ARG22	NH2	3.873	DI	H-bond/polar candidate
**PZ6**	MKSRVIRAAWSWRESAAHEGTRDYT	A	SER146	CB	ARG22	NH2	3.686	DI	H-bond/polar candidate
**PZ6**	MKSRVIRAAWSWRESAAHEGTRDYT	A	SER146	OG	ARG22	NH2	2.618	DI	H-bond/polar candidate
**PZ6**	MKSRVIRAAWSWRESAAHEGTRDYT	A	SER146	HG	ARG22	NH2	3.022	DI	H-bond/polar candidate
**PZ6**	MKSRVIRAAWSWRESAAHEGTRDYT	A	SER146	C	ARG22	NH2	3.557	DI	H-bond/polar candidate
**PZ6**	MKSRVIRAAWSWRESAAHEGTRDYT	A	ASP42	OD2	ARG22	HH21	3.918	DI	salt bridge candidate; H-bond/polar candidate
**PZ6**	MKSRVIRAAWSWRESAAHEGTRDYT	A	SER142	CA	ARG22	HH21	3.623	DI	H-bond/polar candidate
**PZ6**	MKSRVIRAAWSWRESAAHEGTRDYT	A	SER142	CB	ARG22	HH21	2.739	DI	H-bond/polar candidate
**PZ6**	MKSRVIRAAWSWRESAAHEGTRDYT	A	SER142	OG	ARG22	HH21	2.2	DI	H-bond/polar candidate
**PZ6**	MKSRVIRAAWSWRESAAHEGTRDYT	A	SER142	HG	ARG22	HH21	2.918	DI	H-bond/polar candidate
**PZ6**	MKSRVIRAAWSWRESAAHEGTRDYT	A	SER142	C	ARG22	HH21	3.415	DI	H-bond/polar candidate
**PZ6**	MKSRVIRAAWSWRESAAHEGTRDYT	A	SER142	O	ARG22	HH21	3.91	DI	H-bond/polar candidate
**PZ6**	MKSRVIRAAWSWRESAAHEGTRDYT	A	VAL143	N	ARG22	HH21	3.432	DI	H-bond/polar candidate
**PZ6**	MKSRVIRAAWSWRESAAHEGTRDYT	A	VAL143	H	ARG22	HH21	3.538	DI	H-bond/polar candidate
**PZ6**	MKSRVIRAAWSWRESAAHEGTRDYT	A	VAL143	CA	ARG22	HH21	3.966	DI	H-bond/polar candidate

**PZ6**	MKSRVIRAAWSWRESAAHEGTRDYT	A	VAL143	C	ARG22	HH21	3.22	DI	H-bond/polar candidate
**PZ6**	MKSRVIRAAWSWRESAAHEGTRDYT	A	VAL143	O	ARG22	HH21	2.393	DI	H-bond/polar candidate
**PZ6**	MKSRVIRAAWSWRESAAHEGTRDYT	A	SER146	H	ARG22	HH21	3.761	DI	H-bond/polar candidate
**PZ6**	MKSRVIRAAWSWRESAAHEGTRDYT	A	SER146	OG	ARG22	HH21	3.064	DI	H-bond/polar candidate
**PZ6**	MKSRVIRAAWSWRESAAHEGTRDYT	A	SER146	HG	ARG22	HH21	3.186	DI	H-bond/polar candidate
**PZ6**	MKSRVIRAAWSWRESAAHEGTRDYT	A	SER142	CB	ARG22	HH22	3.887	DI	H-bond/polar candidate
**PZ6**	MKSRVIRAAWSWRESAAHEGTRDYT	A	SER142	OG	ARG22	HH22	3.762	DI	H-bond/polar candidate
**PZ6**	MKSRVIRAAWSWRESAAHEGTRDYT	A	VAL143	H	ARG22	HH22	3.827	DI	H-bond/polar candidate
**PZ6**	MKSRVIRAAWSWRESAAHEGTRDYT	A	VAL143	C	ARG22	HH22	3.651	DI	H-bond/polar candidate
**PZ6**	MKSRVIRAAWSWRESAAHEGTRDYT	A	VAL143	O	ARG22	HH22	2.492	DI	H-bond/polar candidate
**PZ6**	MKSRVIRAAWSWRESAAHEGTRDYT	A	SER146	N	ARG22	HH22	3.281	DI	H-bond/polar candidate
**PZ6**	MKSRVIRAAWSWRESAAHEGTRDYT	A	SER146	H	ARG22	HH22	2.925	DI	H-bond/polar candidate
**PZ6**	MKSRVIRAAWSWRESAAHEGTRDYT	A	SER146	CA	ARG22	HH22	3.002	DI	H-bond/polar candidate
**PZ6**	MKSRVIRAAWSWRESAAHEGTRDYT	A	SER146	CB	ARG22	HH22	2.69	DI	H-bond/polar candidate
**PZ6**	MKSRVIRAAWSWRESAAHEGTRDYT	A	SER146	OG	ARG22	HH22	1.659	DI	H-bond/polar candidate
**PZ6**	MKSRVIRAAWSWRESAAHEGTRDYT	A	SER146	HG	ARG22	HH22	2.175	DI	H-bond/polar candidate
**PZ6**	MKSRVIRAAWSWRESAAHEGTRDYT	A	SER146	C	ARG22	HH22	2.946	DI	H-bond/polar candidate
**PZ6**	MKSRVIRAAWSWRESAAHEGTRDYT	A	SER146	O	ARG22	HH22	3.752	DI	H-bond/polar candidate
**PZ6**	MKSRVIRAAWSWRESAAHEGTRDYT	A	ARG164	HH11	ASP23	H	3.753	DI	salt bridge candidate; H-bond/polar candidate
**PZ6**	MKSRVIRAAWSWRESAAHEGTRDYT	A	ARG164	HH21	ASP23	H	3.569	DI	salt bridge candidate; H-bond/polar candidate
**PZ6**	MKSRVIRAAWSWRESAAHEGTRDYT	A	ARG164	HH11	ASP23	CB	3.626	DI	salt bridge candidate; H-bond/polar candidate
**PZ6**	MKSRVIRAAWSWRESAAHEGTRDYT	A	ARG164	CZ	ASP23	CG	3.943	DI	salt bridge candidate; H-bond/polar candidate
**PZ6**	MKSRVIRAAWSWRESAAHEGTRDYT	A	ARG164	NH1	ASP23	CG	3.126	DI	salt bridge candidate; H-bond/polar candidate
**PZ6**	MKSRVIRAAWSWRESAAHEGTRDYT	A	ARG164	HH11	ASP23	CG	2.115	DI	salt bridge candidate; H-bond/polar candidate
**PZ6**	MKSRVIRAAWSWRESAAHEGTRDYT	A	ARG164	HH12	ASP23	CG	3.672	DI	salt bridge candidate; H-bond/polar candidate
**PZ6**	MKSRVIRAAWSWRESAAHEGTRDYT	A	ARG164	NH2	ASP23	CG	3.81	DI	salt bridge candidate; H-bond/polar candidate
**PZ6**	MKSRVIRAAWSWRESAAHEGTRDYT	A	ARG164	HH21	ASP23	CG	3.008	DI	salt bridge candidate; H-bond/polar candidate
**PZ6**	MKSRVIRAAWSWRESAAHEGTRDYT	A	ARG164	CZ	ASP23	OD1	3.822	DI	salt bridge candidate; H-bond/polar candidate
**PZ6**	MKSRVIRAAWSWRESAAHEGTRDYT	A	ARG164	NH1	ASP23	OD1	2.693	DI	salt bridge candidate; H-bond/polar candidate
**PZ6**	MKSRVIRAAWSWRESAAHEGTRDYT	A	ARG164	HH11	ASP23	OD1	1.793	DI	salt bridge candidate; H-bond/polar candidate

**PZ6**	MKSRVIRAAWSWRESAAHEGTRDYT	A	ARG164	HH12	ASP23	OD1	2.929	DI	salt bridge candidate; H-bond/polar candidate
**PZ6**	MKSRVIRAAWSWRESAAHEGTRDYT	A	ARG164	HH21	ASP23	OD1	3.524	DI	salt bridge candidate; H-bond/polar candidate
**PZ6**	MKSRVIRAAWSWRESAAHEGTRDYT	A	ARG164	CZ	ASP23	OD2	3.227	DI	salt bridge candidate; H-bond/polar candidate
**PZ6**	MKSRVIRAAWSWRESAAHEGTRDYT	A	ARG164	NH1	ASP23	OD2	2.808	DI	salt bridge candidate; H-bond/polar candidate
**PZ6**	MKSRVIRAAWSWRESAAHEGTRDYT	A	ARG164	HH11	ASP23	OD2	1.922	DI	salt bridge candidate; H-bond/polar candidate
**PZ6**	MKSRVIRAAWSWRESAAHEGTRDYT	A	ARG164	HH12	ASP23	OD2	3.618	DI	salt bridge candidate; H-bond/polar candidate
**PZ6**	MKSRVIRAAWSWRESAAHEGTRDYT	A	ARG164	NH2	ASP23	OD2	2.774	DI	salt bridge candidate; H-bond/polar candidate
**PZ6**	MKSRVIRAAWSWRESAAHEGTRDYT	A	ARG164	HH21	ASP23	OD2	1.864	DI	salt bridge candidate; H-bond/polar candidate
**PZ6**	MKSRVIRAAWSWRESAAHEGTRDYT	A	ARG164	HH22	ASP23	OD2	3.606	DI	salt bridge candidate; H-bond/polar candidate
**PZ6**	MKSRVIRAAWSWRESAAHEGTRDYT	A	LYS316	HZ1	TYR24	CE1	3.851	DIII	H-bond/polar candidate
**PZ6**	MKSRVIRAAWSWRESAAHEGTRDYT	A	LYS316	NZ	TYR24	CE2	3.822	DIII	H-bond/polar candidate
**PZ6**	MKSRVIRAAWSWRESAAHEGTRDYT	A	LYS316	HZ1	TYR24	CE2	2.804	DIII	H-bond/polar candidate
**PZ6**	MKSRVIRAAWSWRESAAHEGTRDYT	A	LYS316	NZ	TYR24	CZ	3.593	DIII	H-bond/polar candidate
**PZ6**	MKSRVIRAAWSWRESAAHEGTRDYT	A	LYS316	HZ1	TYR24	CZ	2.575	DIII	H-bond/polar candidate
**PZ6**	MKSRVIRAAWSWRESAAHEGTRDYT	A	LYS316	HZ3	TYR24	CZ	3.903	DIII	H-bond/polar candidate
**PZ6**	MKSRVIRAAWSWRESAAHEGTRDYT	A	MET375	CE	TYR24	CZ	3.948	DIII	H-bond/polar candidate; hydrophobic/van der Waals
**PZ6**	MKSRVIRAAWSWRESAAHEGTRDYT	A	LYS316	CE	TYR24	OH	3.022	DIII	H-bond/polar candidate
**PZ6**	MKSRVIRAAWSWRESAAHEGTRDYT	A	LYS316	NZ	TYR24	OH	2.586	DIII	H-bond/polar candidate
**PZ6**	MKSRVIRAAWSWRESAAHEGTRDYT	A	LYS316	HZ1	TYR24	OH	1.735	DIII	H-bond/polar candidate
**PZ6**	MKSRVIRAAWSWRESAAHEGTRDYT	A	LYS316	HZ2	TYR24	OH	3.438	DIII	H-bond/polar candidate
**PZ6**	MKSRVIRAAWSWRESAAHEGTRDYT	A	LYS316	HZ3	TYR24	OH	2.848	DIII	H-bond/polar candidate
**PZ6**	MKSRVIRAAWSWRESAAHEGTRDYT	A	MET375	CE	TYR24	OH	3.335	DIII	H-bond/polar candidate; hydrophobic/van der Waals
**PZ6**	MKSRVIRAAWSWRESAAHEGTRDYT	A	LYS316	CE	TYR24	HH	3.442	DIII	H-bond/polar candidate
**PZ6**	MKSRVIRAAWSWRESAAHEGTRDYT	A	LYS316	NZ	TYR24	HH	3.208	DIII	H-bond/polar candidate
**PZ6**	MKSRVIRAAWSWRESAAHEGTRDYT	A	LYS316	HZ1	TYR24	HH	2.533	DIII	H-bond/polar candidate
**PZ6**	MKSRVIRAAWSWRESAAHEGTRDYT	A	LYS316	HZ3	TYR24	HH	3.259	DIII	H-bond/polar candidate
**PZ6**	MKSRVIRAAWSWRESAAHEGTRDYT	A	MET375	CE	TYR24	HH	3.075	DIII	H-bond/polar candidate; hydrophobic/van der Waals
**PZ6**	MKSRVIRAAWSWRESAAHEGTRDYT	A	GLU329	OE2	THR25	CB	3.81	DIII	H-bond/polar candidate
**PZ6**	MKSRVIRAAWSWRESAAHEGTRDYT	A	MET375	CE	THR25	CB	3.97	DIII	H-bond/polar candidate
**PZ6**	MKSRVIRAAWSWRESAAHEGTRDYT	A	LYS316	NZ	THR25	OG1	3.84	DIII	H-bond/polar candidate

**PZ6**	MKSRVIRAAWSWRESAAHEGTRDYT	A	LYS316	HZ1	THR25	OG1	3.259	DIII	H-bond/polar candidate
**PZ6**	MKSRVIRAAWSWRESAAHEGTRDYT	A	LYS316	HZ2	THR25	OG1	3.977	DIII	H-bond/polar candidate
**PZ6**	MKSRVIRAAWSWRESAAHEGTRDYT	A	LYS316	HZ3	THR25	OG1	3.62	DIII	H-bond/polar candidate
**PZ6**	MKSRVIRAAWSWRESAAHEGTRDYT	A	GLU329	OE2	THR25	OG1	2.948	DIII	H-bond/polar candidate
**PZ6**	MKSRVIRAAWSWRESAAHEGTRDYT	A	MET375	CE	THR25	OG1	3.238	DIII	H-bond/polar candidate
**PZ6**	MKSRVIRAAWSWRESAAHEGTRDYT	A	LYS316	NZ	THR25	HG1	3.444	DIII	H-bond/polar candidate
**PZ6**	MKSRVIRAAWSWRESAAHEGTRDYT	A	LYS316	HZ1	THR25	HG1	3.029	DIII	H-bond/polar candidate
**PZ6**	MKSRVIRAAWSWRESAAHEGTRDYT	A	LYS316	HZ2	THR25	HG1	3.353	DIII	H-bond/polar candidate
**PZ6**	MKSRVIRAAWSWRESAAHEGTRDYT	A	LYS316	HZ3	THR25	HG1	3.284	DIII	H-bond/polar candidate
**PZ6**	MKSRVIRAAWSWRESAAHEGTRDYT	A	GLU329	CG	THR25	HG1	3.945	DIII	H-bond/polar candidate
**PZ6**	MKSRVIRAAWSWRESAAHEGTRDYT	A	GLU329	CD	THR25	HG1	3.191	DIII	H-bond/polar candidate
**PZ6**	MKSRVIRAAWSWRESAAHEGTRDYT	A	GLU329	OE2	THR25	HG1	2.044	DIII	H-bond/polar candidate
**PZ6**	MKSRVIRAAWSWRESAAHEGTRDYT	A	LYS373	HZ2	THR25	HG1	3.421	DIII	H-bond/polar candidate
**PZ6**	MKSRVIRAAWSWRESAAHEGTRDYT	A	MET375	CE	THR25	HG1	3.648	DIII	H-bond/polar candidate
**PZ6**	MKSRVIRAAWSWRESAAHEGTRDYT	A	GLU329	OE2	THR25	CG2	3.604	DIII	H-bond/polar candidate
**PZ6**	MKSRVIRAAWSWRESAAHEGTRDYT	A	LYS373	C	THR25	CG2	3.765	DIII	H-bond/polar candidate
**PZ6**	MKSRVIRAAWSWRESAAHEGTRDYT	A	LYS373	O	THR25	CG2	3.646	DIII	H-bond/polar candidate
**PZ6**	MKSRVIRAAWSWRESAAHEGTRDYT	A	MET374	N	THR25	CG2	3.92	DIII	H-bond/polar candidate
**PZ6**	MKSRVIRAAWSWRESAAHEGTRDYT	A	MET374	CA	THR25	CG2	3.971	DIII	H-bond/polar candidate
**PZ6**	MKSRVIRAAWSWRESAAHEGTRDYT	A	LYS373	O	THR25	C	3.949	DIII	H-bond/polar candidate
**PZ6**	MKSRVIRAAWSWRESAAHEGTRDYT	A	LYS373	N	THR25	O	3.945	DIII	H-bond/polar candidate
**PZ6**	MKSRVIRAAWSWRESAAHEGTRDYT	A	LYS373	H	THR25	O	3.271	DIII	H-bond/polar candidate
**PZ6**	MKSRVIRAAWSWRESAAHEGTRDYT	A	LYS373	CA	THR25	O	3.901	DIII	H-bond/polar candidate
**PZ6**	MKSRVIRAAWSWRESAAHEGTRDYT	A	LYS373	CB	THR25	O	3.4	DIII	H-bond/polar candidate
**PZ6**	MKSRVIRAAWSWRESAAHEGTRDYT	A	LYS373	C	THR25	O	3.84	DIII	H-bond/polar candidate
**PZ6**	MKSRVIRAAWSWRESAAHEGTRDYT	A	LYS373	O	THR25	O	3.335	DIII	H-bond/polar candidate
